# Metal-free photochemical silylations and transfer hydrogenations of benzenoid hydrocarbons and graphene

**DOI:** 10.1038/ncomms12962

**Published:** 2016-10-06

**Authors:** Raffaello Papadakis, Hu Li, Joakim Bergman, Anna Lundstedt, Kjell Jorner, Rabia Ayub, Soumyajyoti Haldar, Burkhard O. Jahn, Aleksandra Denisova, Burkhard Zietz, Roland Lindh, Biplab Sanyal, Helena Grennberg, Klaus Leifer, Henrik Ottosson

**Affiliations:** 1 Department of Chemistry—Ångström Laboratory, Box 523, 751 20 Uppsala, Sweden; 2 Department of Chemistry—BMC, Uppsala University, Box 576, 751 23 Uppsala, Sweden; 3 Department of Engineering Sciences, Applied Materials Science, Uppsala University, Box 534, 751 21 Uppsala, Sweden; 4 AstraZeneca R&D Mölndal, Medicinal Chemistry-KH471, 43183 Mölndal, Sweden; 5 Department of Physics and Astronomy, Uppsala University, Box 516, 751 20 Uppsala, Sweden; 6 Department of Chemistry—Ångström Laboratory, Box 523, 751 20 Uppsala, Sweden; 7 Uppsala Center for Computational Chemistry—UC_3_, Uppsala University, Box 523, 751 20 Uppsala, Sweden

## Abstract

The first hydrogenation step of benzene, which is endergonic in the electronic ground state (S_0_), becomes exergonic in the first triplet state (T_1_). This is in line with Baird's rule, which tells that benzene is antiaromatic and destabilized in its T_1_ state and also in its first singlet excited state (S_1_), opposite to S_0_, where it is aromatic and remarkably unreactive. Here we utilized this feature to show that benzene and several polycyclic aromatic hydrocarbons (PAHs) to various extents undergo metal-free photochemical (hydro)silylations and transfer-hydrogenations at mild conditions, with the highest yield for naphthalene (photosilylation: 21%). Quantum chemical computations reveal that T_1_-state benzene is excellent at H-atom abstraction, while cyclooctatetraene, aromatic in the T_1_ and S_1_ states according to Baird's rule, is unreactive. Remarkably, also CVD-graphene on SiO_2_ is efficiently transfer-photohydrogenated using formic acid/water mixtures together with white light or solar irradiation under metal-free conditions.

## Abstract (web summary)

Baird's rules say that the first triplet state of benzene displays antiaromatic character. Here, the authors exploit this to show that aromatic molecules can undergo rapid transfer hydrogenation or silylations without the need for metal catalysts when photochemcially excited into this state.

## Graphical abstract

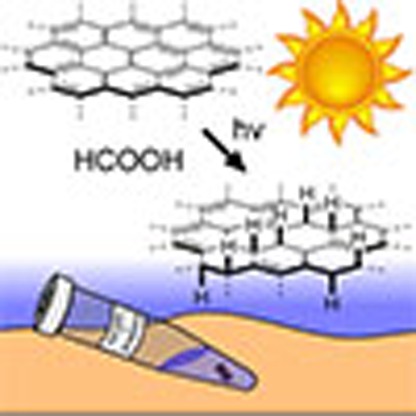

Aromaticity is a core concept in organic chemistry^1^,^2^, yet, it is essentially only used to rationalize properties and processes in the S_0_ state. In 1972, Baird applied perturbation molecular orbital theory to show that annulenes with 4*n*+2 *π*-electrons in their lowest ππ* triplet states are destabilized (antiaromatic) relative to two polyenyl monoradicals while annulenes with 4*n π*-electrons are stabilized (aromatic)^3^,^4^. The rule, which is opposite to Hückel's rule for the S_0_ state^5^,^6^, has been verified by numerous high-level quantum chemical calculations^7^,^8^, and it also applies to the S_1_ states of cyclobutadiene, benzene and cyclooctatetraene (COT)^9^,^10^,^11^. Wan and co-workers pioneered the experimental development of excited state aromaticity when reporting the involvement of aromatic 4*π* cationic species in the photosolvolysis of fluoren-9-ol and photoreactions leading to 4*nπ*-electron arenes^12^,^13^,^14^. Later, Baird's rule was utilized by Ottosson and co-workers to rationalize experimentally determined T_1_- and S_1_-state energies of substituted fulvenes^15^,^16^. Indeed, excited-state aromaticity and antiaromaticity can be used to explain a range of earlier photophysical and photochemical observations^8^,^17^.

Recently, spectroscopic evidence for Baird's rule in the T_1_ state was provided by Kim and co-workers^18^, who investigated two *bis*-rhodium hexaphyrins of which one is aromatic and the other is antiaromatic in the S_0_ state. In their T_1_ states, the hexaphyrins interchanged the absorption spectral characteristics, and computed aromaticity indices revealed that this change could be connected to an aromaticity/antiaromaticity reversal when going from the S_0_ to the T_1_ state. A similar spectral switch was recently observed for another pair of hexaphyrins on excitation to S_1_ (ref. 19). Tovar and co-workers also reported on a tetracyano-substituted quinoidal methano[10]annulenic species with a low-lying triplet state^20^, belonging to a compound class that can be described as Hückel–Baird hybrids^21^. Furthermore, two of us reviewed theoretical/computational findings on the T_1_ and S_1_ antiaromatic character of benzene jointly with findings on various photoreactions of benzene derivatives, concluding that excited state antiaromaticity triggers photoreactivity^17^. With reference to the ‘Dr Jekyll and Mr Hyde' split personality drama^22^, benzene in its antiaromatic first excited states was labelled a molecular ‘Mr Hyde' while benzene in the S_0_ state is a molecular ‘Dr Jekyll'^8^,^17^. Indeed, both Baird and Aihara postulated from theory that antiaromaticity drastically increases the reactivity of benzene in its first excited states^3^,^23^, and Alabugin and co-workers recently utilized this feature to rationalize the photochemically initiated C_1_–C_5_ cycloaromatization reaction of benzannelated enynes to benzofulvenes^24^. In short, recognition of the antiaromatic destabilization of benzene should be useful for the development of new photoreactions.

The first hydrogenation step of benzene in S_0_ is endothermic due to aromaticity loss, contrary to the hydrogenation of alkenes and acyclic polyenes. Hence, the hydrogenation of arenes typically progresses only with metal catalysts under high pressures and/or high temperatures. Yet, in light of Baird's rule, hydrogenations and other formal *σ*-bond additions to benzene should be considerably more facile in the T_1_ and S_1_ states than in the S_0_ state. A few such additions to arenes have earlier been observed, but none were linked to relief of excited state antiaromaticity. Low-temperature (−196 °C) solid-state photoreactions of small polycyclic aromatic hydrocarbons (PAHs) with aliphatic hydrocarbons, yielding alkylated or hydroxyalkylated products as well as dihydrogenated aromatics, were reported.^25^,^26^ For mixed durene–naphtalene crystals, variations in phosphorescence decay rates with temperature, pressure and deuterium labelling were attributed to hydrogen abstraction from durene by triplet-state naphthalene, leading to naphthyl radicals^27^. Acenaphtylene, benz[*a*]anthracene and phenanthrene in benzene solution were photoreduced by tri-*n*-butyltin hydride to dihydrogenated PAHs^28^. Yet, at room temperature small PAHs only photoreacted with alcohols or alkanes when very high pressures were applied^29^, although Jeevarajan and Fessenden showed by electron paramagnetic resonace spectroscopy at ambient temperature and pressure that benzoic acid derivatives in their ππ* states, that is, with the benzene ring as the photoreactive moiety, abstract hydrogen from *iso*-propanol to yield transient benzenium (cyclohexadienyl) radicals^30^. Combined with Baird's rule and the computationally verified excited-state antiaromatic character of benzene^8^,^17^, these studies point to a largely underexploited area of benzene photochemistry that could provide access to formal *σ*-bond photoadditions (stepwise or concerted) to benzene, PAHs and graphene under mild metal-free conditions.

Herein, we present computational and experimental results that show that our basic hypothesis is correct. According to computations, the first photohydrogenation step of benzene is exergonic while that of COT is endergonic. Metal-free photoreactions with triethylsilane, used as a heavy ‘dihydrogen', gave silylated products with the highest yield for naphthalene (21% combined yield of the two triethylsilylnaphthalenes and 6% for triethylsilylbenzene). Experiments as well as computations indicate that the photoreactions progress via the T_1_ state. Yet, large PAHs were photo(hydro)silylated, and also transfer-photohydrogenated, to gradually lower degrees with increased size. Thus, it is remarkable that graphene on SiO_2_ was efficiently transfer-photohydrogenated and photo(hydro)silylated on white light or solar irradiation. Two tentative explanations involving excited-state antiaromaticity for the graphene photoreactions are given, yet, these reactions may also be outside the realm of excited state antiaromaticity alleviation.

## Results

### Computational assessment of the hypothesis

**Figure 1:**
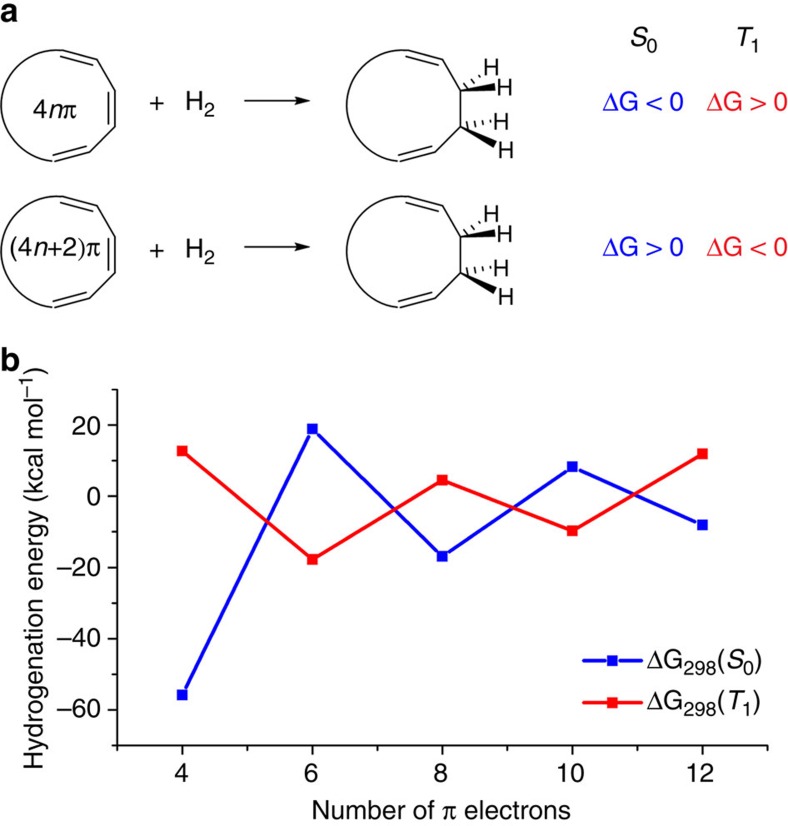
Hypothesis and computed reaction energies for the first step in the S_0_- and T_1_-state hydrogenation of annulenes. (**a**) Schematics of our hypothesis that exergonicity/endergonicity of the first hydrogenation step reverses when going from the S_0_ to the T_1_ state, both for [4*n*]- and [4*n*+2]annulenes. (**b**) Calculated average free energies of the first hydrogenation reaction at (U)B3LYP/6-311+G(d,p) level for a selection of annulenes (neutral, cationic and anionic) with 4, 6, 8, 10 and 12 *π*-electrons. For electronic energies, see Supplementary Tables 3–7.

We argue that the first hydrogenations of [4*n*+2]annulenes are considerably more facile (possibly exothermic and exergonic) in the T_1_ than in the S_0_ state, and the opposite for [4*n*]annulenes (Fig. 1a) reflecting that antiaromaticity is alleviated in the first while aromaticity is lost in the latter. Hence, in both the S_0_ and T_1_ states, there should be zigzag variations in the first hydrogenation energies for each additional *π*-bond in the annulene series, however, our hypothesis is that the sense in the zigzag relationships is opposite in the two states.

Neutral, cationic and anionic annulenes with *m*=4, 6, 8, 10 and 12 *π*-electrons, all with (near-)planar structures in both the S_0_ and T_1_ states, were examined computationally. The number of hydrogenation reactions regarded for each *m* range from 2 (*m*=4) to 31 (*m*=12) (Supplementary Figs 95–97). Clear zigzag patterns in the first hydrogenation energies at (U)B3LYP/6-311+G(d,p) level were evident in both states (in Fig. 1b displayed as the average first hydrogenation energies for each *m*). Importantly, the sense in the zigzag relationships on the number of *π*-electrons is opposite in the T_1_ and S_0_ states, confirming our hypothesis. In particular, the first hydrogenation step of T_1_-state benzene at 298 K, leading to cyclohexa-1,3-diene, is exergonic by 17.7 kcal mol^−1^ while this reaction for COT in the T_1_ state is endergonic by 18.6 kcal mol^−1^. Thus, T_1_-state COT takes the position of benzene in the S_0_ state and benzene in the T_1_ state takes that of COT in the S_0_ state.

### Motivations for experimental set-up and reagents used

We considered direct photohydrogenation with dihydrogen too hazardous experimentally and turned to (i) metal-free photohydrosilylations using triethylhydrosilane (Et_3_SiH) as a heavy ‘dihydrogen', and (ii) metal-free transfer photohydrogenations with formic acid (HCOOH). The photohydrosilylation reactions were mainly performed in a Rayonet photoreactor at ambient conditions with lamp emission at 254 (4.88 eV) or 300 nm (4.13 eV); however, for some experiments an immersion well photoreactor with a Hg lamp was used or a Fluorolog-3-22 instrument equipped with a Xe lamp as a light source allowing for monochromatic irradiation. The trialkylmonosilane selected absorbs only in the vacuum–ultraviolet region with a strong excitation measured at 151 nm (8.21 eV; Supplementary Fig. 29)^31^, and the first transition (a nearly forbidden transition) calculated at 181 nm (6.84 eV) according to TD-B3LYP (Supplementary Methods). Thus, photodissociation of triethylsilane into silyl radicals is not feasible (for experiments disproving free radical formation through photolysis of Et_3_SiH, see Supplementary Figs 30–40 and the corresponding discussion). Moreover, HCOOH shows a weak excitation at 215 nm (5.77 eV), extending until ∼250 nm/4.96 eV, but photodecomposition into CO and H_2_O, and into CO_2_ and H_2_, occurs only with *λ*<260 nm (ref. 32). Our photoreactions using HCOOH were performed at *λ*≥300 nm.

### On the metal-free photosilylations of benzene versus COT

**Figure 2:**
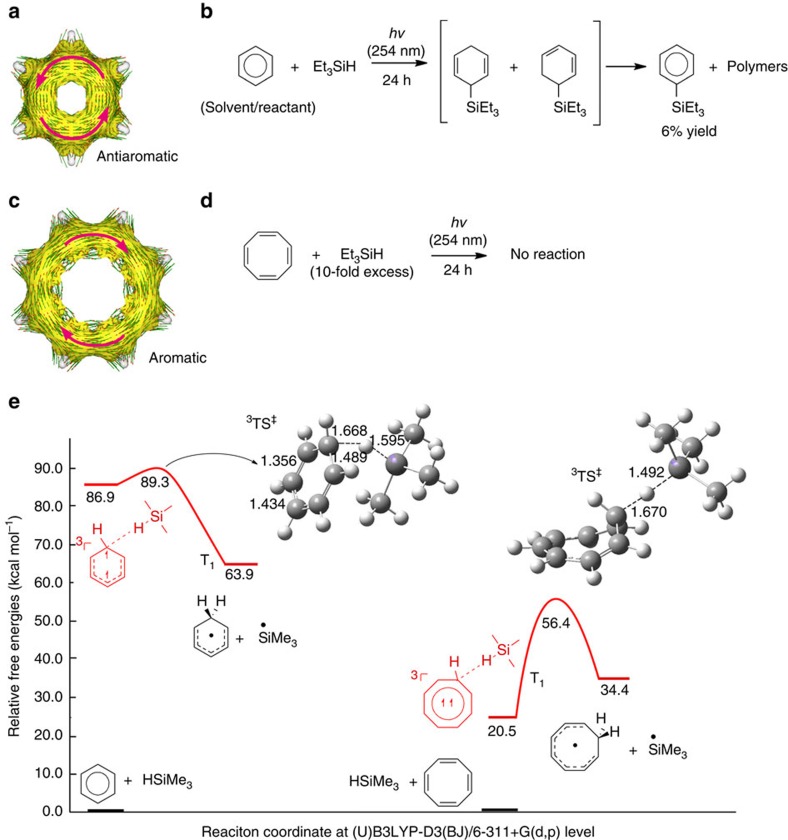
Photosilylation of benzene and attempted photosilylation of cyclooctatetraene. (**a**) ACID plot of benzene in its antiaromatic T_1_ state with the direction of the paramagnetic ring current indicated by arrows. (**b**) Scheme showing the photoreaction of benzene with Et_3_SiH leading to two isomeric hydrosilylated intermediates, and subsequently, to phenyltriethylsilane and polymeric products. (**c**) ACID plot of COT in its aromatic T_1_ state with the direction of the diamagnetic ring current indicated by arrows. (**d**) Scheme showing that COT remains unreacted when irradiated in 10-fold excess of Et_3_SiH for 24 h. (**e**) Potential energy curves for H-atom abstraction from Me_3_SiH by T_1_-state benzene and by T_1_-state COT, respectively. Insets: transition-state structures (bond distances in Å).

When a benzene solution of triethylhydrosilane was irradiated at *λ*=254 nm, several different products were formed; the most significant soluble non-polymeric product being phenyltriethylsilane obtained in 6% isolated yield. This product forms via a route that involves triethylsilylcyclohexadiene intermediates (Fig. 2b), compounds identified in transient quantities by gas chromatography–mass spectrometry (GC–MS). Yet, the major photoproduct is insoluble polymers, which could form along a few different routes (Supplementary Fig. 109). When the photoreaction was carried out with an aerated benzene solution of triethylsilane, no polymers were formed. However, the GC–MS conversion to phenyltriethylsilane was significantly reduced (∼0.35%), and instead the formation of siloxanes was observed (Supplementary Fig. 46).

So what is the mechanism for triethylsilane photoaddition to benzene, and does excited state antiaromaticity relief play a role? The photoreactivity of COT towards Et_3_SiH is elucidating: contrary to benzene, COT is recovered unchanged after 24 h of 254 nm irradiation (Fig. 2d). Computations with Me_3_SiH as a model for Et_3_SiH reveal that benzene in its T_1_ state is excellent at H-atom abstraction (while T_1_-state COT is the opposite) because the activation free energy for the reaction leading from a preceding van der Waals complex to the benzenium and trimethylsilyl radicals at B3LYP-D3(BJ)/6-311+G(d,p) level is merely 2.4 kcal mol^−1^ (Fig. 2e; for CCSD(T) results see Supplementary Fig. 101). The activation energy for H-atom abstraction from Me_3_SiH by the tert-butoxy radical, a well-known radical photoinitiator^33^, is 8.2 kcal mol^−1^, whereas H-atom abstraction by T_1_-state COT requires 35.9 kcal mol^−1^ (Fig. 2e) and by cycloocta-1,3,5-triene, a T_1_ nonaromatic linearly conjugated triene, requires 15.5 kcal mol^−1^. Thus, the T_1_ potential energy surface for H-atom abstraction from Me_3_SiH by T_1_-state benzene unambiguously support our experimental observation. Moreover, the differences in degree of (anti)aromaticity between the isolated T_1_-state benzene and COT, and the corresponding transition-state structures, as well as annulenium product radicals (see the Supplementary Information), reveal that T_1_ antiaromaticity is alleviated in the reaction for benzene while T_1_ aromaticity is lost for COT, showcasing the predictive power of Baird's rule.

In support of our conclusion that the reaction progresses in the T_1_ state, the activation energy for H-atom abstraction from Me_3_SiH by S_1_-state benzene, calculated with CASPT2//CASSCF, is 27.2 kcal mol^−1^ relative to isolated reactants, 20 kcal mol^−1^ higher than in the T_1_ state. Formation of the triethylsilylcyclohexadiene intermediates should occur after spin-flip of the triplet benzenium and triethylsilyl radical pair to a singlet radical pair, which according to our calculations (CASPT2 energies on UB3LYP geometries) progresses without activation barrier leading to a gain in free energy of 39.6 kcal mol^−1^. The polymers that are observed as major product could form along a variety of pathways: (i) the silylcyclohexa-1,4-diene intermediates photopolymerize as confirmed by irradiation of pre-prepared triethylsilylcyclohexa-1,4-diene (Supplementary Figs 52–54 and the corresponding discussion); (ii) the silyl radicals formed could initiate radical chain processes through addition with benzene, although the free energy of activation for this process (14.8 kcal mol^−1^ with UB3LYP-D3) represents a substantial barrier in contrast to the barrierless and exergonic collapse of the singlet benzenium and triethylsilyl radical pair; and finally (iii) benzene when excited could undergo photocycloaddition reactions with the silylcyclohexadiene intermediates. Yet, the yield for the photohydrosilylation step could be higher than that observed for phenyltriethylsilane formation. It can also be noted that formations of triethylsilylcyclohexadienes are endergonic reactions in S_0_ (21.6 kcal mol^−1^ for formation of the cyclohexa-1,3-diene and 20.7 kcal mol^−1^ for formation of the cyclohexa-1,4-diene).

### T_1_-state antiaromaticity of PAHs

**Figure 3:**
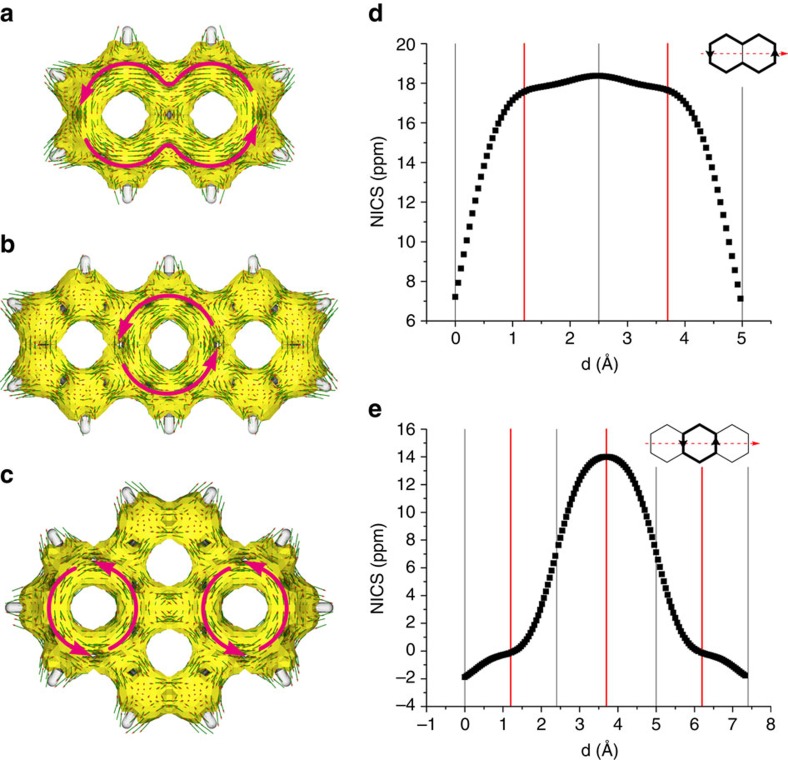
T_1_-state antiaromatic character of small PAHs. ACID plots of naphthalene (**a**), anthracene (**b**) and pyrene (**c**) in their T_1_ states, with the directions of the paramagnetic ring currents indicated by magenta arrows, as well as NICS-XY scans for naphthalene (**d**) and anthracene (**e**) in their T_1_ states. Calculations were performed at B3LYP/6-311+G(d,p) level.

The T_1_ antiaromaticity of benzene, naphthalene, anthracene and larger PAHs was analysed through ACID plots (Anisotropy of the Induced Current Density) (Supplementary Figs 111–117 and Supplementary Methods: Computational part), a method to visualize electron delocalization and ring currents, and two-dimensional (2D) NICS scans, NICS-XY (Nucleus Independent Chemical Shifts). Focus on the T_1_ antiaromaticity in the computations is justified by the high intersystem crossing rates of PAHs and the longer T_1_- than S_1_-state lifetimes. In the T_1_ state, benzene, naphthalene, anthracene and pyrene all display paramagnetic ring currents typical for antiaromaticity (Figs 2a and 3). Importantly, antiaromaticity is present even though the T_1_ structures undergo geometry relaxations. Yet, while the antiaromatic ring current is global for benzene and naphthalene, it is localized to the central ring in anthracene and to two of the four rings in pyrene, revealing that in larger PAHs the T_1_ antiaromaticity is not distributed over all rings (Fig. 3). The results from this analysis is further validated by the NICS(1)_zz_ values (Supplementary Fig. 125). The antiaromatic character of the PAHs based on the magnetic indices also agree with an earlier graph theoretical study using Hückel molecular orbital theory which revealed substantial destabilization of PAHs in their first ππ* states.^34^ A recent CASPT2 study of electronic aromaticity indices of small PAHs further confirm that they lose aromaticity upon excitation to the S_1_ state.^35^

### Metal-free photoreactions of PAHs

**Figure 4:**
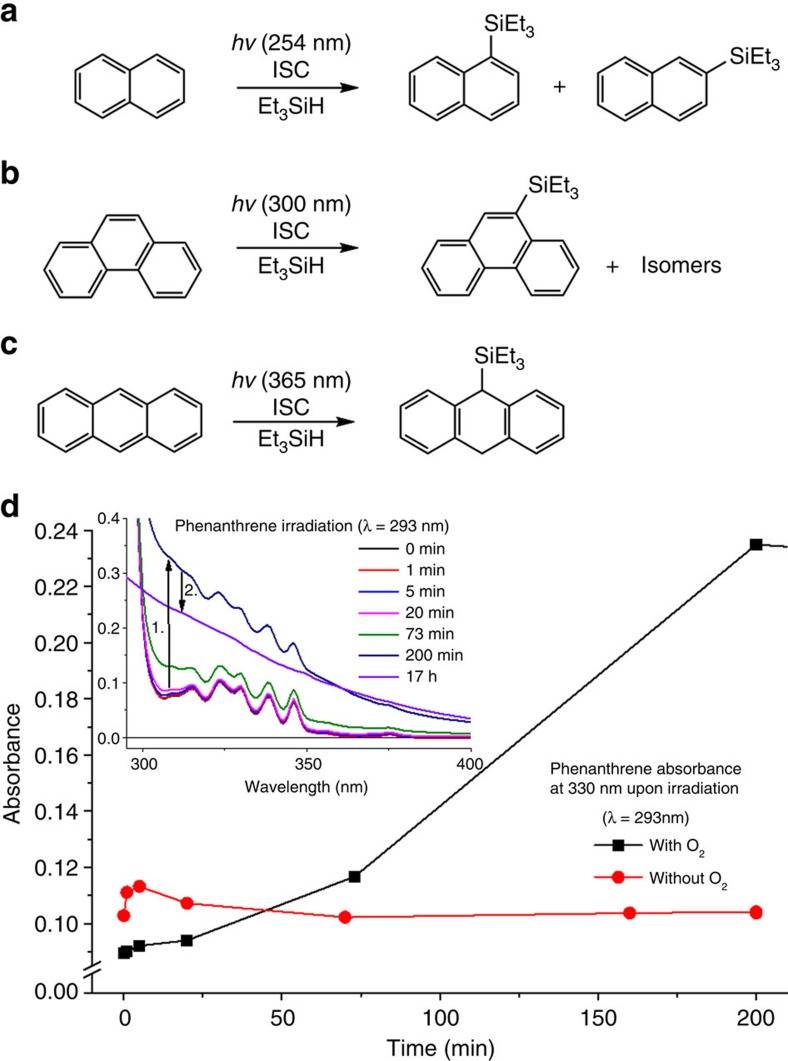
Experimental results on metal-free photosilylations of small PAHs. (**a**) Scheme showing the photoreaction of naphthalene with Et_3_SiH and the two isomeric monosilylated products (irradiation at 254 nm in *n*-heptane). (**b**) Scheme describing the photoreaction of phenanthrene with Et_3_SiH and the major silylated product (irradiation at 300 nm in *n*-heptane). (**c**) Scheme describing the photoreaction of anthracene with Et_3_SiH and the hydrosilylated product (irradiation at 365 nm in benzene). (**d**) Changes in phenanthrene absorbance at 330 nm versus irradiation time (*λ*_ex_=293 nm) in the presence and absence of O_2_. The inset shows the changes in the absorbance spectra over time.

Naphthalene was irradiated at *λ*=254 nm in *n*-heptane with an excess of Et_3_SiH (Fig. 4a), leading to an approximate 1:1 mixture of the two isomeric triethylsilylnaphthalenes in 21% combined yield. In addition, traces of two isomeric hydrosilylated naphthalenes were identified through GC–MS (Supplementary Fig. 59). As naphthalene has a high intersystem crossing rate, the H-atom abstraction from Et_3_SiH should involve T_1_-state naphthalene (*vide infra*). The calculated activation energies for this process are higher (14.3 kcal mol^−1^ for transition state leading to the 1-naphthalenium radical and 18.5 kcal mol^−1^ for that leading to the 2-naphthalenium radical) than for benzene. Yet, although the barriers are high one may infer that tunnelling plays a role as earlier concluded for hydrogen abstraction in the mixed naphthalene–durene crystals^27^.

Phenanthrene in *n*-heptane gave three different products in a combined conversion of ∼11%, corresponding to monosilylated phenanthrenes with the highest conversion (7%) to 9-triethylsilylphenantrene (Fig. 4b). Noteworthy, phenanthrene, which is known to not form excimers^36^, is photoreactive (activation energy for H-atom abstraction: 15.5 kcal mol^−1^). Interestingly, while the calculated activation barriers for the H-atom abstraction by naphthalene and phenantrene in the T_1_ states are much higher than that for benzene the yields/conversions for the photosilylations of the first two compounds are higher. This suggests that the initial yields of triethylsilylcyclohexadienes are higher than reflected in the low yield of phenyltriethylsilane and that the triethylsilylcyclohexadienes involve in photopolymerizations.

At this point we carried out independent irradiation experiments with a Xe lamp under strictly anaerobic conditions to test if (i) light from other Hg lines (especially short wavelength) is involved in the product formation and (ii) if the strict exclusion of oxygen yields the same products as previous experiments (Supplementary Figs 46–49, 75–76 and corresponding discussion). Benzene and phenanthrene were irradiated at 254 and 293 nm, respectively. Silylated products were formed in all cases. In aerated samples of benzene and phenanthrene, less amount of silylated products was formed and phenanthrene degraded faster due to side reactions compared with the absence of O_2_ (Fig. 4d). A fast rise due to the formation of degradation products is followed by a decrease of absorbance due to secondary reactions, mainly after 200 min.

Pyrene, fluoranthene and coronene were dissolved in benzene in the presence of an excess of Et_3_SiH, and irradiated with *λ*=300 nm. Anthracene, on the other hand, was irradiated in benzene with *λ*=365 nm, and at this wavelength the outcome of the photoreaction was not affected by benzene. According to ^1^H nuclear magnetic resonance experiments the anthracene photodimer^37^ was the major product and 9-(triethylsilyl)-9,10-dihydroanthracene was formed in a GC–MS yield of 4.5%. Fluoranthene gave traces of monosilylated products according to GC–MS, while pyrene and coronene were unreactive.

Clearly, several factors lower the photoreactivities of the PAHs towards Et_3_SiH when compared with benzene. First, the computed activation energies for H-atom abstraction from HSiMe_3_ increase gradually with the size of the PAHs (for pyrene in the T_1_ state it is 28.1 kcal mol^−1^, in line with pyrene being unreactive) and are higher than non-aromatic cycloocta-1,3,5-triene (15.5 kcal mol^−1^), showing that other factors than excited-state antiaromaticity relief also influence the activation barriers and reactivities of larger T_1_-state PAHs. Moreover, excimers of PAHs could form, and recent computational studies reveal such excimers to display less C–C bond length alternation than monomeric PAHs in their S_1_ and T_1_ states^38^,^39^, suggesting reduced antiaromaticity. Excimer-binding energies, calculated to 8–22 kcal mol^−1^ in the T_1_ state and 17–39 kcal mol^−1^ in the S_1_ state for benzene, naphthalene and anthracene, are potentially associated with loss of antiaromaticity leading to lowered reactivity.

Transfer photohydrogenations of PAHs with excess of formic acid (HCOOH) as the transfer hydrogenation agent were carried out in benzene/ethyl acetate (1:1 volumetric ratio). Benzene and naphthalene were excluded from these experiments since HCOOH photodecomposes at *λ*<260 nm (ref. 32). Among the PAHs investigated, phenanthrene was converted to 9,10-dihydrophenanthrene in 3–4% conversion (determined through GC–MS; Supplementary Fig. 25), while anthracene gave 9,10-dihydroanthracene in 4–5% yield together with anthracene dimer, the latter as main product. In contrast to the observation from the hydrosilylation experiments, pyrene reacted to give a low yield of 4,5-dihydropyrene, while fluoranthene and coronene again were unreactive. The observed selectivity of photohydrogenation and photohydrosilylation is mostly consistent with the patterns of T_1_ antiaromaticity given by ACID plots (Supplementary Figs 112–117), but other factors besides excited-state antiaromaticity also seem to control the selectivity, likely hydrogen migrations to optimize aromaticity on return to S_0_.

### On the potential T_1_-state antiaromaticity of graphene

As the larger PAHs display very poor or no photoreactivity in our experiments, one may infer that the infinitely large PAH, graphene, would be unreactive. However, earlier computations of NICS and electron sharing indices, as well as calculations with the adaptive natural density partitioning method reveal that graphene aromaticity, in contrast to that of PAHs, is local with two *π*-electrons over every hexagon^40^. Owing to the zero bandgap, an excited-state continuum is accessible with infinitesimal excitation energies, making it impossible to determine the influence of excited-state antiaromaticity. In addition, if applicable to these states, the antiaromatic character could be dispersed in the infinite 2D sheet and hence become exceptionally weak unless there are means for exciton and excited-state antiaromaticity localization. Moreover, graphene quantum dots (large PAHs) were found to display high intersystem crossing rates to long-lived triplet states (4 μs)^41^, possibly enabling sequential multi-photon excitations and thus reactivity from higher (triplet) states. Although the triplet states of graphene undoubtedly are complex, two features connected to triplet-state antiaromaticity can be important for the understanding of graphene photochemistry. First, for PAHs such as anthracene, pyrene, chrysene and triphenylene, T_1_ antiaromaticity localizes to a single or a few rings (Fig. 3 and Supplementary Figs 113, 116, 119 and 120). For graphene this could imply that triplet-state antiaromaticity localizes to edges having, for example, armchair segments. Second, Stone–Wales defects, composed of two 5–7 defects in antiparallel orientation^42^,^43^, could play a role. Although the Stone–Wales defect has a 14*π*-electron perimeter, which could be T_1_ antiaromatic, our computations indicate that the two five-membered rings become antiaromatic in the T_1_ state (Supplementary Figs 135–136). Hence, at least two possibilities for triplet-state antiaromaticity localization exist in graphene, providing for increased photoreactivity than the larger PAHs. Yet, to what extent these play a role for graphene photochemistry need separate theoretical and computational investigations.

### Metal-free photoreactions with graphene

Even though our tests could be outside the realm of excited-state antiaromaticity, we now also examined metal-free transfer-photohydrogenations and photohydrosilylations of graphene. Graphane, that is, fully hydrogenated graphene, has been postulated as useful for numerous applications^44^,^45^,^46^,^47^. The main methodologies to hydrogenated graphene are hitherto either Birch reductions of graphite^48^ and other metal-mediated liquid phase reductions^49^, or gas phase hydrogenations of either graphite oxide^50^ or graphene on transition metal substrates such as Pt (ref. 51), Ir (ref. 47) and Ni (ref. 52). These methodologies require high pressures of hydrogen and/or metal catalysts. To the best of our knowledge, metal-free hydrogenation of graphene monolayers at ambient conditions has not been reported.

**Figure 5:**
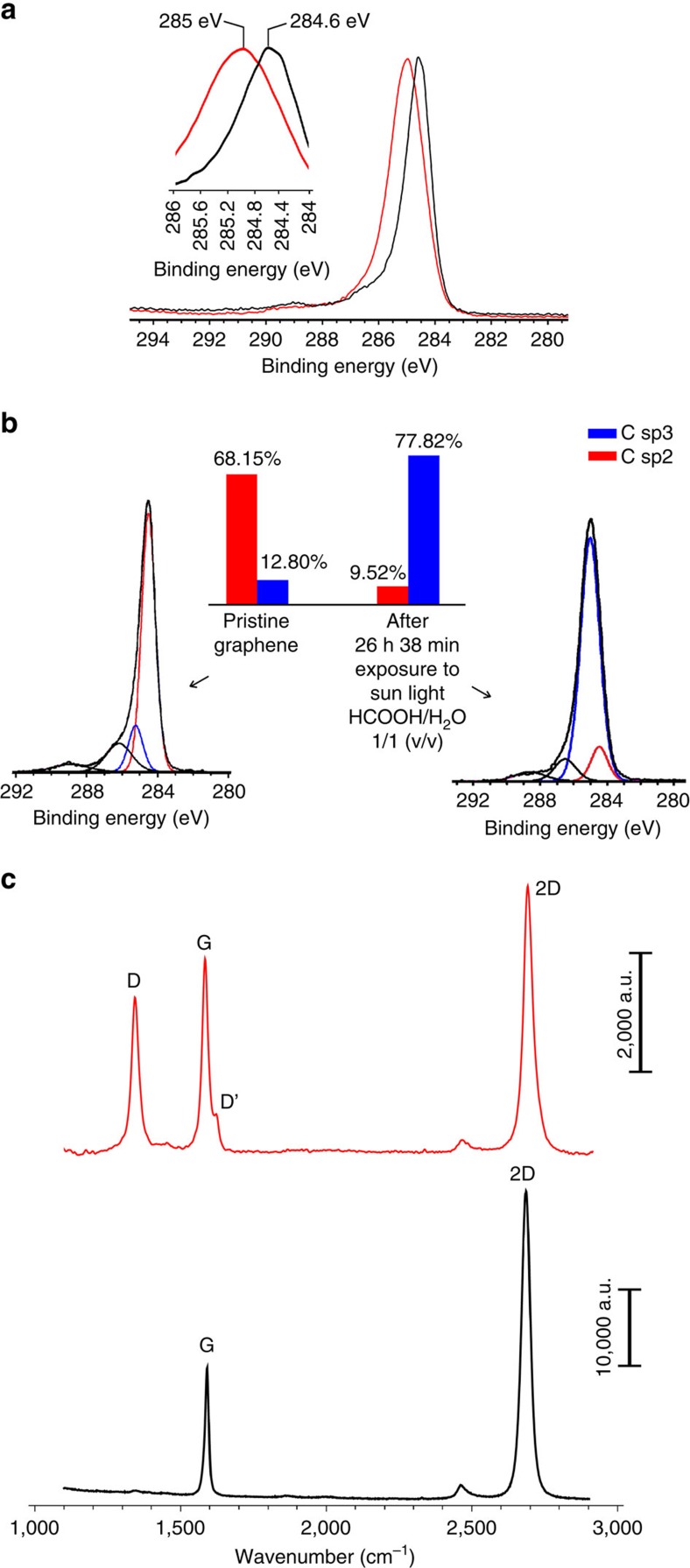
Metal-free photochemical transfer hydrogenation of graphene. (**a**) C1s XPS for graphene (black line) and graphane-like material formed on solar irradiation for 26 h and 38 min in aqueous HCOOH (red line). Inset: magnification of the C1s peaks depicting the observed shift on photohydrogenation. (**b**) The C1s XPS of **a** after deconvolution. Inset showing the ratio of the areas between *sp*^2^ (red) and *sp*^3^ (blue) hybridized carbon deconvoluted peaks. (**c**) Raman scattering of pristine graphene (black line) and graphene after photohydrogenation with solar irradiation in aqueous HCOOH for 26 h and 38 min (red line).

Photohydrogenation of chemical vapour deposition (CVD)-graphene on SiO_2_ was attempted with aqueous HCOOH as hydrogen transfer agent and white-light-emitting diode (LED) irradiation. This resulted in extensive hydrogenation as confirmed by X-ray photoelectron spectroscopy (XPS). Here it is noteworthy that the water content of the HCOOH/H_2_O mixtures influences the degree of hydrogenation, and the optimal HCOOH/H_2_O ratio was found to be 1/1 (v/v; Supplementary Fig. 82). Also the irradiation time influenced the extent of hydrogenation; 24 h with white light resulted in a twofold increase of the C1s *sp*^3^ signal area (and a roughly twofold decrease of C1s *sp*^2^ signal area) as compared with 6 h irradiation. Interestingly, irradiation of graphene with solar light for nearly 27 h on Crete, Greece, over two consecutive cloud-free days in August 2014 at 26–27 °C resulted in highly hydrogenated graphene according to XPS (Fig. 5a,b). For detailed description of the experiment see Supplementary Fig. 85 and Supplementary Methods. Noteworthy, the degree of hydrogenation after this experiment was higher than after white-LED irradiation using the same 1/1 mixture (see the Supplementary Information). Raman analysis of the treated samples also showed an intense D band, in addition to a significantly less intense 2D band when compared with pristine samples (Fig. 5c). These observations fully agree with those previously reported for hydrogenated graphene^46^.

A few photoinduced reactions with graphene have been reported. Of these, the photochemical phenylation by Liu *et al*.^53^ occurs by hot-electron transfer from photoexcited graphene to the lowest unoccupied molecular orbital (LUMO) of benzoyl peroxide, initiating photodissociation, decarboxylation and formation of phenyl radicals that attach to graphene. Noteworthy, tert-butyl peracetate, having a higher-energy LUMO, could not participate in hot-electron transfer and, accordingly, was unreactive. Computations now reveal that neither a hot-electron nor a hot-hole transfer is possible from white-light photoexcited graphene to Et_3_SiH or HCOOH as their LUMOs and highest occupied molecular orbitals (HOMOs) are placed at too high and too low energies, respectively (Supplementary Fig. 131). It should be mentioned that other transfer hydrogenation reagents tried were less efficient than HCOOH/water mixtures.

**Figure 6:**
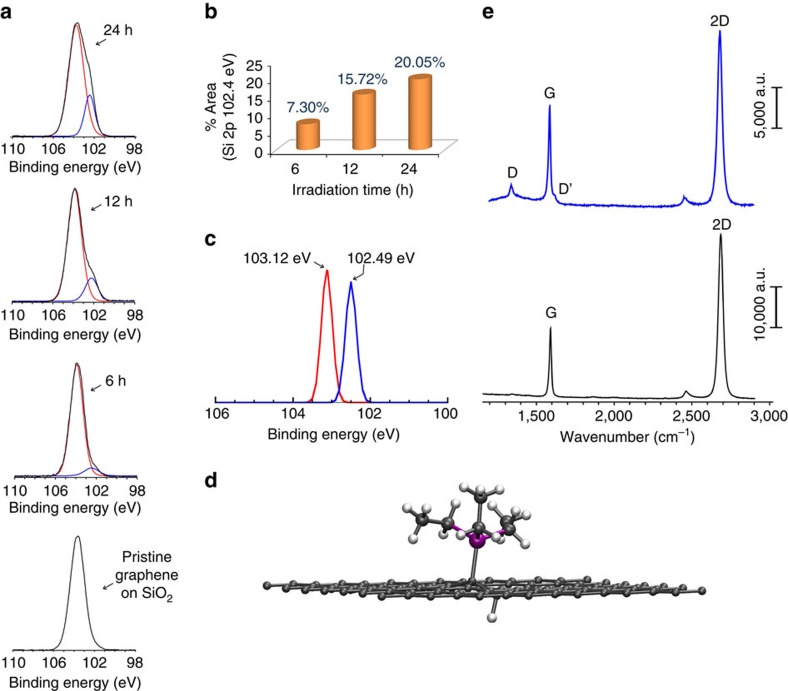
Metal-free photo(hydro)silylation of graphene. (**a**) The deconvoluted Si2p XPS of pristine graphene sample on SiO_2_ and of graphene exposed to white light in presence of Et_3_SiH for 6, 12 and 24 h. Red curve: Si2p band of SiO_2_ (centred at 103.7 eV), blue curve Si–C band (centred at 102.4 eV) appearing after photohydrosilylation of graphene. (**b**) Histogram showing the intensity of the latter band at different illumination times. (**c**) Calculated Si2p XPS bands for SiO_2_ (red) and partially hydrosilylated graphene (blue). (**d**) Optimized structure of Si–H dissociated triethylsilane bonded to graphene. Colour code: C, grey; H, white; Si, purple. (**e**) Raman scattering spectra of pristine graphene (black) and graphene after photoreaction with Et_3_SiH for 24 h (blue).

Treatment of CVD-graphene on SiO_2_ with Et_3_SiH using white-light irradiation results in photo(hydro)silylation, as evidenced by the appearance of a new Si signal at 102.4 eV in the Si2p XPS spectrum of the treated graphene (Fig. 6a). The degree of graphene photo(hydro)silylation depends on illumination time, reaching a plateau after ∼24 h (Fig. 6b). Evidence that the new peak results from a triethylsilyl group covalently bonded to graphene is obtained from density functional theory (DFT) calculations using the projector-augmented wave^54^-based GPAW^55^ code (Fig. 6c). The geometry optimized partially *trans*- and *cis*-hydrosilylated graphene sheets (Fig. 6d for *trans*-adduct) shows Si2p XPS peaks at 102.49 and 102.70 eV, respectively, in line with our experimental value of 102.4 eV (Fig. 6a,c). Noteworthy, *cis*-addition yields a hydrosilylated graphene, which is 9.5 kcal mol^−1^ (0.41 eV) less stable per formula unit than the *trans*-adduct. The computed bulk Si2p XPS peak of SiO_2_ is at 103.12 eV, which should be close to the substrate peak for pristine graphene on SiO_2_, that is, in the absence of Et_3_SiH. This value is rather consistent with the experimental value of 103.7 eV obtained for the SiO_2_ substrate. The shift to lower binding energies for the Si2p XPS signal of the triethylsilyl group when compared with that of SiO_2_ can be rationalized by the lower electronegativity of carbon than of oxygen. Thus, our results clearly reveal photofunctionalization of graphene by silyl group attachments, although it is not resolved whether the product is a hydrosilylated or silylated graphene.

## Methods

### Irradiation experiments of benzene

A solution of triethylsilane in dry benzene (16 ml, 12 mM) was purged with Ar and then added in degassed quartz tubes sealed with rubber septa, using a syringe. The tubes were placed in a Rayonet photoreactor and irradiated at *λ*=254 nm for 24 h. After the reaction, formation of a yellowish polymer on the surface of the tubes was observed. The solutions were combined and solvent (benzene) was evaporated *in vacuo*. The residual was purified with column chromatography to yield phenyltriethylsilane in 6% yield. Isolation-purification details, nuclear magnetic resonance spectra and GC–MS are found in Supplementary Figs 42–44).

### Photo(hydro)silylation of naphthalene

In a 70 ml cylindrical quartz tube, a solution of naphthalene in dry *n*-heptane was added (187 mg, 1.46 mmol in 50 ml *n*-heptane). The tube was sealed, the solution was degassed with Ar, and 242 μl (15.15 mmol) of triethylsilane was added under Ar. The tube was placed in a Rayonet photoreactor and irradiated for 72 h at 254 nm. Subsequently, the solvent was evaporated on a rotary evaporator and the remaining oil was purified by column chromatography (details in Supplementary Figs 58–64 and Supplementary Methods).

### Photo(hydro)silylation of larger PAHs

A method similar to naphthalene was applied for anthracene, phenanthrene, pyrene, fluoranthene and coronene. Benzene was used as a solvent instead of *n*-heptane for the insoluble PAHs. The concentrations of the PAHs in all cases were 5 mM and the irradiation wavelength was *λ*=300 nm for 24–48 h (details in Supplementary Methods and Supplementary Figs 66–74).

### Transfer photohydrogenation of PAHs

Method for photohydrogenation of PAHs (the ones absorbing *λ*≥300 nm); the aromatic compound of interest was dissolved in 100 ml of benzene/AcOEt (1/1: v/v) in concentration of 5 mM, and then degassed using Ar. In the degassed solution, 10 ml HCOOH were added (HCOOH concentration: 2.5 M; 500 × equimolar). The resulting solution was irradiated for 48 h at 300 nm. Then the solvent was evaporated and the residue was purified by column chromatography.

### Transfer photohydrogenation of graphene

A typical experiment was performed as follows: a CVD-graphene sample (details in Supplementary Methods and Supplementary Figs 81–85) was immersed in aqueous HCOOH (HCOOH/H_2_O mixtures were used in volume ratios: 1/4, 1/1, 4/1) in a cylindrical tube (borosilicate glass; 5 mm wide), capped with a rubber septum and then the liquid mixture was degassed with Ar. Subsequently, the tube was placed in the centre of a home-made white-LED photoreactor and irradiated (images and descriptions in the Supplementary Methods section: Photochemical reactors and glassware). The illumination time ranged between 6 and 48 h. After this, the liquid mixture was removed from the tube and the sample was collected, washed carefully with deionized water and dried with a stream of Ar gas. For the analysis, see Supplementary Methods.

### Photo(hydro)silylation of graphene

A CVD-graphene sample was immersed in neat triethylsilane (Et_3_SiH) in a tube (borosilicate glass; 5 mm wide) degassed with Ar, and illuminated using a home-made white-LEDs photoreactor (see Supplementary Methods section: Photochemical reactors and glassware) for a time period varying between 6 and 24 h. The sample after irradiation was washed using large amounts of dichloromethane to remove residual unreacted Et_3_SiH from the graphene surface and then dried using a stream of Ar gas. For the analysis see Supplementary Figs 87–89 and Supplementary Methods.

### Quantum chemical calculations on benzene and PAHs

DFT calculations were performed with Gaussian09 (ref. 56) at the B3LYP and the dispersion-corrected B3LYP-D3(BJ) levels^57^,^58^ using the 6-311+G(d,p) basis set^59^. Free energies were calculated at 298 K and 1 atm under the ideal gas approximation. ACID plots were generated with the AICD 2.0 programme^60^ using the CSGT method. The CASSCF and CASPT2 calculations^61^ were done with the ANO-RCC basis sets^62^ and MOLCAS 8.1 (ref. 63) and NICS-XY scans^64^ were generated with the Aroma package^65^. The single-reference characters of the geometry-relaxed T_1_ states of benzene and the PAHs (excluding coronene) were confirmed through RASSCF/CASSCF computations.

### Modelling of XPS of hydrosilylated graphene

For the modelling of XPS, we chose a 7 × 7 × 1 graphene supercell with Et_3_SiH attached onto it, dissociated into a triethylsilyl group and an H atom bonded to opposite sides of the graphene sheet. We have used plane-wave based DFT code VASP^66^ to optimize the structure. The PBE^67^ exchange-correlation potential has been used within generalized gradient approximation. The structures were optimized using the conjugate gradient method with forces calculated from the Hellman–Feynman theorem. The energy and the Hellman–Feynman force thresholds are kept at 10^−5^ eV and 10^−2^ eV Å^−1^, respectively. For geometry optimization, a 5 × 5 × 1 Monkhorst–Pack k grid is used. This optimized structure was used to calculate XPS with the DFT using the PAW^54^-based GPAW^55^ code. To compute the binding energy measured by XPS, we computed the total energy difference between the ground state and the Si2p core-ionized state.

### Data availability

Detailed experimental procedures, characterization of compounds and computational details are found in the Supplementary Figs 1–136, Supplementary Tables 1–12 and Supplementary Methods. All other data are available from the authors on reasonable request.

## Additional information

**How to cite this article**: Papadakis, R. *et al*. Metal-free photochemical silylations and transfer hydrogenations of benzenoid hydrocarbons and graphene. *Nat. Commun.* **7**, 12962 doi: 10.1038/ncomms12962 (2016).

## Supplementary Material

Supplementary Information Supplementary Figures 1-136, Supplementary Tables 1-12, Supplementary Methods and Supplementary References

Peer review file
