# Supplementary material for: Metal-free photochemical silylations and transfer hydrogenations of benzenoid hydrocarbons and graphene
Source: Nat Commun. 2016 Oct 6;7:12962. doi: 10.1038/ncomms12962 (PMC5059713; doi:10.1038/ncomms12962)
Supplement: Supplementary Information — Supplementary Figures 1-136, Supplementary Tables 1-12, Supplementary Methods and Supplementary References [file ncomms12962-s1.pdf]

Supplementary Figures

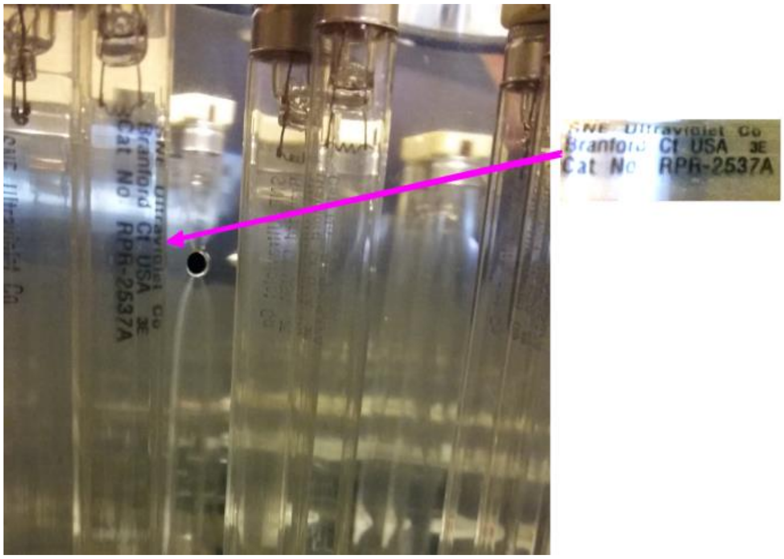

Supplementary Figure 1. Photo showing the model of the lamps that we used for the 254 nm reactions (RPR-2537A).

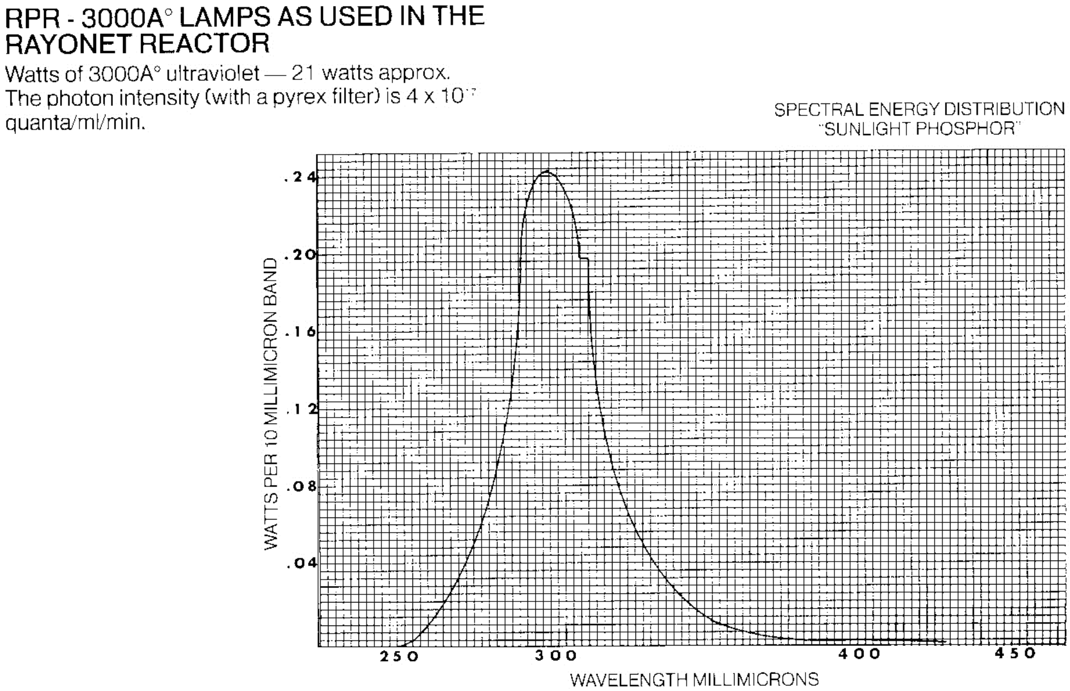

Supplementary Figure 2. Intensity characteristics for the RPR-3000 A lamps.

A

WATTS OF 2537 Å ULTRAVIOLET - - 35 WATTS APPROX.

PHOTONS OF 2537 Å ULTRAVIOLET - - 1.65×10^{16} /sec/cm^3

INTENSITY READINGS IN MICROWATTS /cm^2 AT:

Wave Length (Å)	Center	2 Inches From Lamps	1.5 Inches From Lamps
2537	12800	16000	21000
2652	388	488	640
2804	14	16	21
2894	18	22	29
2967	66	83	109
3022	32	40	52
3129	250	313	411
3654	213	267	351
4047	250	316	416
4359	768	960	1260
5461	418	523	687
5780	91	113	149

ABOVE DATA FROM NEW LAMPS WITH A REACTOR TEMPERATURE OF 44°C

B

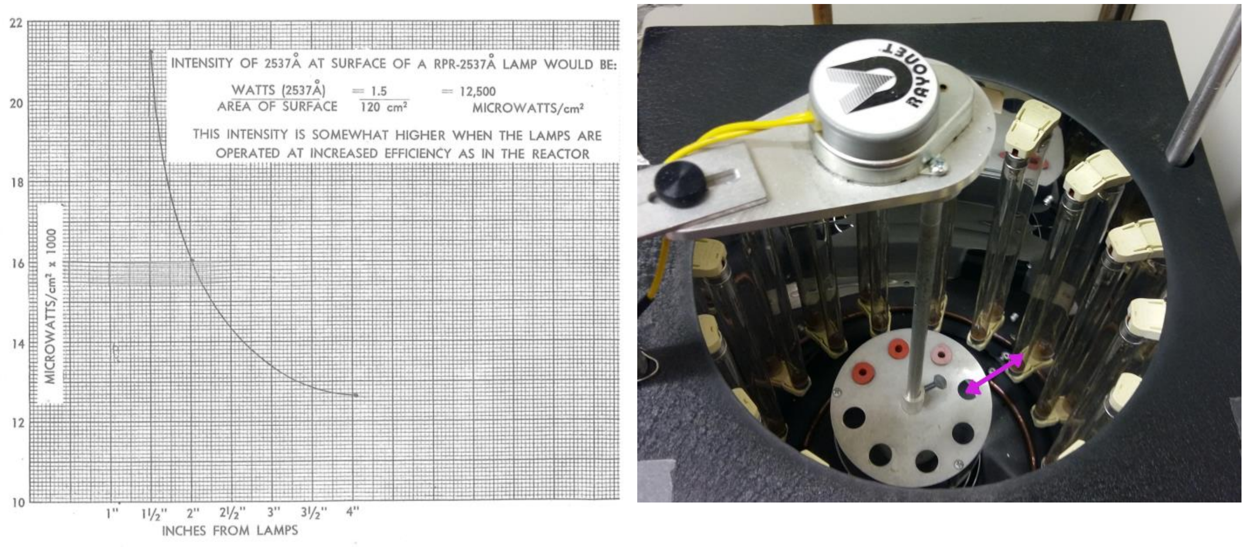

Supplementary Figure 3. (A) Intensity characteristics for the RPR-2537A lamps. (B) A photo of the RPR-100 Rayonet Photochemical Chamber Reactor indicating the distance between the sample holder and the lamps (see magenta colored arrow: 8.5 cm ≈ 3.3")

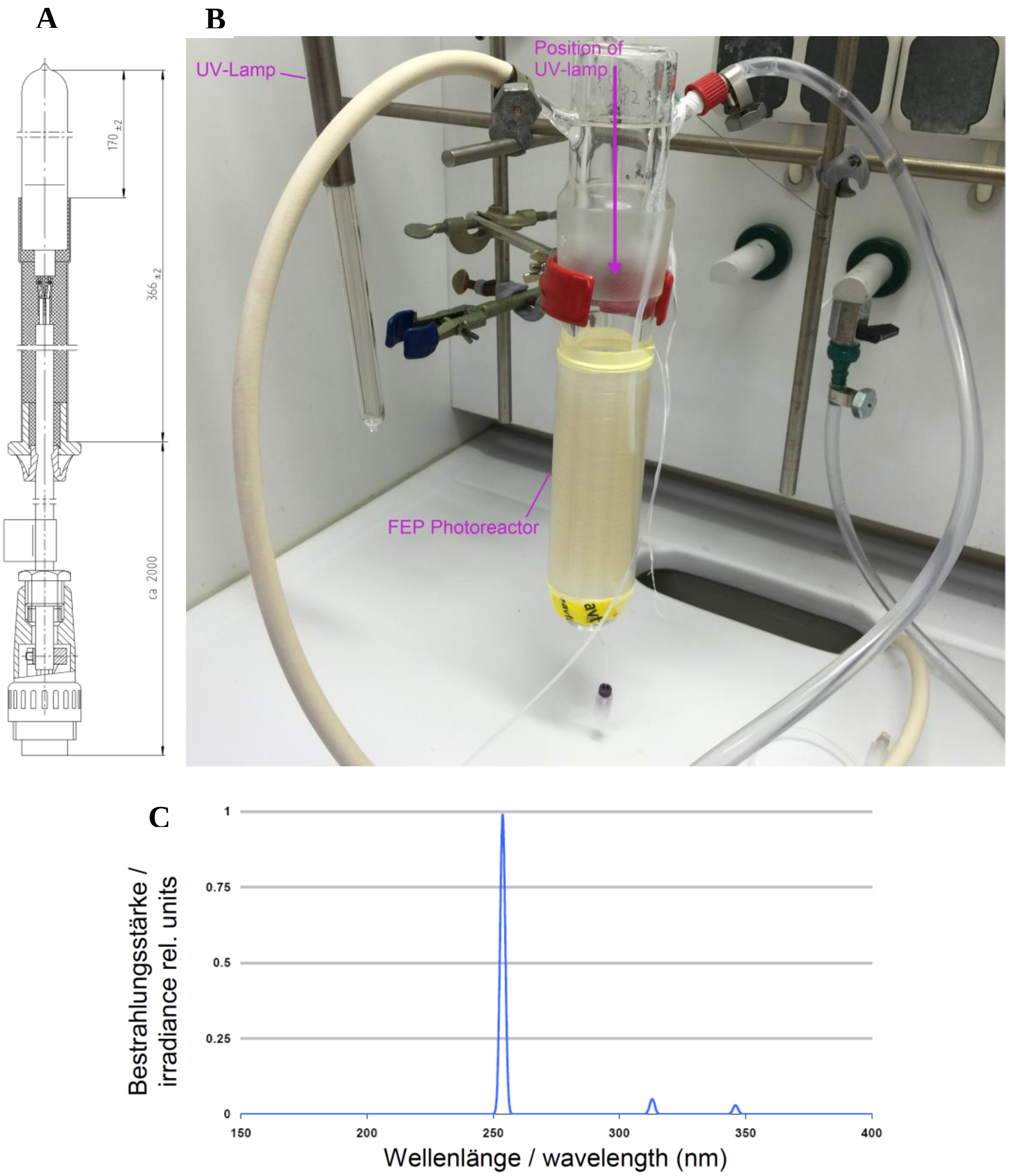

Supplementary Figure 4. (A) Illustration of the Peschl 254 nm Hg lamp (B) Photograph depicting the home-made FEP photoreactor and the Peschl 254 nm Hg lamp (on the left). The purple colored arrow indicates the position of the lamp inside the FEP-photoreactor. (C) Emission spectrum of the Peschl UV-lamp.

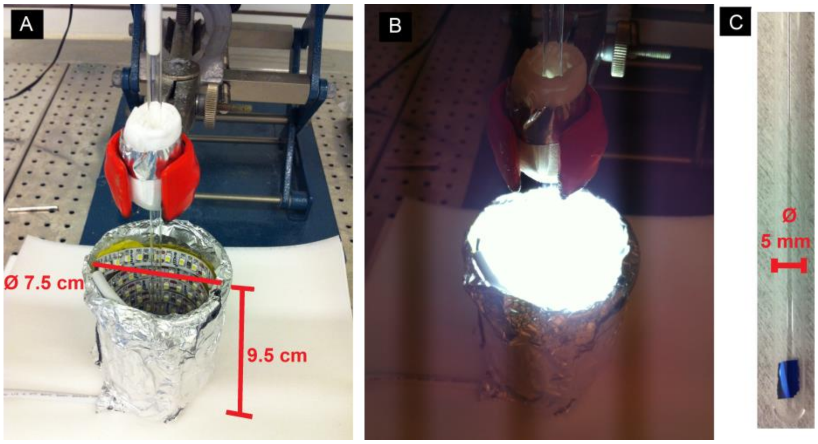

Supplementary Figure 5. (A) Photograph of the photoreactor used for graphene photohydrogenation and photo(hydro)silylation. Ø 7.5 cm, height 9.5 cm. Number of LEDs: 120. (B) Photoreactor shining light. C) A CVD graphene sample inside a 5 mm Ø NMR tube (borosilicate) used for illumination.

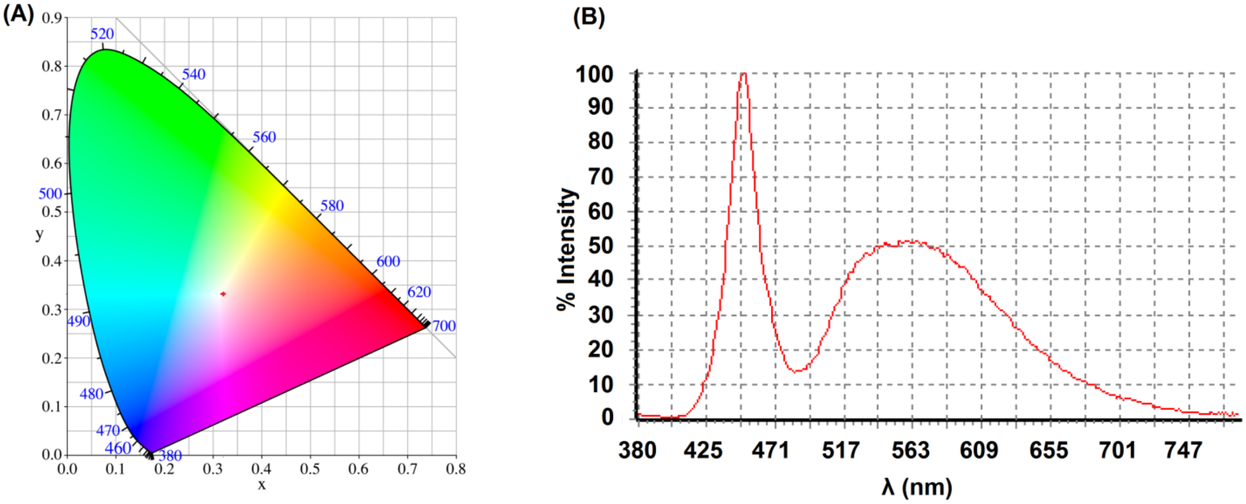

Supplementary Figure 6. (A) CIE Chromaticity Diagram for the WLED used [red mark corresponds to (x,y,) = (0.3196, 0.3317)]. (B) Emission spectrum of the white LEDs.

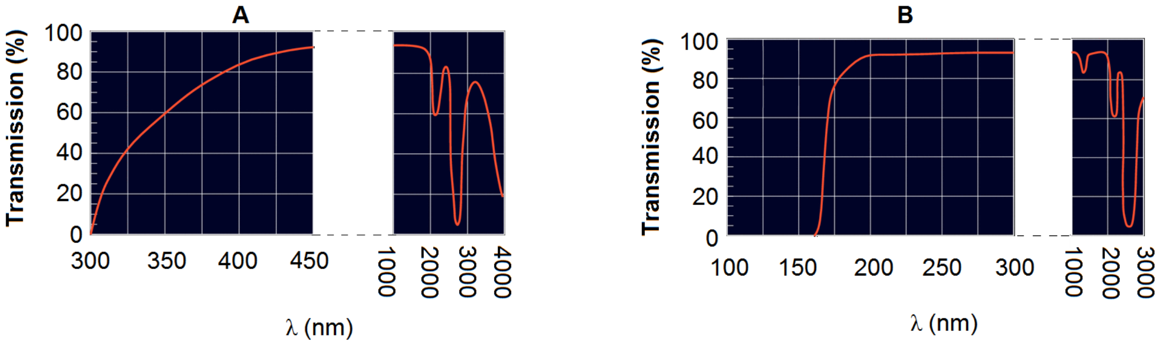

Supplementary Figure 7. Optical transmission spectra for borosilicate (optical) glass tubes (A) and quartz tubes (B) used in this work.

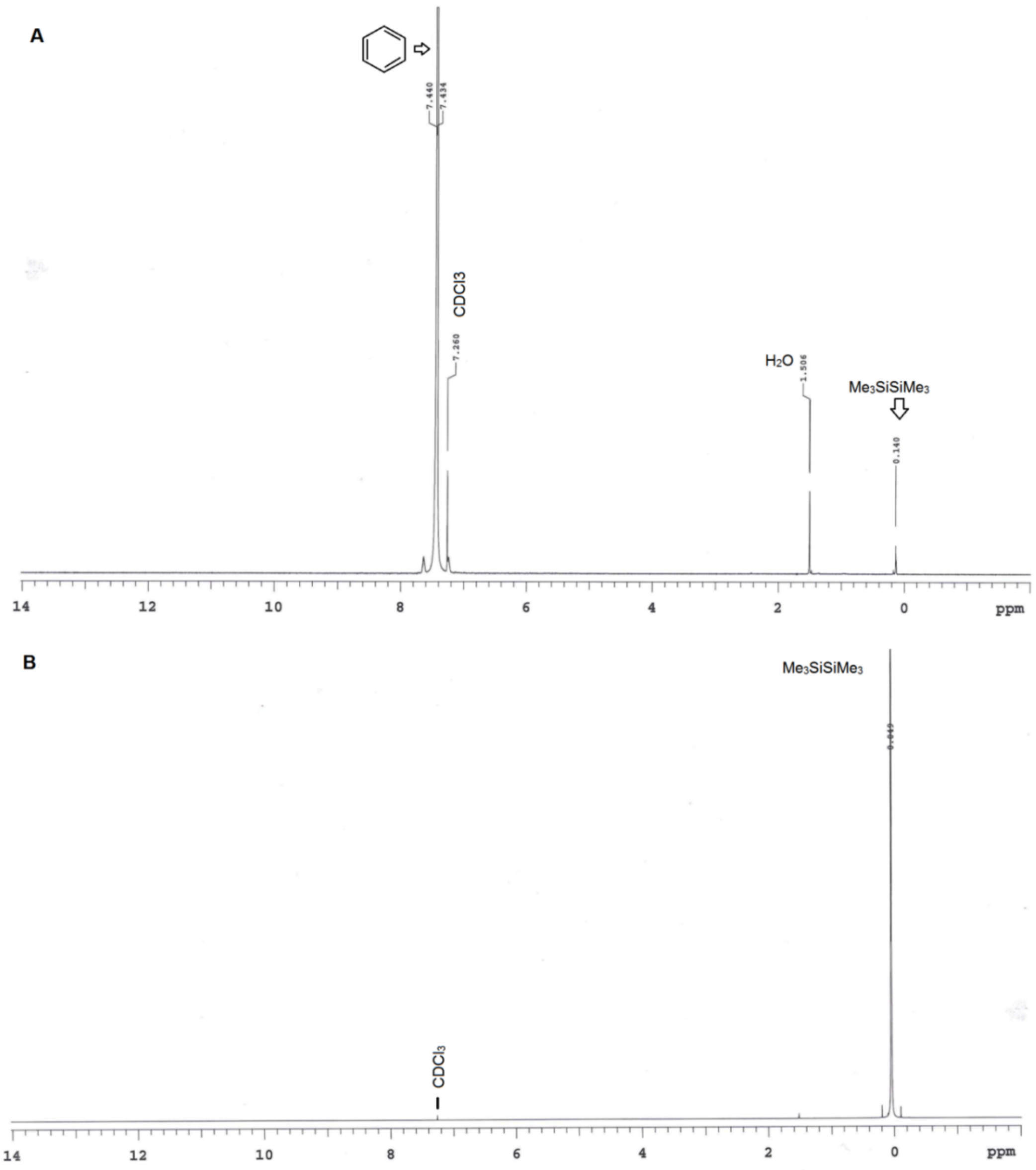

Supplementary Figure 8. (A) ^{1}H-NMR spectrum of the solution obtained after 24 h irradiation of Si_2Me_6 in benzene ($[Si_2Me_6]$= 12 mM) at 254 nm, and (B) ^{1}H-NMR spectrum of pure Si_2Me_6. Both spectra were recorded in $CDCl_3$.

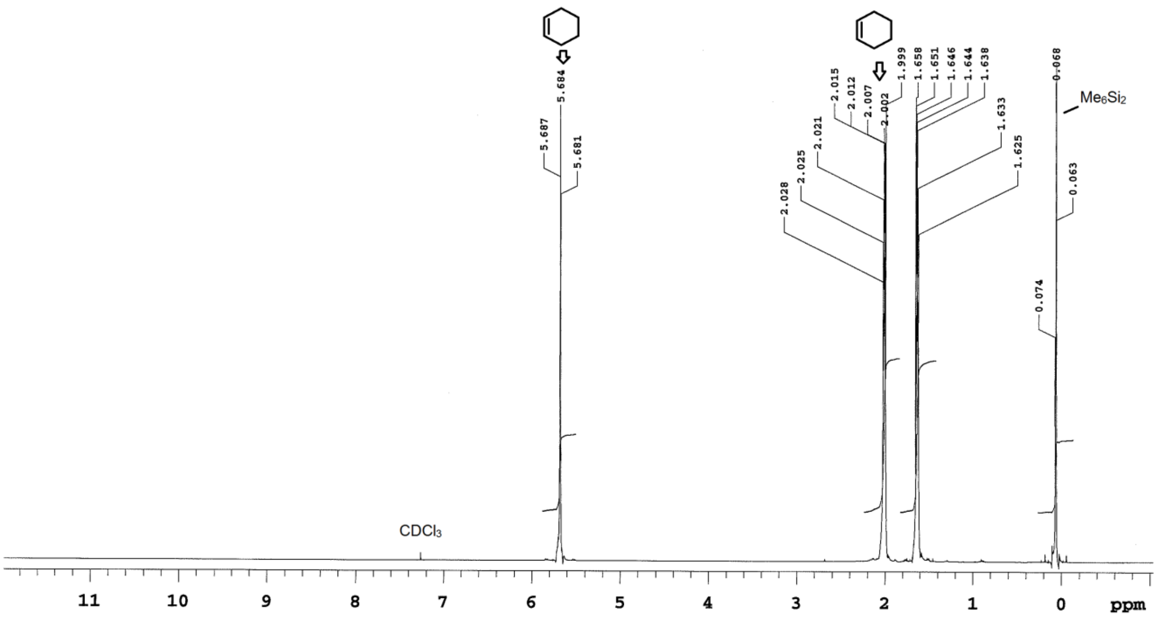

Supplementary Figure 9. ^{1}H-NMR spectrum of the solution obtained after 24 h irradiation of Si_2Me_6 in cyclohexene ([Si_2Me_6] = 0.542 M) at 254 nm. The ^{1}H-NMR spectrum was recorded in $CDCl_3$.

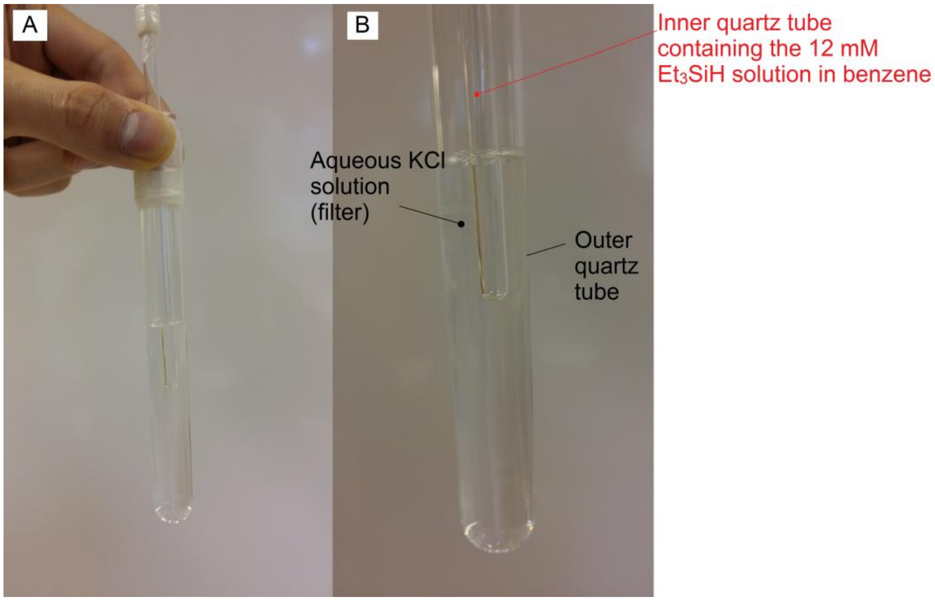

Supplementary Figure 10. (A) Photograph depicting a NMR quartz tube (inner tube) immersed in a bigger quartz tube (RQV-5 Rayonet; Ø 13 mm) containing a 10% (w/v) KCl aqueous solution acting as a filter of light with λ in the region 180 to 200 nm. (B) A magnification of the photo in A.

Supplementary Figure 11. (A) GC obtained after irradiation of a 12 mM benzene solution of Et_3SiH at 254 nm for 24 h using a KCl_{aq} filter. Main product was $PhEt_3Si$ with a GC-MS yield of ~6%. (B) MS of the obtained $PhEt_3Si$.

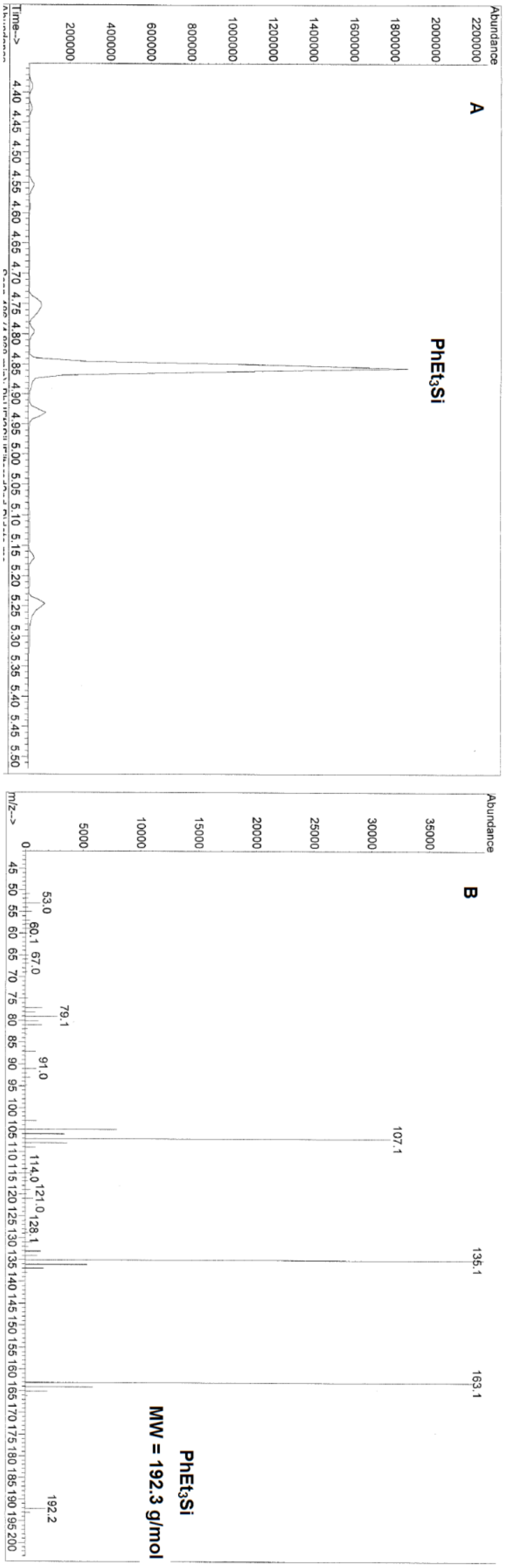

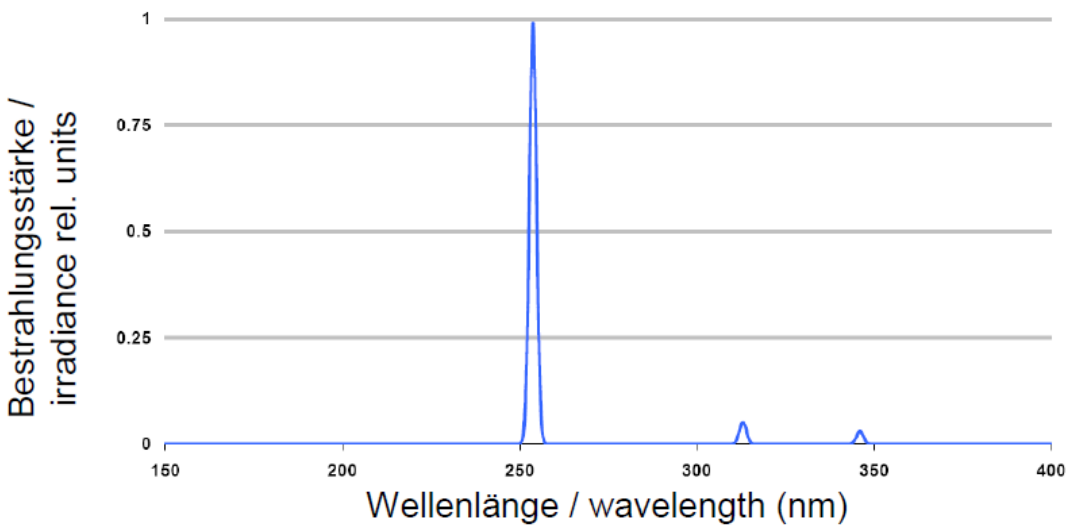

Supplementary Figure 12. Emission spectrum of the TNN 15/32 UV-lamp obtained from the Peschl Ultraviolet company.

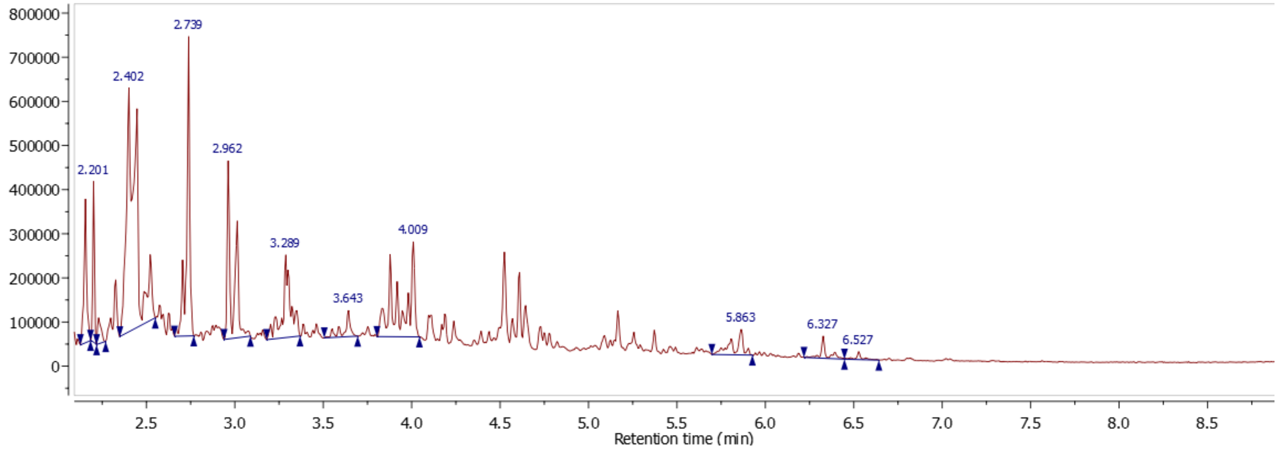

Supplementary Figure 13. GC of the solution obtained after 2 h irradiation of an equimolar solution of benzene and Et_3SiH in *n*-heptane ([Et_3SiH] = [benzene] =10 mM) under simultaneous ozone bubbling. (No silylated or hydrosilylated benzenes were formed).

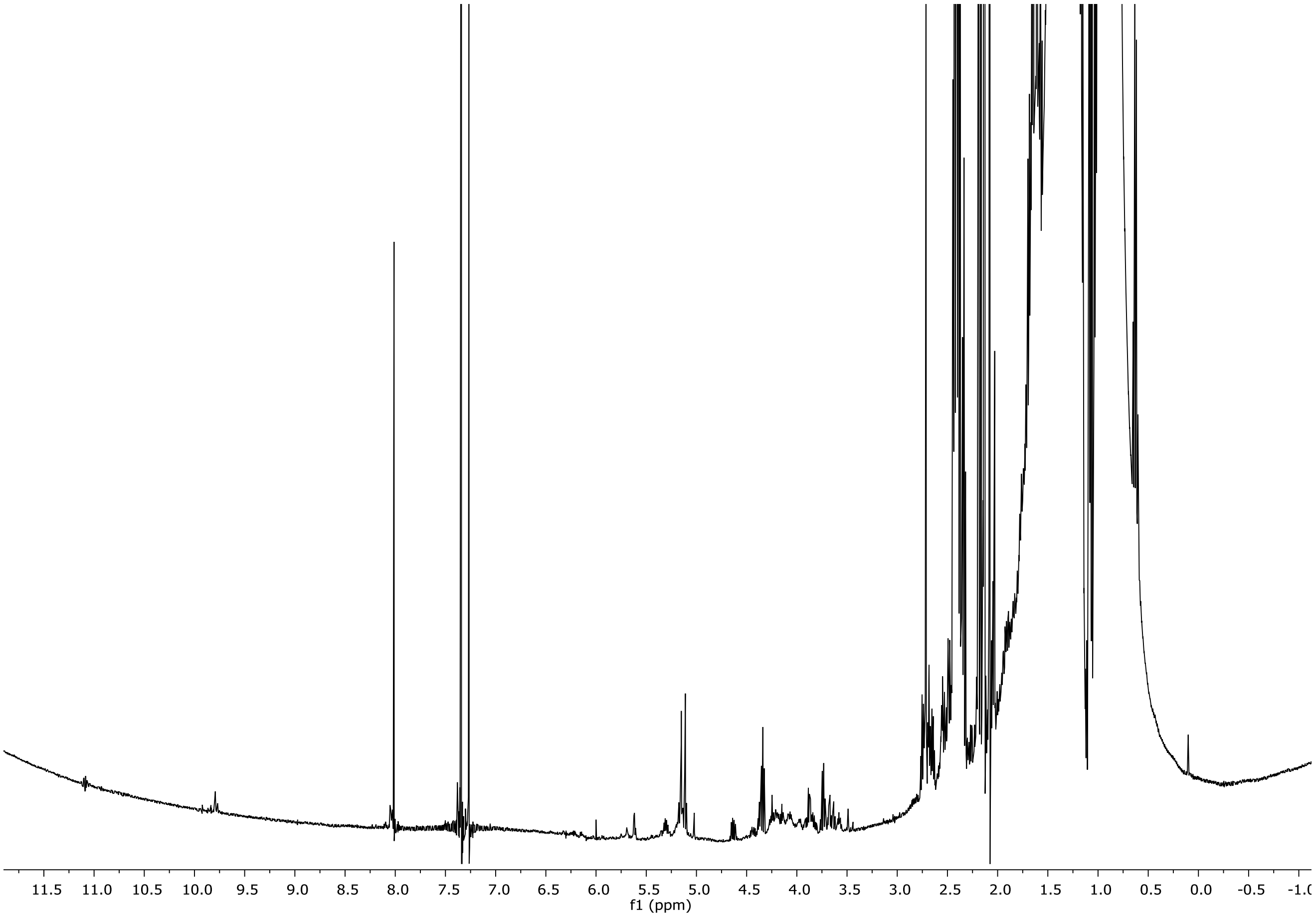

Supplementary Figure 14. ^{1}H-NMR spectrum of the solution obtained after 2 h irradiation of an equimolar solution of benzene and Et_3SiH in *n*-heptane ($[Et_3SiH]$ = [benzene] =10 mM) under simultaneous ozone bubbling. The spectrum was recorded in $CDCl_3$.

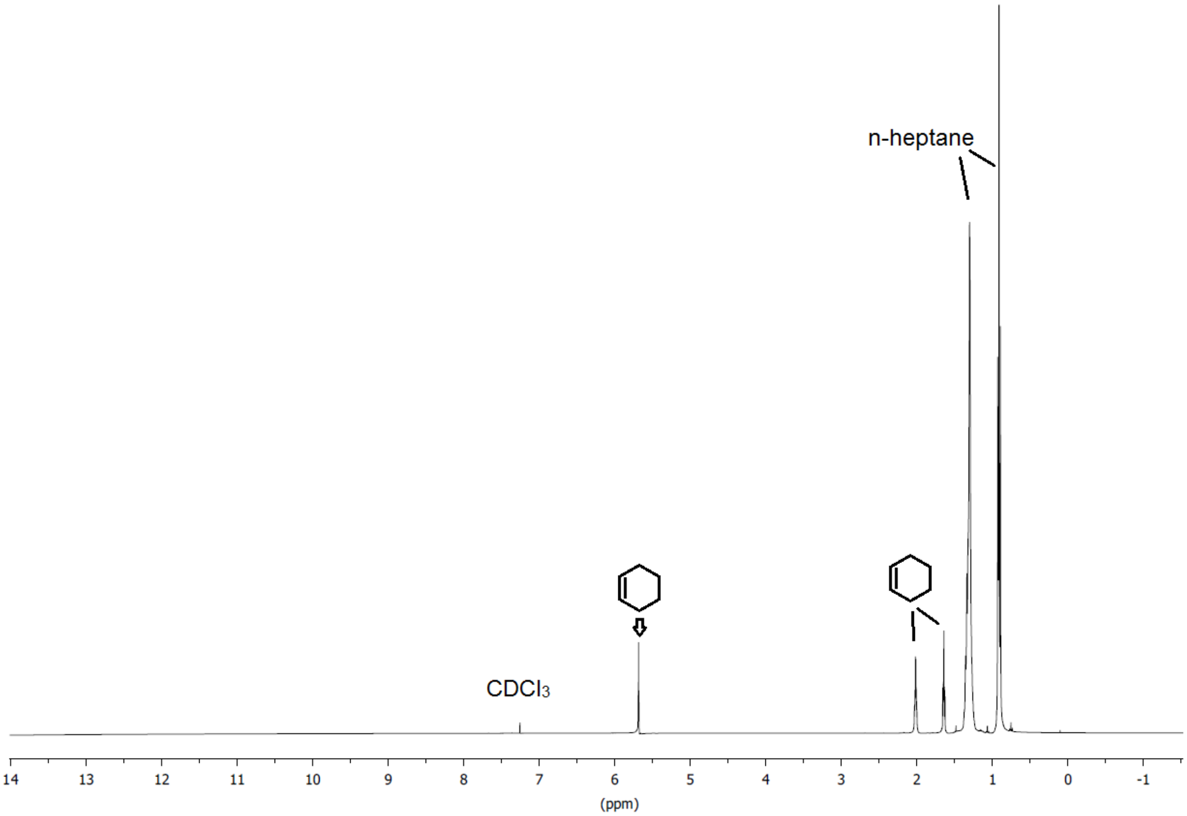

Supplementary Figure 15. ^{1}H-NMR spectrum of the solution obtained after 24 h irradiation of cyclohexene in *n*-heptane at 254 nm (open tube). The ^{1}H-NMR spectrum was recorded in $CDCl_3$.

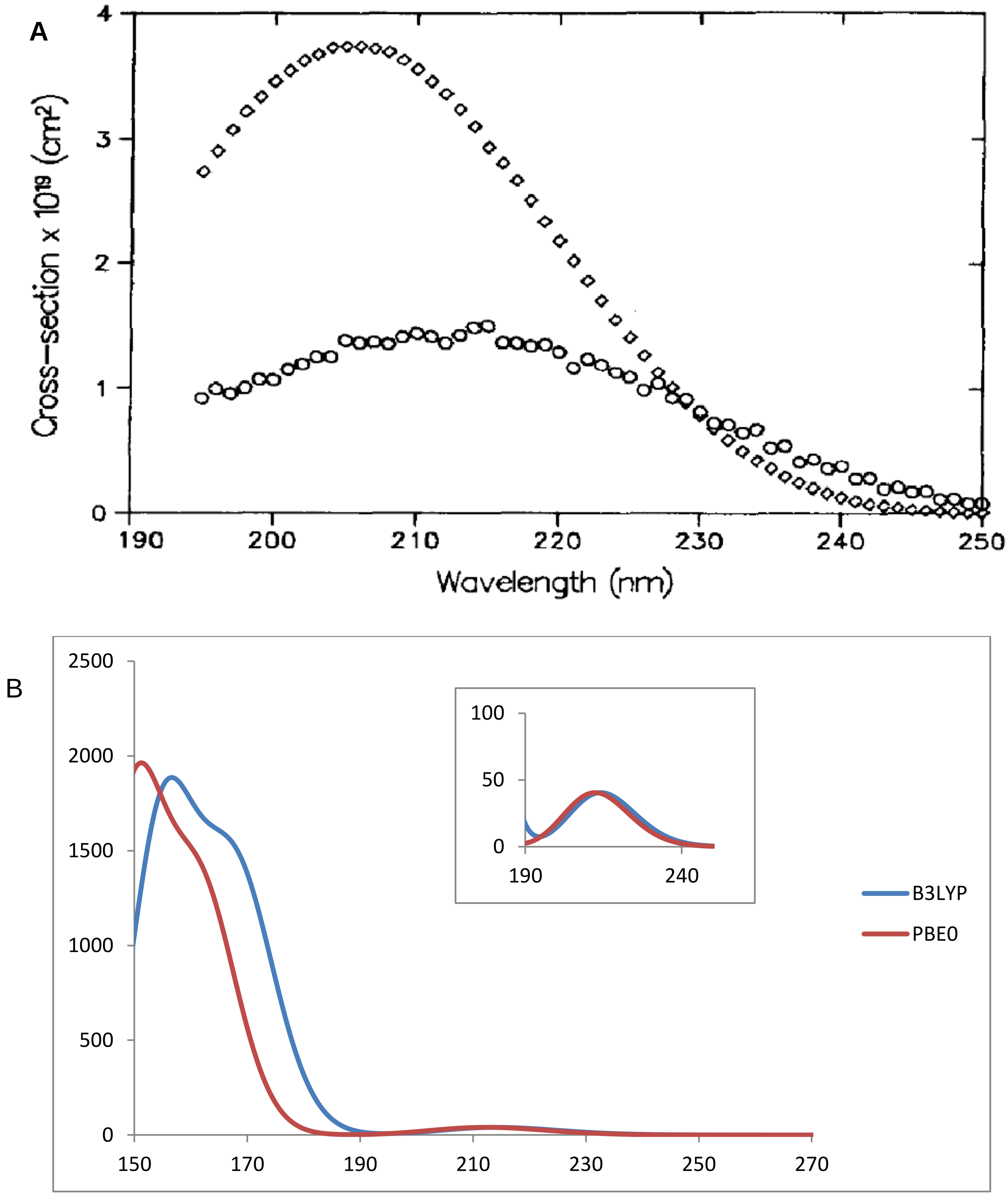

Supplementary Figure 16. Vacuum absorption cross-sections of the monomer (○) and dimer (◊) of formic acid as a function of wavelength at 302 K. Figure taken from. UV absorption cross-sections of the monomer and dimer of HCOOH according to Singleton *et al.*[1] (A) and TD-DFT spectrum of HCOOH with TD-B3LYP and TD-PBE0 and the 6-311+G(2d,p) basis set (B).

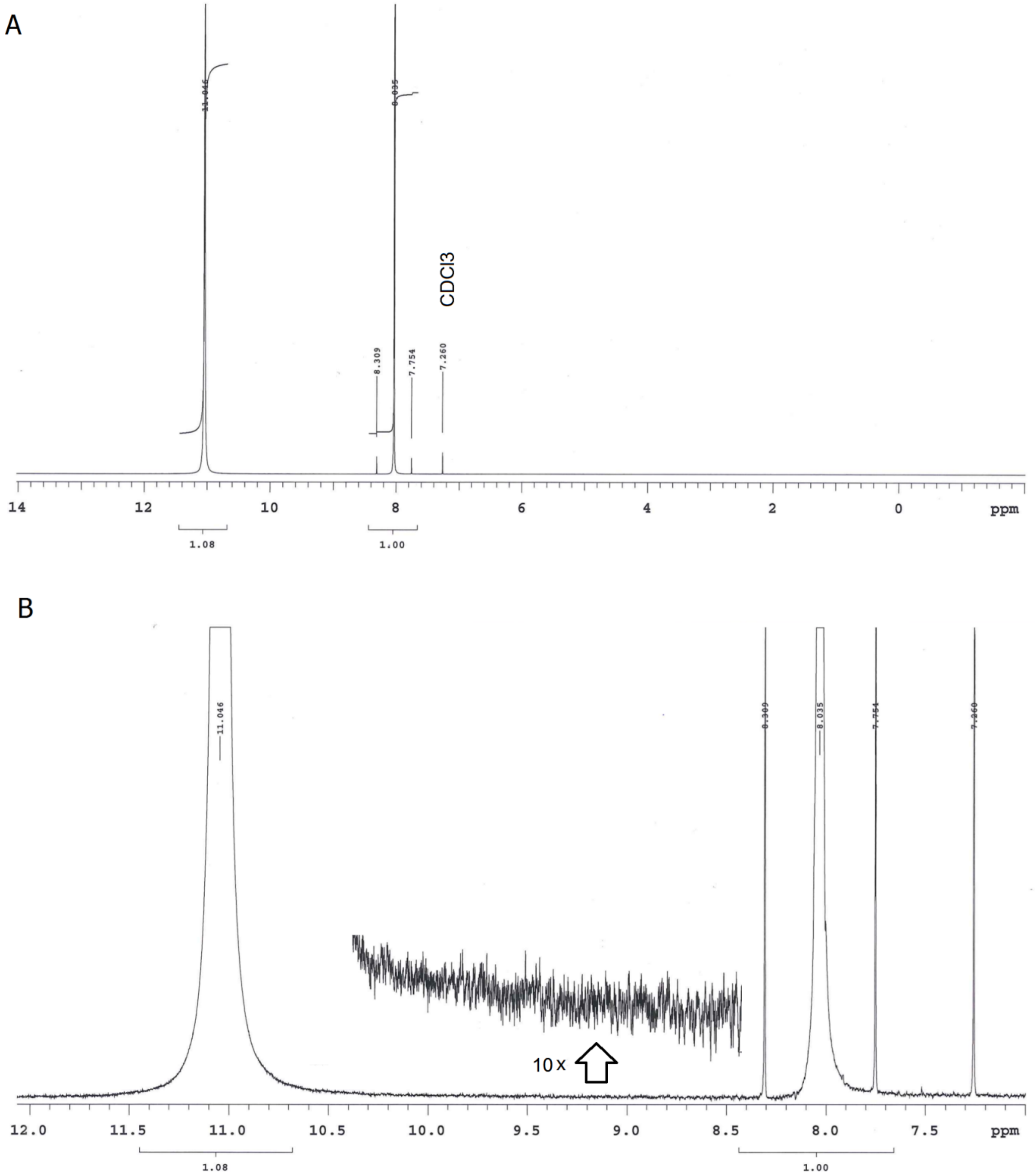

Supplementary Figure 17. (A) Full ^{1}H-NMR spectrum of formic acid in $CDCl_3$ after irradiation at 300 nm in a quartz tube. (B) Partial ^{1}H-NMR spectrum of formic acid in $CDCl_3$ after irradiation at 300 nm. Inset: magnification of the region between 8.5 and 10.5 ppm showing the absence of any peak corresponding to formaldehyde.

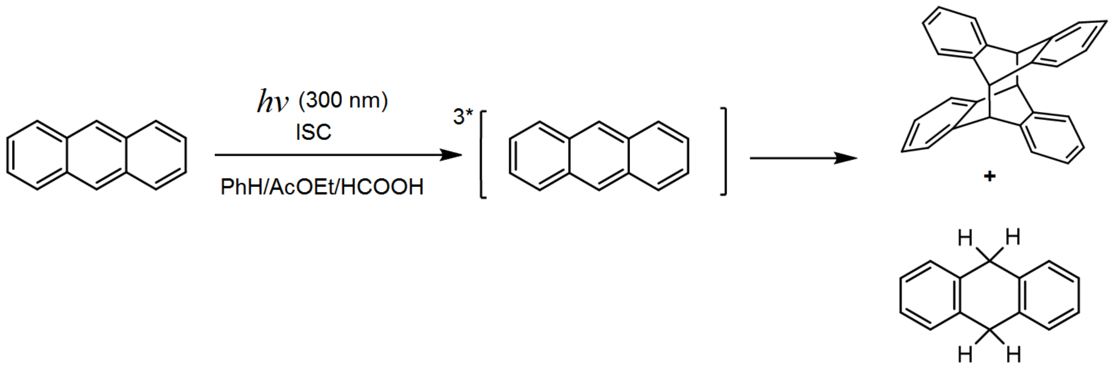

Supplementary Figure 18. Photoreaction of anthracene in a mixture of PhH/AcOEt/HCOOH (5/5/1: v/v/v) upon UV-irradiation (300 nm) to give anthracene dimer and 9,10-dihydroanthracene.

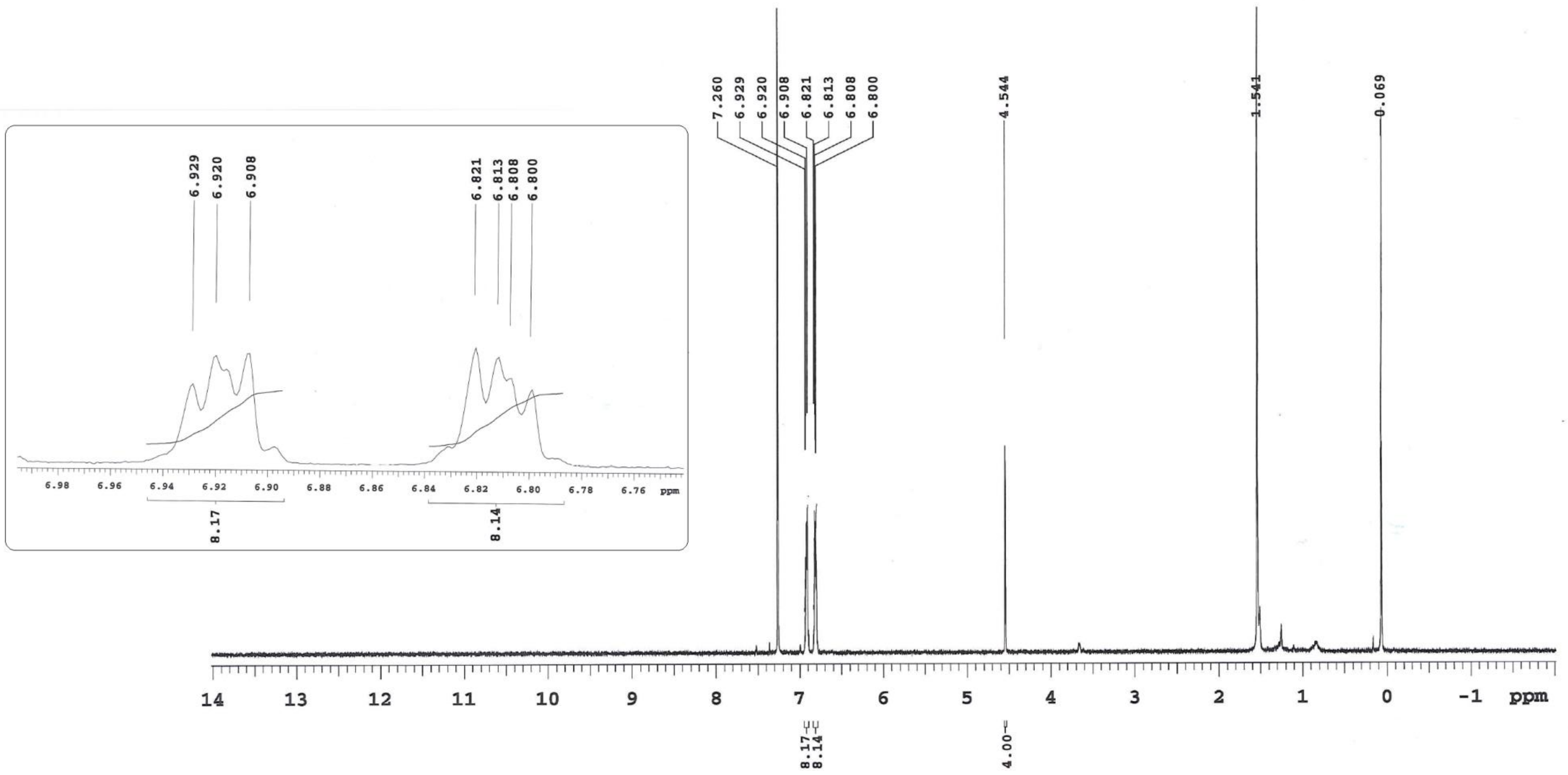

Supplementary Figure 19. ^{1}H-NMR spectrum of the isolated anthracene dimer after scaled-up photohydrogenation of anthracene in PhH/AcOEt/HCOOH (5:5:1 v:v:v), recorded in $CDCl_3$. (Relaxation Delay: 10.000 sec; Pulse: 45.0 degrees; 16 repetitions). Inset: Magnification of the aromatic region.

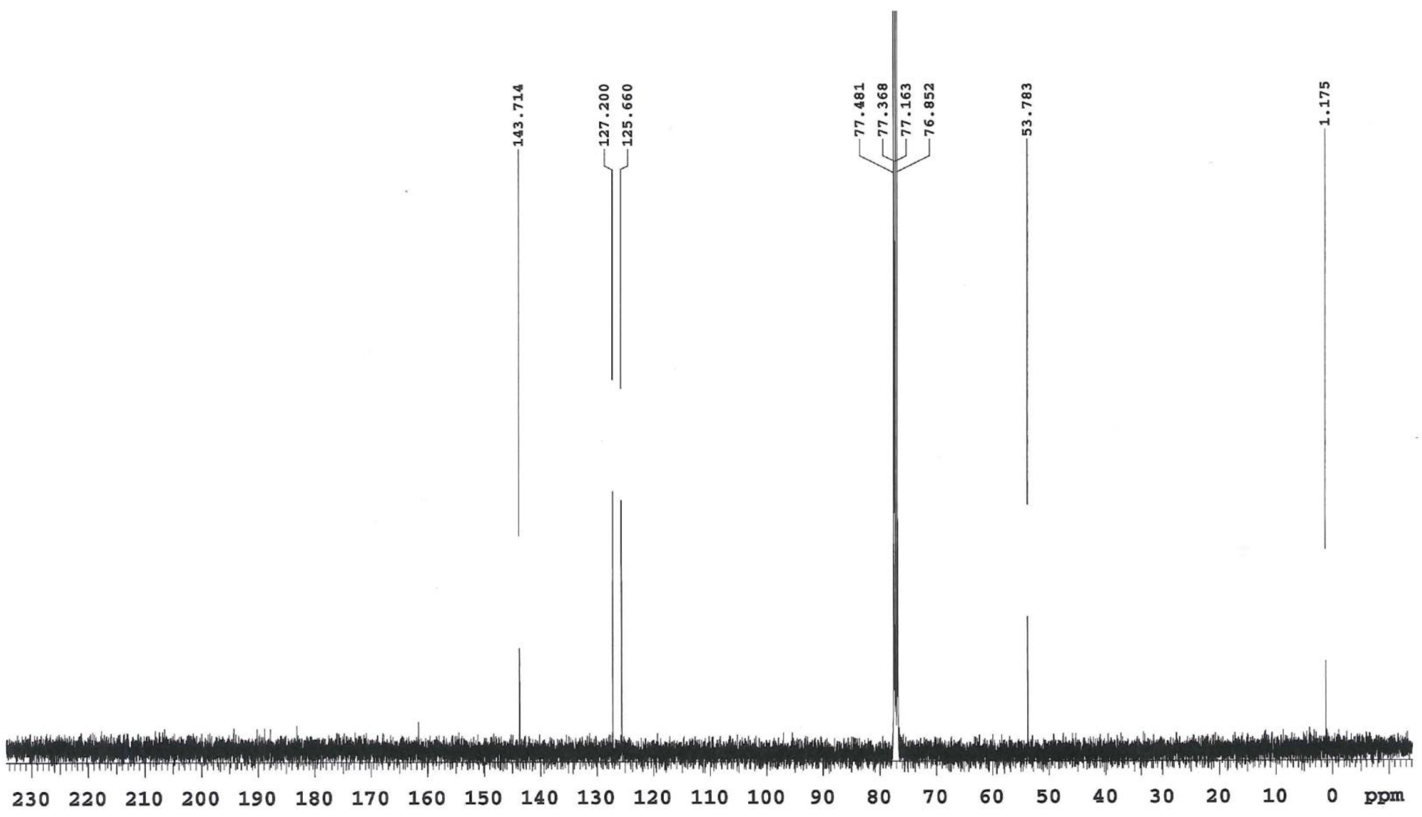

Supplementary Figure 20. ^{13}C-NMR spectrum of the isolated anthracene dimer, after scaled-up photohydrogenation of anthracene in PhH/AcOEt/HCOOH (5/5/1: v/v/v), recorded in $CDCl_3$. (Relaxation Delay: 1.000 sec; Pulse: 45.0 degrees; 5000 repetitions).

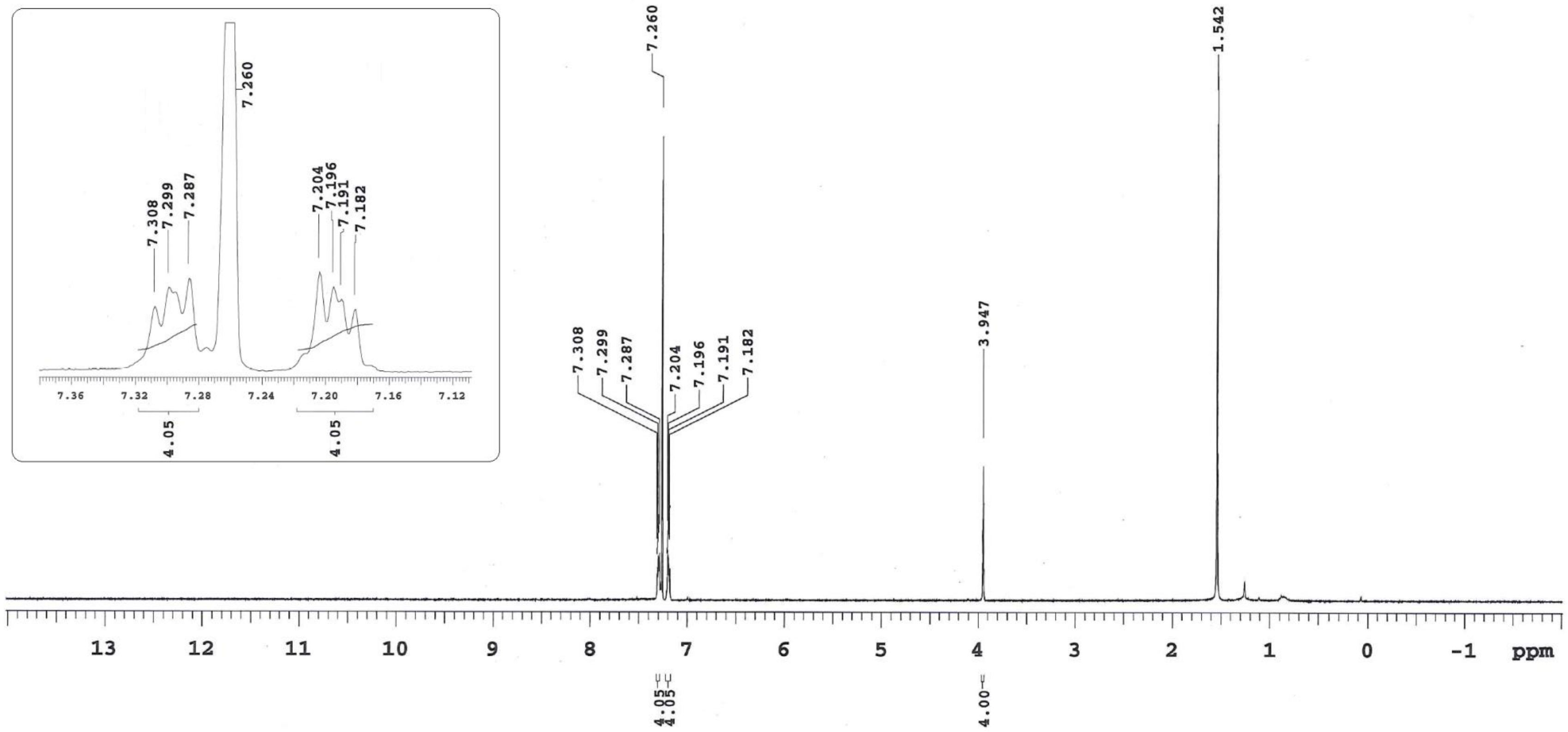

Supplementary Figure 21. ^{1}H-NMR spectrum of the isolated 9,10-dihydroanthracene after scaled-up photohydrogenation of anthracene in PhH/AcOEt/HCOOH (5:5:1 v:v:v), recorded in $CDCl_3$. (Relaxation Delay: 10.000 sec; Pulse: 45.0 degrees; 16 repetitions). Inset: Magnification of the aromatic region.

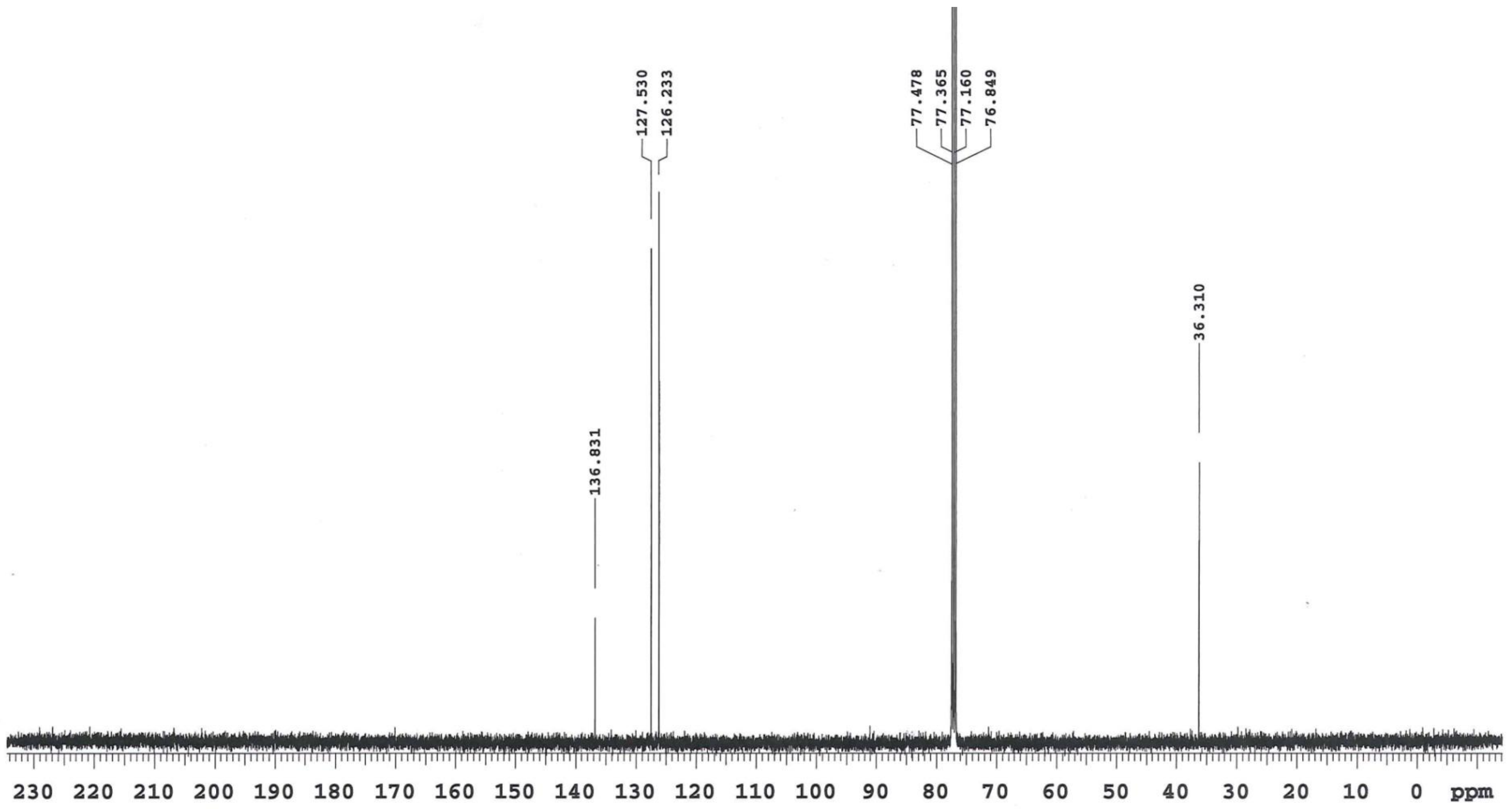

Supplementary Figure 22. ^{13}C-NMR spectrum of the isolated 9,10-dihydroanthracene after scaled-up photohydrogenation of anthracene in PhH/AcOEt/HCOOH (5/5/1: v/v/v), recorded in $CDCl_3$. (Relaxation Delay: 1.000 sec; Pulse: 45.0 degrees; 5000 repetitions).

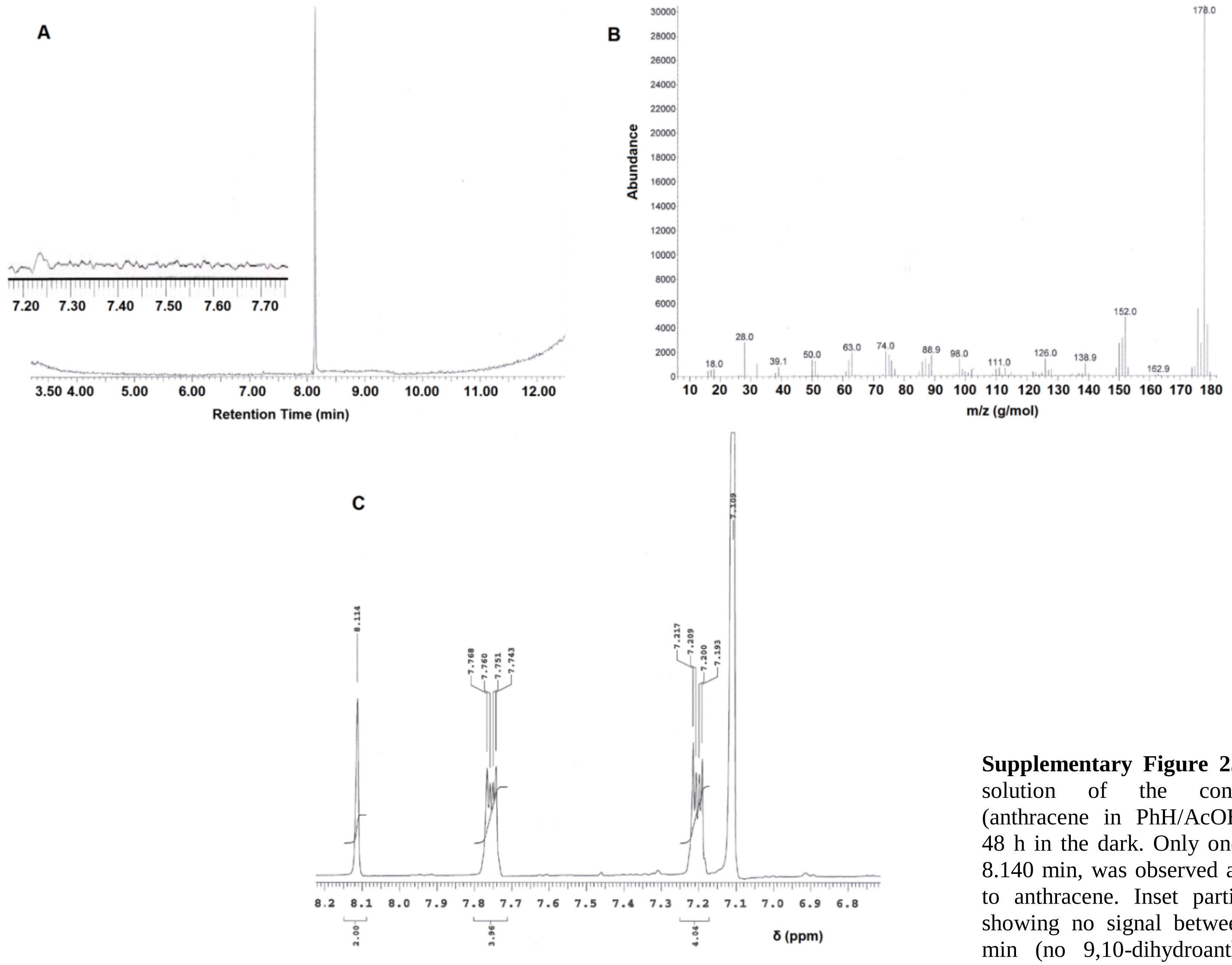

Supplementary Figure 23. (A) GC of the solution of the control experiment (anthracene in PhH/AcOEt/HCOOH) after 48 h in the dark. Only one peak, located at 8.140 min, was observed and it corresponds to anthracene. Inset partial chromatogram showing no signal between 7.20 and 7.70 min (no 9,10-dihydroanthracene formed). (B) Mass spectra of the anthracene signal of the chromatogram of panel A. (C) Partial ^{1}H-NMR (aromatic region) of the residual of control experiment (anthracene in PhH/AcOEt/HCOOH, 48 h in the dark) dissolved in $CDCl_3$. Note that only anthracene signals are present.

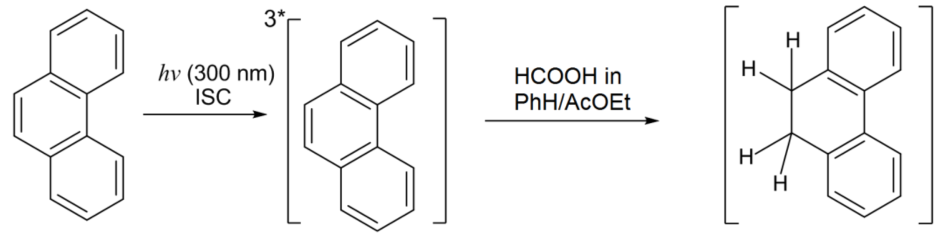

Supplementary Figure 24. Photoreaction of phenanthrene in a mixture of PhH/AcOEt/HCOOH (5/5/1: v/v/v) upon UV-irradiation (300 nm) to give 9,10-dihydrophenanthrene.

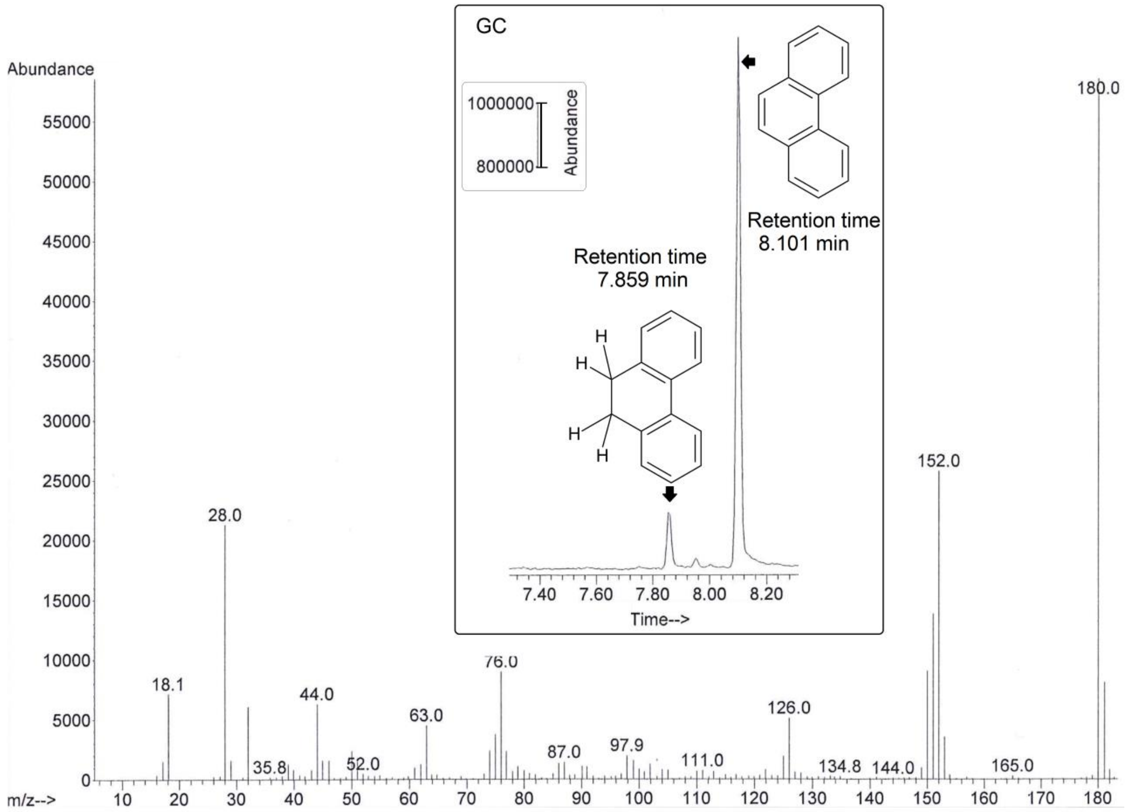

Supplementary Figure 25. MS of produced DHP (MW=180 g/mol) after photoreaction of phenanthrene in PhH/AcOEt/HCOOH (300 nm). Inset: Chromatogram (GC). Appearance of 9,10-dihydrophenanthrene (DHP). Retention time 7.859, was verified with commercially available DHP.

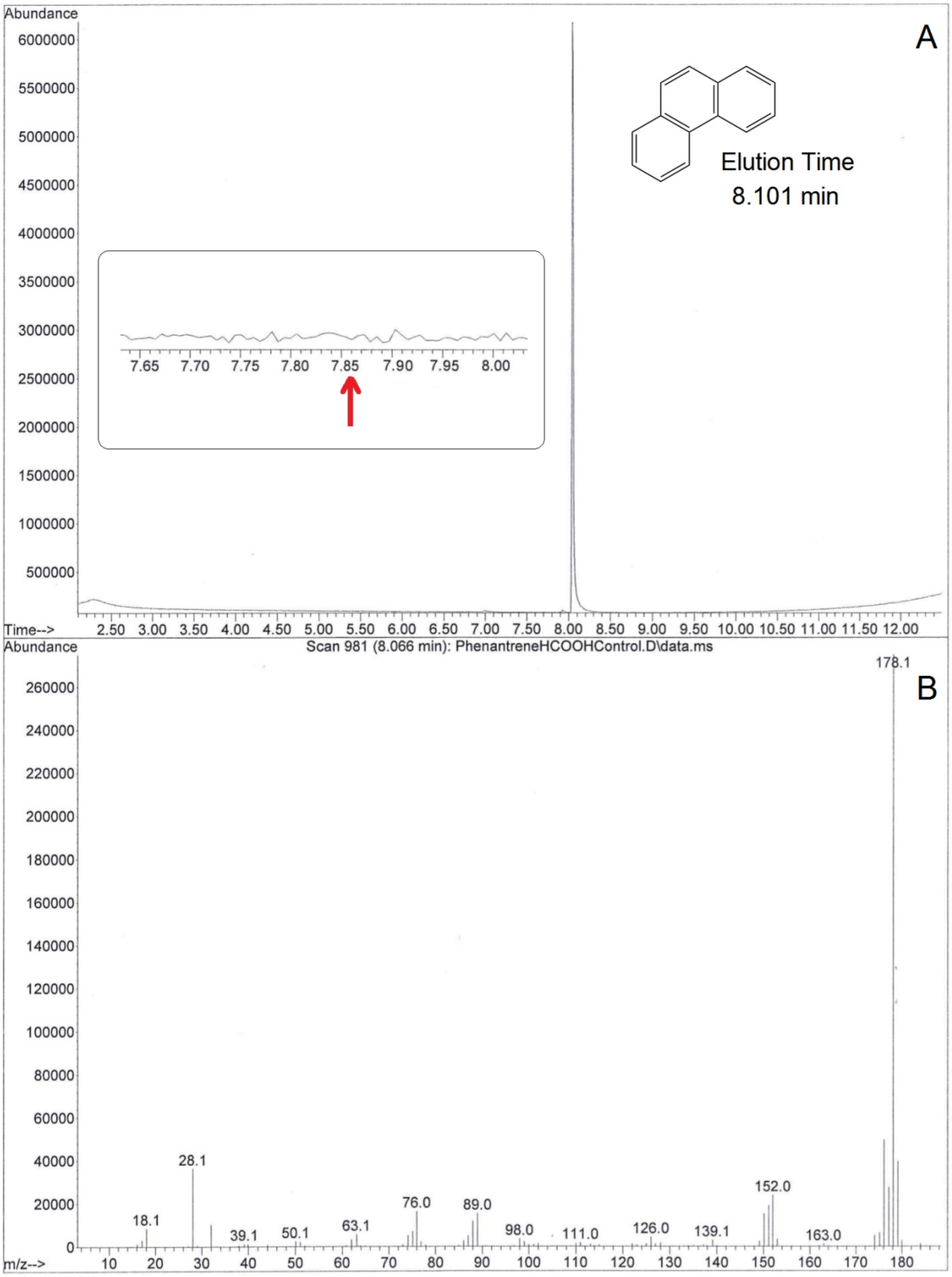

Supplementary Figure 26. (A) GC after control experiment: phenanthrene in PhH/AcOEt/HCOOH in the dark for 48 h. Inset: partial chromatogram exhibiting the lack of any signal corresponding to DHP. Red arrow showing the retention time commercially available DHP exhibits. (B) MS of the only signal appearing (retention time: 8.101 min) after the control experiment, corresponding to phenanthrene.

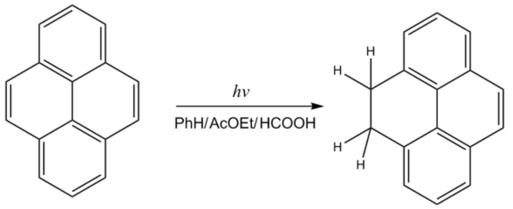

Supplementary Figure 27. Photohydrogenation of pyrene in PhH/AcOEt/HCOOH.

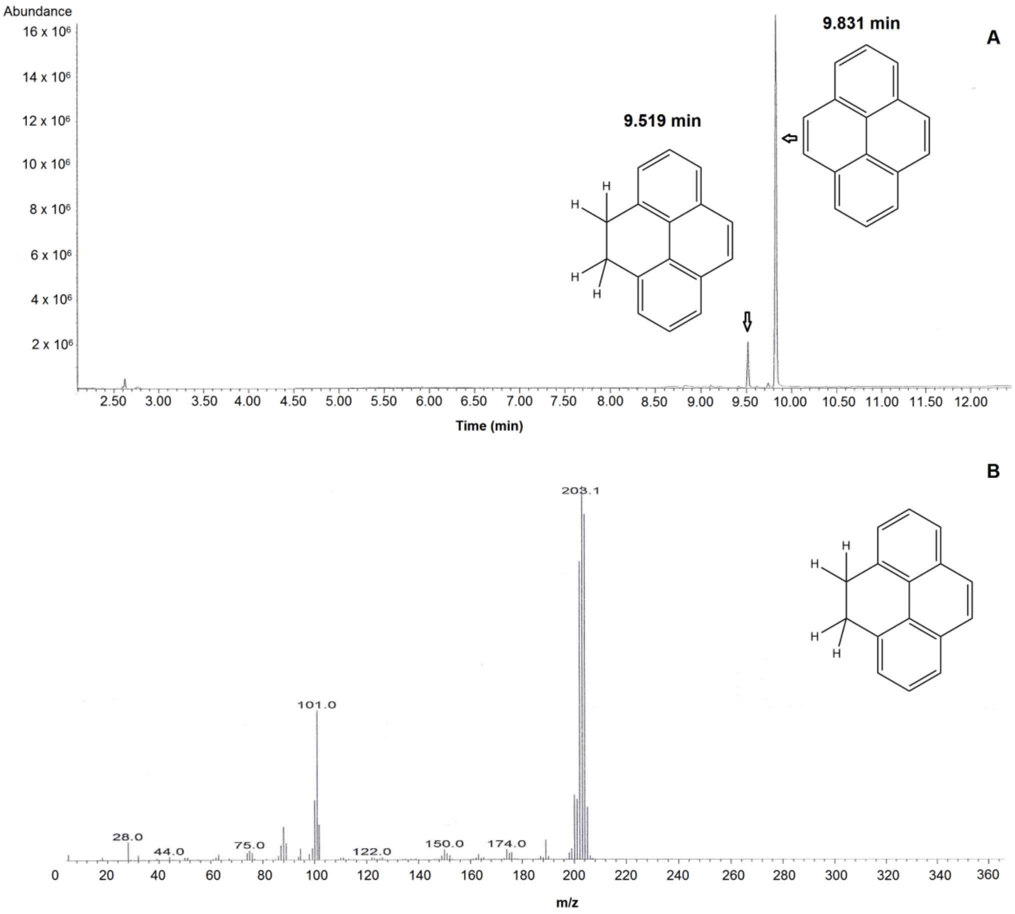

Supplementary Figure 28. (A) GC after photohydrogenation of pyrene and (B) MS of 4,5-DHPy.

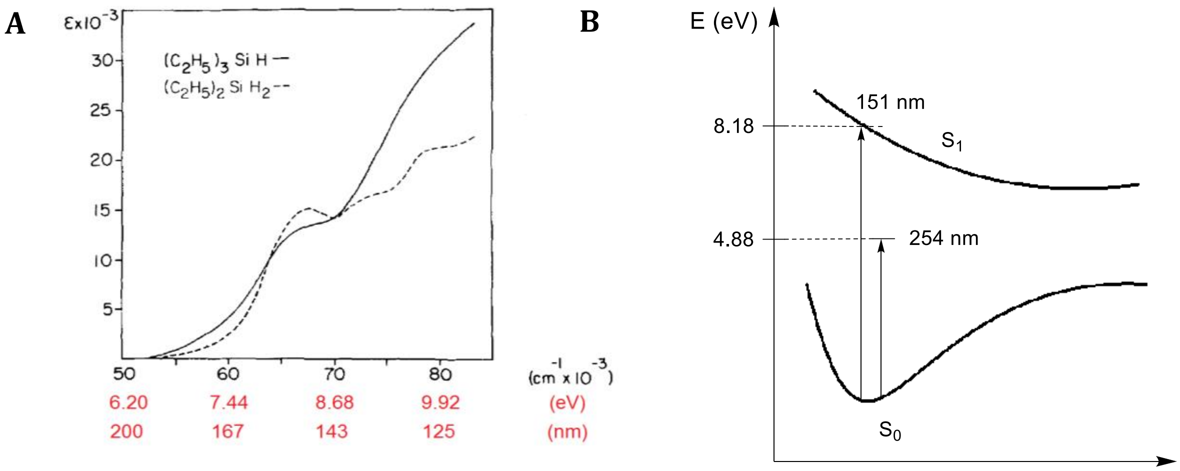

Supplementary Figure 29. (A) The vacuum UV spectrum of Et_3SiH recorded by Roberge *et al.*[2] (B) schematic drawing of the insufficient energy provided by the 254 nm irradiation for photolysis of Et_3SiH.

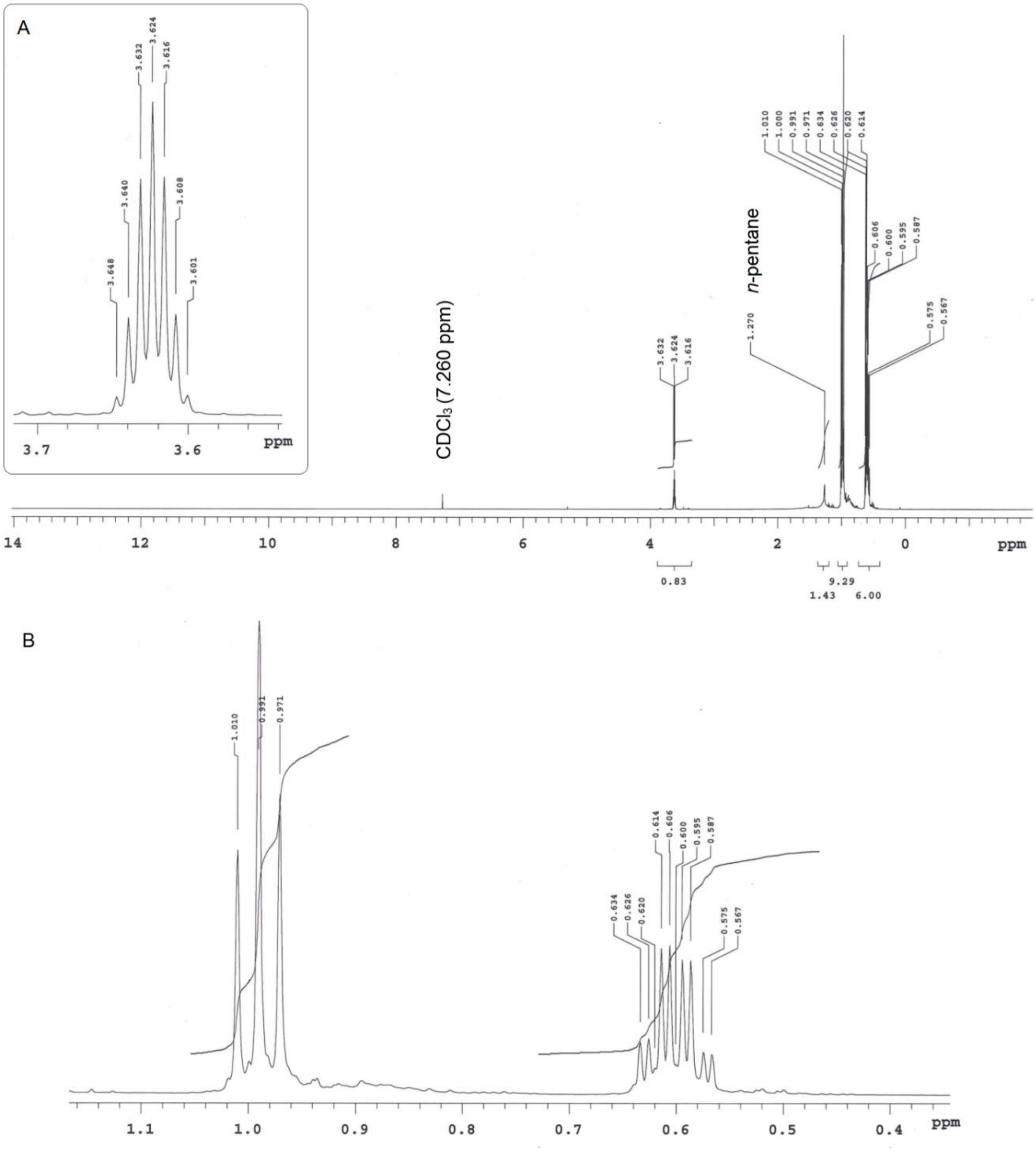

Supplementary Figure 30. ^{1}H-NMR spectrum of Et_3SiH after irradiation in *n*-pentane for 24 h at 254 nm, recorded in $CDCl_3$. (A) Full ^{1}H-NMR spectrum; inset magnification of the ^{1}H(Si) signal. Bottom (B) partial ^{1}H-NMR spectrum showing the methylene and methyl protons of Et_3SiH.

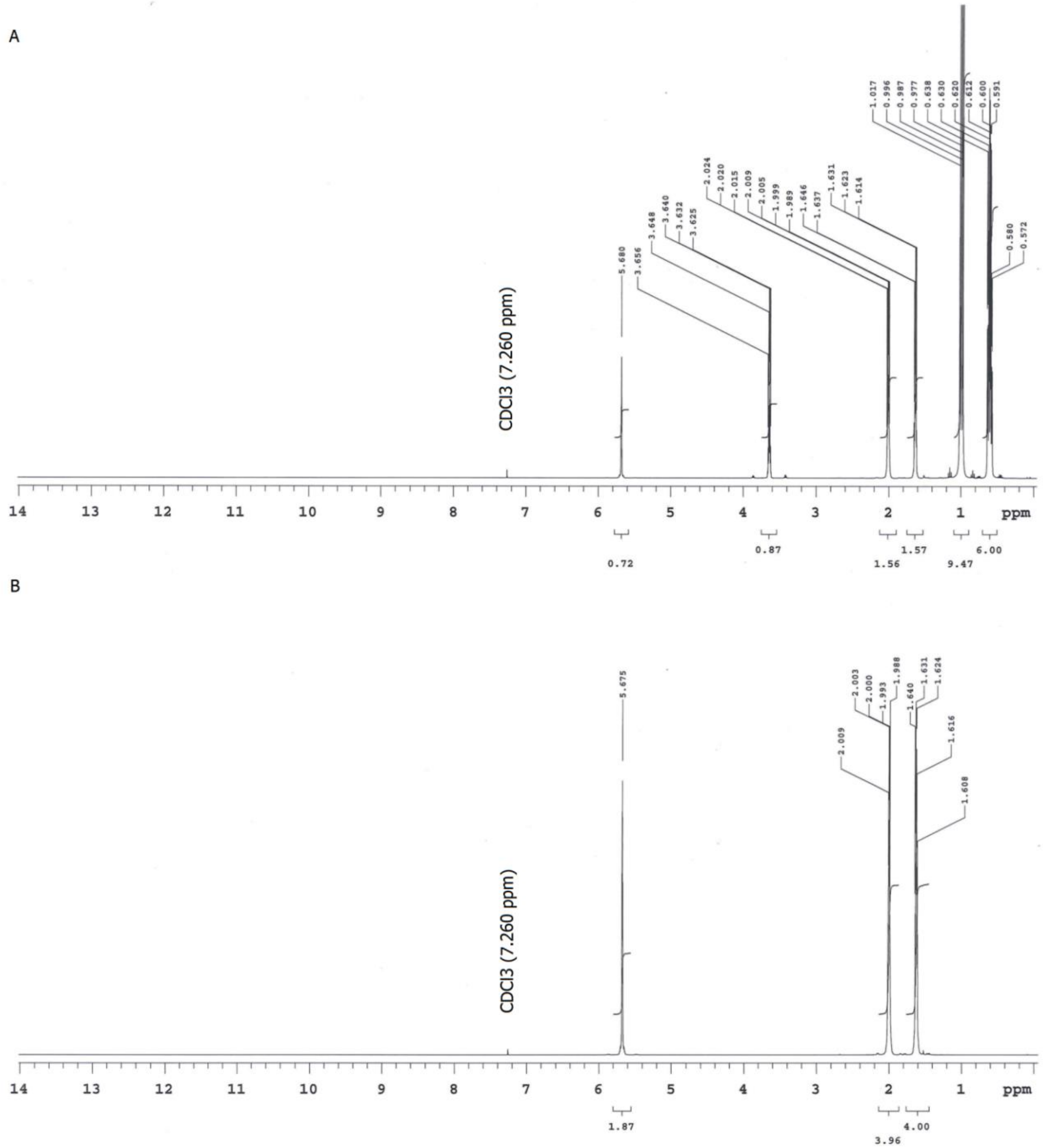

Supplementary Figure 31. (A) ^{1}H-NMR spectrum after irradiation of Et_3SiH in cyclohexene at 254 nm recorded in $CDCl_3$. (B) ^{1}H-NMR spectrum of cyclohexene recorded in $CDCl_3$.

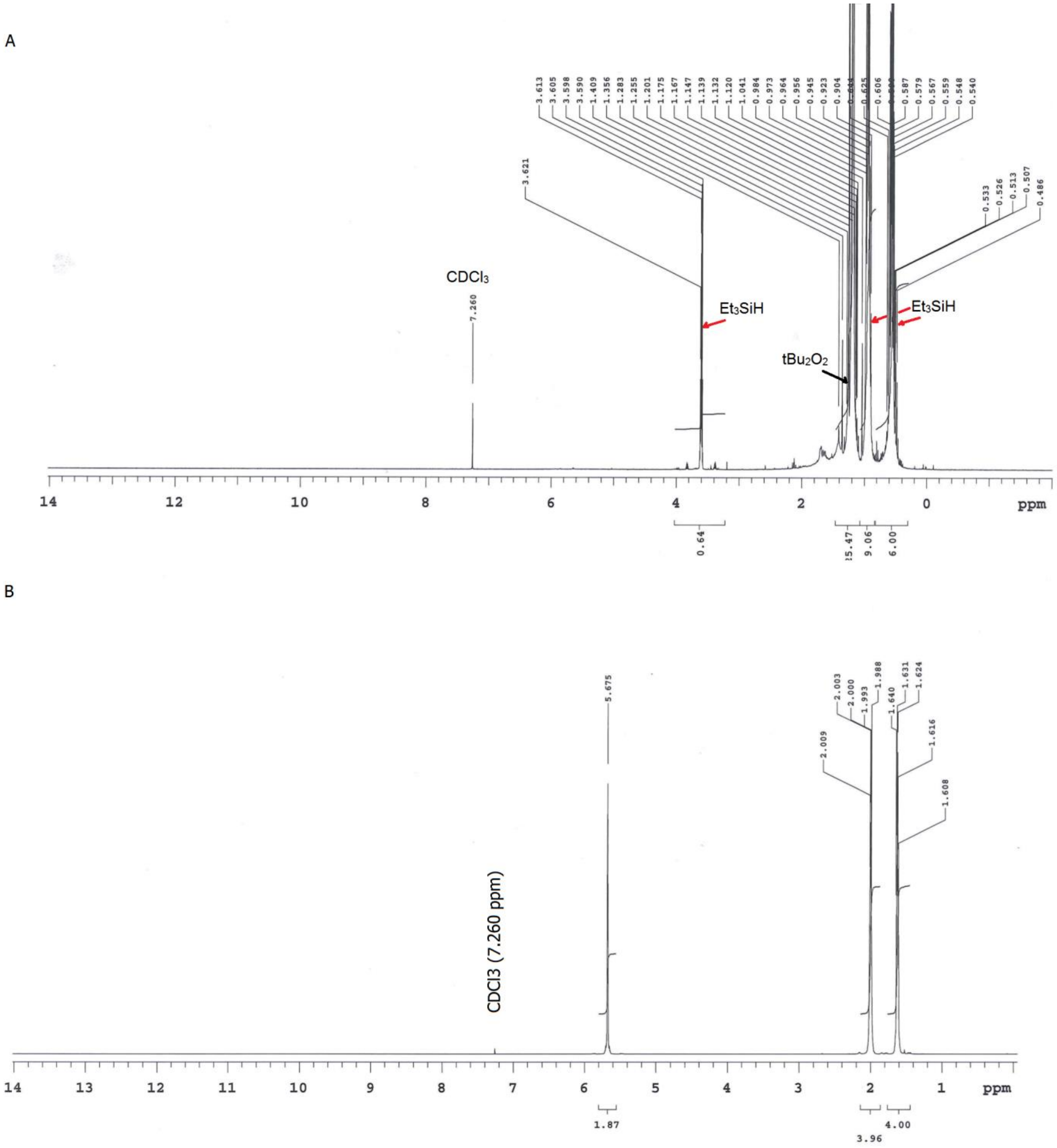

Supplementary Figure 32. (A) ^{1}H-NMR spectrum of the reaction mixture obtained after irradiation of cyclohexene in excess of tBu$_2$O$_2$ and Et$_3$SiH at 254 nm for 24 h, and (B) ^{1}H-NMR spectrum of pure cyclohexene. Both spectra were recorded in CDCl$_3$.

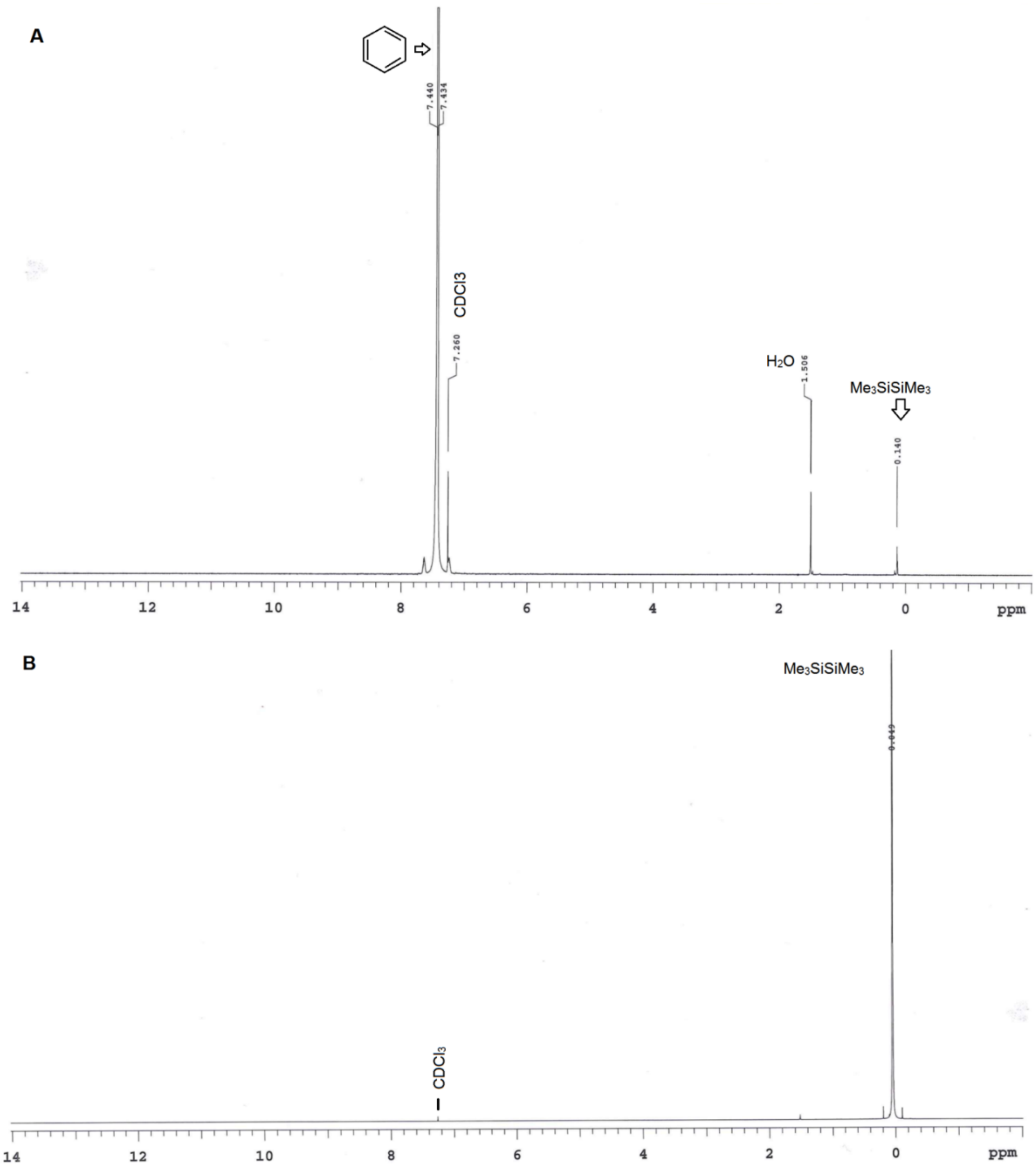

Supplementary Figure 33. (A) ^{1}H-NMR spectrum of the solution obtained after 48 h irradiation of Si_2Me_6 in benzene ($[Si_2Me_6]$= 12 mM) at 254 nm, and (B) ^{1}H-NMR spectrum of pure Si_2Me_6. Both spectra were recorded in $CDCl_3$.

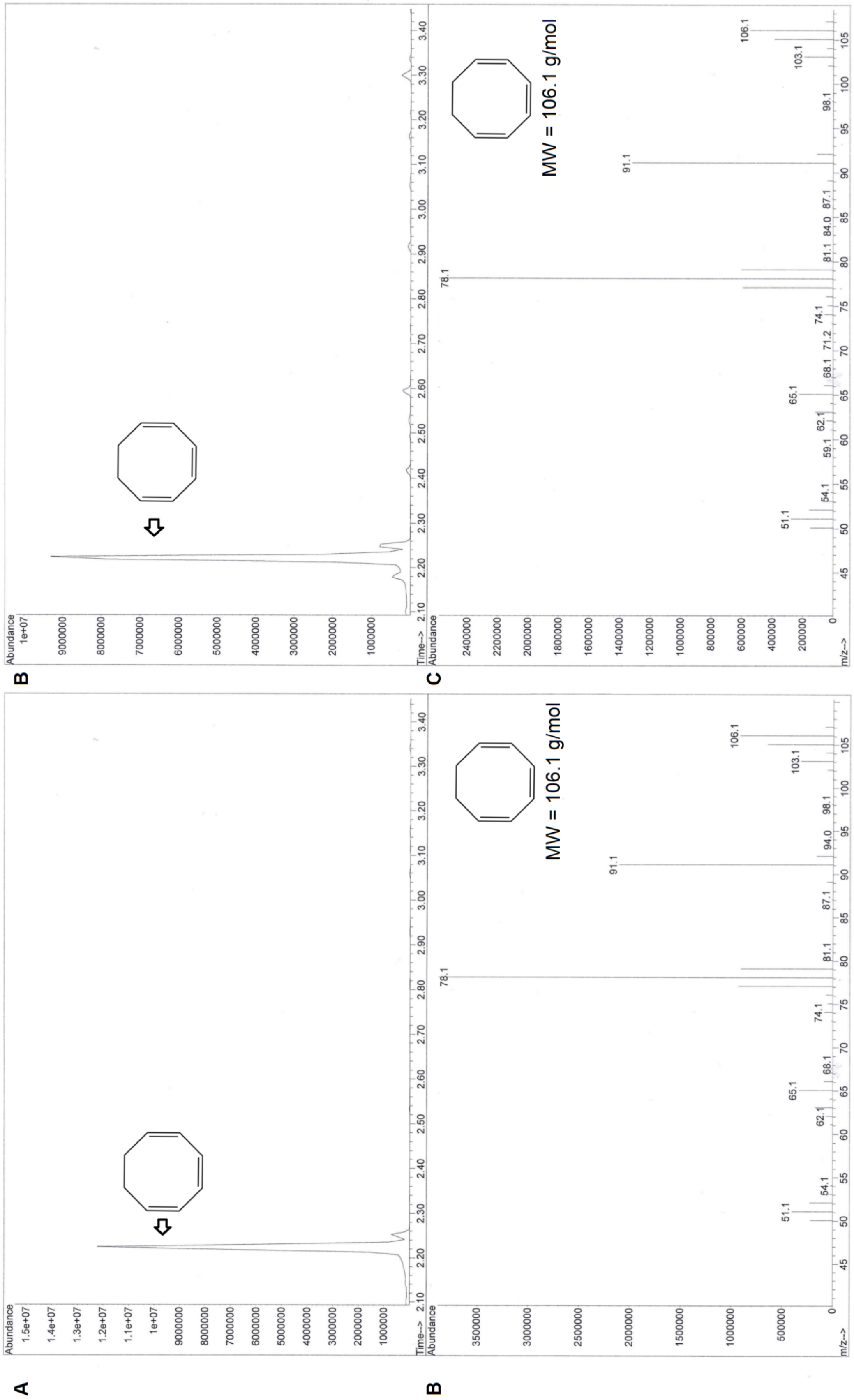

Supplementary Figure 34. GC of (A) the starting 625 mM solution of 1,3,5-cyclooctatriene in neat Et_3SiH, and (B) of the solution obtained after 24 h irradiation of the starting solution at 254 nm. (C) MS of the GC peak at 2.220 min corresponding to 1,3,5-cyclooctatriene identified before irradiation, and (D) MS of the corresponding peak identified after irradiation.

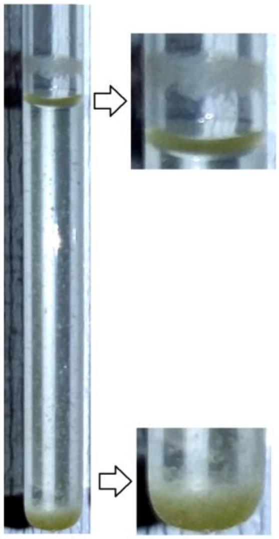

Supplementary Figure 35. Photo of NMR quartz tube containing the reaction mixture obtained after 24 h irradiation at 254 nm of a 340 mM solution of 1,3,5-cyclooctatriene, in a 7-fold excess of Et_3SiH, and a 10.5-fold excess of $t$$Bu_2O_2$. Note the yellow precipitate formed.

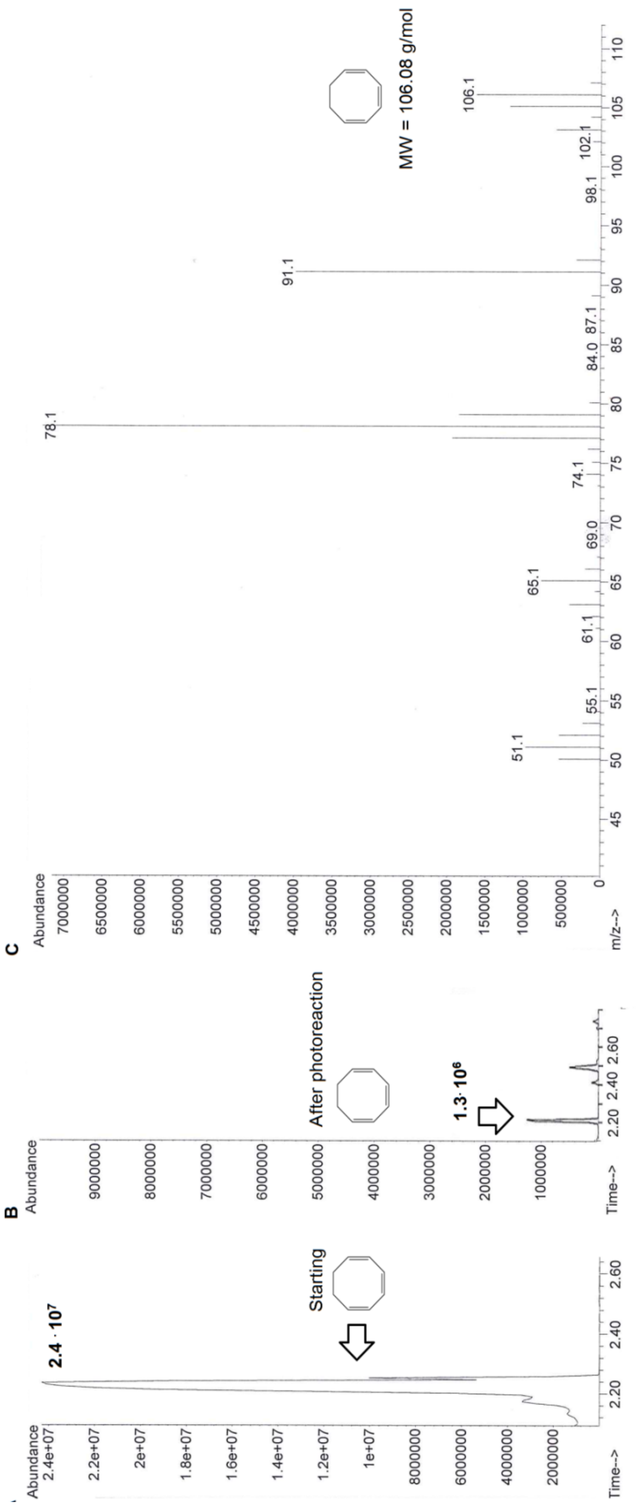

Supplementary Figure 36. GC of (A) starting 340 mM solution of 1,3,5-cyclooctatriene, in a 7-fold excess of Et_3SiH, and a 10.5-fold excess of tBu_2O_2, and (B) of the supernatant solution obtained after 24 h of irradiation at 254 nm. (C) Mass spectrum of the GC peak appearing at 2.20 min (see GC of A and B) corresponding to 1,3,5-cyclooctatriene with M.W. = 106.08 g/mol.

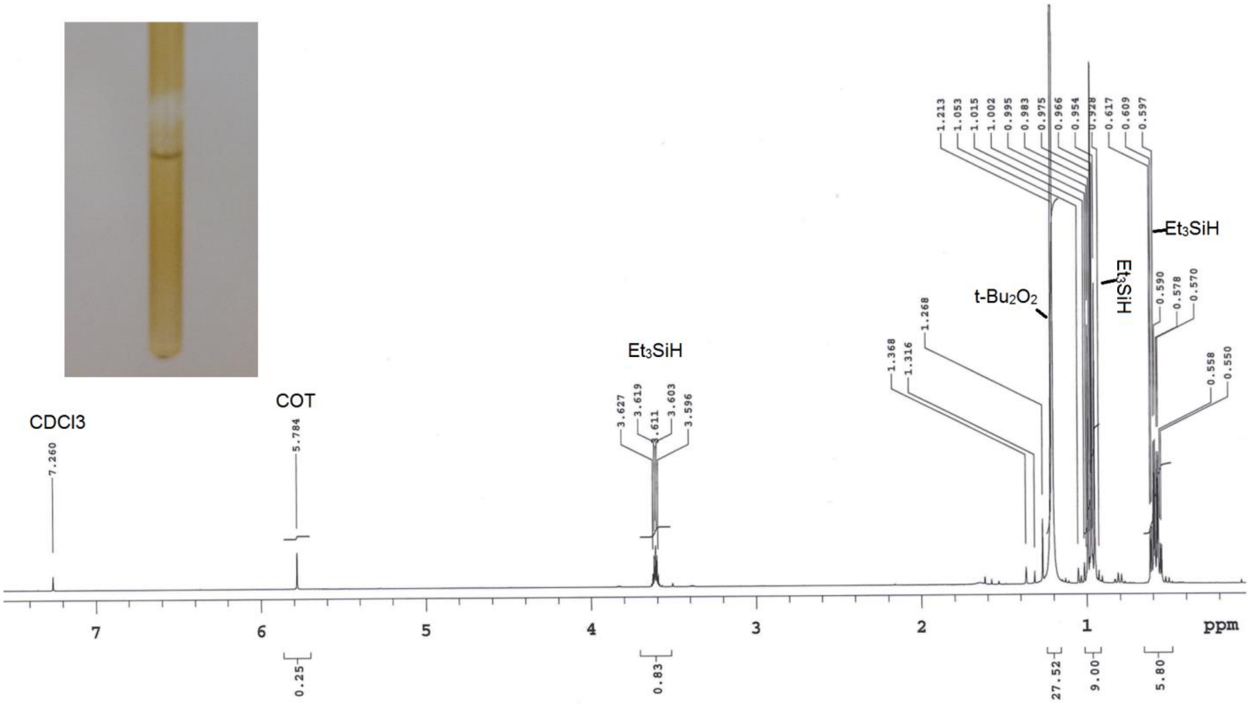

Supplementary Figure 37. ^{1}H-NMR spectrum obtained after irradiation of a 0.340 M COT solution in excess of *t*Bu_2O_2 and Et_3SiH for 24 h. Inset: photo of the quartz NMR tube containing the reaction mixture after irradiation. Note the sticky yellow polymer formed on the surface of the tube.

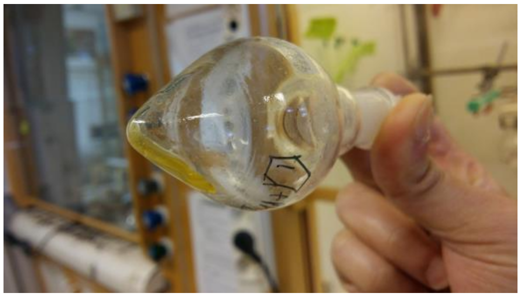

Supplementary Figure 38. Yellow viscous insoluble product of the photoreaction of cyclohexene and Ph_3CH (irradiation at 254 nm for 24 h).

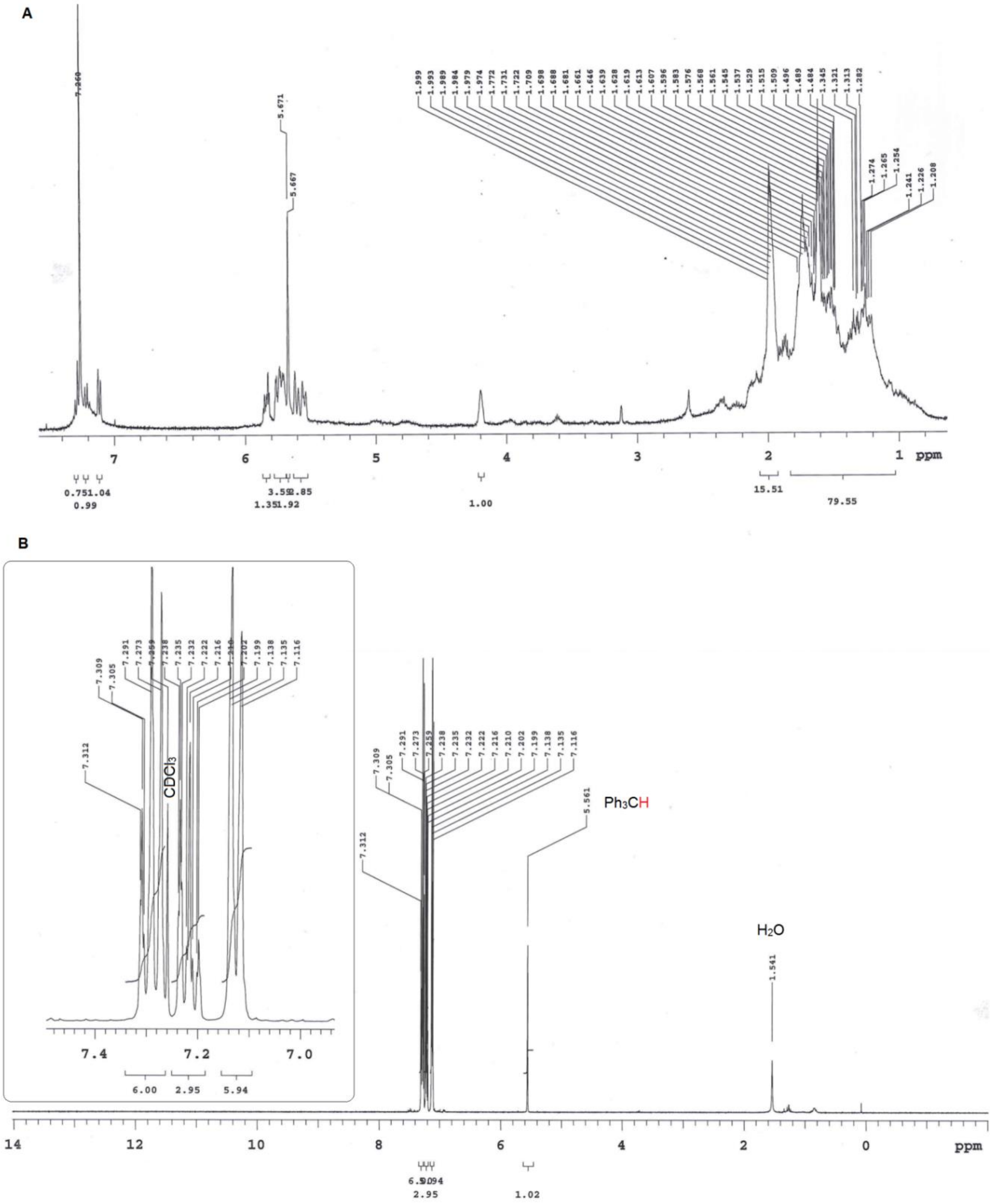

Supplementary Figure 39. ^{1}H-NMR spectrum of (A) reaction mixture obtained after 24 h irradiation at 254 nm of a 12.5 mM Ph_3CH solution in cyclohexene, and (B) of unreacted Ph_3CH, both recorded in $CDCl_3$.

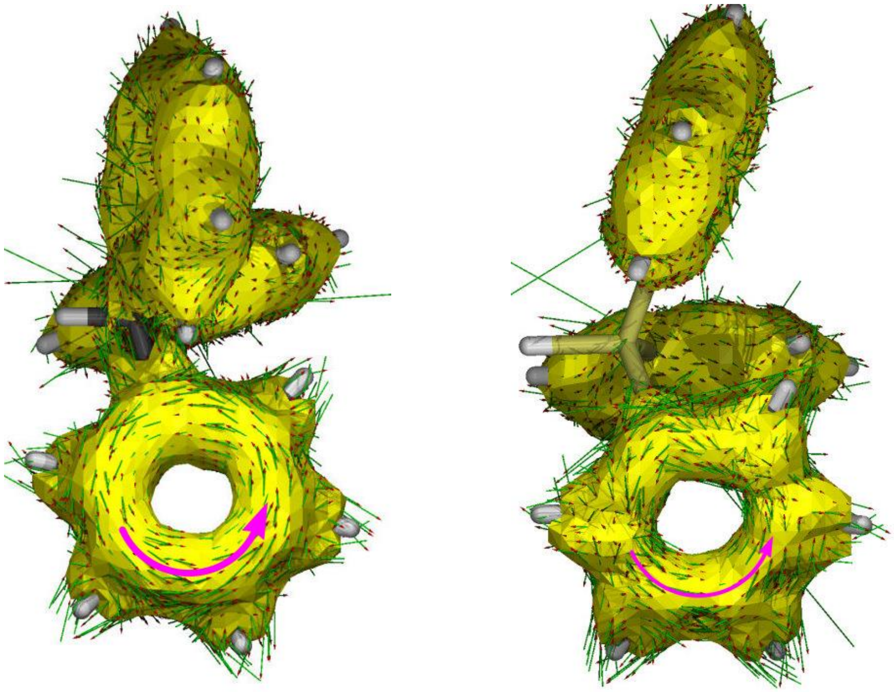

Supplementary Figure 40. ACID plots of Ph_3CH (left) and Ph_3SiH (right) showing antiaromatic ring currents. Calculated with B3LYP/6-311+G(d,p).

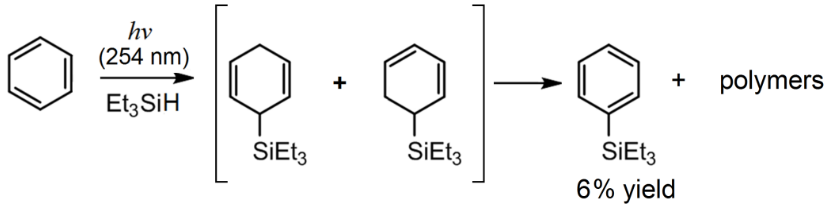

Supplementary Figure 41. Photo(hydro)silylation of benzene.

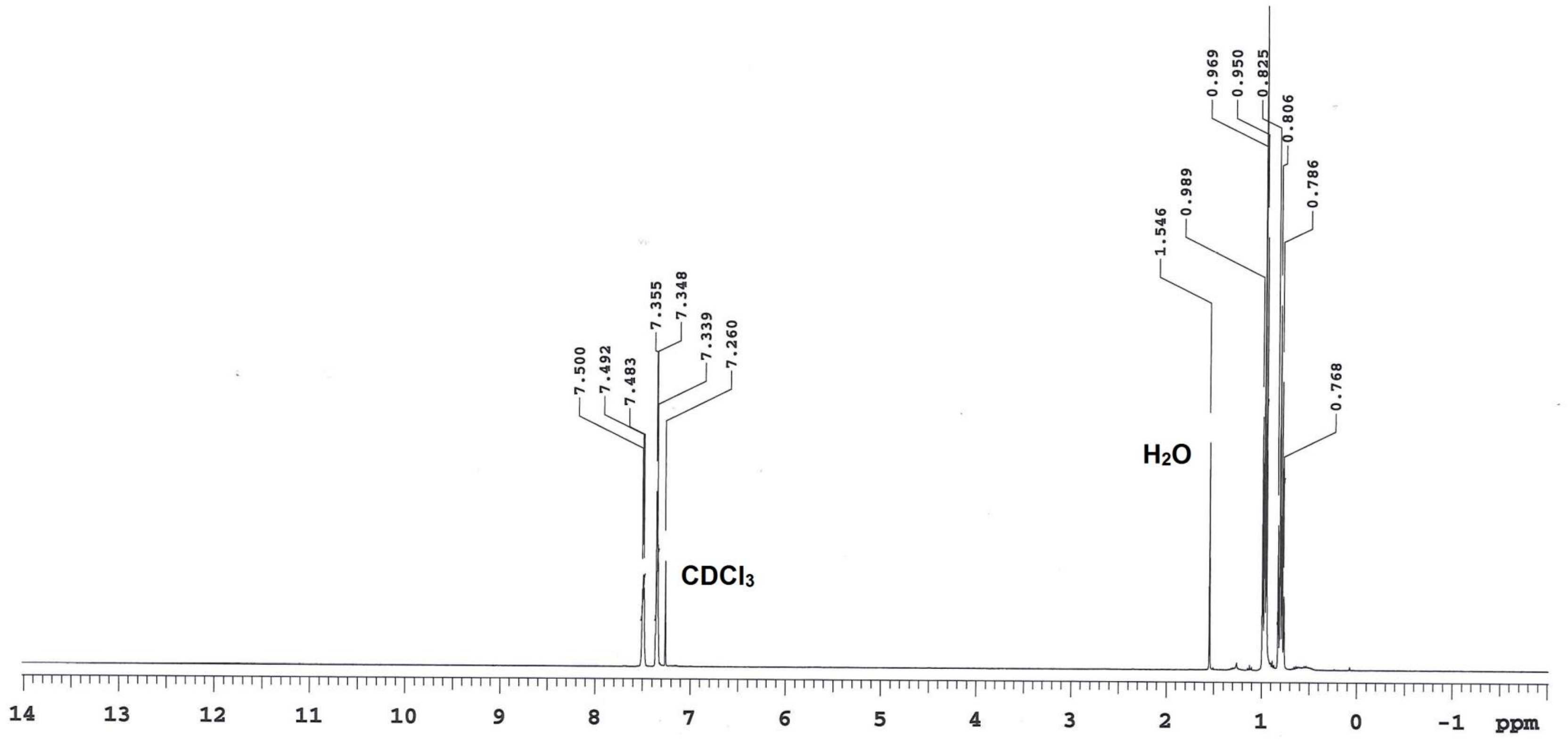

Supplementary Figure 42. ^{1}H-NMR spectrum of isolated $PhEt_3Si$ produced after irradiation of a 12 mM Et_3SiH benzene solution at 254 nm for 24 h, recorded in $CDCl_3$

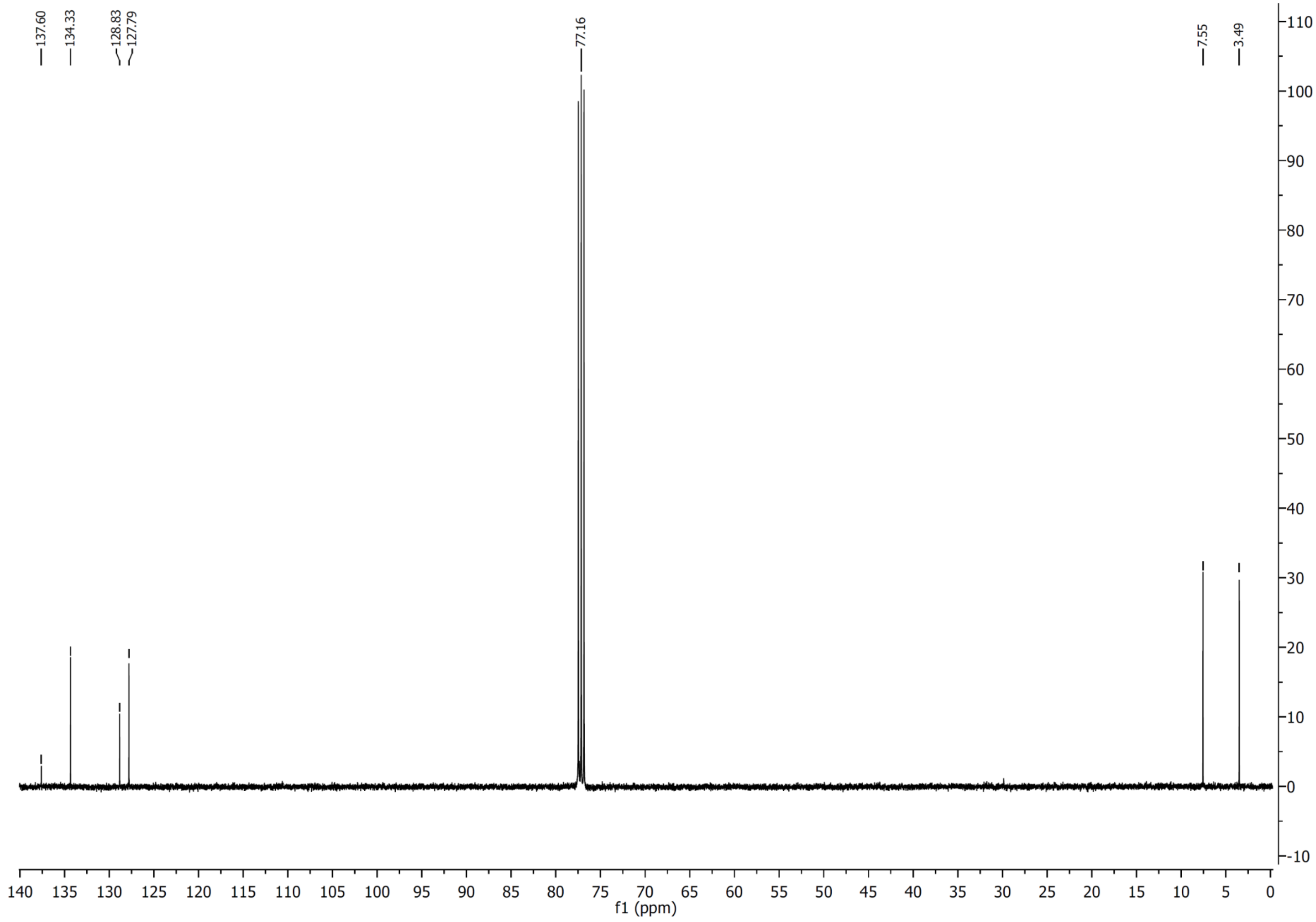

Supplementary Figure 43. ^{13}C-NMR spectrum of isolated $PhEt_3Si$ produced after irradiation of a 12 mM Et_3SiH benzene solution at 254 nm for 24 h, recorded in $CDCl_3$

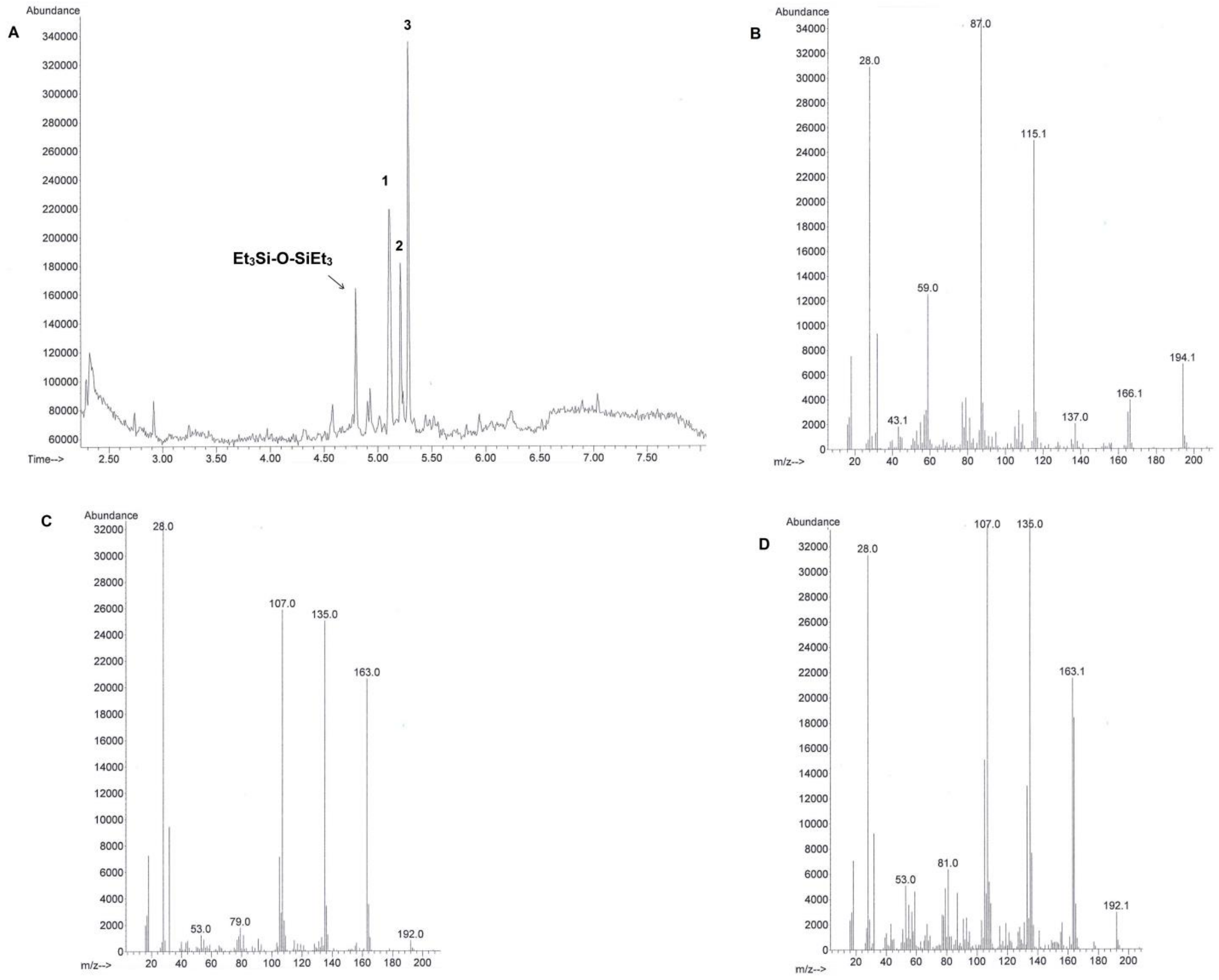

Supplementary Figure 44. (A) GC after photoreaction of a benzene solution in Et_3SiH (see alternative method above). Identified products: 1: a product exhibiting a molecular ion of 194 g/mol (product No 1) possibly corresponding to a monohydrosilylated benzene derivative (**Supplementary Figures 41**) in addition to phenyl triethylsilane was identified. (B) MS of the monohydrosilylated benzene derivative (product 1) retention time verified with synthesized $PhEt_3Si$. (C) MS of product 2 of panel A: $PhEt_3SiH$ and (D) MS of product 3 of panel A.

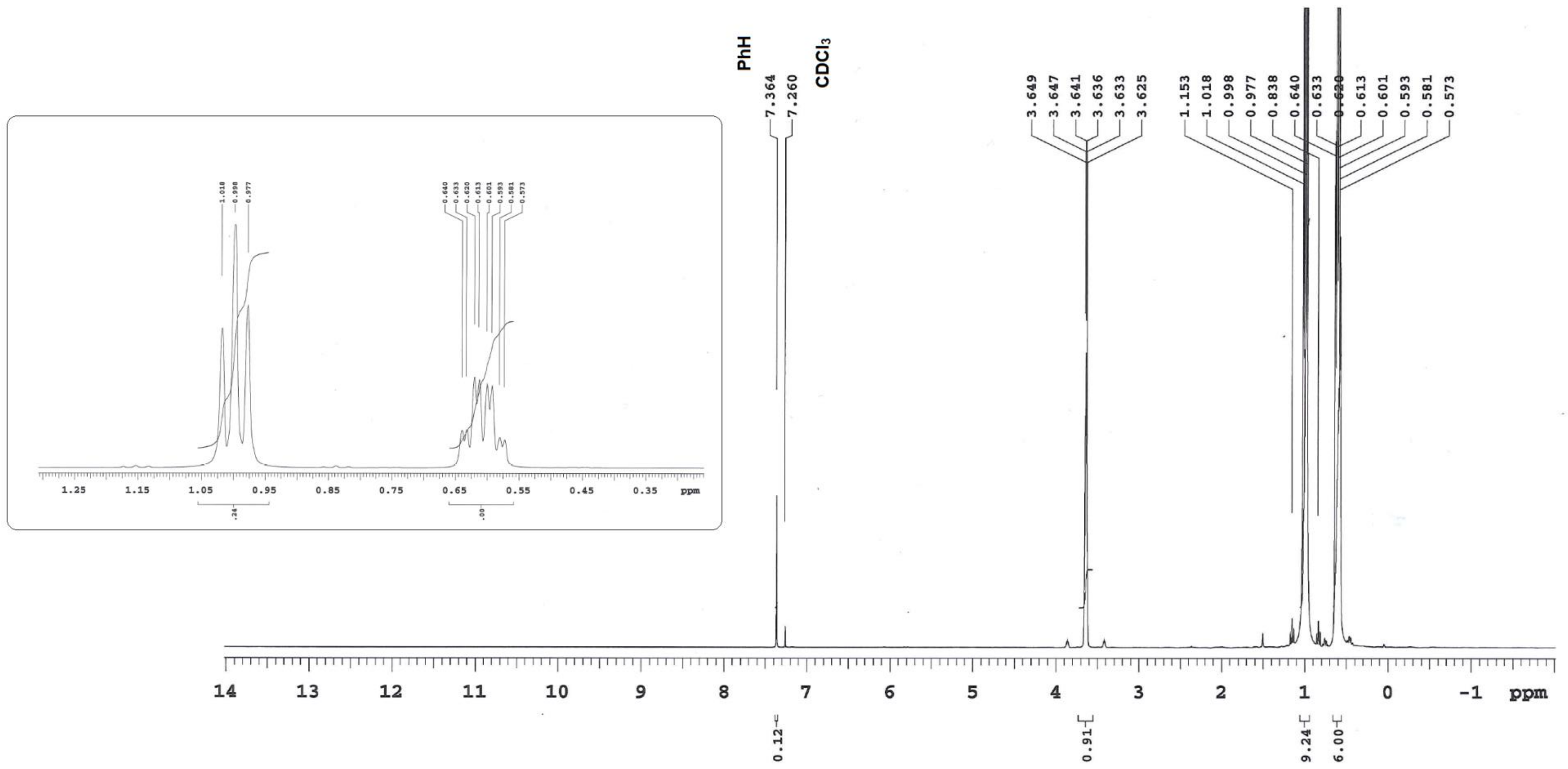

Supplementary Figure 45. ^{1}H-NMR spectrum for the control experiment (PhH and Et_3SiH, 48 h in the dark) in $CDCl_3$. Note that only Et_3SiH and benzene can be observed. Inset: a magnification of a part of the aliphatic region showing the proton signals of the ethyl groups of Et_3SiH.

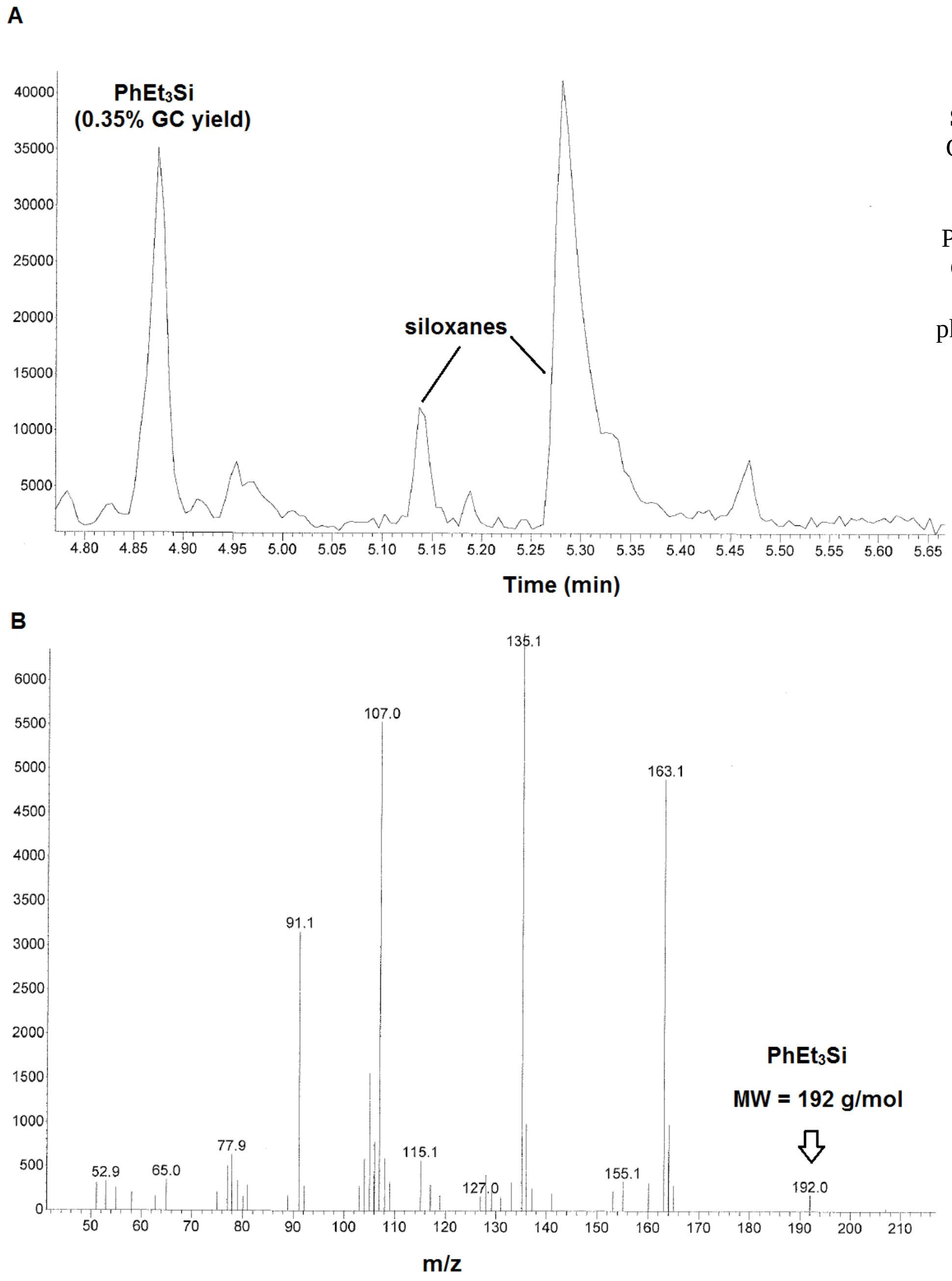

Supplementary Figure 46.(A) GC obtained after 24 irradiation of an aerated 12 mM benzene solution of Et_3SiH at 254 nm. $PhEt_3Si$ was obtained in a 0.35% GC yield and various siloxanes were identified. (B) MS of the photosilylation product ($PhEt_3Si$).

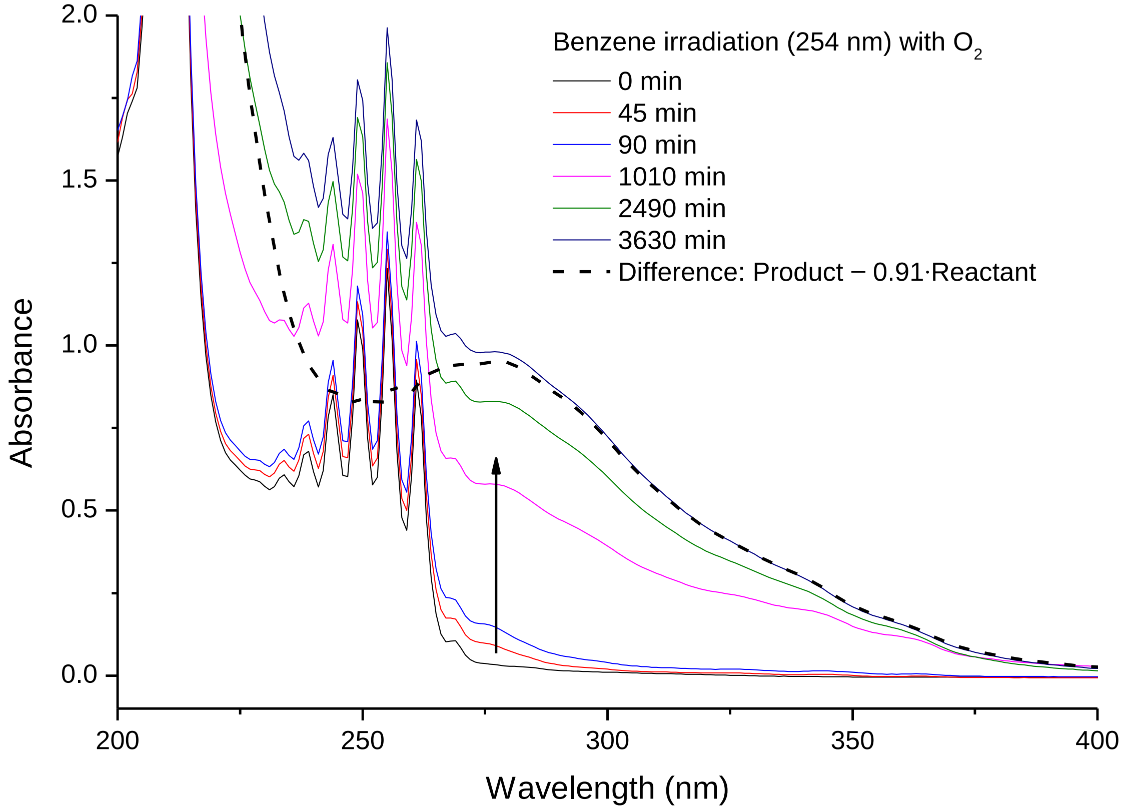

Supplementary Figure 47. Changes in benzene absorbance with irradiation time (254 nm) in the presence of O_2

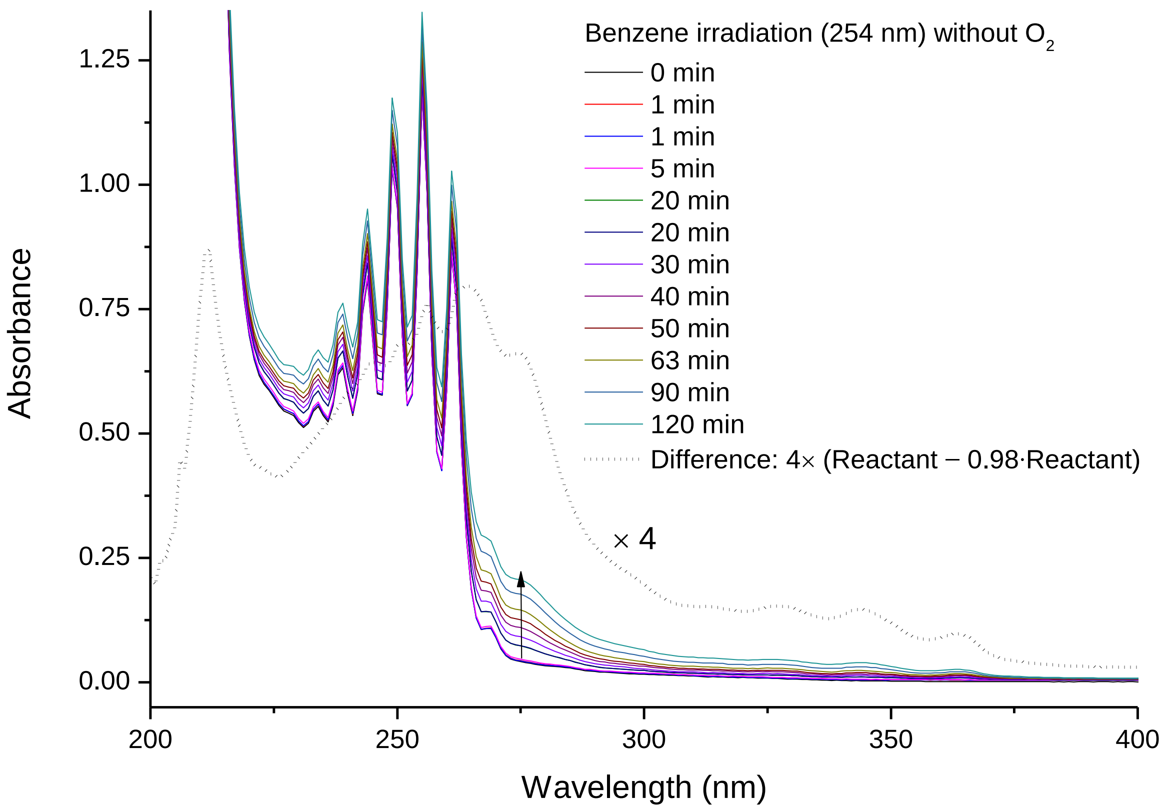

Supplementary Figure 48. Changes in benzene absorbance with irradiation time (254 nm) in the absence of O_2

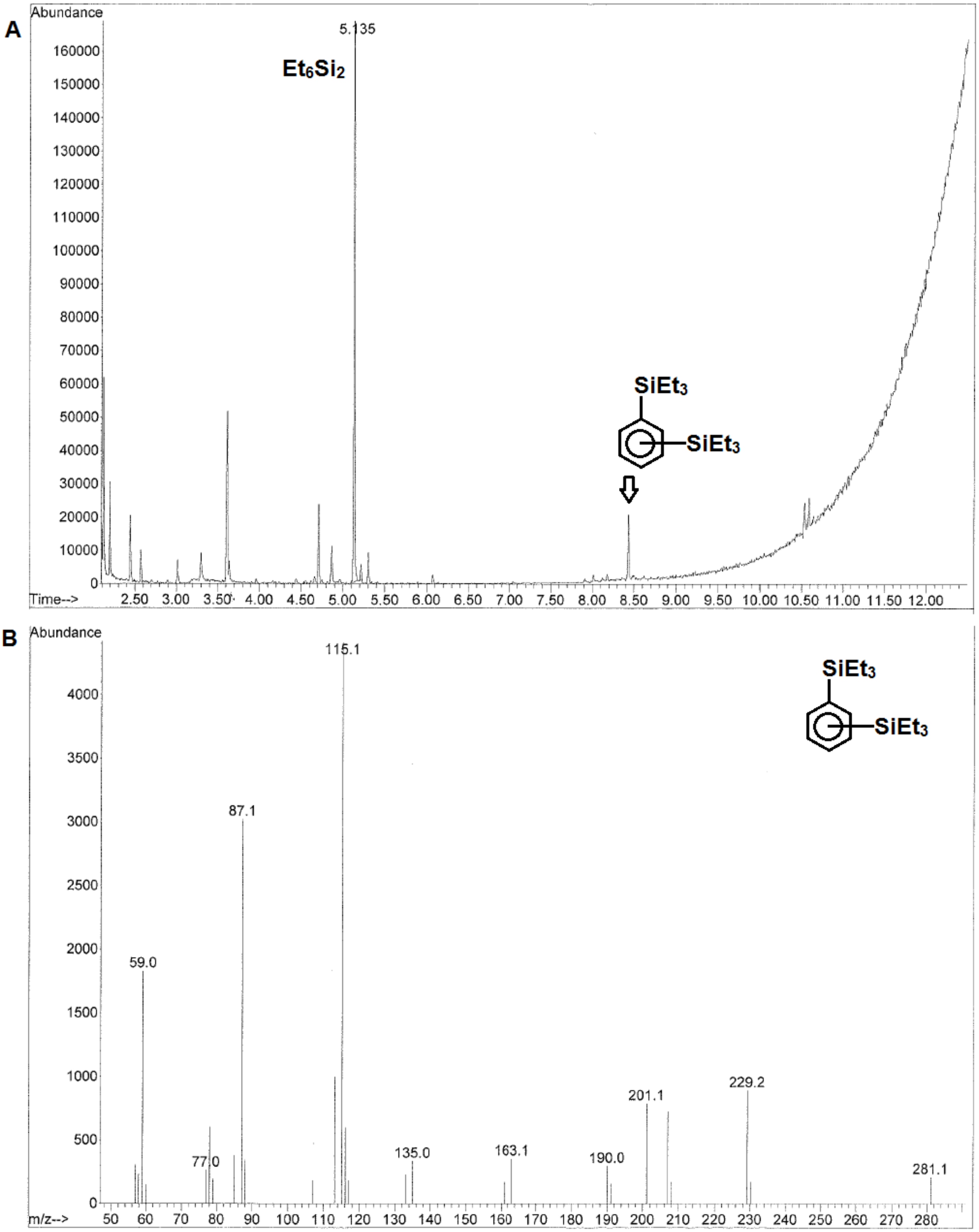

Supplementary Figure 49. (A) GC obtained after irradiation of an n-heptane solution of benzene in excess of Et_3SiH using monochromatic irradiation at λ=254 nm (Xe lamp). (B) MS of a di-triethylsilylbenzene identified though GC.

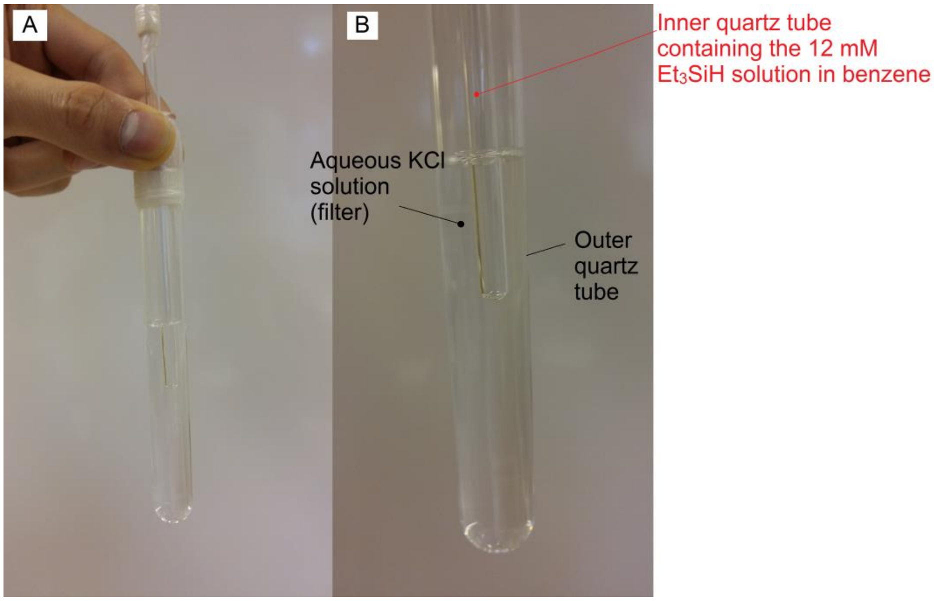

Supplementary Figure 50. (A) Photograph depicting a NMR quartz tube (inner tube) immersed in a bigger quartz tube (RQV-5 Rayonet; Ø 13 mm) containing a 10% (w/v) KCl aqueous solution acting as a filter of light with λ in the region 180 to 200 nm. (B) A magnification of the photo in A.

Supplementary Figure 51. (A) GC obtained after irradiation of a 12 mM benzene solution of Et_3SiH at 254 nm for 24 h using a KCl_{aq} filter. Main product was $PhEt_3Si$ with a GC-MS yield of ~6%. (B) MS of the obtained $PhEt_3Si$.

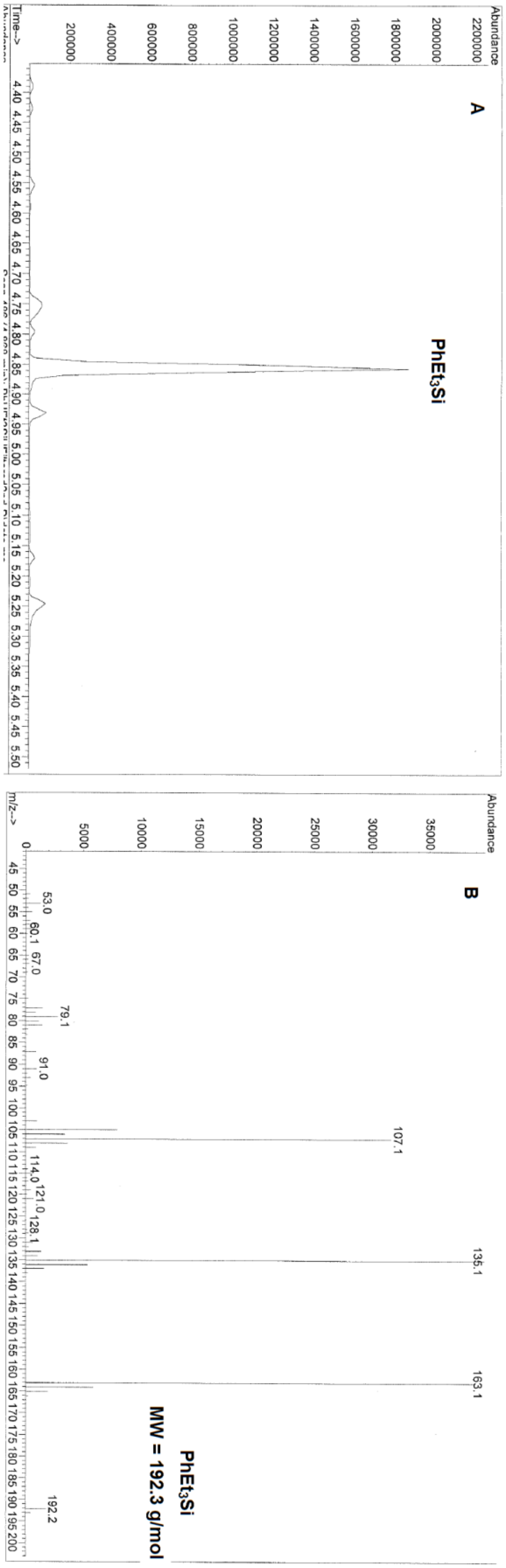

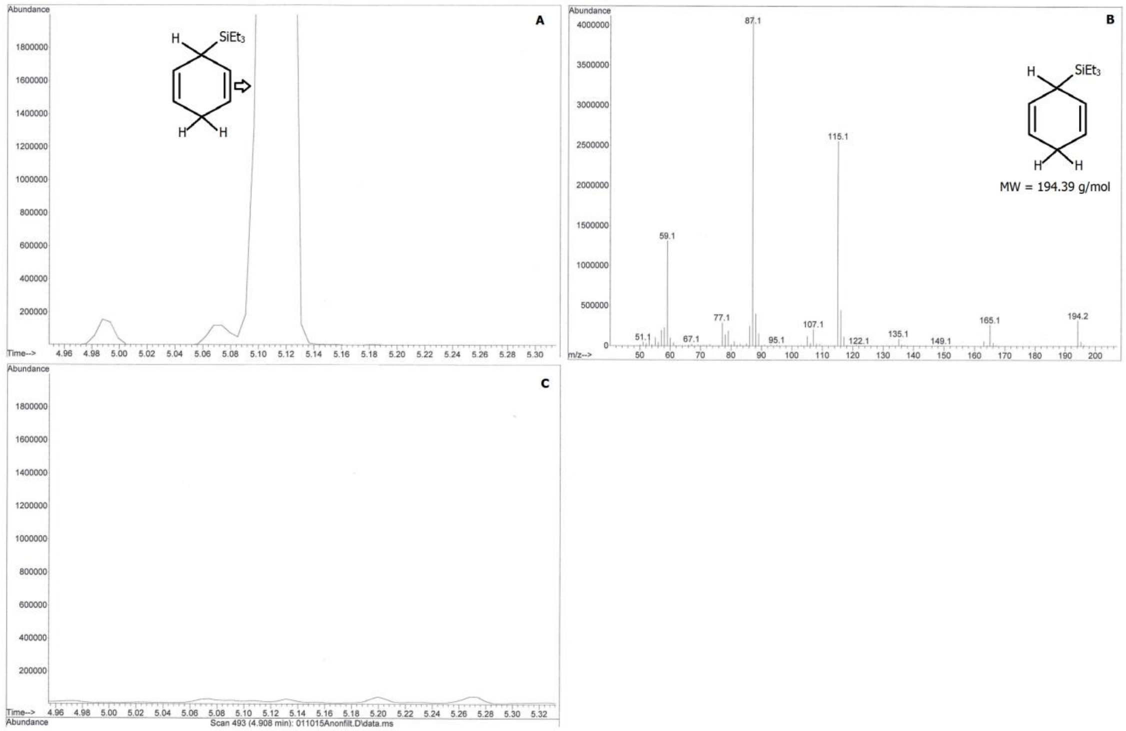

Supplementary Figure 52. (A) GC of the 41.2 mM starting solution triethylsilylcyclohexa-1,4-diene (before irradiation). (B) MS of triethylsilylcyclohexa-1,4-diene. (C) GC after irradiation for 24 h at 254 nm in *n*-heptane.

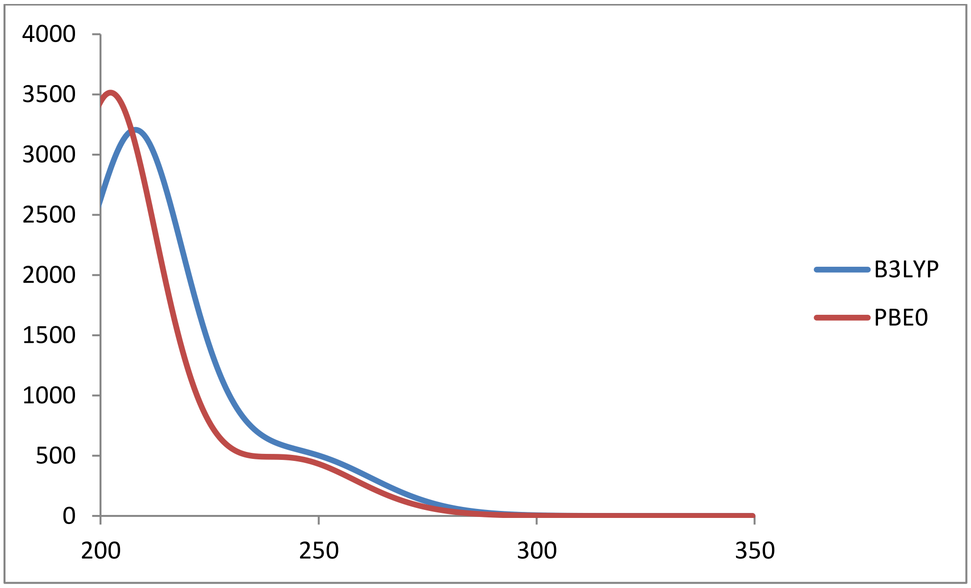

Supplementary Figure 53. TD-DFT spectrum triethylsilylcyclohexa-1,4-diene with B3LYP and PBE0 and the 6-311+G(2d,p) basis set.

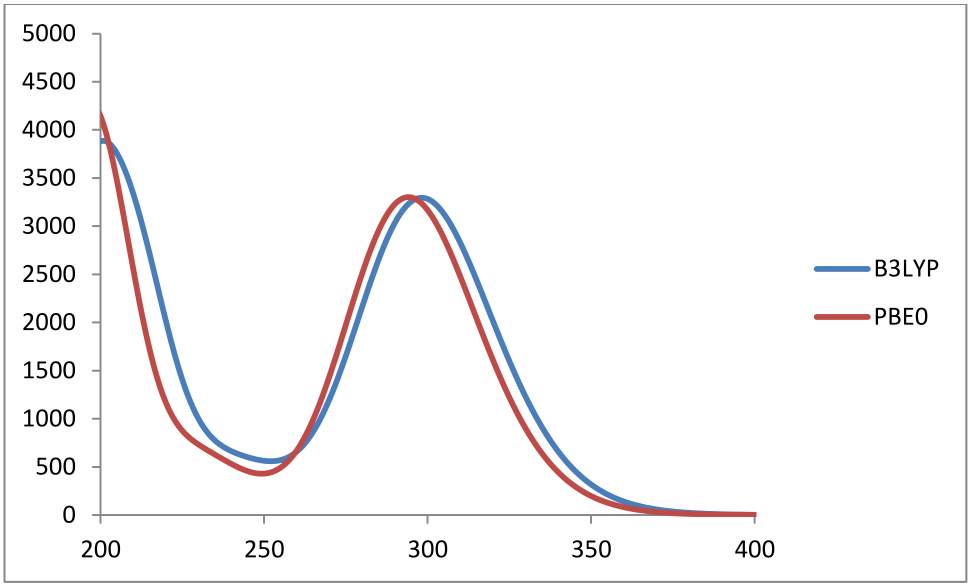

Supplementary Figure 54. TD-DFT spectrum of triethylsilylcyclohexa-1,3-diene with B3LYP and PBE0 and the 6-311+G(2d,p) basis set.

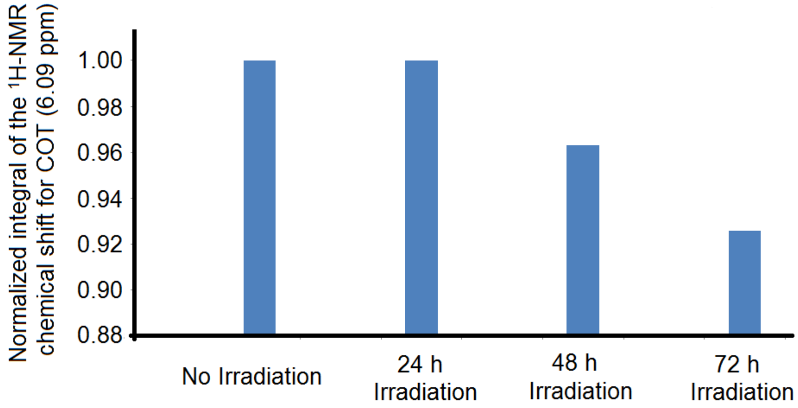

Supplementary Figure 55. Column chart showing the ^{1}H-NMR normalized integral for COT (6.09 ppm) for non-irradiated solution and for the solutions obtained after 24, 48, and 72 h of irradiation at 254 nm in a 10-fold excess of Et_3SiH (for the non-irradiated sample the integral of the ^{1}H-NMR signal was 0.17).

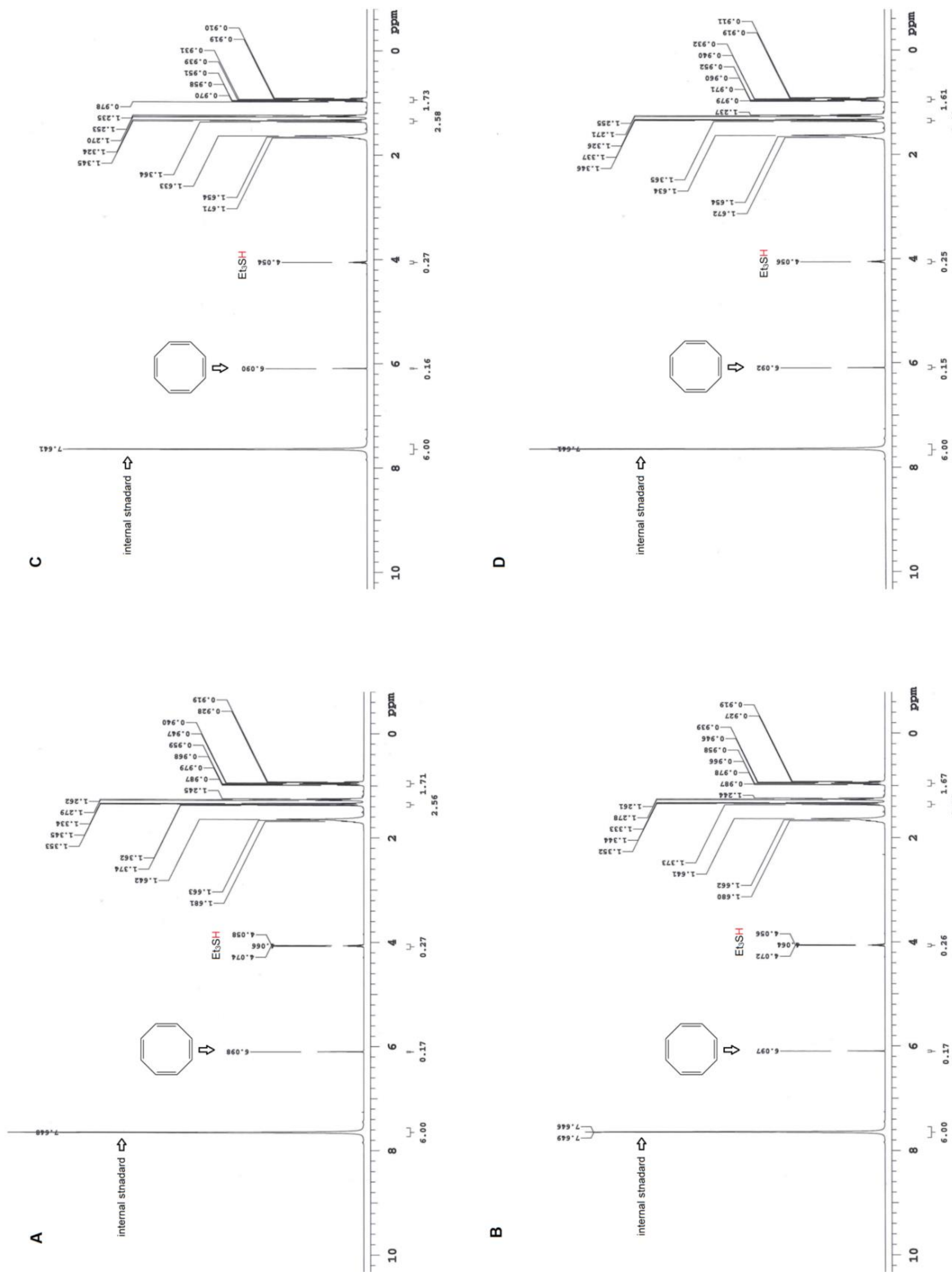

Supplementary Figure 56. ^{1}H-NMR spectrum of (A) a solution of COT (0.313 mM) and Et_3SiH (3.130 mM) in *n*-heptane without irradiation , (B) after irradiation at 254 nm for 24 h, (C), after irradiation at 254 nm for 48 h, and (D) after irradiation at 254 nm for 72 h (D). All spectra were recorded in $CDCl_3$. Benzene was used as an internal standard in all cases at a concentration of 8 mM.

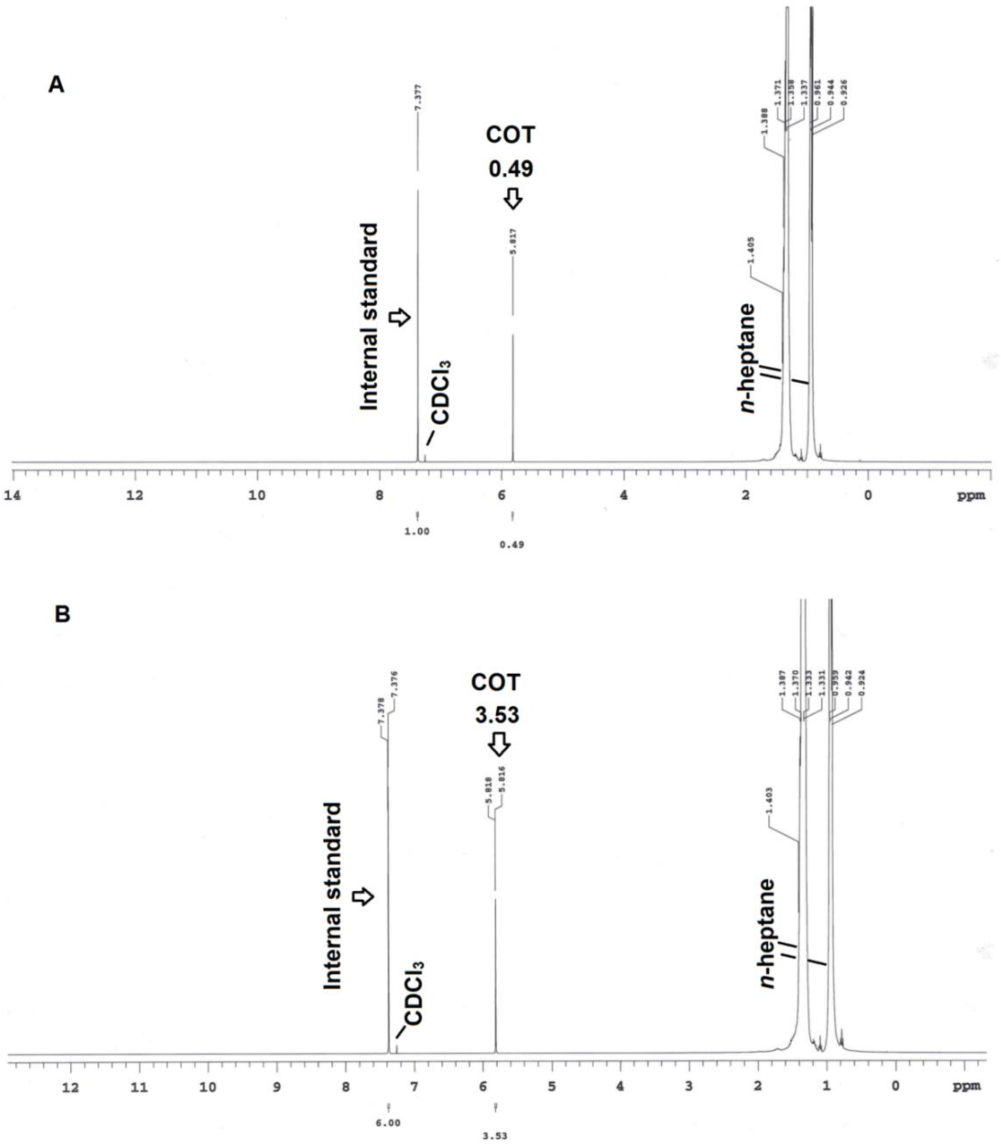

Supplementary Figure 57. ^{1}H-NMR spectrum of (A) a solution of COT (63 mM) in *n*-heptane after 72 h irradiation at 254 nm, and (B) without irradiation. In both A and B, for the quantification of the amount of COT, benzene was used as an internal standard. Integration of the COT signal shows that 58% of COT was consumed.

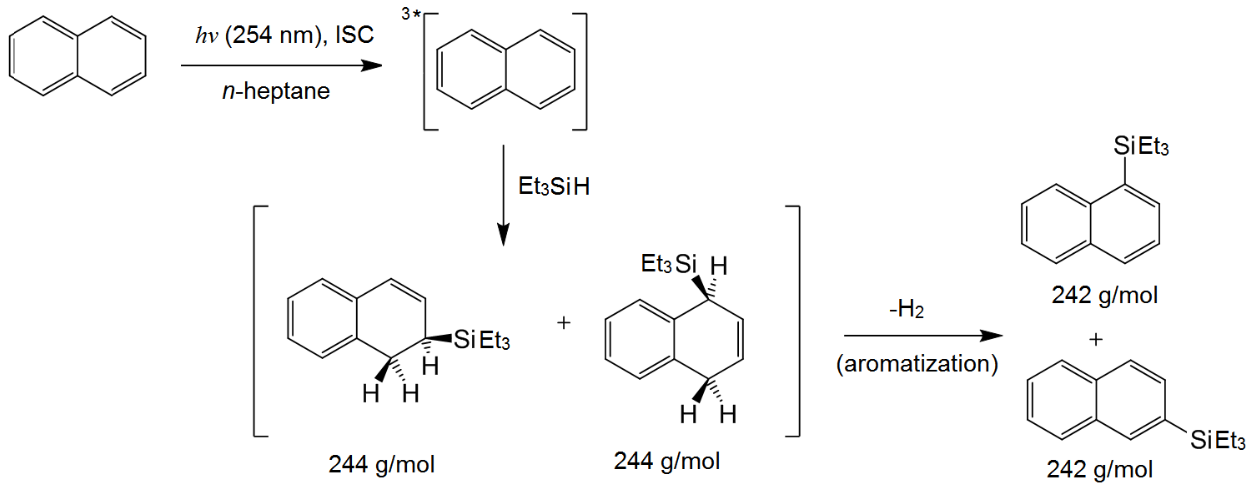

Supplementary Figure 58. Photo(hydro)silylation of naphthalene in *n*-heptane in excess of Et_3SiH (254 nm) and dehydrogenation/aromatization to yield a 1/1 mixture of α- and β-naphthyltriethylsilane.

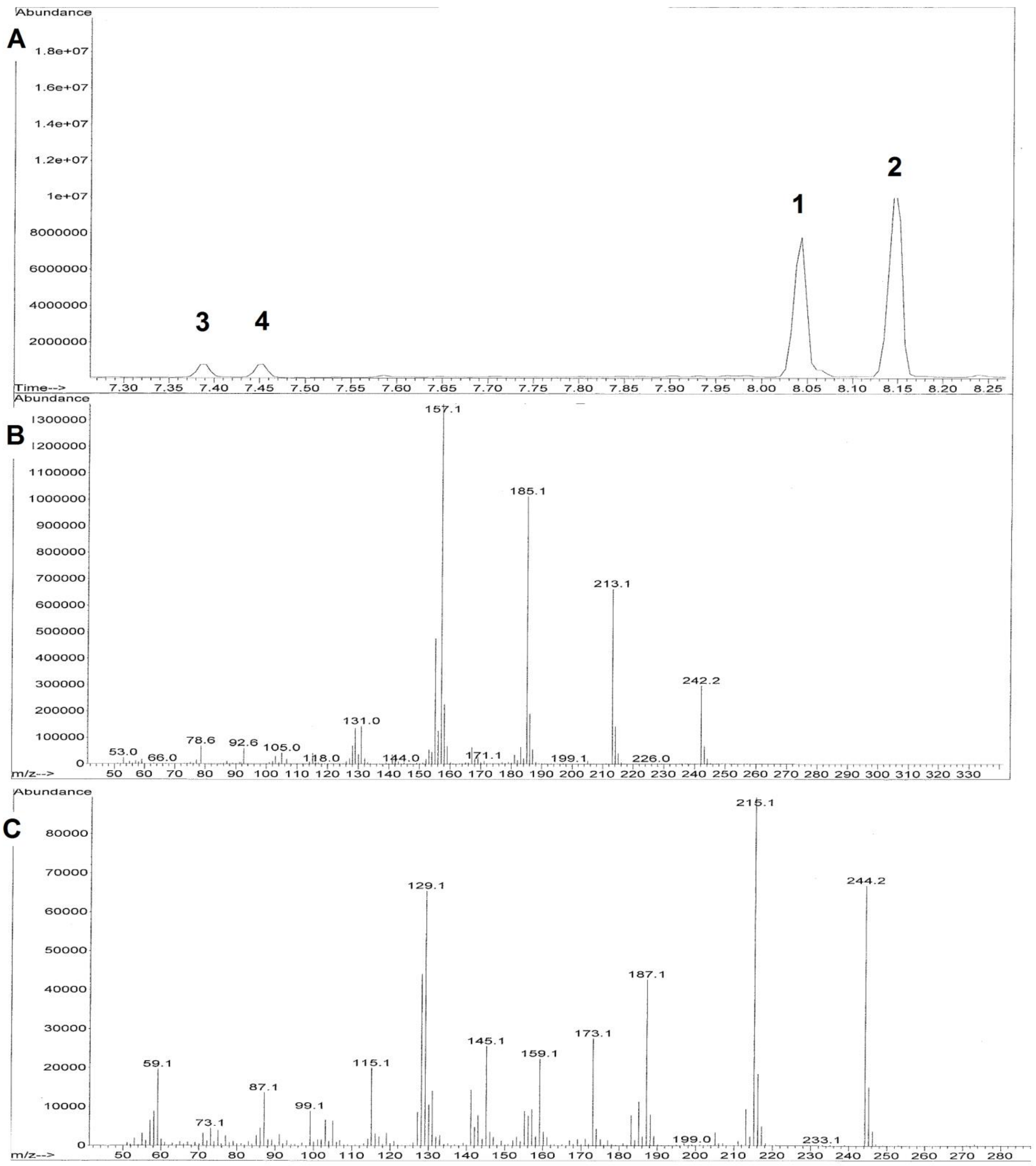

Supplementary Figure 59. (A) Partial GC of the mixture of the crude product obtained after photo(hydro)silylation of naphthalene using a 50-fold excess of Et_3SiH in a home-made FEP-photoreactor. Products 1 and 2 correspond to α- and β-naphthyltriethylsilane whereas products 3 and 4 correspond to two isomeric hydroslilylated naphthalenes. (B) The MS of the products 1 and 2 (both show molecular ions with m/z = 242.2). (C) The MS of the products 3 and 4 (both show molecular ions with m/z = 244.2).

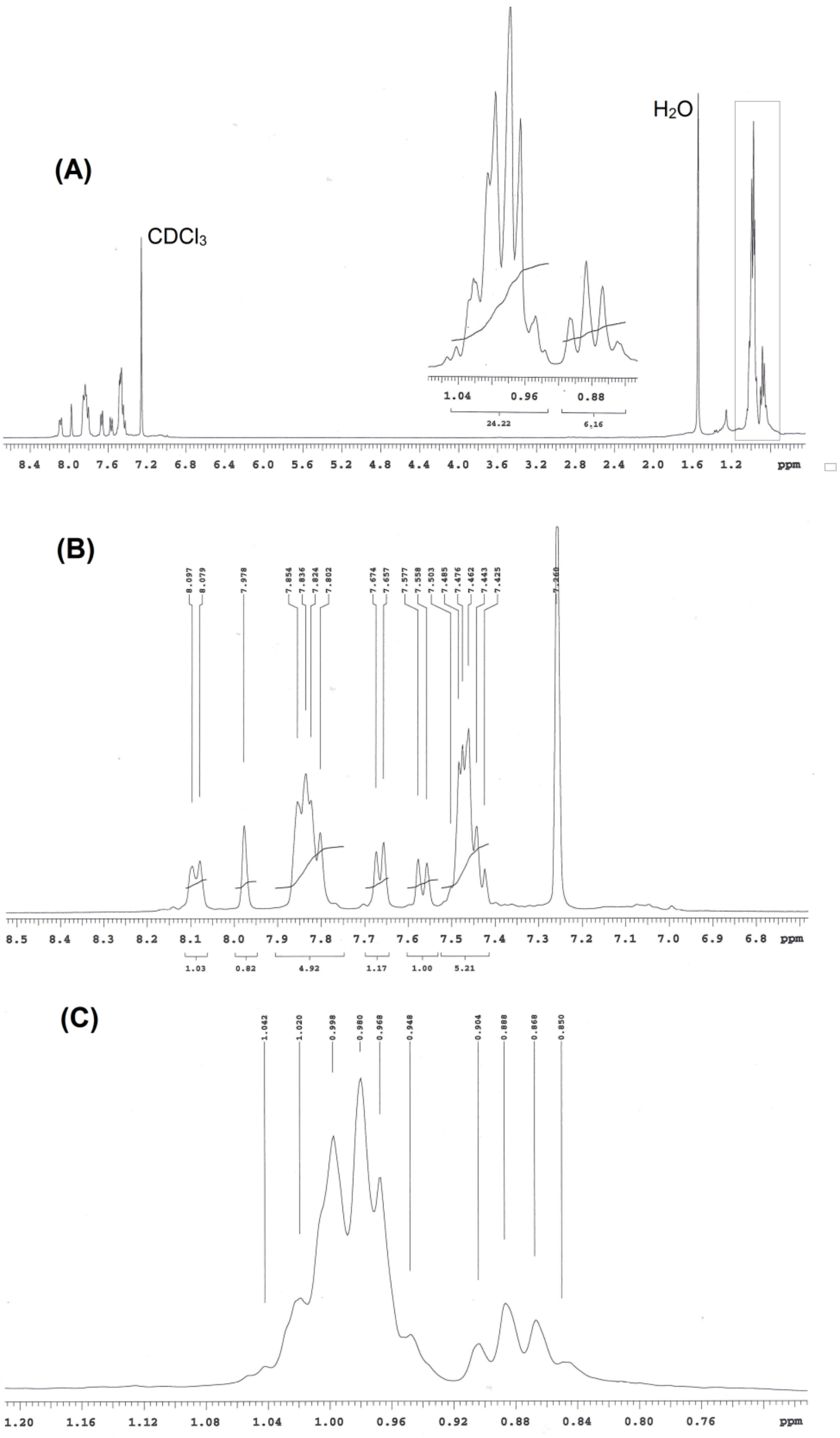

Supplementary Figure 60. (A) ^{1}H-NMR spectrum of the product of naphthalene silylation (Relaxation Delay: 10.00 sec; Pulse: 45.0 degrees; 64 repetitions). Inset magnification and integration of the aliphatic ^{1}H signals. Partial ^{1}H-NMR spectrum of the product of naphthalene silylation (B): aromatic signals, (C): aliphatic signals.

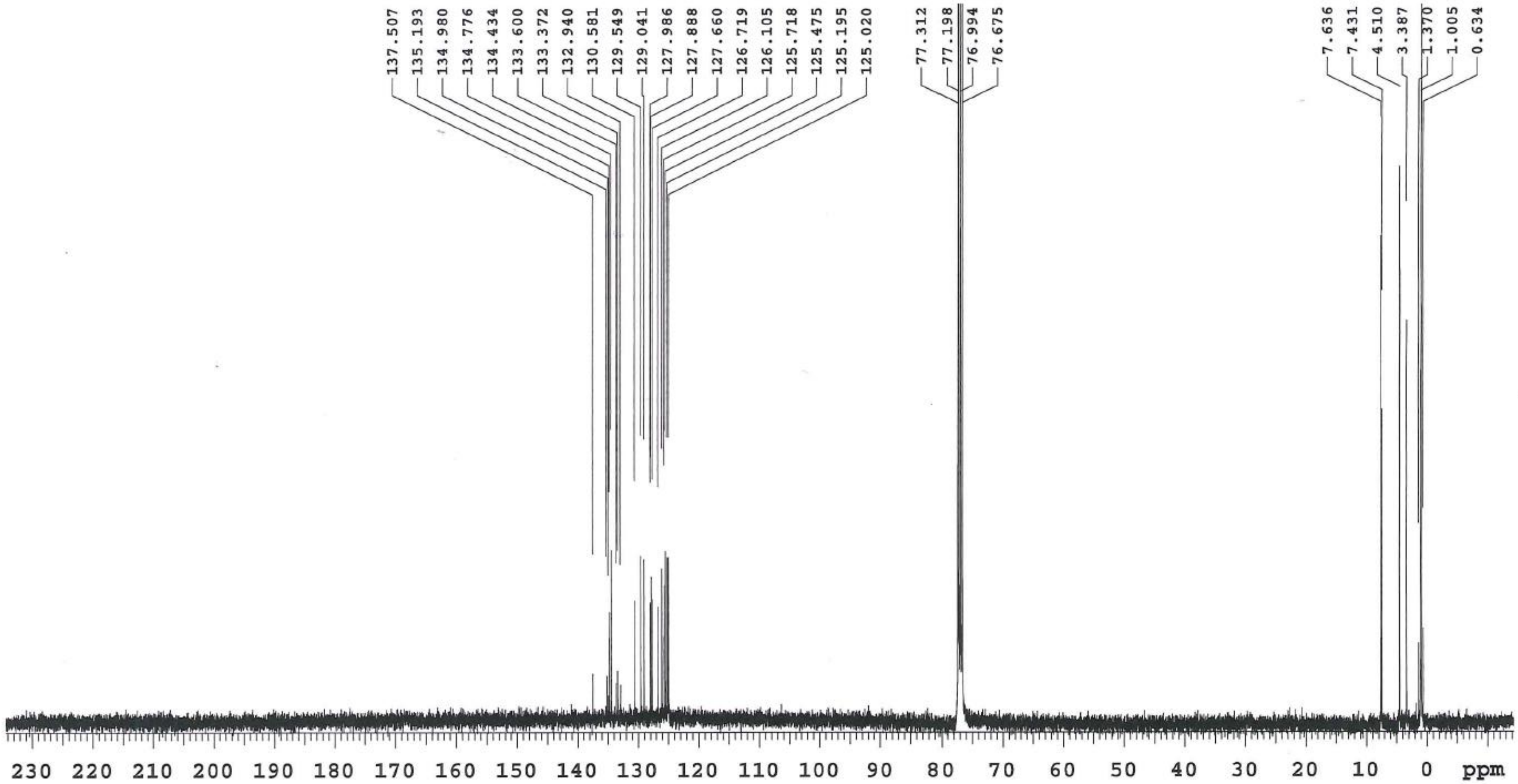

Supplementary Figure 61. ^{13}C-NMR spectrum of the isolated mixture of α- and β-naphthyltriethylsilane, measured in $CDCl_3$. (Relaxation Delay: 0.45 sec; Pulse: 45.0 degrees; 25000 repetitions).

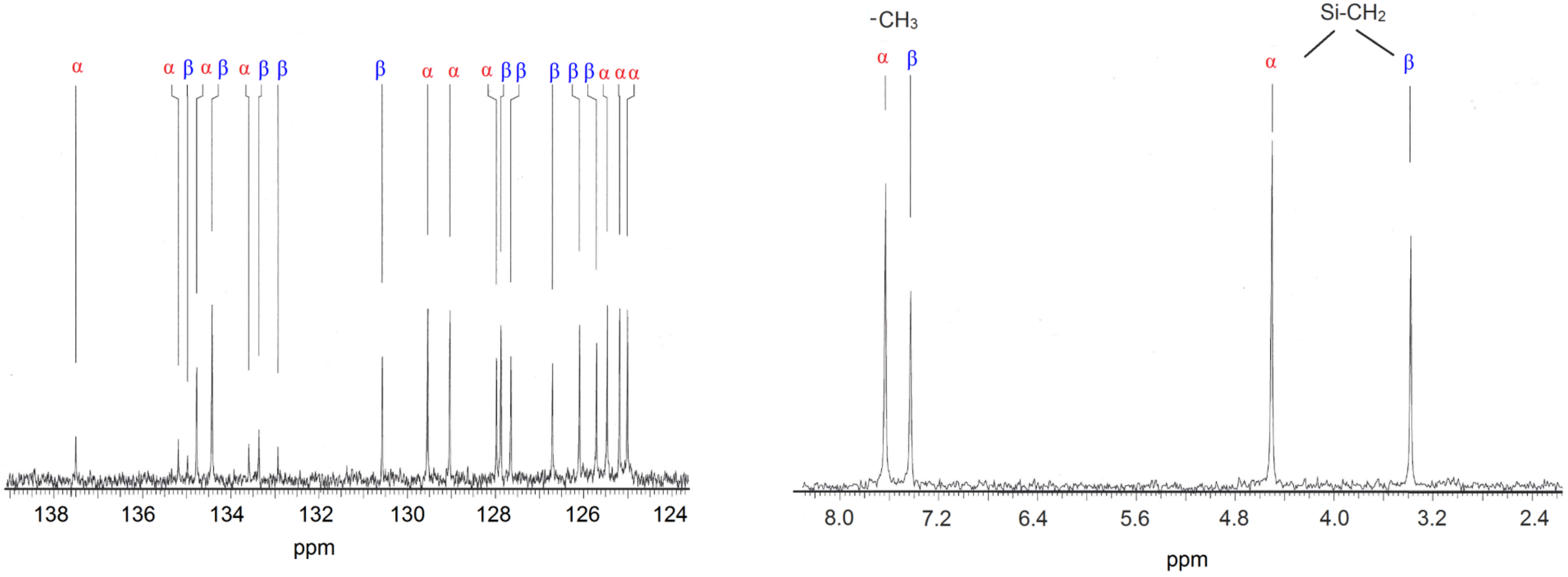

Supplementary Figure 62. Partial ^{13}C-NMR spectrum of the isolated mixture of α- and β-naphthyltriethylsilane, recorded in $CDCl_3$ including assignment of each peak (α: α-naphthyltriethylsilane; β: β-naphthyltriethylsilane).

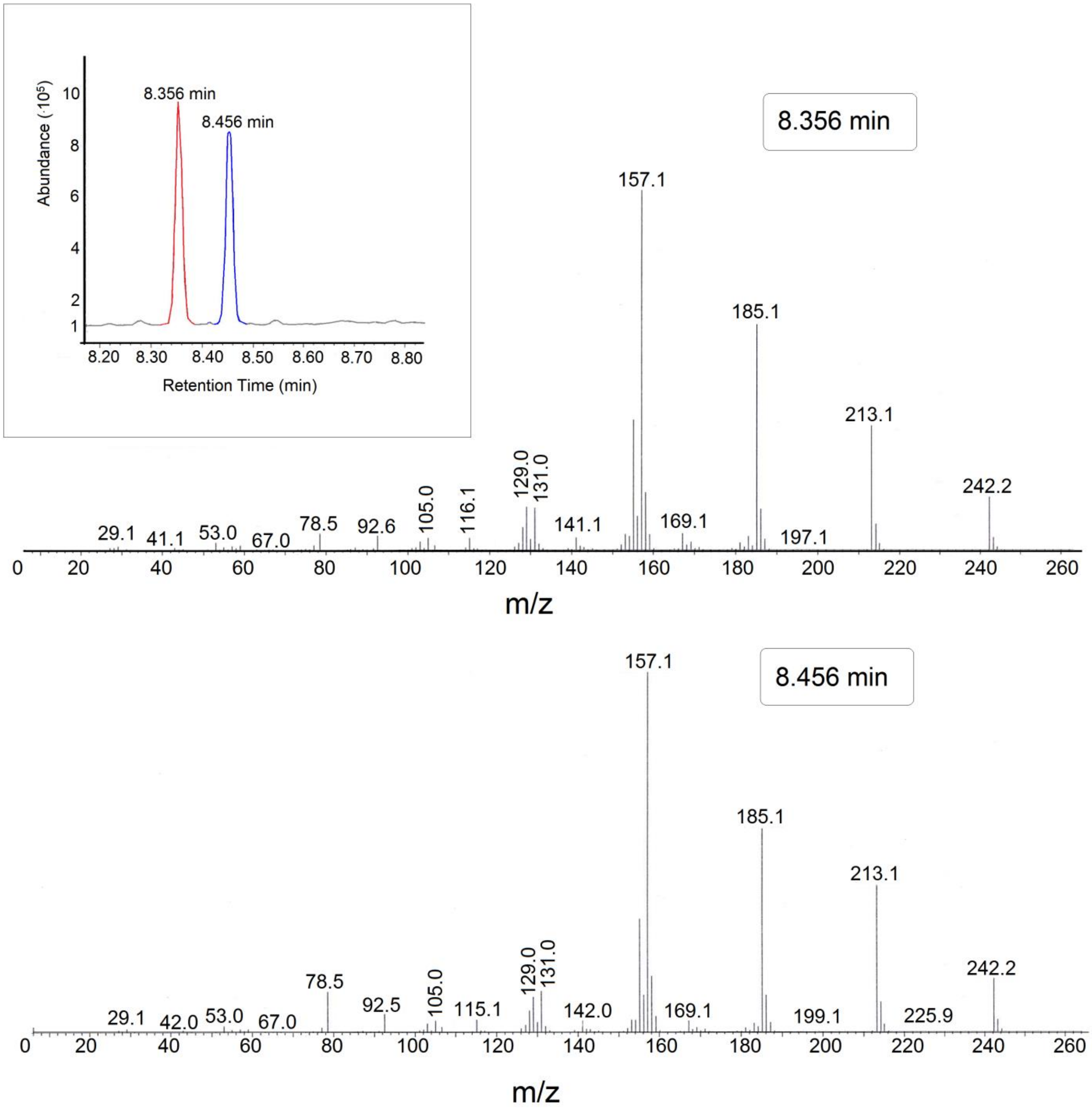

Supplementary Figure 63. Partial GC of the mixture of α- and β-naphthyltriethylsilane obtained after photoreaction of naphthalene with triethylhydrosilane (inset). The MS of the two products (both show molecular ions with m/z = 242.2).

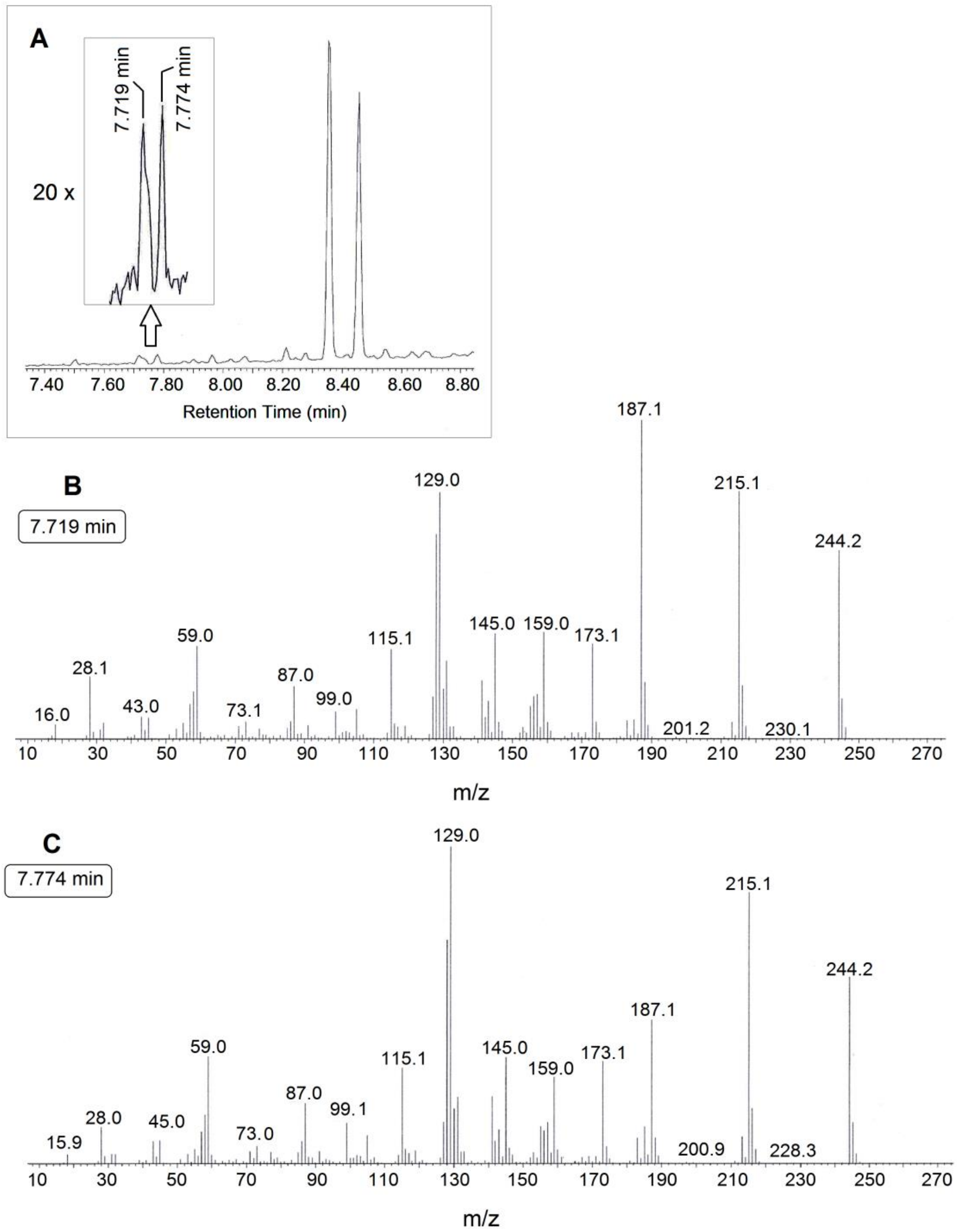

Supplementary Figure 64. (A) Partial GC of the mixture obtained after photoreaction of naphthalene with triethylhydrosilane in *n*-heptane, showing the traces of two possible hydrosilylated naphthalene products (magnification in inset: 20x). (B) and (C) mass spectra of the products with retention times: 7.719 and 7.774 min respectively (both products show molecular ions with m/z: 244.2 Da).

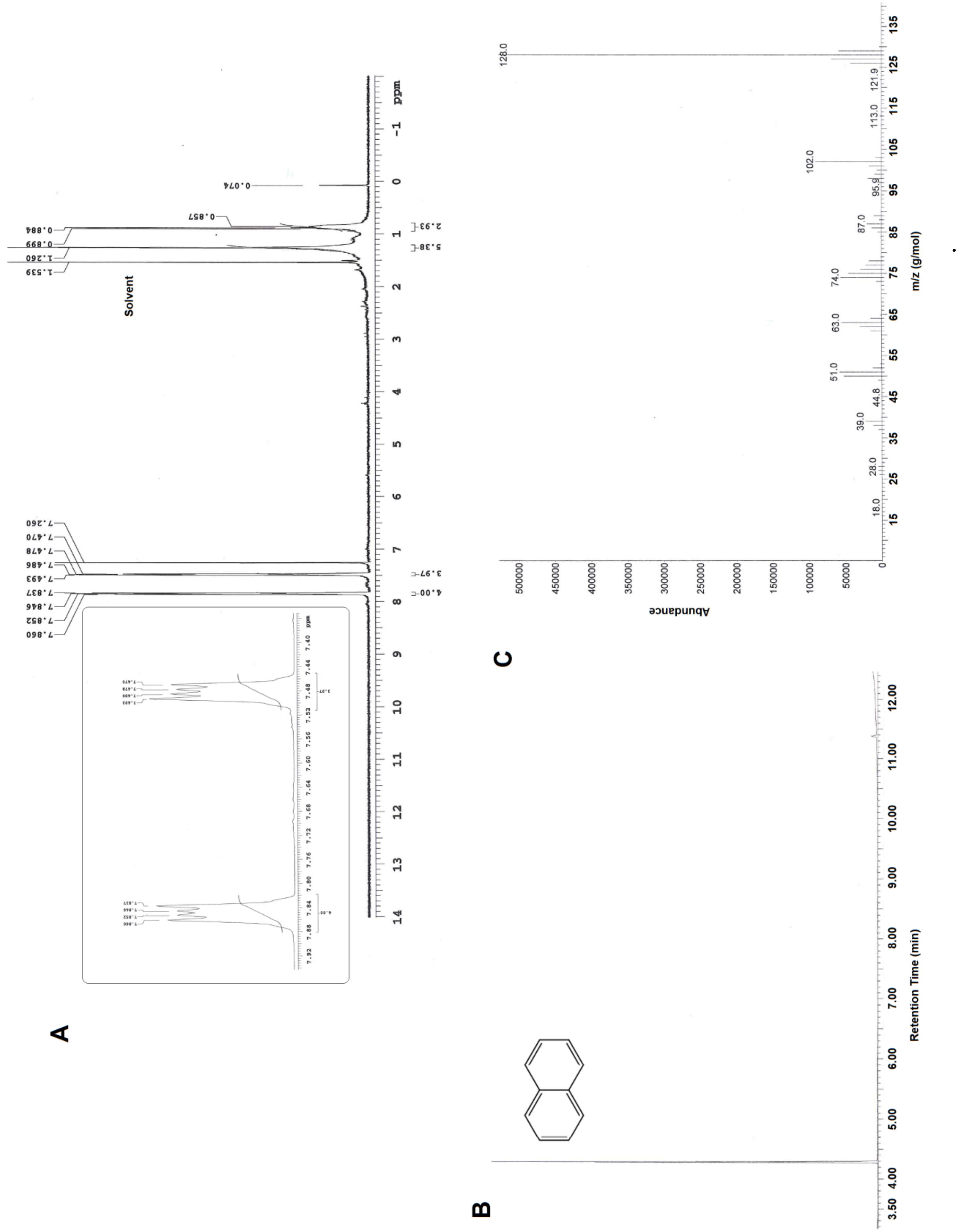

Supplementary Figure 65. ^{1}H NMR spectrum after control experiment: naphthalene in *n*-heptane (2.92 mM) with Et_3SiH (30.3 mM) in the dark for 48 h. (B) GC after the same control experiment showing only one signal (naphthalene) and (C) corresponding MS (of naphthalene). Both ^{1}H NMR spectroscopy and GC-MS indicated: no reaction in the dark.

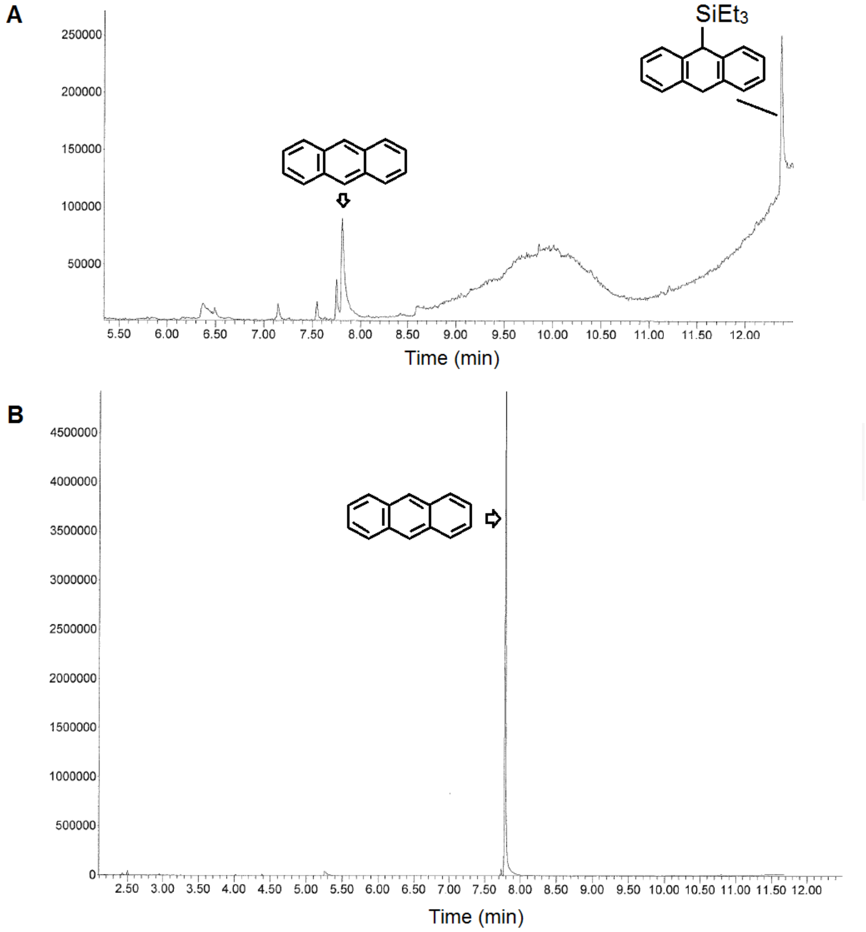

Supplementary Figure 66. (A) GC after irradiation of a 5 mM benzene solution of anthracene containing a 125-fold excess of Et_3SiH at λ= 365 nm in borosilicate glass under strictly unaerated conditions for 24 h. (B) GC of the starting anthracene/Et_3SiH benzene solution.

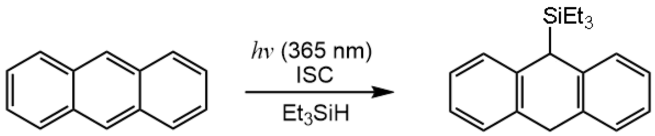

Supplementary Figure 67. Photoreaction of anthracene in benzene in excess of Et_3SiH in borosilicate glass at λ=365 nm.

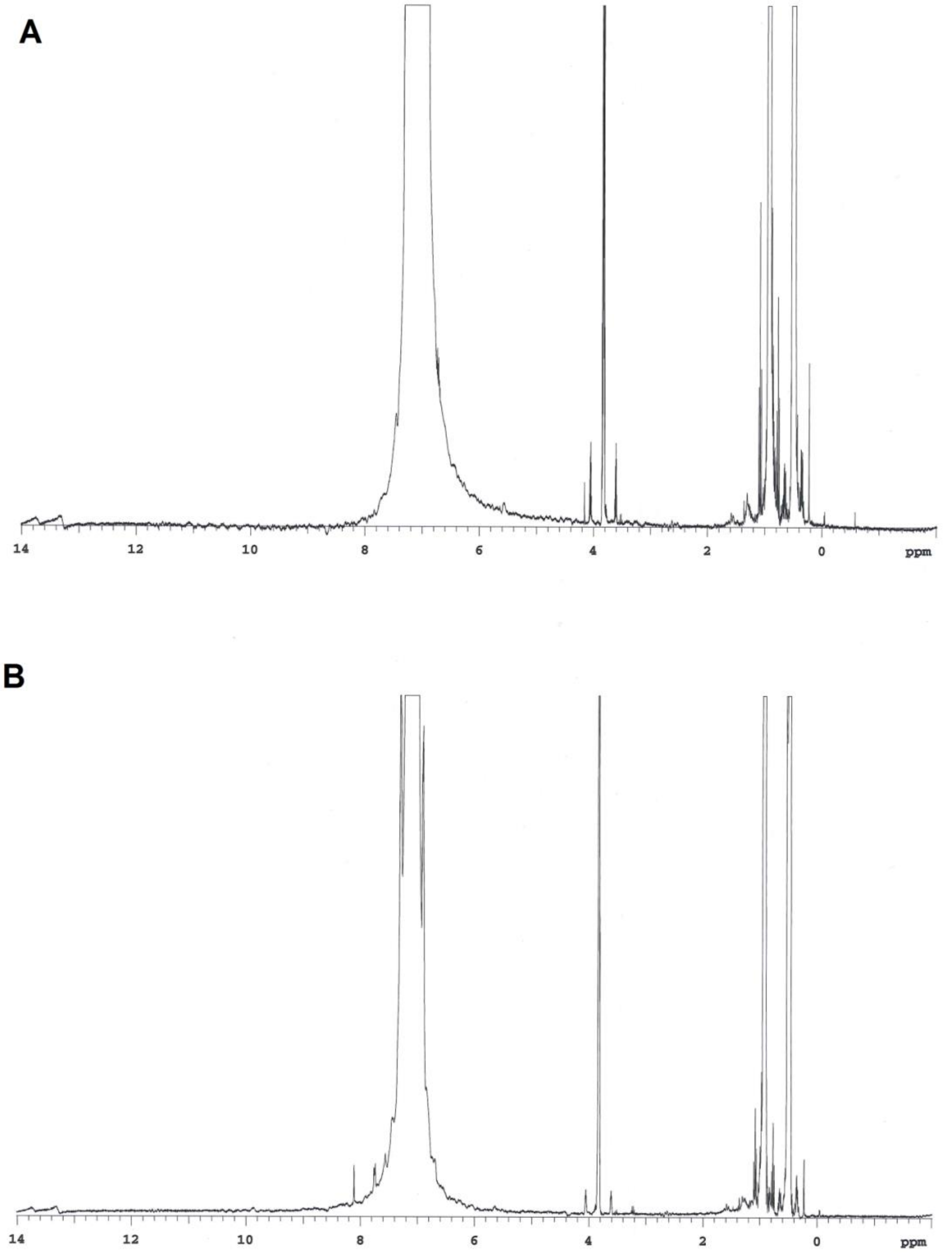

Supplementary Figure 68. (A) Full ^{1}H-NMR obtained after irradiation of a 5 mM benzene solution of anthracene containing a 125-fold excess of Et_3SiH at λ= 365 nm in borosilicate glass under strictly unaerated conditions for 24 h. (B) Full ^{1}H-NMR spectrum of anthracene/Et_3SiH benzene solution. In both cases 20% of C_6D_6 was added.

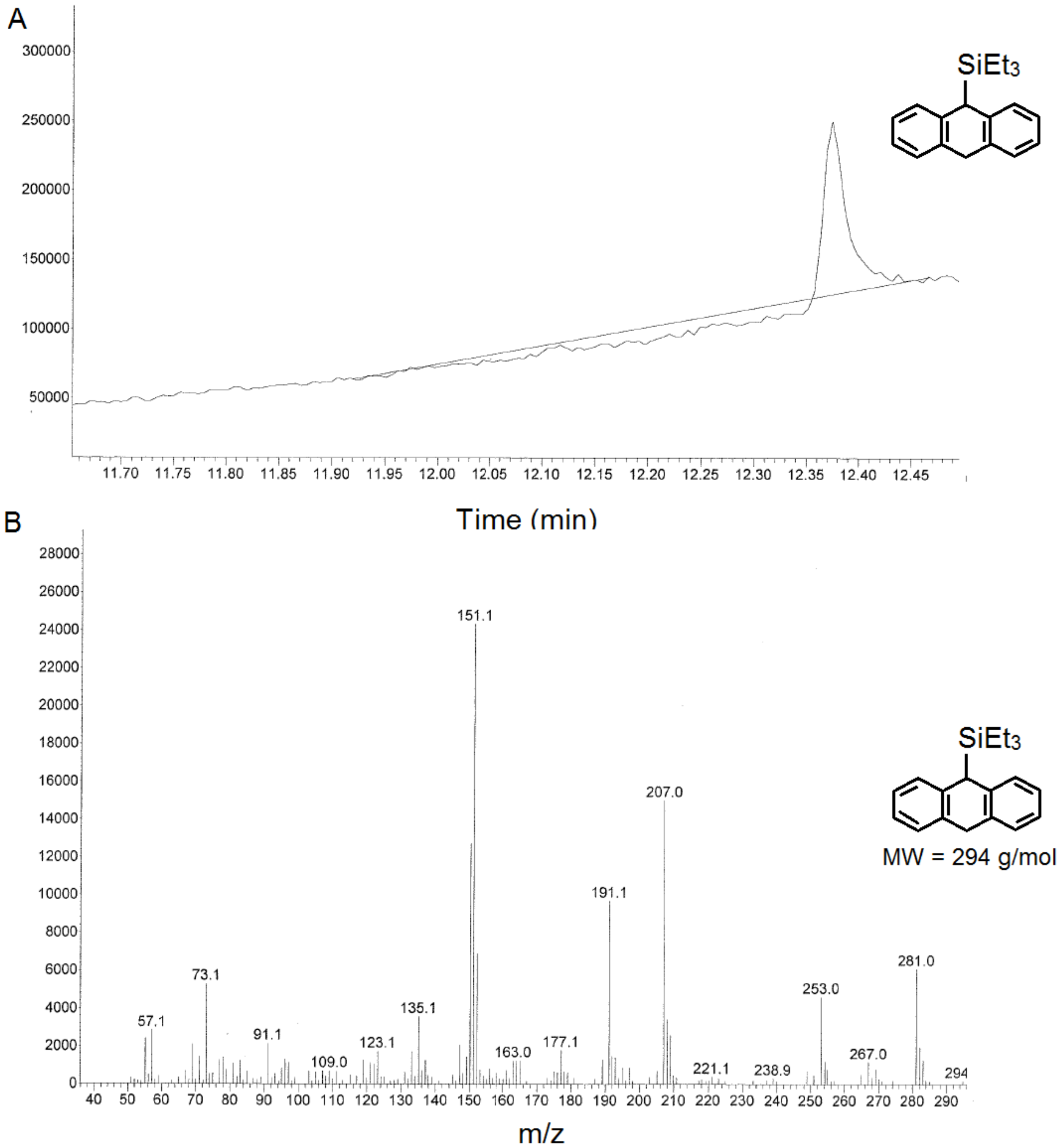

Supplementary Figure 69. (A) Partial GC after 24 h irradiation of a 5mM benzene solution of anthracene in excess of Et_3SiH (125-fold excess) in borosilicate glass at λ=365 nm under strictly anaerated conditions. (B) The MS of the peak shown in A corresponding to 9-(triethylsilyl)-9,10-dihydroanthracene.

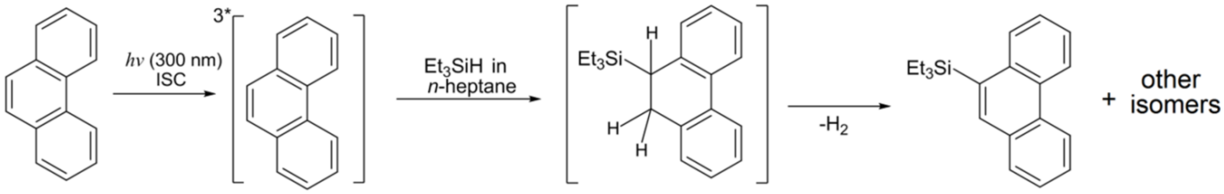

Supplementary Figure 70. Photo(hydro)silylation of phenanthrene.

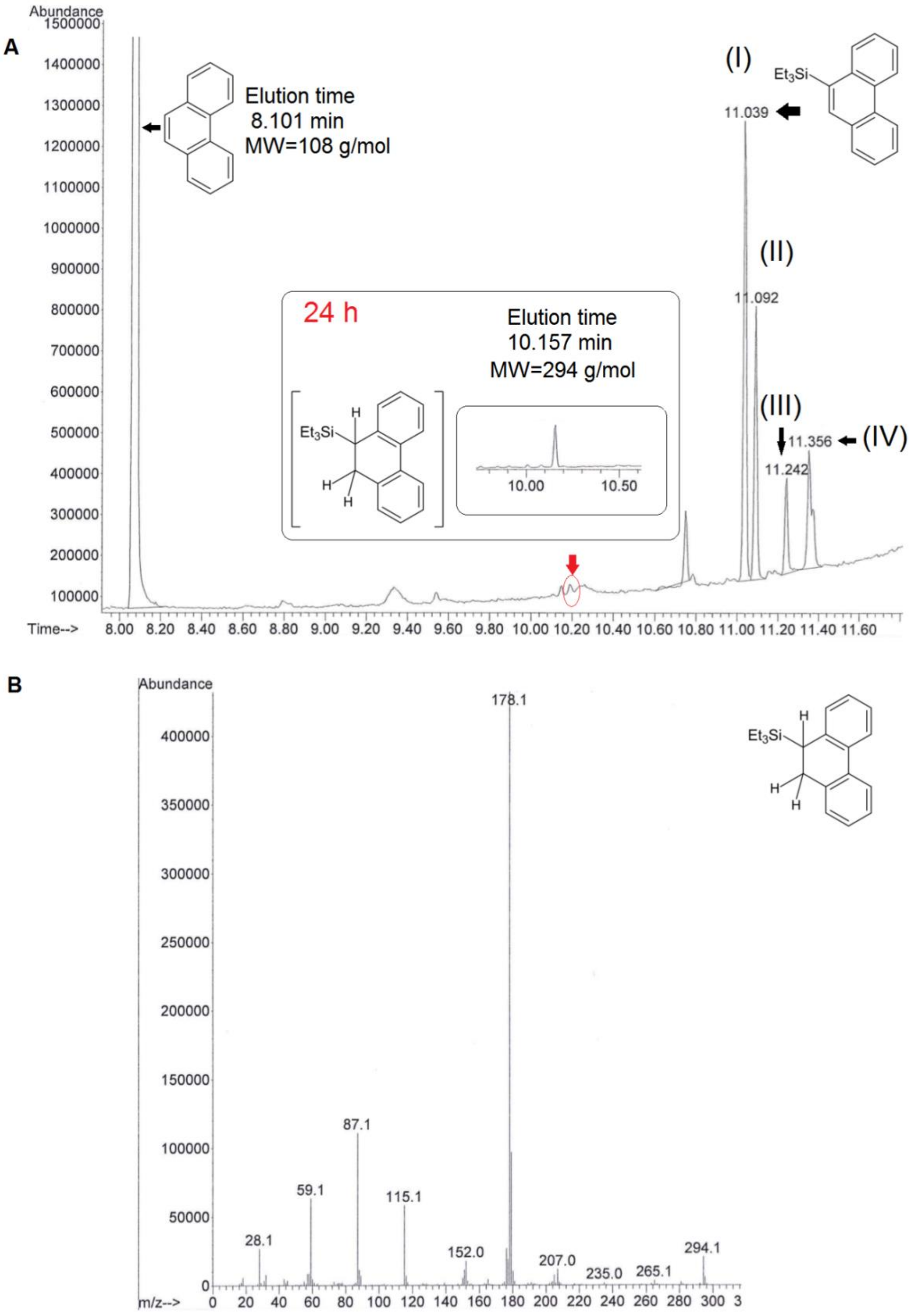

Supplementary Figure 71. (A) GC after hydrosilylation of phenanthrene in *n*-heptane. Traces of a monohydrosilylated phenanthrene (retention time: 10.157 min) as well as products I to IV, all giving molecular ions with m/z = 294 g/mol. (B) MS of hydrosilylated product with retention time 10.157 min.

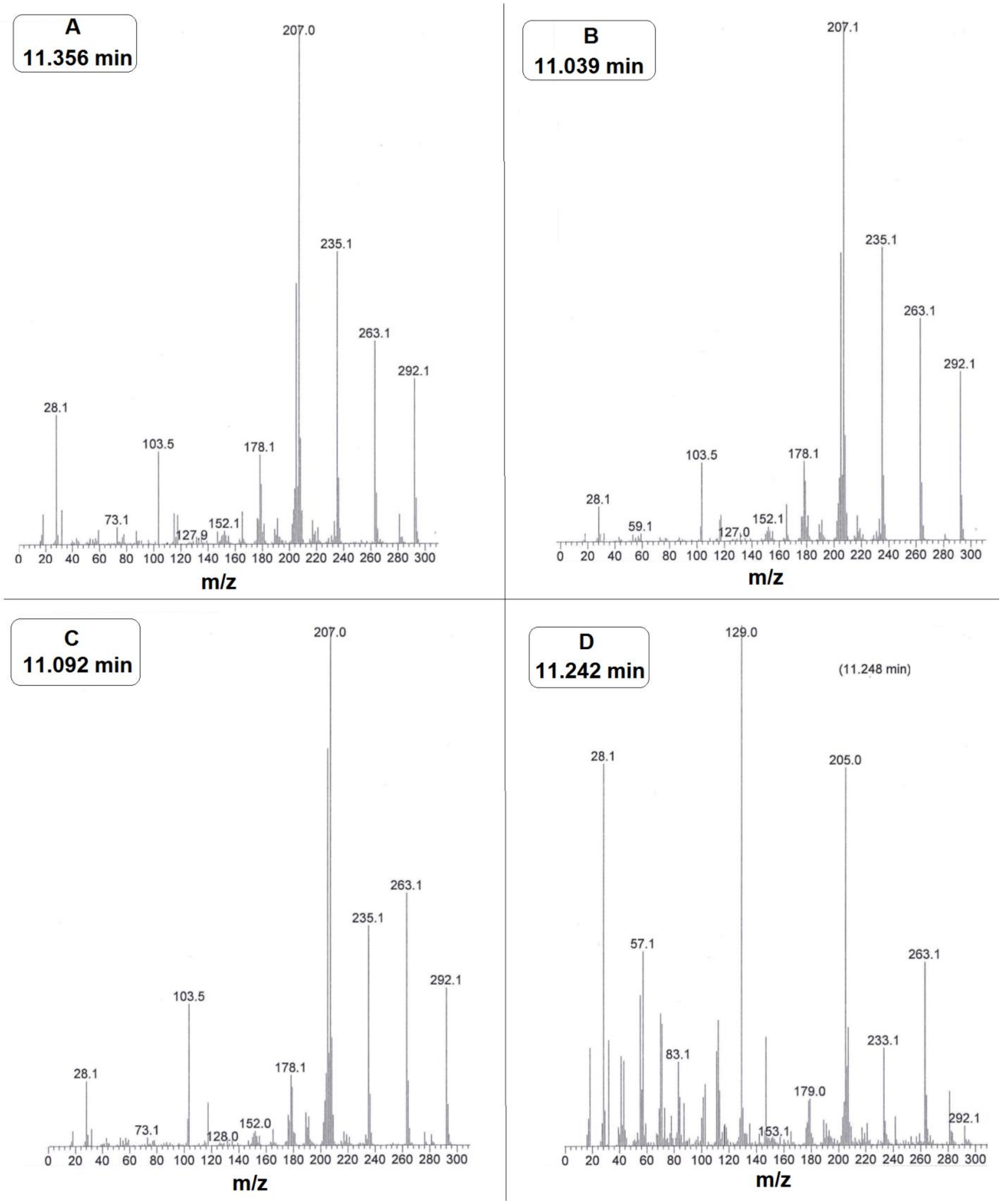

Supplementary Figure 72. MS of isomeric monsilylated phenanthrenes (MW = 292 g/mol) produced after photoreaction of phenanthrene in *n*-heptane in excess of Et_3SiH. Retention times (GC) are mentioned in all cases.

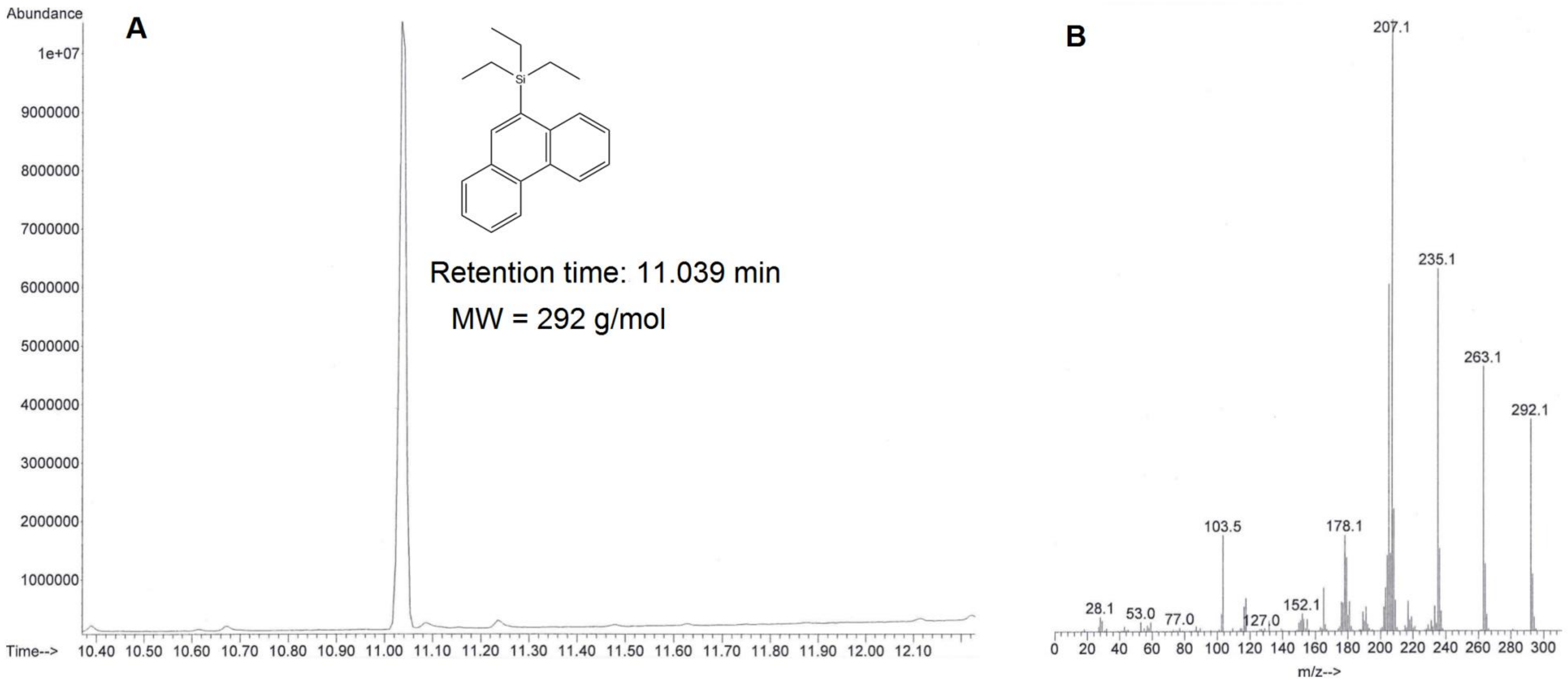

Supplementary Figure 73. (A) GC of 9-triethylsilylphenanthrene synthesized according to literature[20] (B) MS of synthesized 9-triethyl-silylphenanthrene.

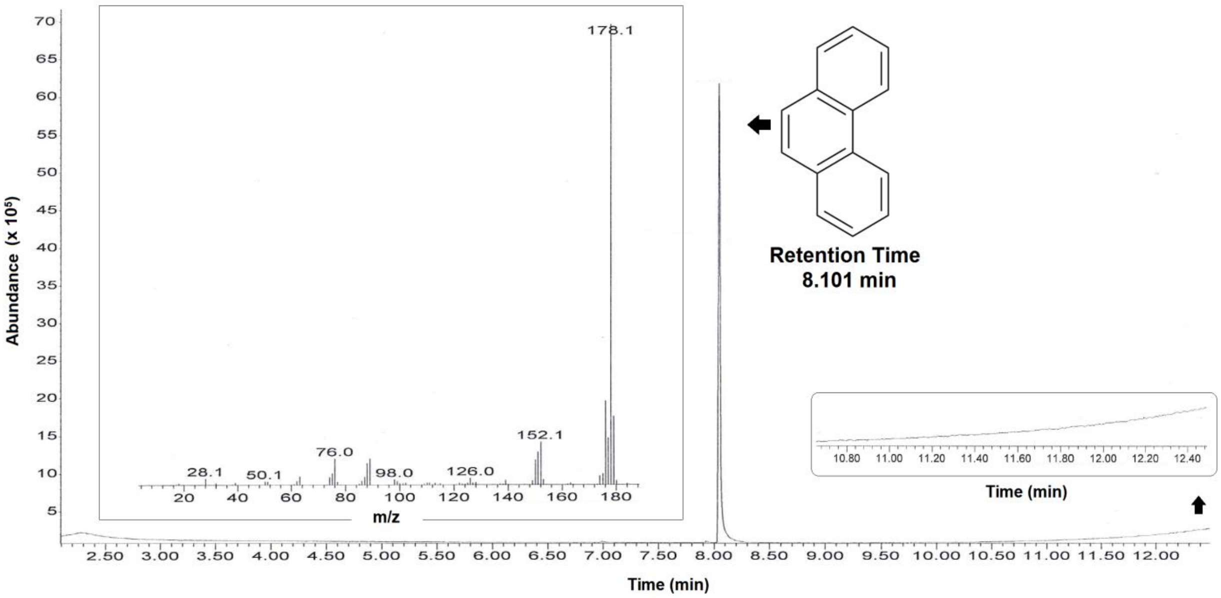

Supplementary Figure 74. GC after control experiment: phenanthrene and Et_3SiH in *n*-heptane in the dark for 48 h. Only phenanthrene was identified. Inset shows the MS of phenanthrene (retention time 8.101 min). No product identified with retention time higher than 8.105 min (no silylated or hydrosilylated products).

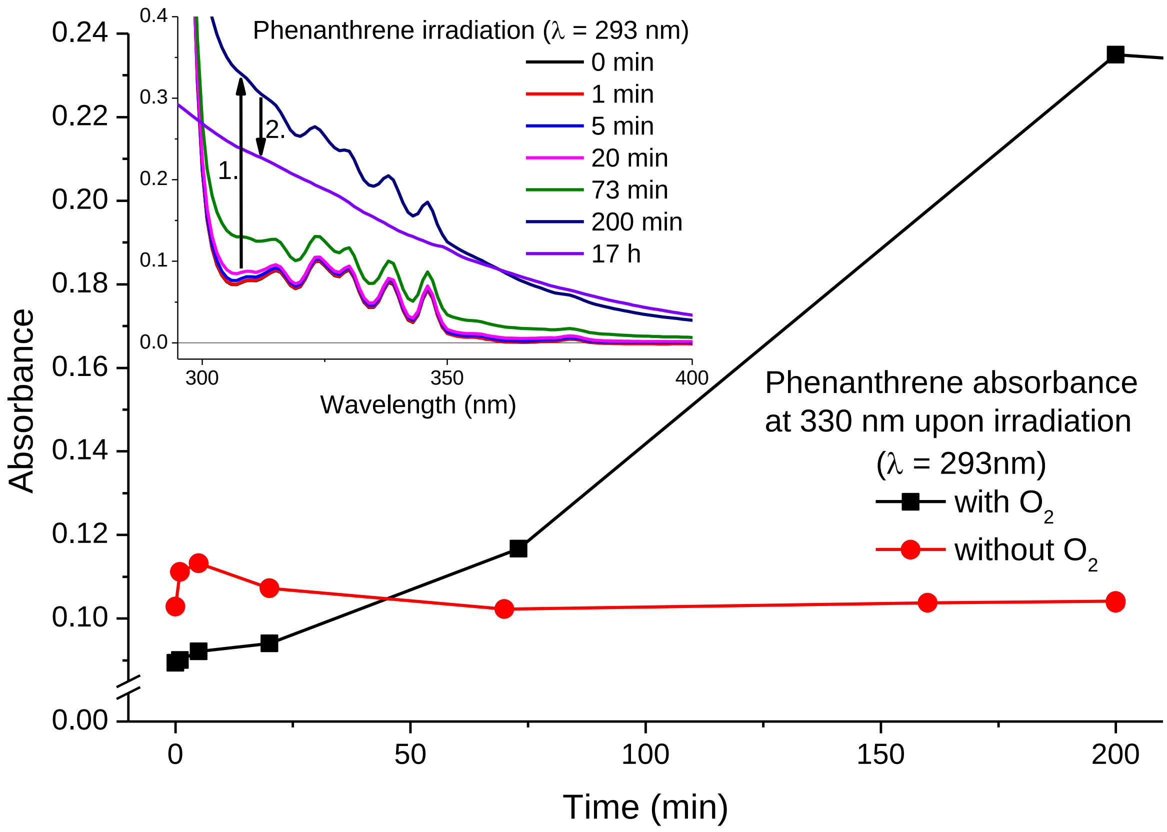

Supplementary Figure 75. Changes in phenanthrene absorbance at 330 nm *vs*. irradiation time (λ_{ex} = 293 nm) in the presence and absence of O_2. The inset shows the changes in the absorbance spectra over time.

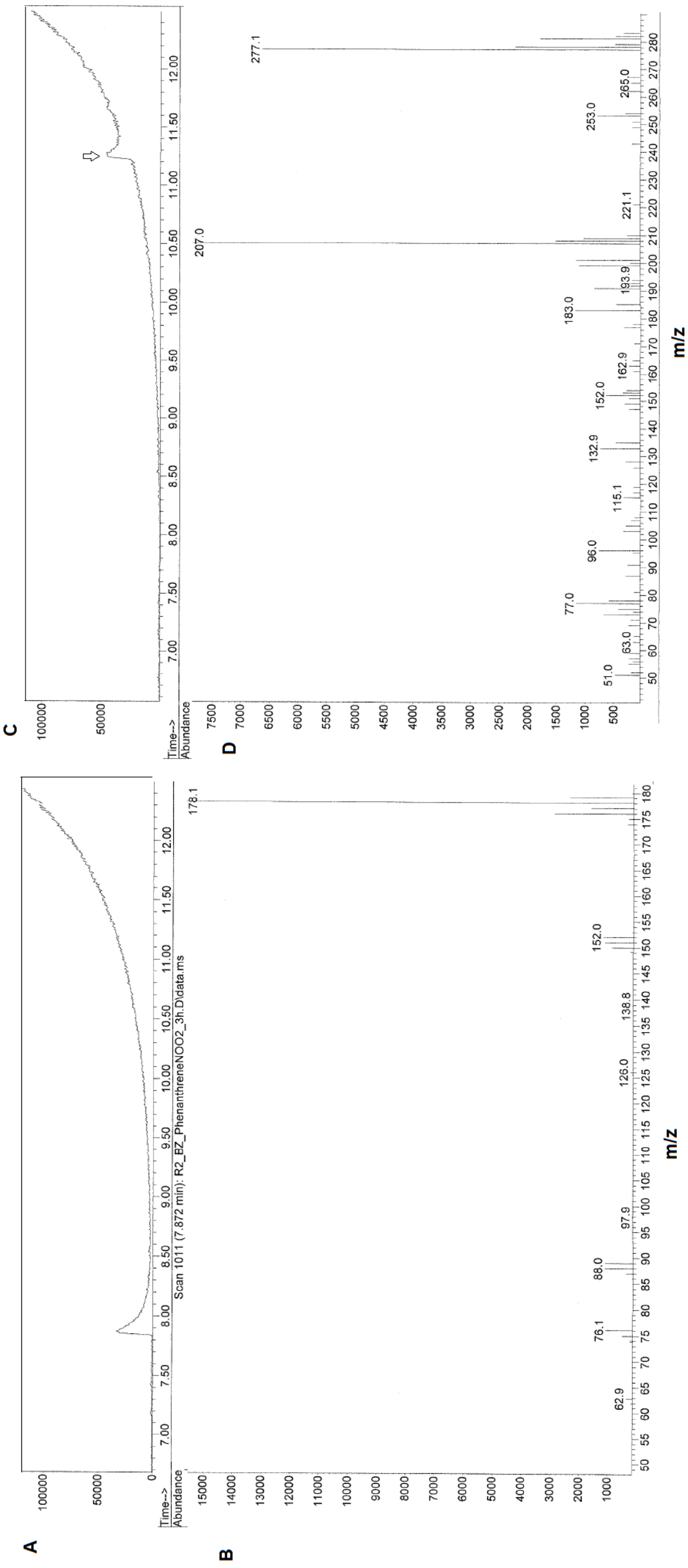

Supplementary Figure 76. (A) GC of the starting phenanthrene solution containing excess of Et_3SiH. (B) MS of phenanthrene. (C) GC after irradiation at λ=293 nm of phenanthrene in *n*-heptane in excess of Et_3SiH. (D) MS of the observed new peak in the GC of (B). A product which does not correspond to a (hydro)silylated phenanthrene.

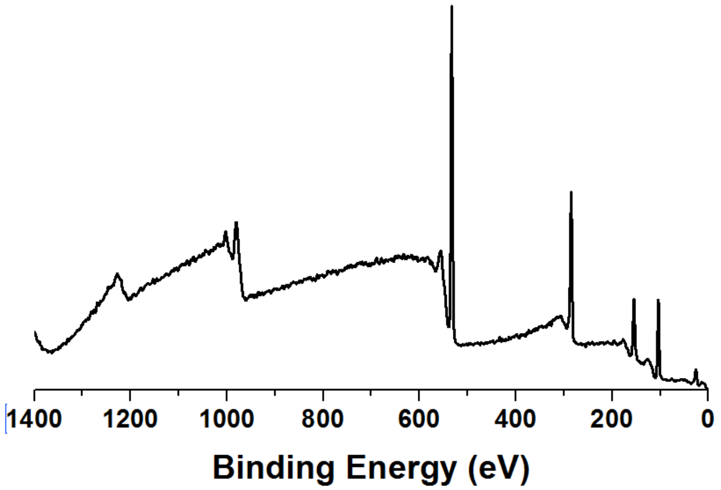

Supplementary Figure 77. Survey XPS of a pristine CVD graphene sample

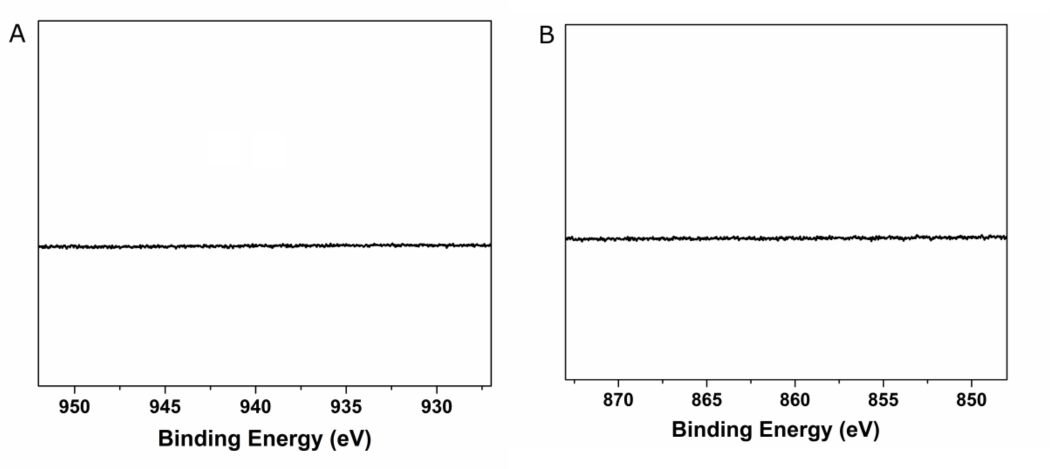

Supplementary Figure 78. High resolution XPS of pristine CVD graphene (A) of Cu2p and (B) of Ni2p.

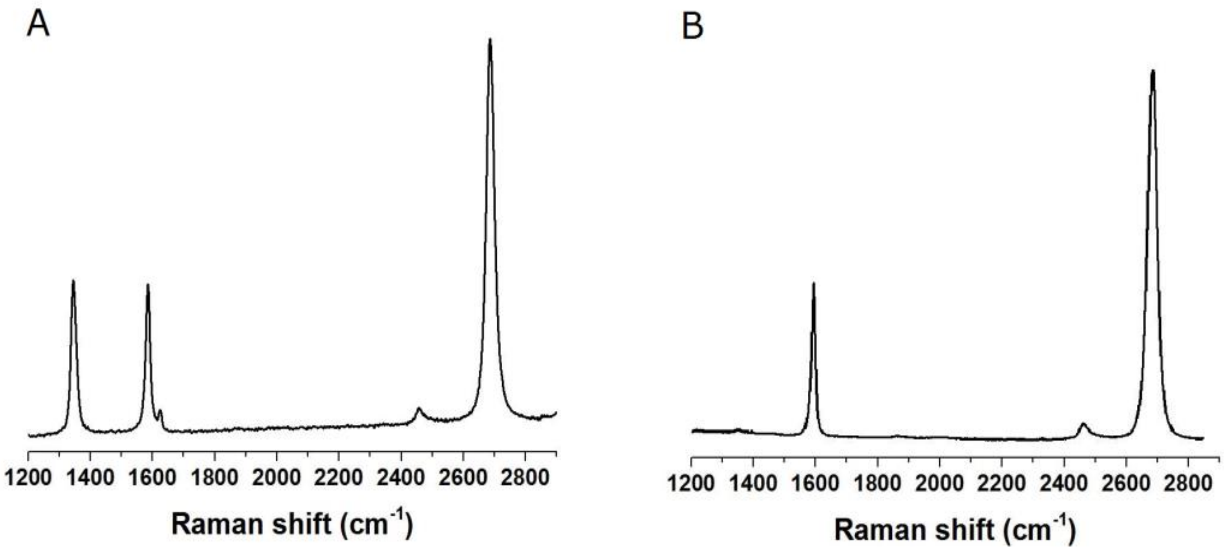

Supplementary Figure 79. Raman scattering spectrum of (A) defective graphene and (B) pristine graphene.

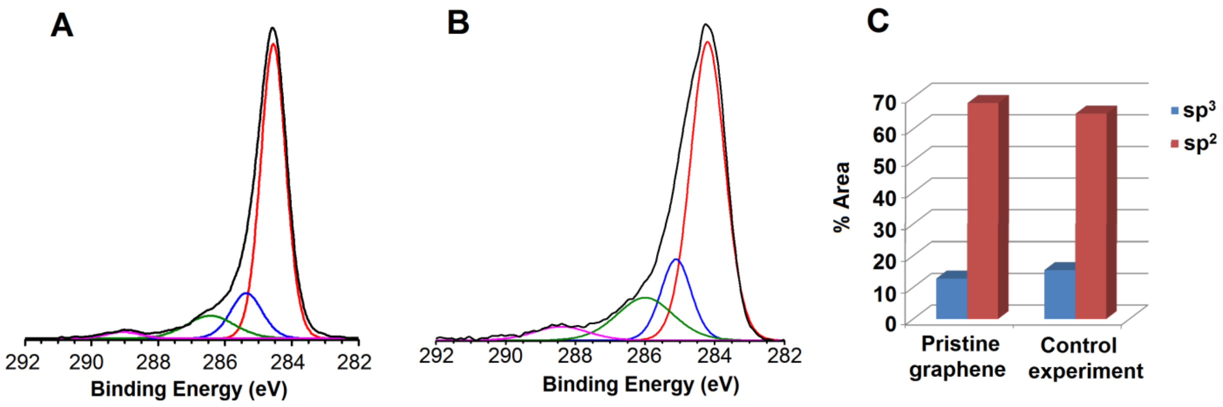

Supplementary Figure 80. High Resolution C1s XPS for (A) pristine graphene and (B) graphene after control experiment (HCOOH/H_2O: 1/1 (v/v) in the dark for 24 h). (C) Histogram showing the % area of the sp^2 and sp^3 deconvoluted carbon of the XPS of panels A and B.

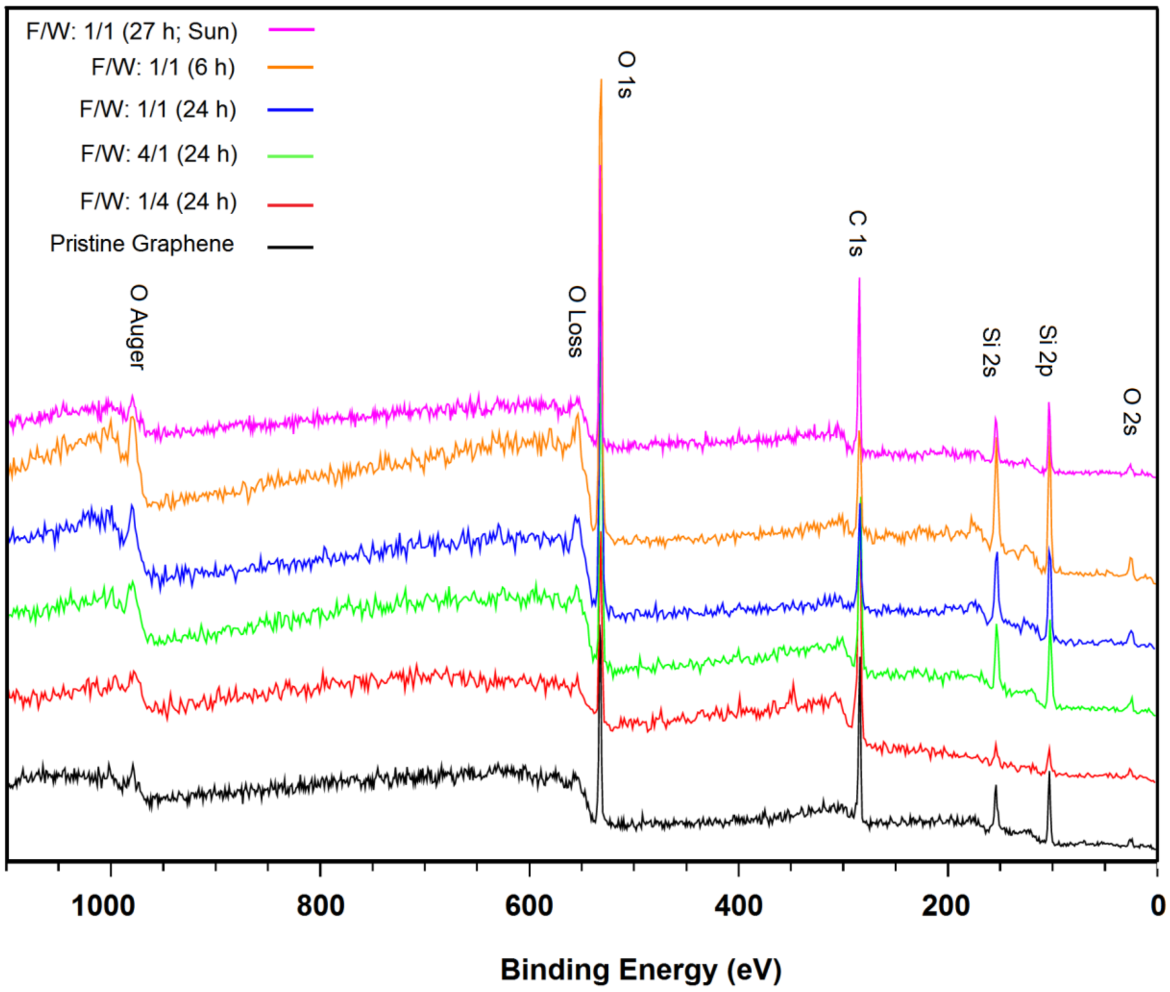

Supplementary Figure 81. Survey XPS for pristine graphene and graphene samples after photohydrogenation *via* different conditions [HCOOH/H_2O: 1/1 (v/v): ~26.6 h Sun-light exposed; 6 h illuminated in WLED photoreactor; 24 h illuminated in WLED photoreactor. HCOOH/H_2O: 4/1 (v/v): 24 h illuminated in WLED photoreactor. HCOOH/H_2O: 4/1 (v/v): 24 h illuminated in WLED photoreactor].

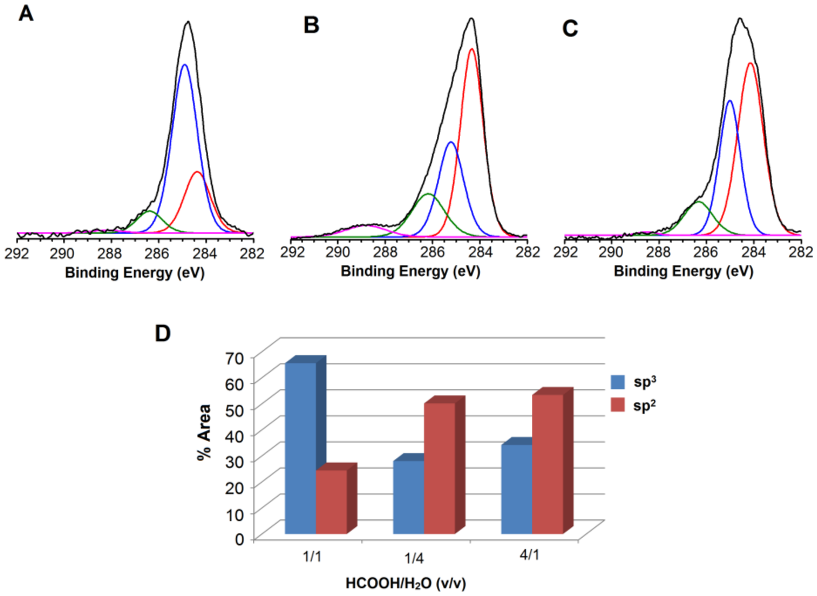

Supplementary Figure 82. High Resolution C1s XPS for graphene after illumination for 24 h in a WLED photoreactor in (A) HCOOH/H_2O: 1/1 (v/v), (B) HCOOH/H_2O: 1/4 (v/v), and (C) HCOOH/H_2O: 4/1 (v/v). Line-colors: C-sp^2: red, C-sp^3: blue, C-O: green, C=O: violet. (D) Histogram showing the % area of the sp^2 and sp^3 deconvoluted carbon signals for the graphene photohydrogenation experiments of panels A-C.

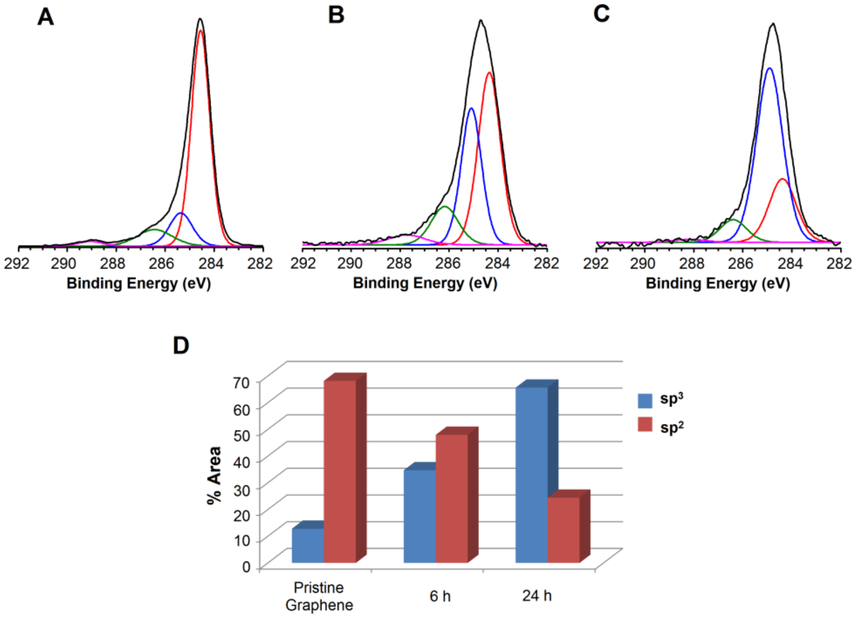

Supplementary Figure 83. High Resolution C1s XPS for pristine graphene (A) and after illumination in a WLED photoreactor in HCOOH/H_2O: 1/1 (v/v) for: 6 h (B) and 24 h (C). Line-colors: C-sp^2: red, C-sp^3: blue, C-O: green, C=O: violet. (D) Histogram showing the % area of the sp^2 and sp^3 deconvoluted carbon signals for pristine graphene and photohydrogenation experiments of panels B and C.

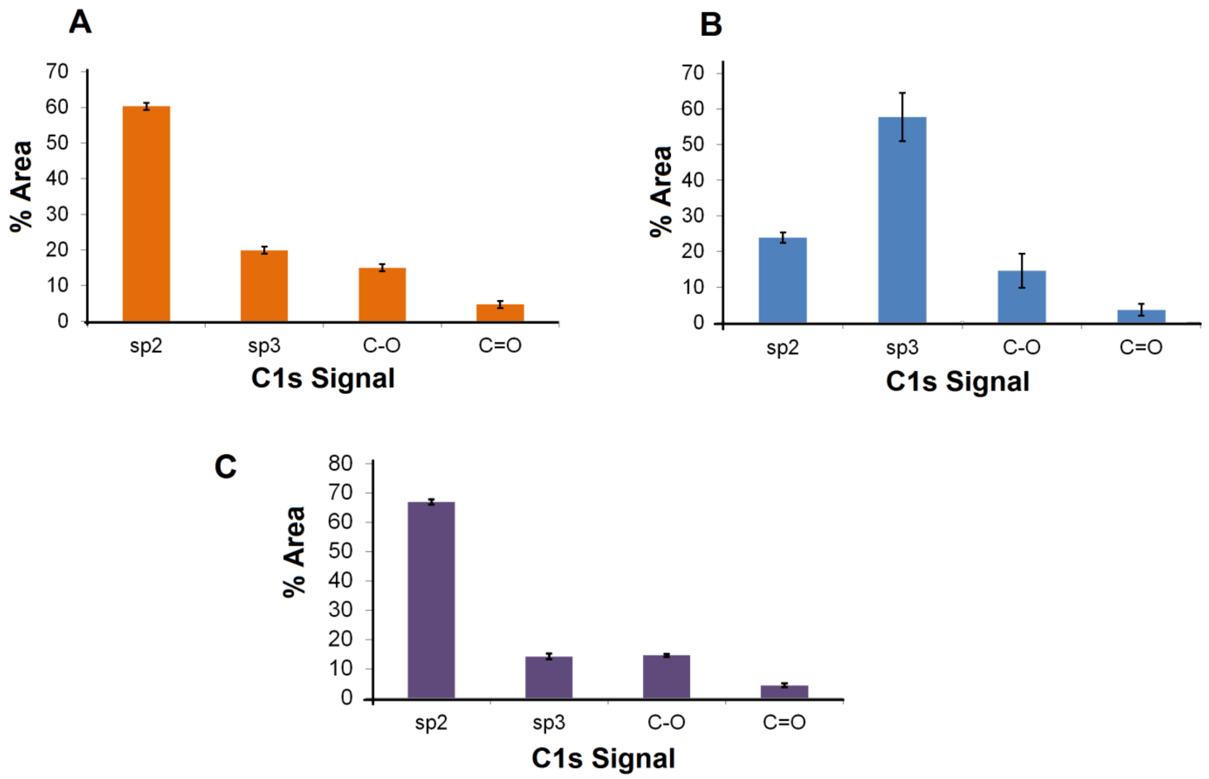

Supplementary Figure 84. Histograms of the % area of the deconvoluted C1s XPS signals for (A) control experiment: graphene in HCOOH/H_2O 1/1(v/v) in the dark for 24 h, (B) after graphene illumination for 24 h (WLED photoreactor) in HCOOH/H_2O 1/1 (v/v) and (C) pristine graphene. Error bars correspond to standard deviation of three replicates.

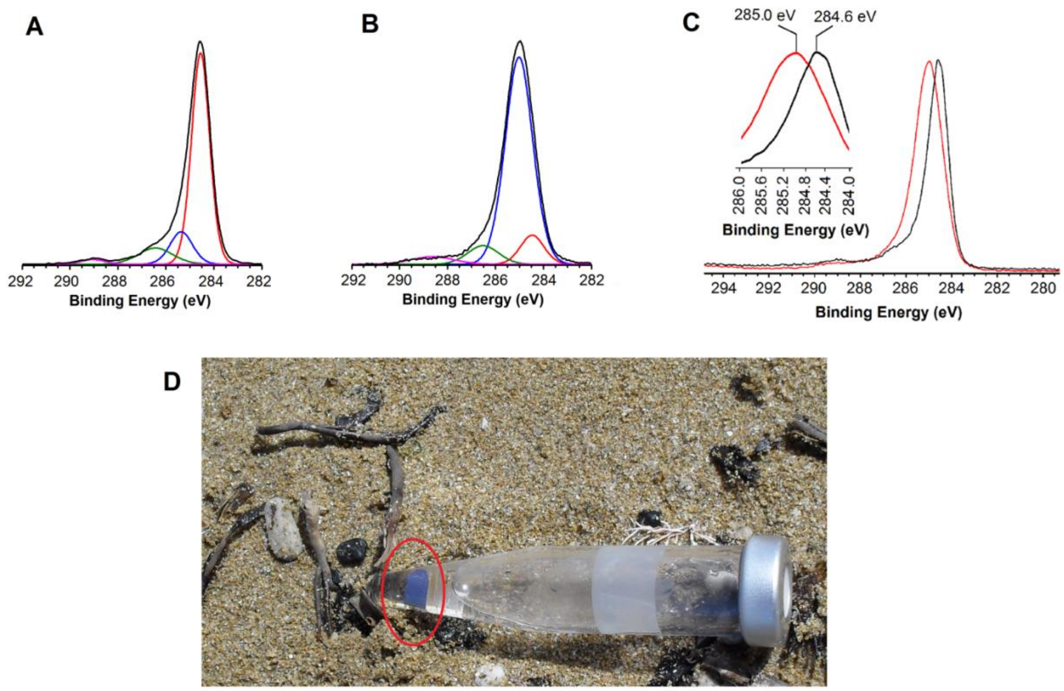

Supplementary Figure 85. (A) High Resolution C1s XPS for pristine graphene. (B) High Resolution C1s XPS of graphene exposed to sun-light when in $HCOOH/H_2O$: 1/1 (v/v) for: 26 h and 38 min. Line-colors in both cases: C-sp^2: red, C-sp^3: blue, C-O: green, C=O: violet. (C) Superimposed high resolution C1s XPS of (A) in black and (B) in red. Inset: Magnification of the XPS, showing where each signal is centered. (D) Photograph depicting the graphene sample in a tube containing $HCOOH/H_2O$: 1/1 (v/v), exposed to sun-light. The vial was sealed and then exposed to sun-light. The experiment was carried out close to Heraklion, Crete, Greece (35.3333° N, 25.1333° E) during the day-time of the 20th and 21st of August 2014, two days without any clouds. The total illumination time was 26 h and 38 min. Average temperatures: 26 ^{o}C on August 20 and 27 ^{o}C on August 21, 2014.

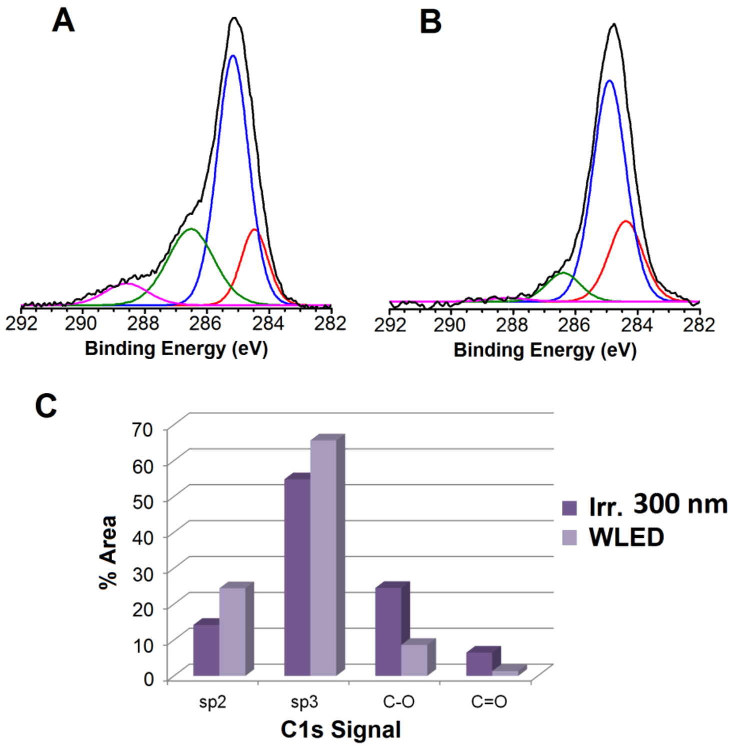

Supplementary Figure 86. (A) High Resolution C1s XPS of graphene after irradiation in Rayonet (300 nm) when in HCOOH/H_2O: 1/1 (v/v) for 24 h. (B) High Resolution C1s XPS after graphene illumination for 24 h (WLED photoreactor) in HCOOH/H_2O 1/1 (v/v). Line-colors in both cases: C-sp^2: red, C-sp^3: blue, C-O: green, C=O: violet. (C) Histogram showing the % area of the deconvoluted C1s XPS signals of A and B, for sp^2 C, sp^3 C, C-O, and C=O.

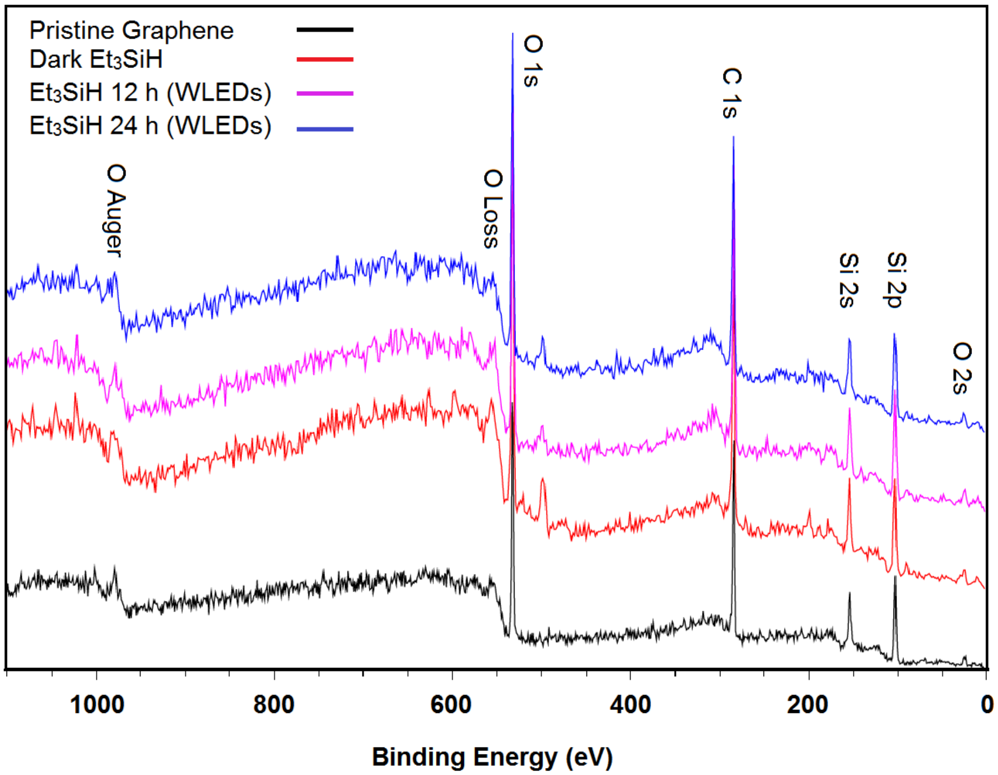

Supplementary Figure 87. Survey XPS for pristine graphene and graphene samples after photo(hydro)silylation *via* different conditions (in Et_3SiH: in the dark; in Et_3SiH - 12 h illuminated in WLED photoreactor; in Et_3SiH - 24 h illuminated in WLED photoreactor).

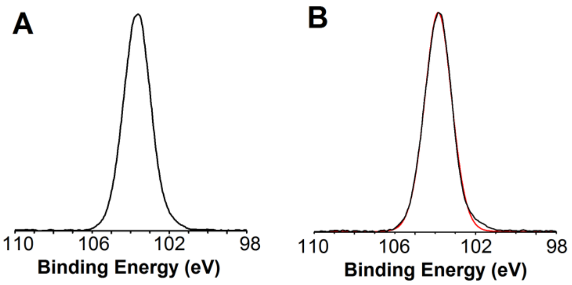

Supplementary Figure 88. High Resolution Si 2p XPS for (A) pristine graphene on SiO_2 and (B) graphene after control experiment (in neat Et_3SiH in the dark for 24 h).

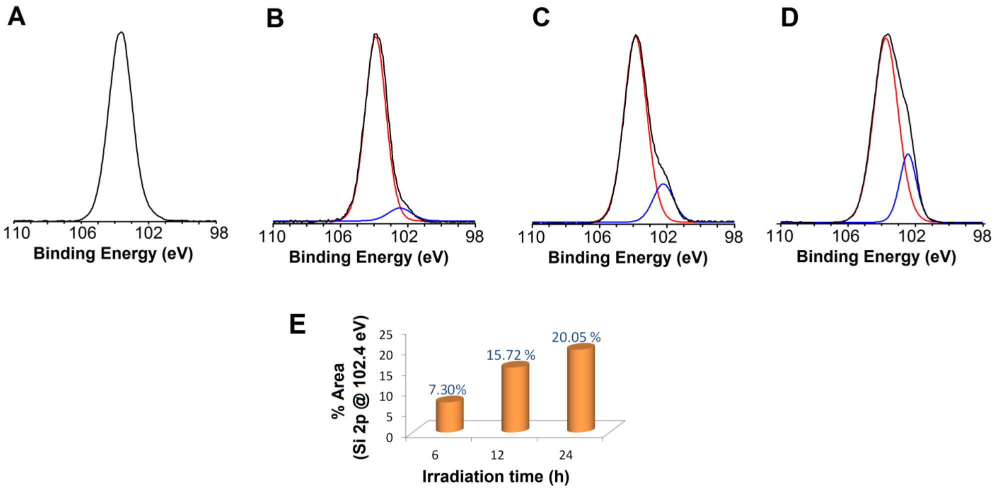

Supplementary Figure 89. High Resolution Si 2p XPS for (A) pristine graphene on SiO_2 and graphene after illumination in a WLED photoreactor in neat Et_3SiH for: (B) 6 h, (C) 12 h, and (D) 24 h. Deconvoluted signals: red: SiO_2; blue: new signal at 102.4 eV corresponding to (hydro)silylated graphene. (E) Histogram showing the % area of the new band appearing (at 102.4 eV) after photo(hydro)silylation of graphene at different illumination times.

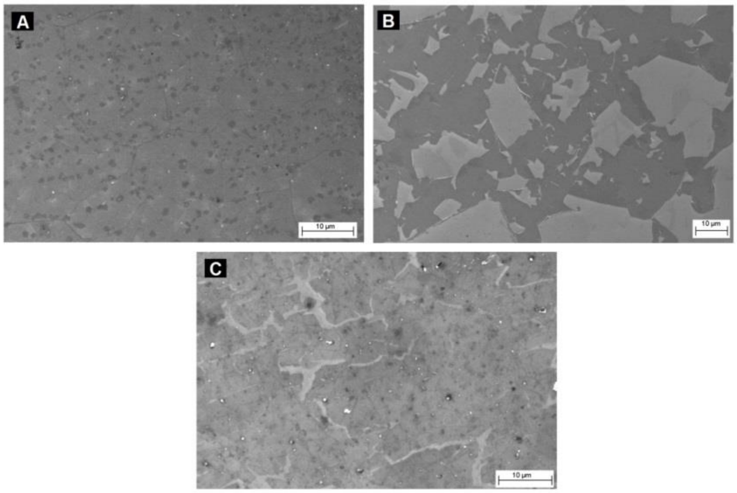

Supplementary Figure 90. (A) SEM image of a pristine CVD graphene sample on SiO_2. (B) SEM image of a CVD graphene sample after illumination for 24 h in a white LED photoreactor in $HCOOH/H_2O$ 1/1 (v/v), and (C) after illumination for 24 h in a white LED photoreactor in neat Et_3SiH.

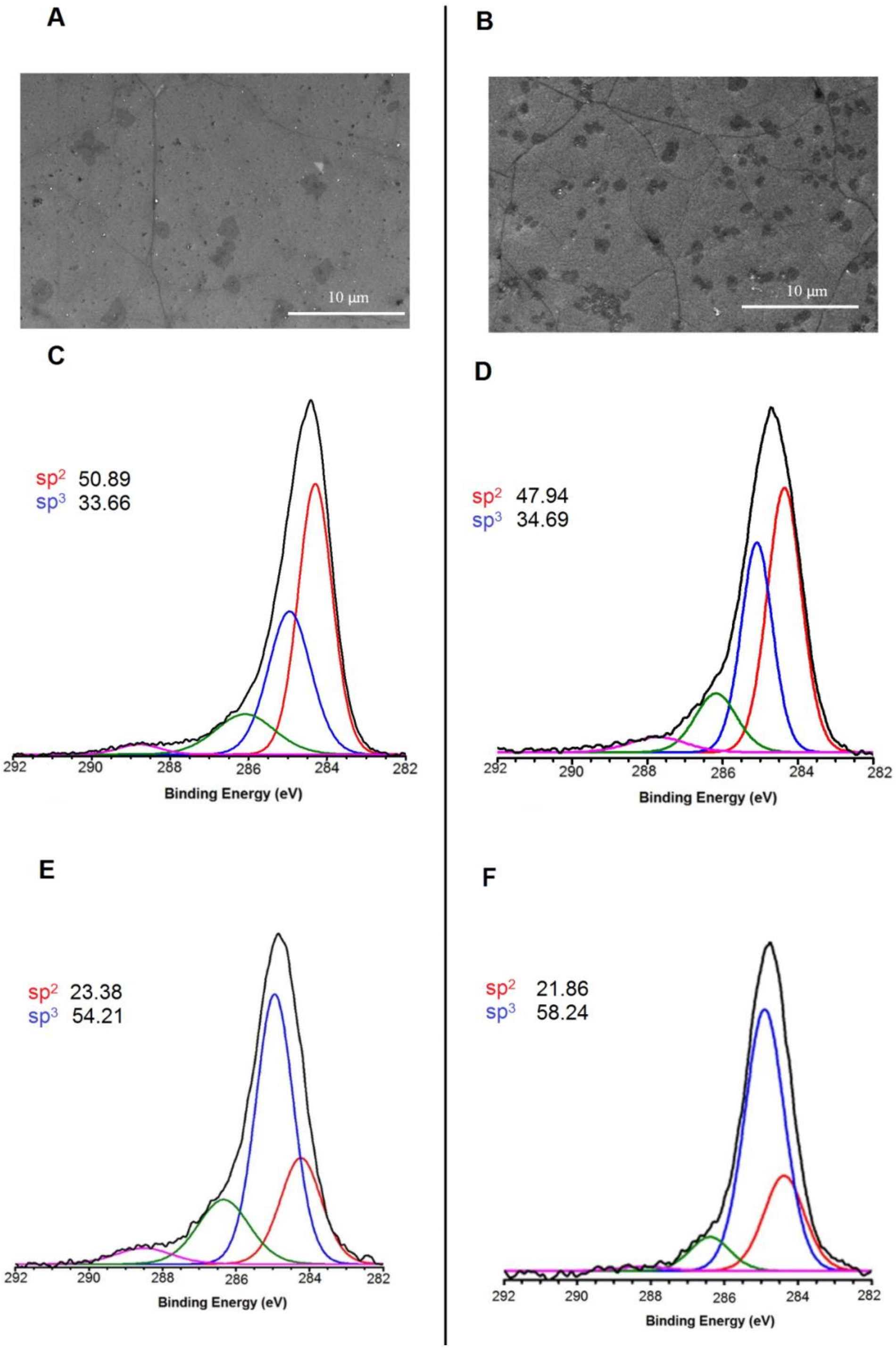

Supplementary Figure 91. CVD grown pristine graphene samples of different grain size (A) obtained from *Graphenea* and (B) obtained from the *Graphene Supermarket*. (C) High resolution C1s XPS after irradiation of the *Graphenea* samples in 1/1 $HCOOH/H_2O$ (v/v) with a white LED photoreactor for 6 h. (D) C1s XPS after irradiation of the *Graphene Supermarket* samples in 1/1 $HCOOH/H_2O$ (v/v) with a white LED photoreactor for 6 h. (E) High resolution C1s XPS after irradiation of the *Graphenea* samples in 1/1 $HCOOH/H_2O$ (v/v) with a white LED photoreactor for 24 h. (F) C1s XPS after irradiation of the *Graphene Supermarket* samples in 1/1 $HCOOH/H_2O$ (v/v) with a white LED photoreactor for 24 h.

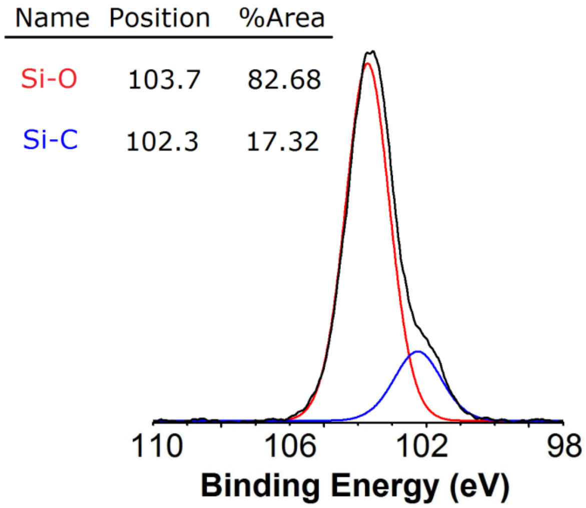

Supplementary Figure 92. High resolution Si2p XPS obtained after 24 h irradiation with white light of a CVD graphene sample in the presence of O_2 (compare with **Supplementary Figure 89D**).

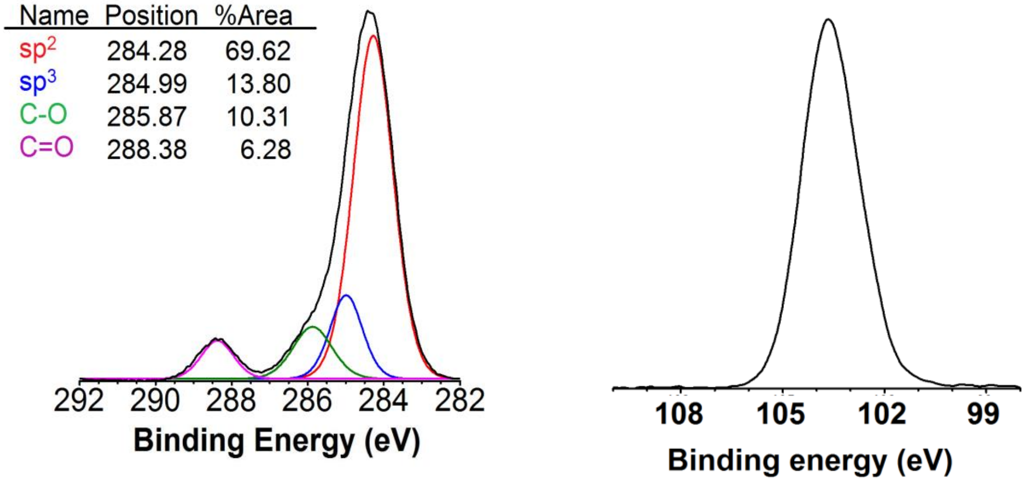

Supplementary Figure 93. Left: High resolution C1s XPS of a graphene sample after 12 h microwave irradiation at 180 °C in Et_3SiH containing 10% DMF by volume. Right: corresponding Si2p high resolution XPS.

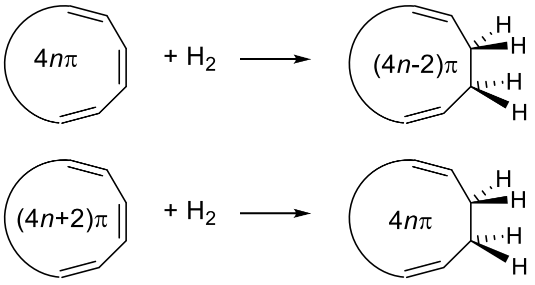

Supplementary Figure 94. General reaction scheme for the hydrogenation of annulenes with 4*n* and (4*n* + 2)π-electrons to give the product with the longest linear conjugation path.

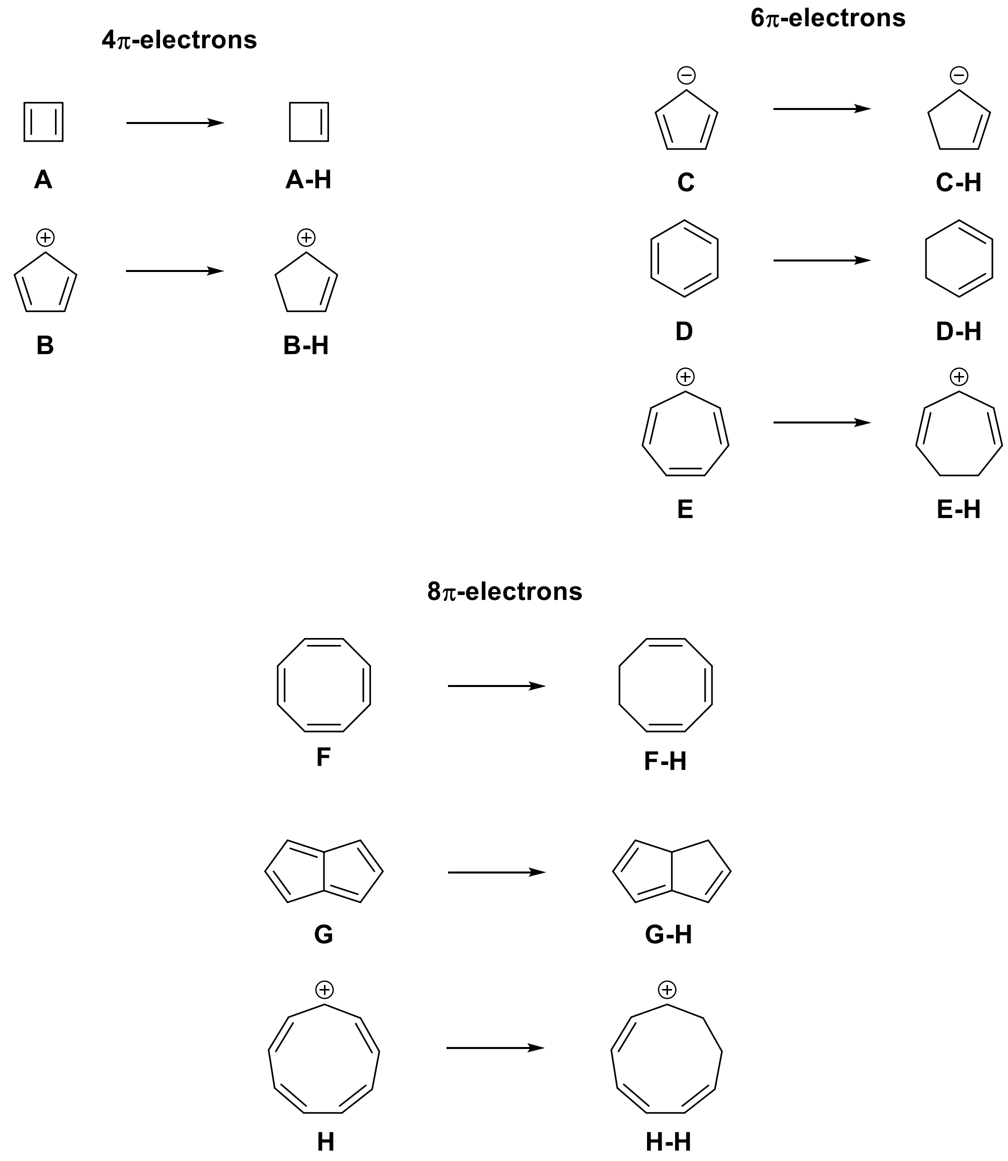

Supplementary Figure 95. Hydrogenation reactions calculated for compounds with 4, 6 and 8 π-electrons.

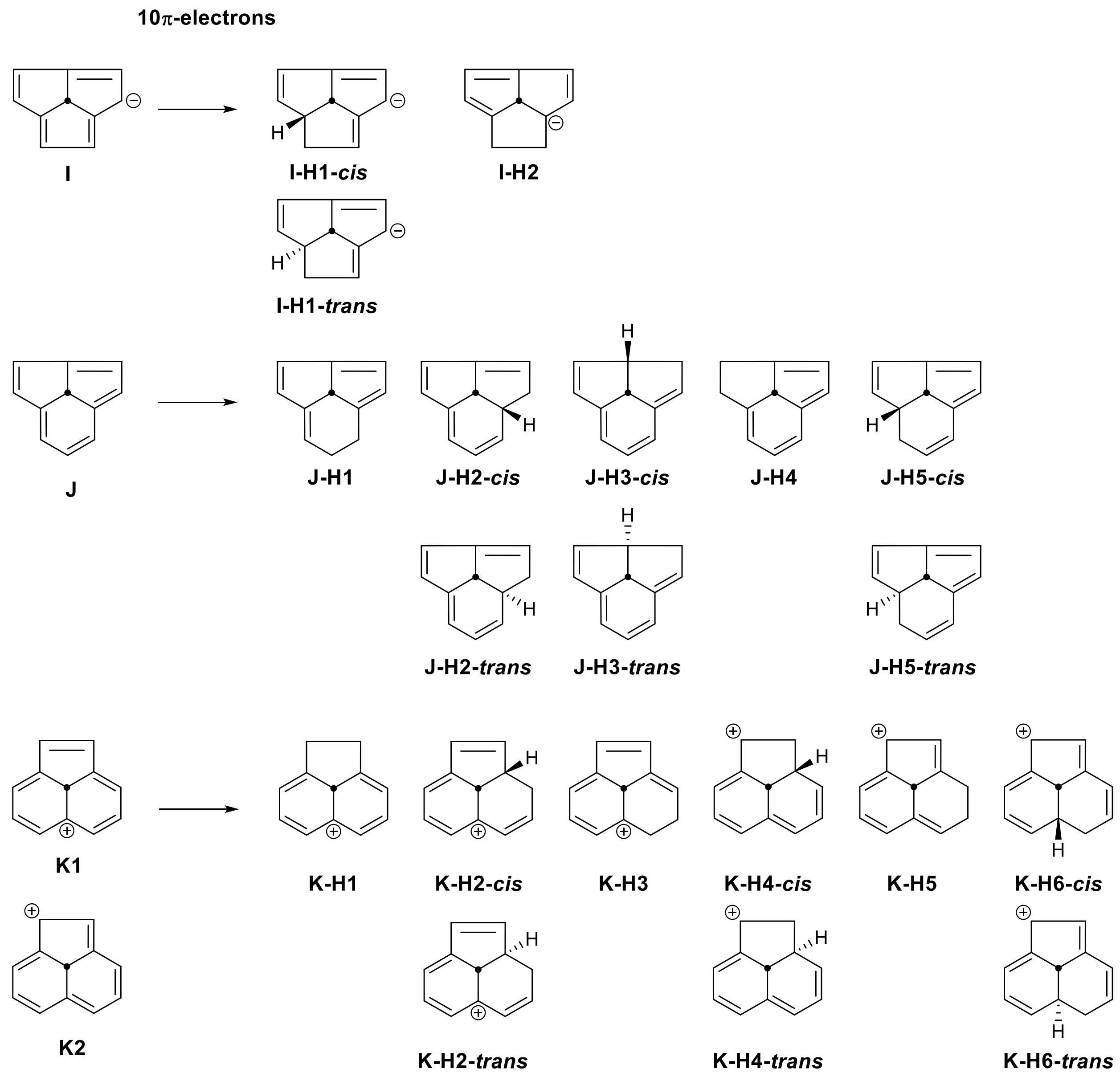

Supplementary Figure 96. Hydrogenation reactions calculated for compounds with 10 π-electrons.

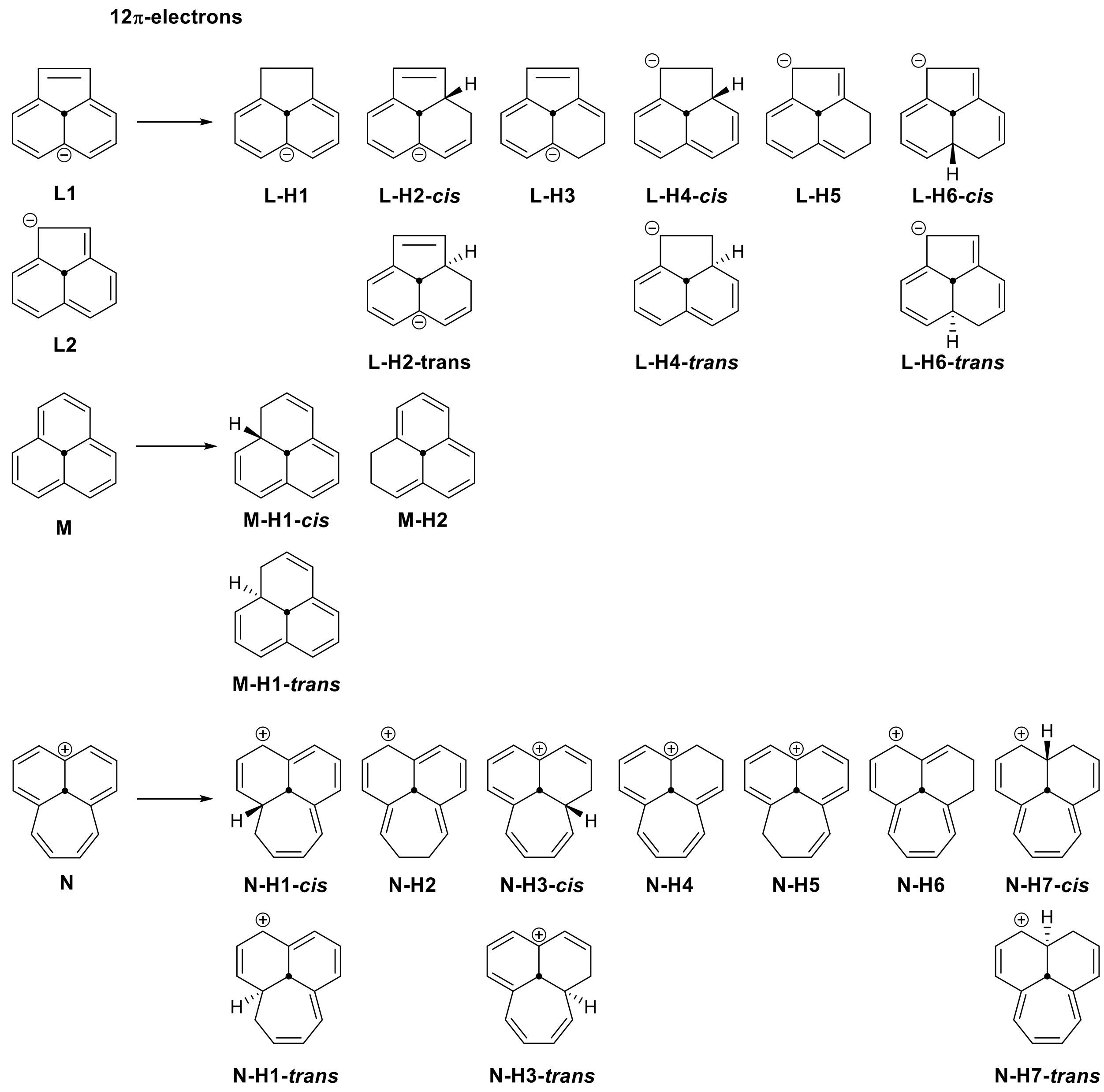

Supplementary Figure 97. Hydrogenation reactions calculated for compounds with 12 π-electrons

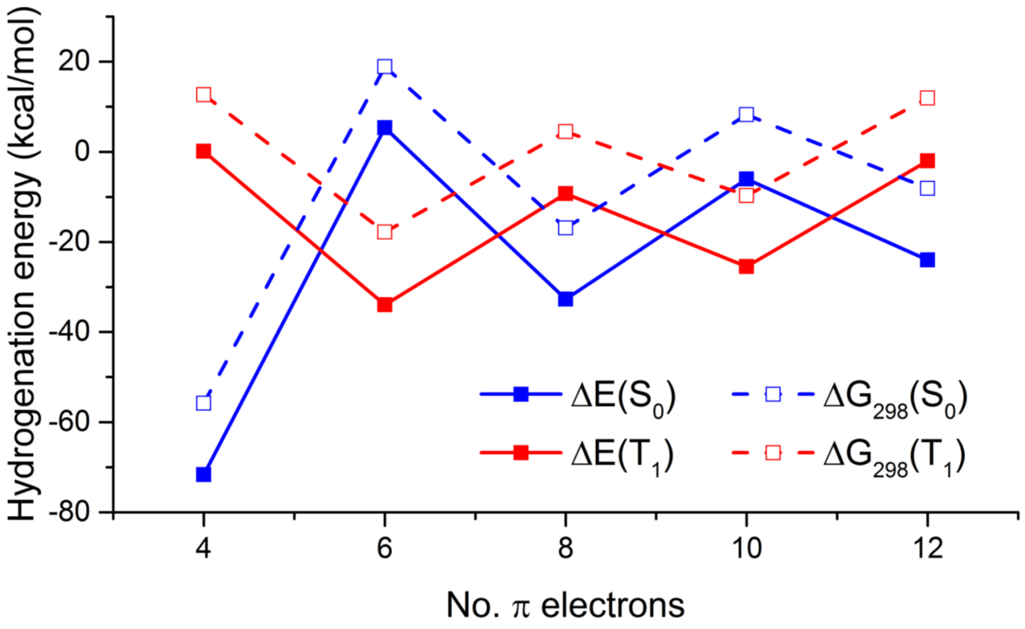

Supplementary Figure 98. Calculated average first hydrogenation energies at (U)B3LYP/6-311+G(d,p) level for a selection of annulenes (neutral, cationic, and anionic) with 4,6,8,10, and 12 π-electrons; both electronic energies (full lines) and free energies (dashed lines).

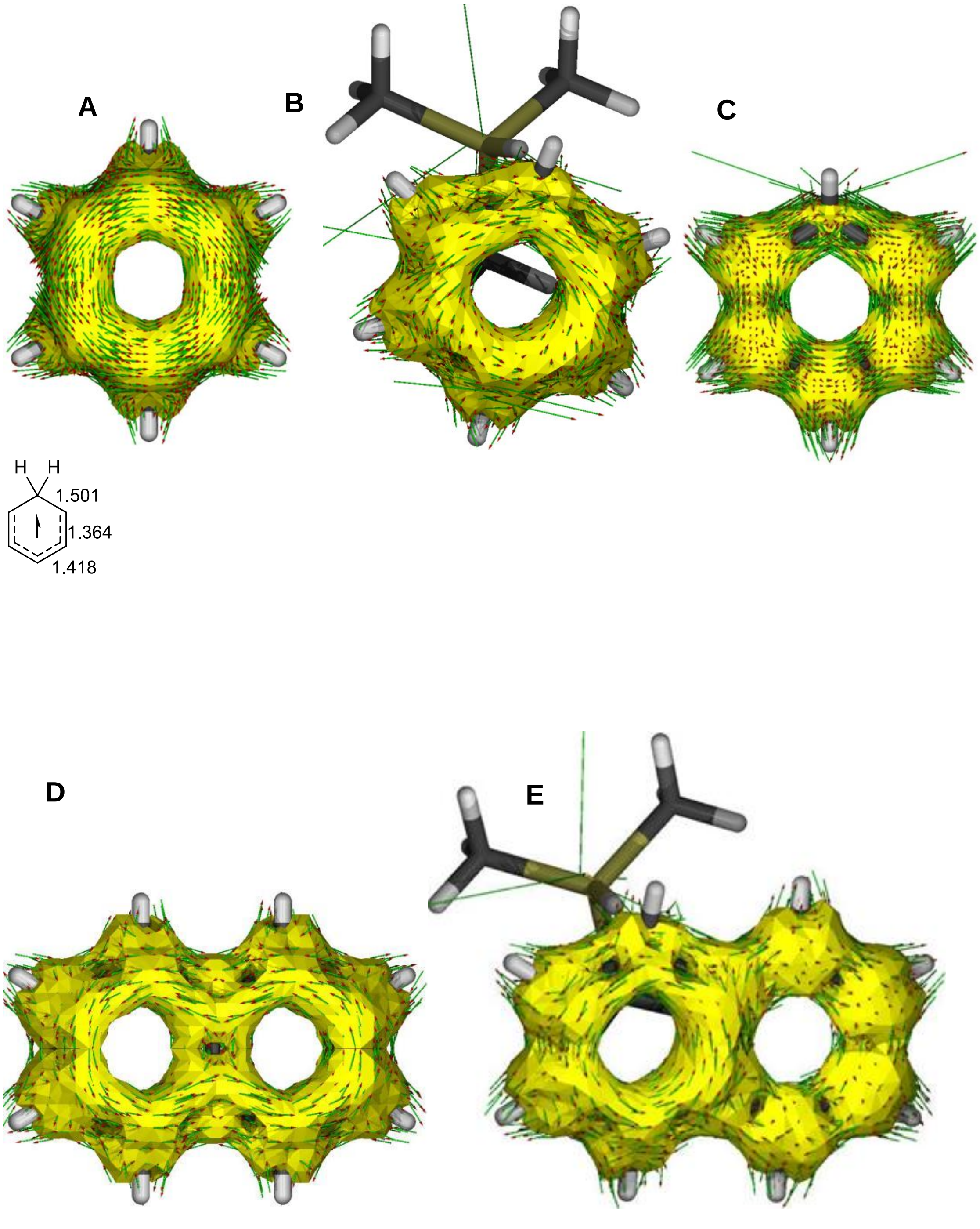
A
B
C
H H
1.501
1.364
1.418
D
E

F

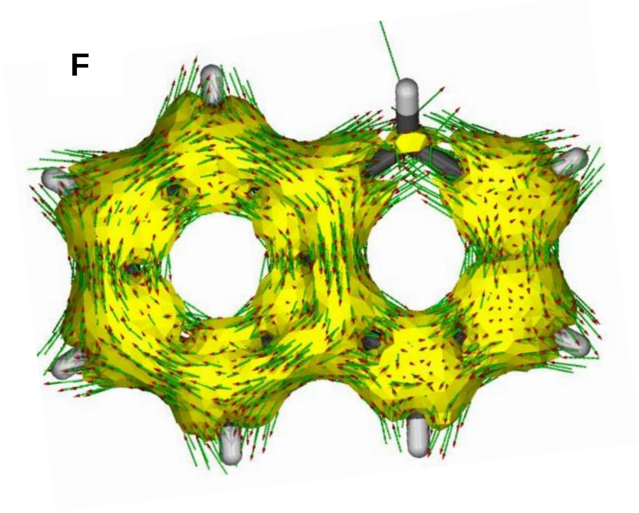

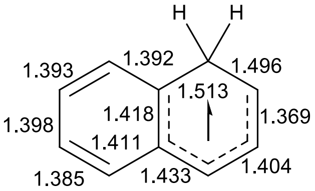

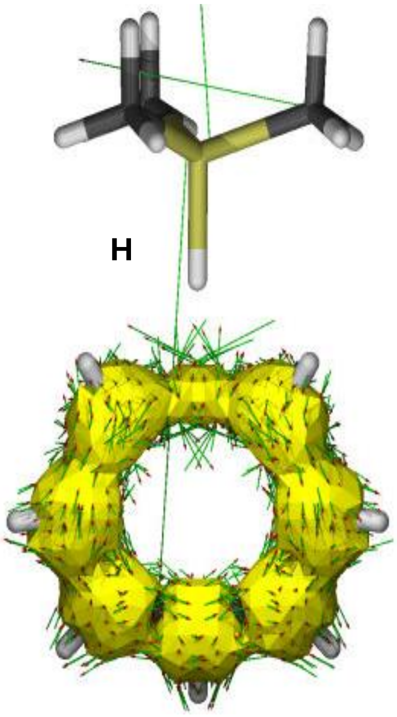

H

G

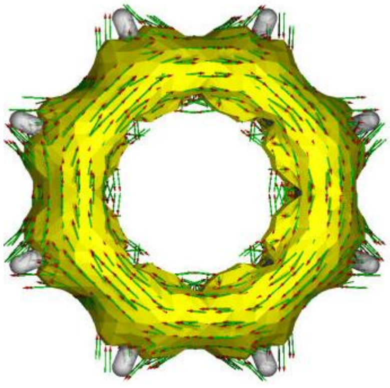

I

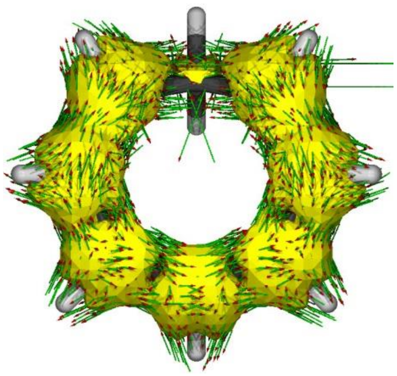

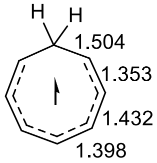

Supplementary Figure 99. ACID plots of (A) T_1 D_{2h} benzene, (B) transition state structure of T_1 benzene hydrosilylation, (C) Benzenium radical (D) T_1 D_{2h} naphthalene, (E) transition state structure of T_1 naphthalene

hydrosilylation, (F) α-naphthalenium radical (G) T_1 D_{8h} COT, and (H), transition state structure of T_1 COT hydrosilylation (I) cyclooctatetraenium radical.

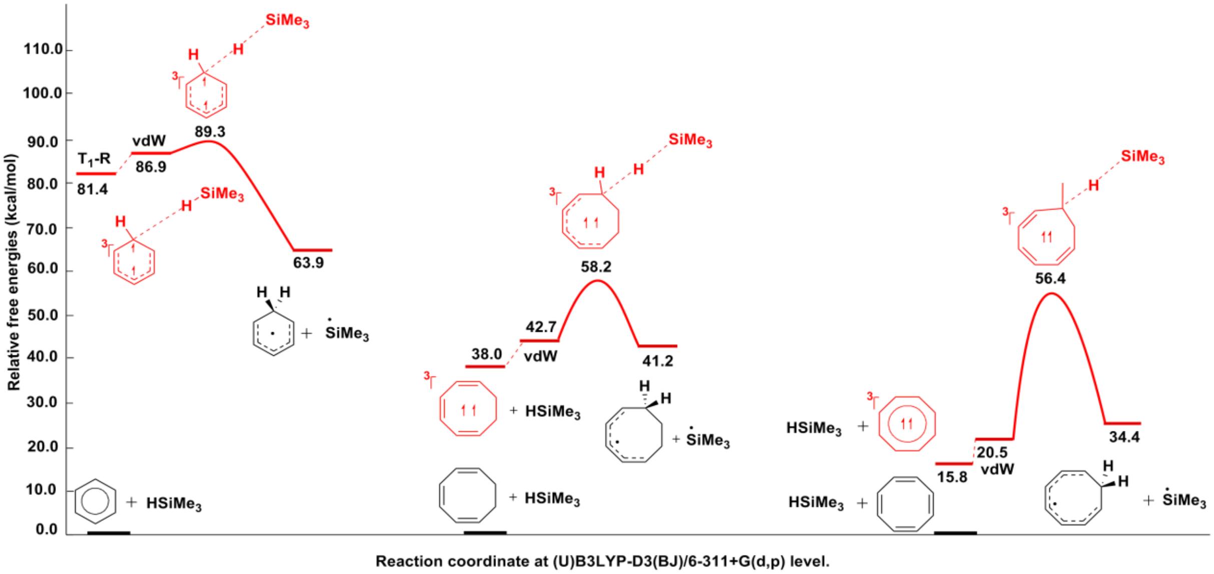

Supplementary Figure 100. Potential energy surfaces of the hydrogen-atom abstraction from Me_3SiH by T_1 state benzene, T_1 state 1,3,5-cycloocatriene, and T_1 state COT at (U)B3LYP-D3(BJ)6-311+G(d,p) level.

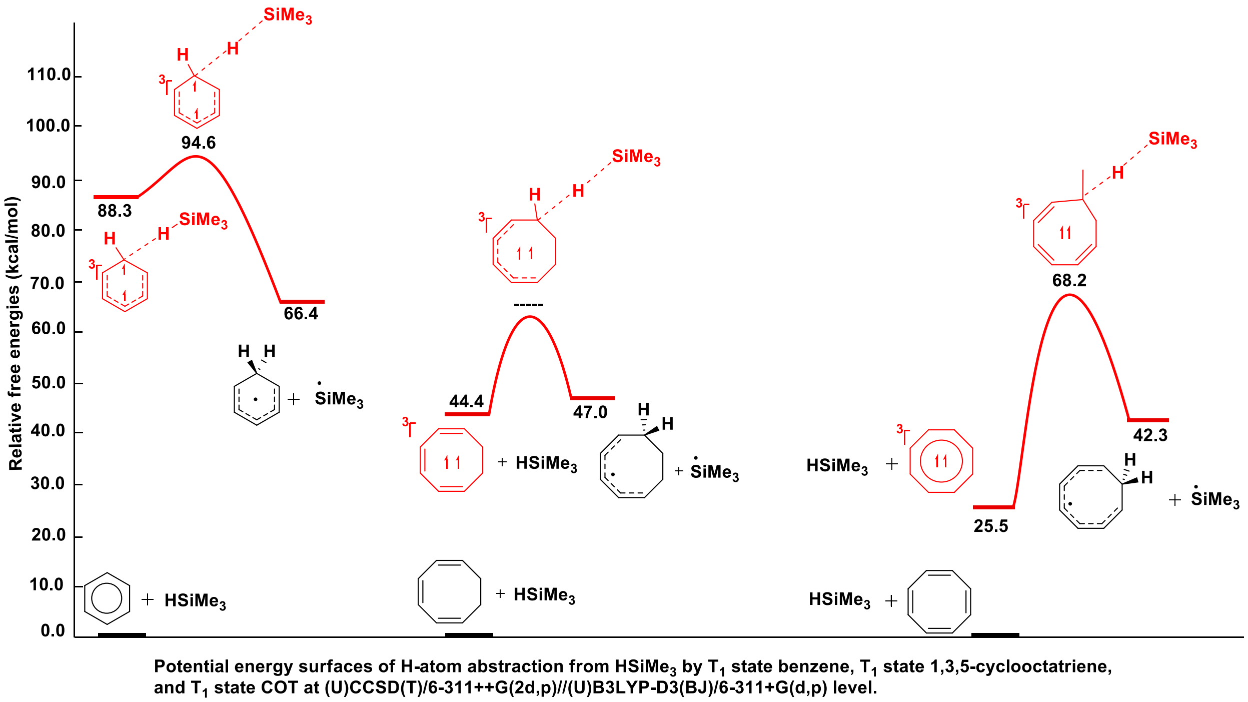

Supplementary Figure 101. Potential energy surfaces of the hydrogen-atom abstraction from Me3SiH by T1 state benzene, T1 state 1,3,5-cycloocatriene, and T_1 state COT at (U)CCSD(T)/6-311++G(2d,p)//B3LYP-D3(BJ)/6-311+G(d,p) level

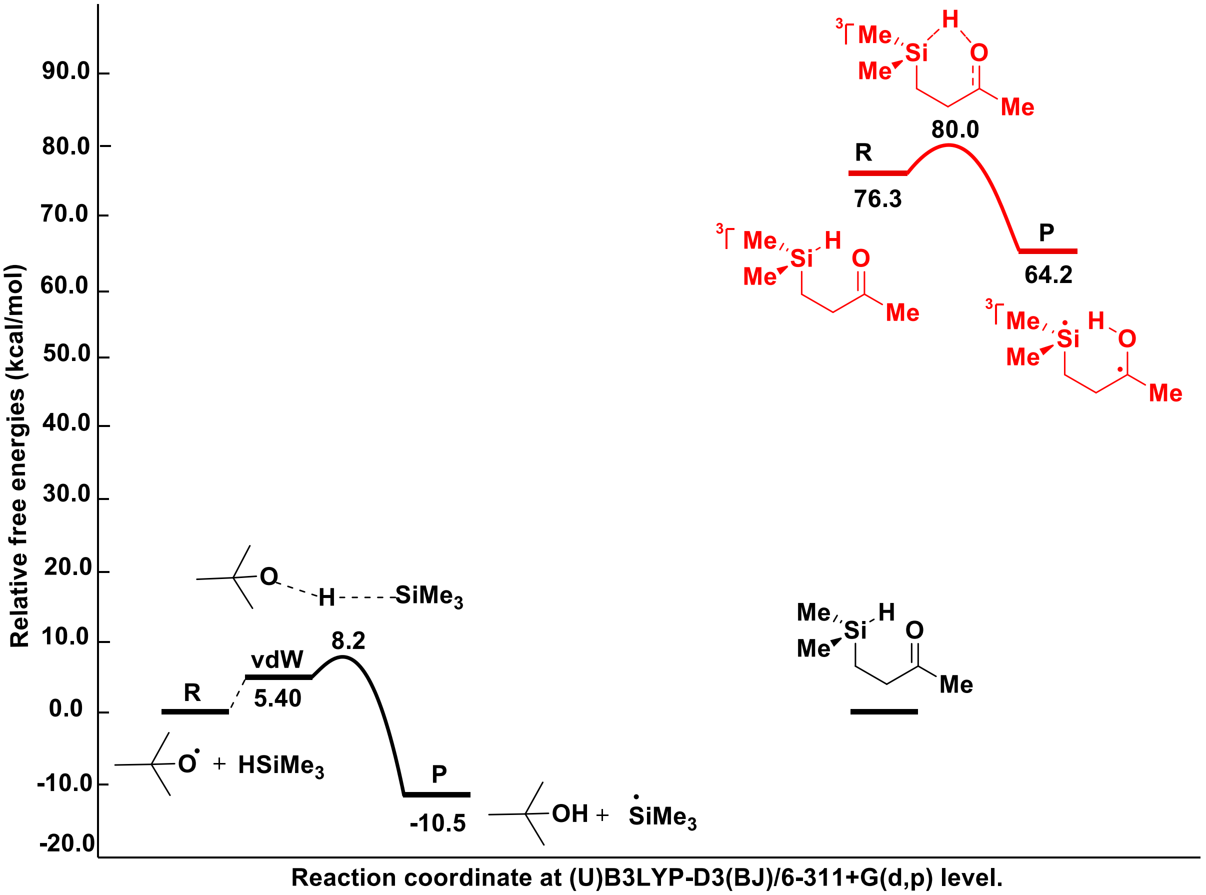

Supplementary Figure 102. Potential energy surfaces of the hydrogen-atom abstraction from Me_3SiH by ground state *tert*-butyloxy radical, and by T_1 state 4-(dimethyl)silabutan-2-one.

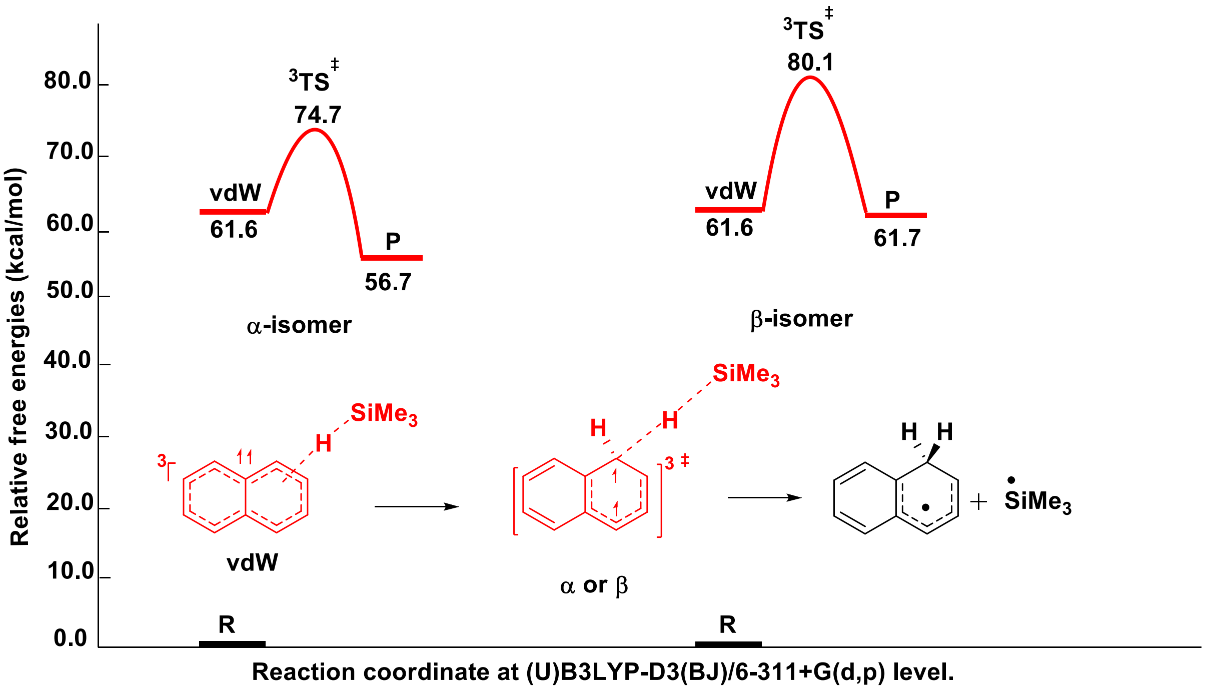

Supplementary Figure 103. Potential energy surfaces of the hydrogen-atom abstraction from Me_3SiH by T_1 state naphthalene at two different positions (α and β) at (U)B3LYP-D3(BJ)6-311+G(d,p) level.

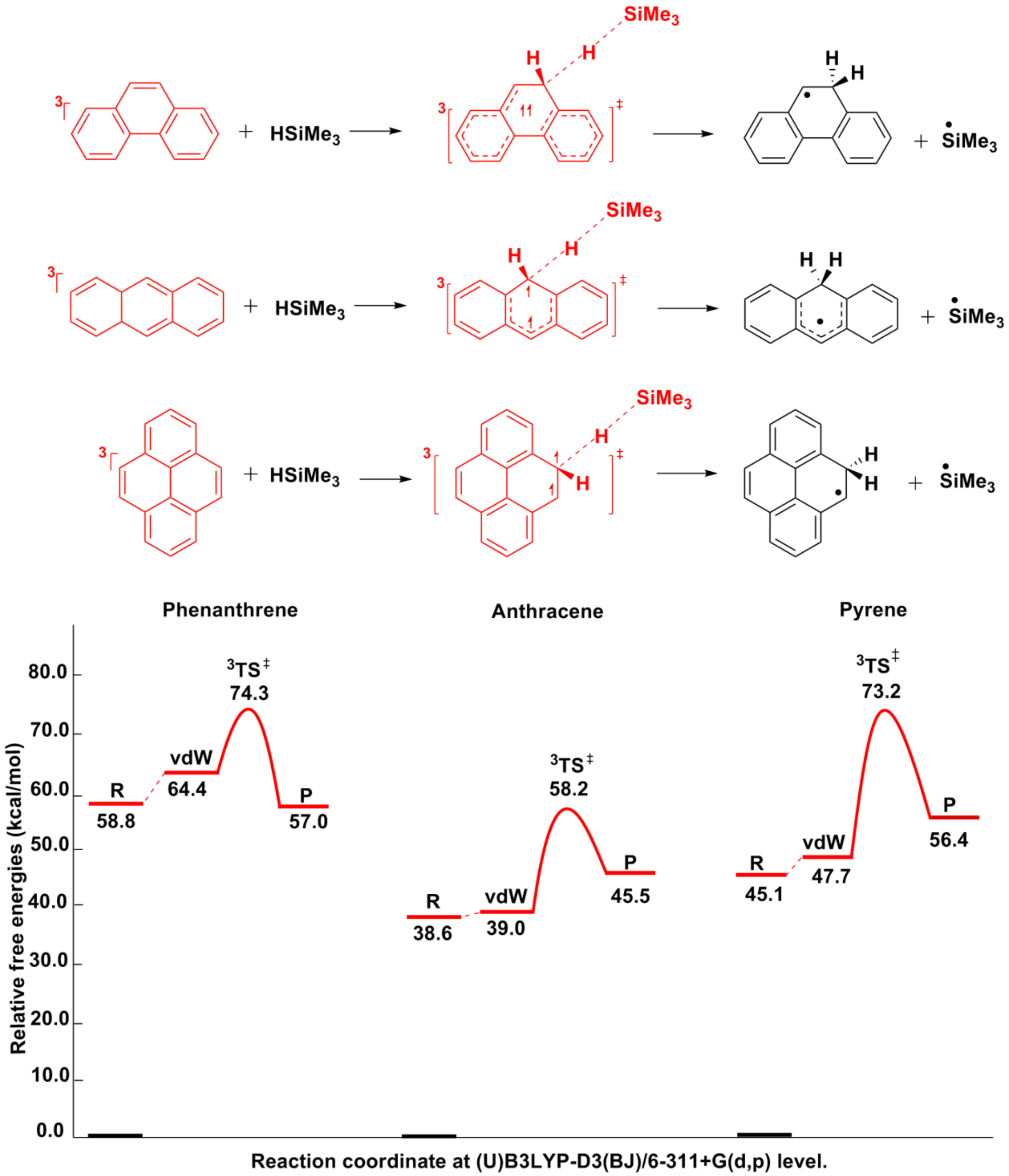

Supplementary Figure 104. Potential energy surfaces of the hydrogen-atom abstraction from Me_3SiH by T_1 state phenanthrene, T_1 state anthracene, and T_1 state pyrene (U)B3LYP-D3(BJ)6-311+G(d,p) level.

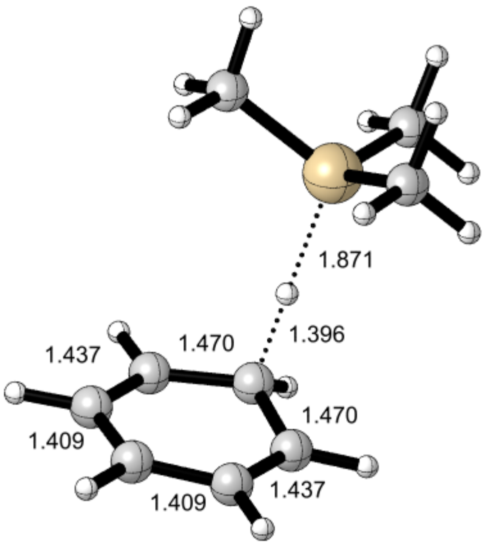

Supplementary Figure 105. CASSCF/ANO-RCC-VDZP TS geometry in S_1 for the hydrogen transfer reaction between benzene and Et_3SiH.

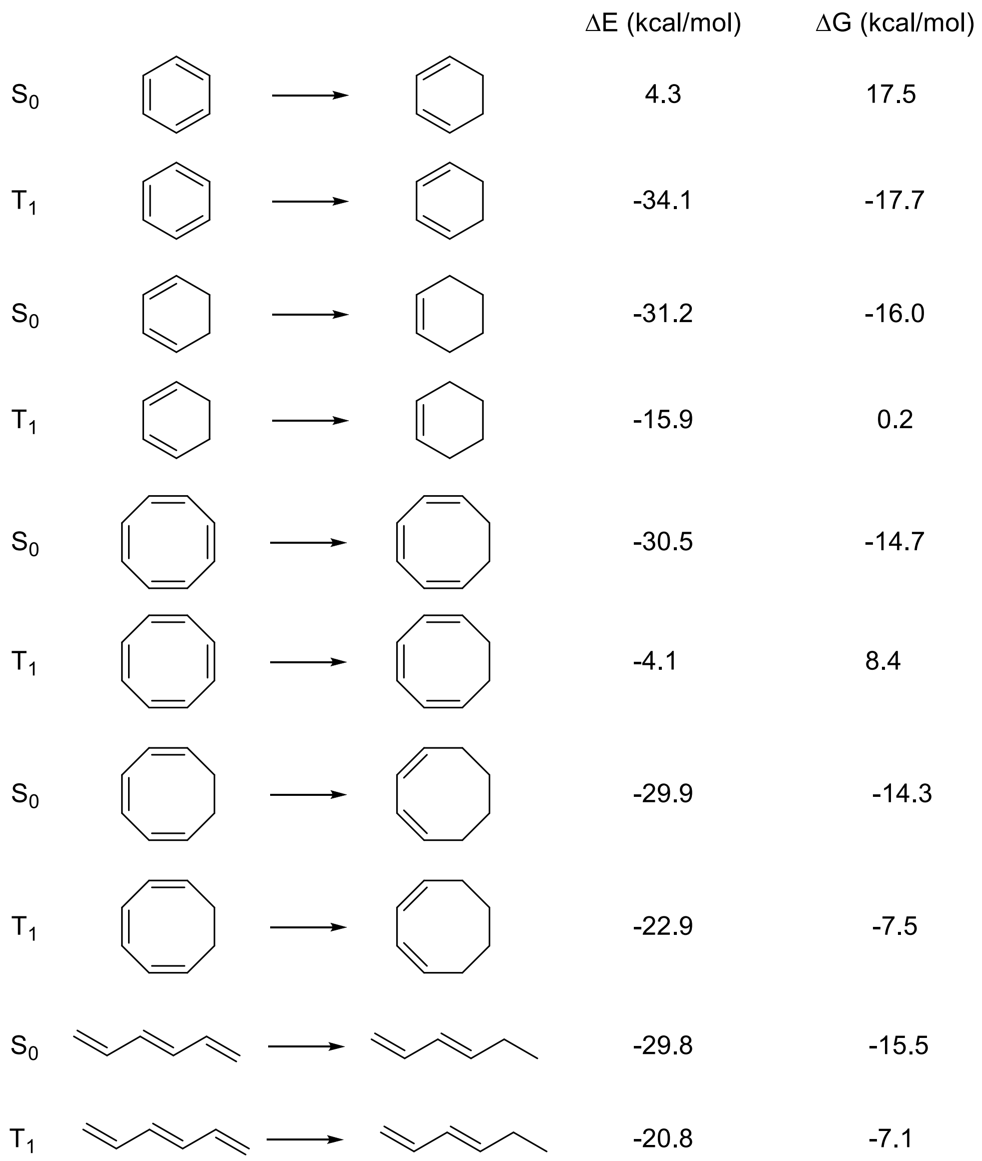

Supplementary Figure 106. Hydrogenation energies for benzene and selected non-aromatic alkenes.

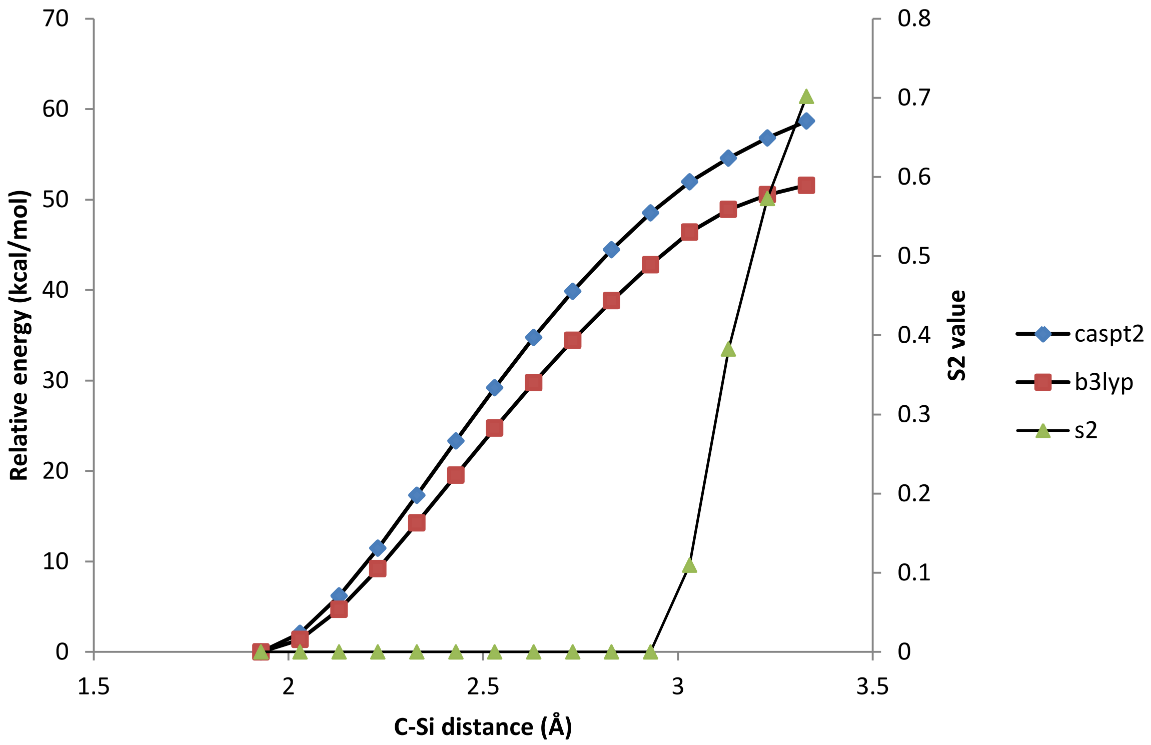

Supplementary Figure 107. Relaxed scan of the C-Si distance in trimethylsilyl cyklohexadiene at the (U)B3LYP/6-31G(d) level and CASPT2(6in6)/ANO-RCC-VDZP single point.

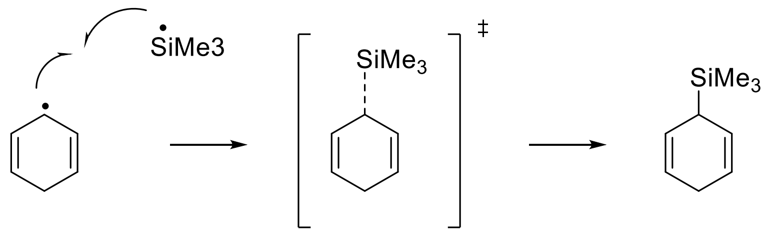

Supplementary Figure 108. Radical combination of the benzenium radical and ·SiMe3 which can occur with or without a transition state.

A

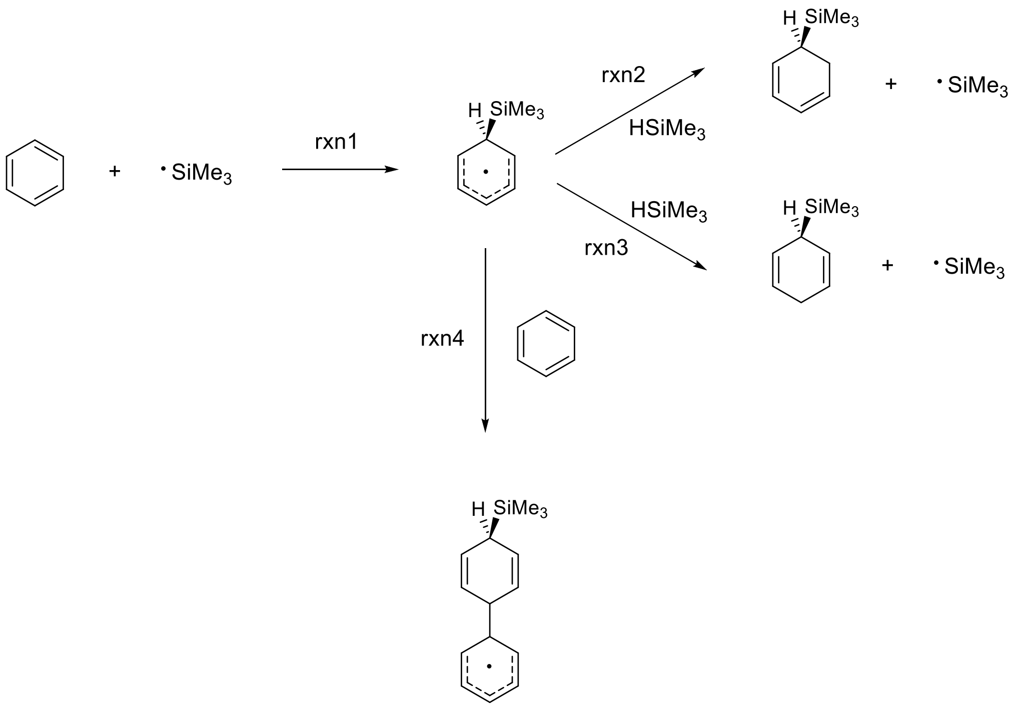

B

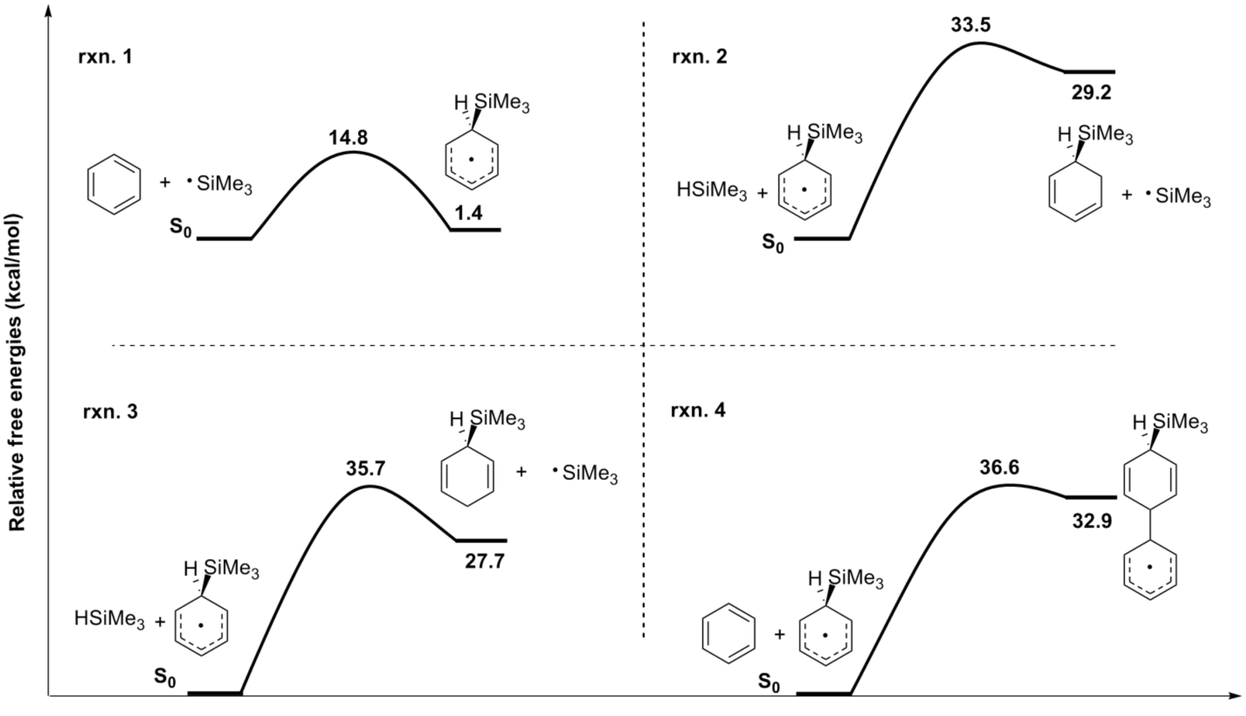

Supplementary Figure 109. (A) Reaction scheme depicting selected radical reactions and (B) activation energies for selected radical reactions calculated at (U)B3LYP-D3/6-311++G(d,p) level.

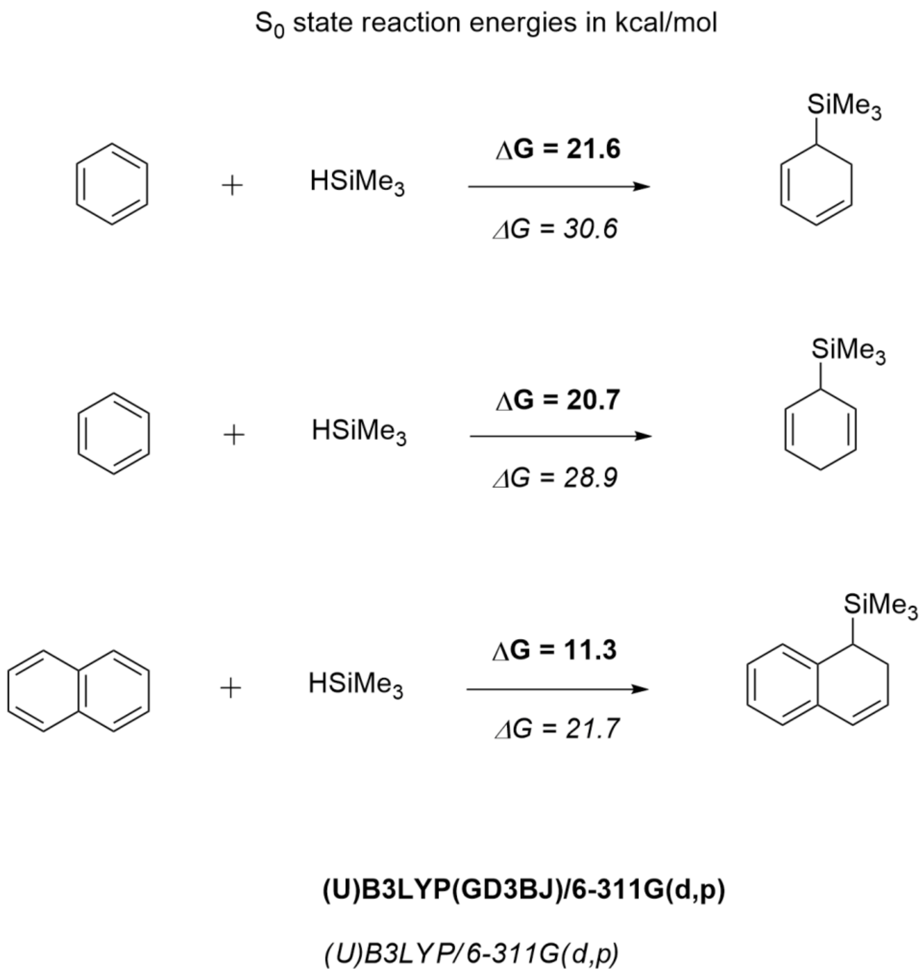

Supplementary Figure 110. Calculated hydrosilylation reaction energies in S_0 of benzene and naphthalene.

**S_0**			
B3LYP	M06	ωB97X-D	HF//B3LYP
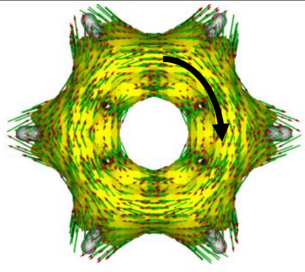	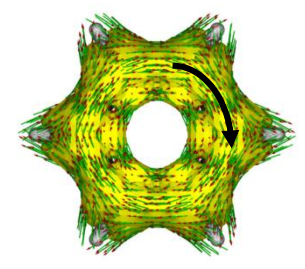	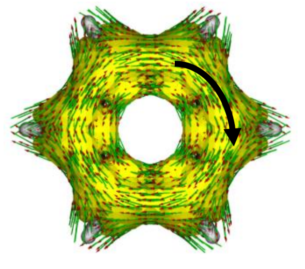	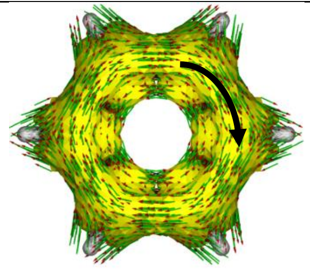
**T_1**			
B3LYP	M06	ωB97X-D	HF//B3LYP
$\langle S^2 \rangle = 2.0492$	2.0530	2.0661	2.3569
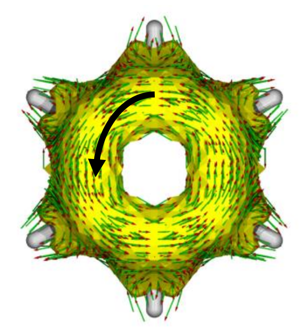	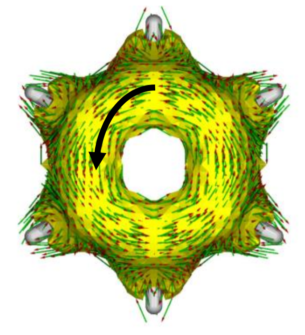	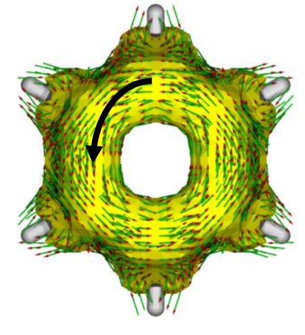	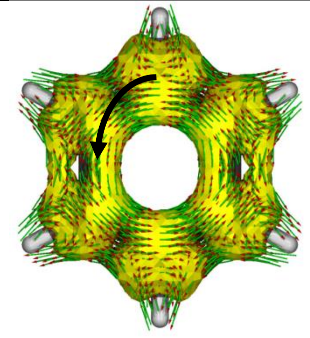

Supplementary Figure 111. ACID plots of benzene in the S_0 and T_1 states with B3LYP/6-311+G(d,p), M06/6-311+G(d,p), ωB97X-D/6-311+G(d,p) and HF/6-311+G(d,p)//B3LYP/6-311+G(d,p). Isosurface values of 0.050.

S_0			
B3LYP	M06	ωB97X-D	HF//B3LYP
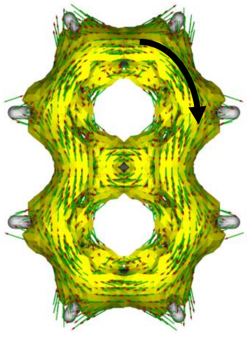	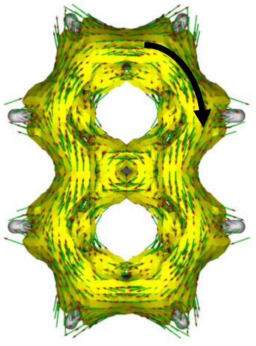	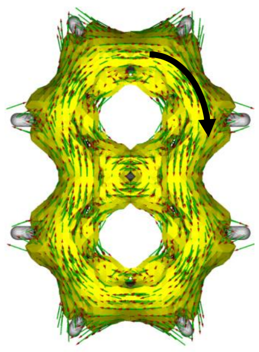	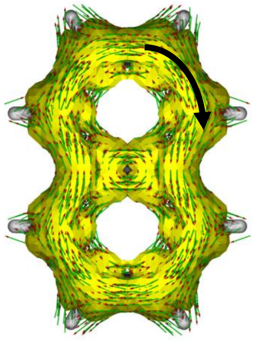
**T_1**			
B3LYP	M06	ωB97X-D	HF//B3LYP
$\langle S^2 \rangle$ = 2.0492	2.0530	2.0661	2.3569
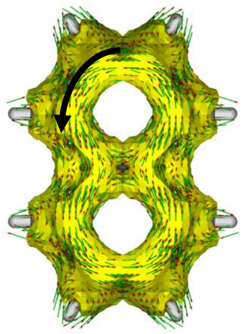	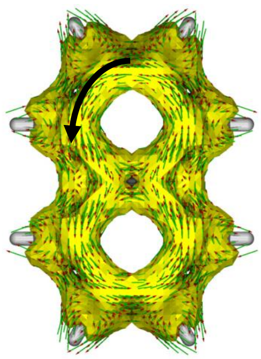	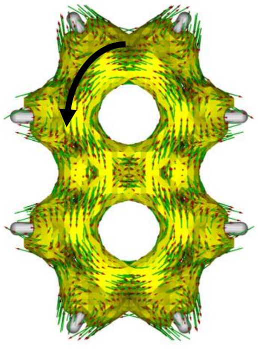	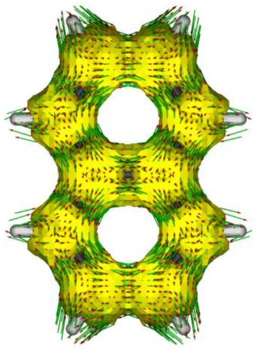

Supplementary Figure 112. ACID plots of naphthalene in the S_0 and T_1 states with B3LYP/6-311+G(d,p), M06/6-311+G(d,p), ωB97X-D/6-311+G(d,p) and HF/6-311+G(d,p)//B3LYP/6-311+G(d,p). Isosurface values of 0.050.

S_0			
B3LYP	M06	ωB97X-D	HF//B3LYP
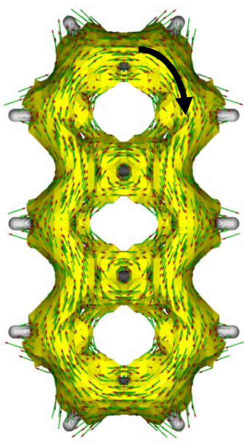	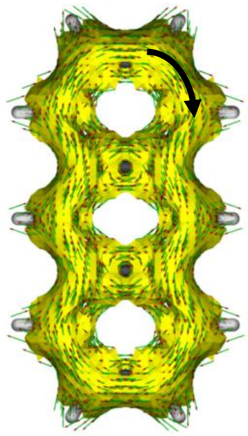	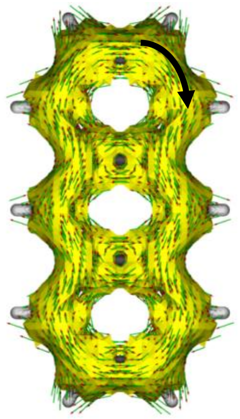	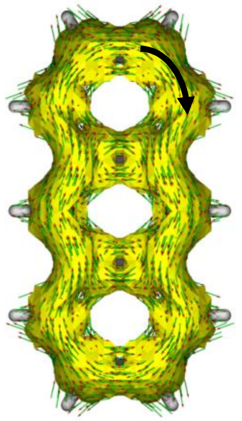
T_1			
B3LYP	M06	ωB97X-D	HF//B3LYP
$\langle S^2 \rangle$ = 2.0208	2.0271	2.0334	2.1535
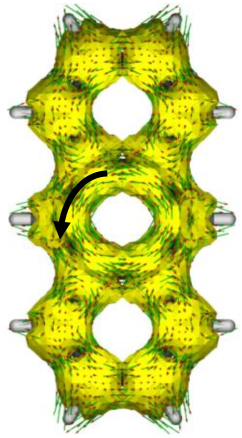	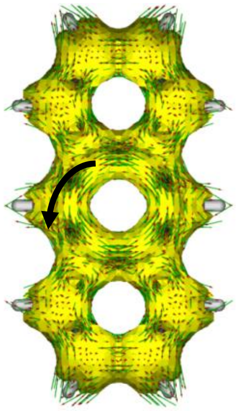	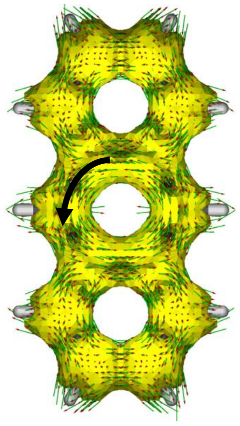	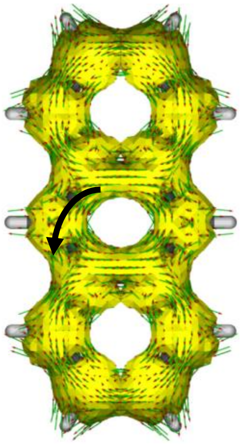

Supplementary Figure 113. ACID plots of anthracene in the S_0 and T_1 states with B3LYP/6-311+G(d,p), M06/6-311+G(d,p), ωB97X-D/6-311+G(d,p) and HF/6-311+G(d,p)//B3LYP/6-311+G(d,p). Isosurface values of 0.050.

**S_0**			
B3LYP	M06	ωB97X-D	HF//B3LYP
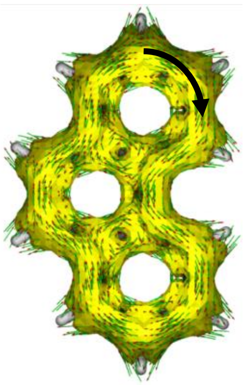	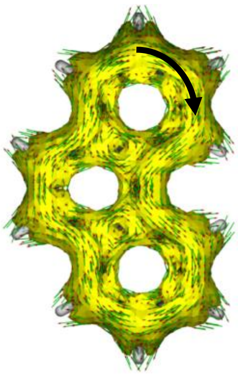	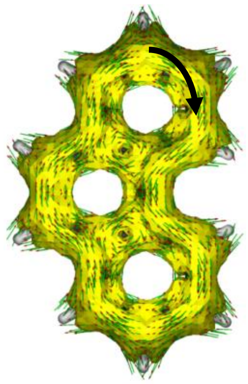	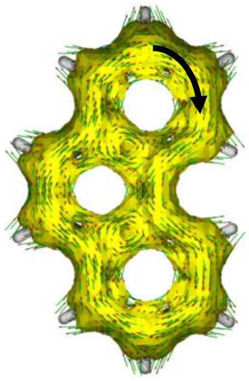
**T_1**			
B3LYP	M06	ωB97X-D	HF//B3LYP
$\langle S^2 \rangle$ = 2.0425	2.0545	2.0787	3.0134
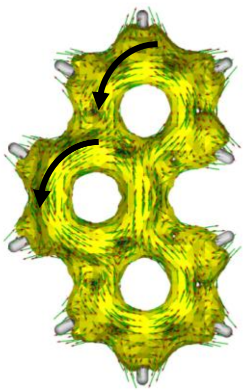	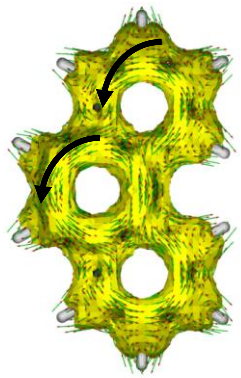	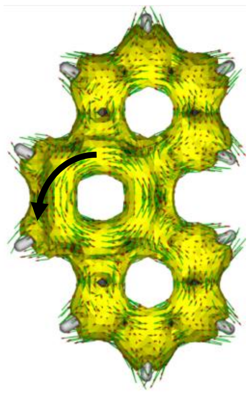	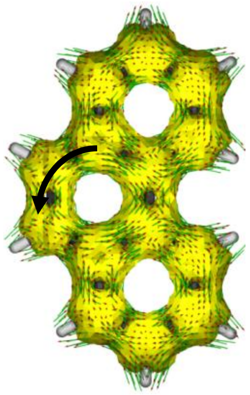

Supplementary Figure 114. ACID plots of phenanthrene in the S_0 and T_1 states with B3LYP/6-311+G(d,p), M06/6-311+G(d,p), ωB97X-D/6-311+G(d,p) and HF/6-311+G(d,p)//B3LYP/6-311+G(d,p). Isosurface values of 0.050.

**S_0**			
B3LYP	M06	ωB97X-D	HF//B3LYP
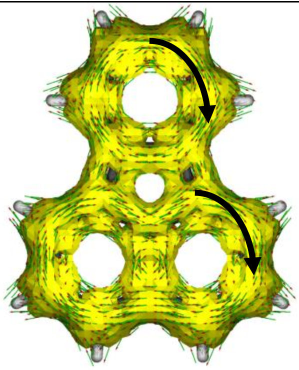	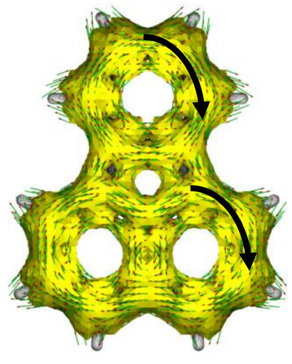	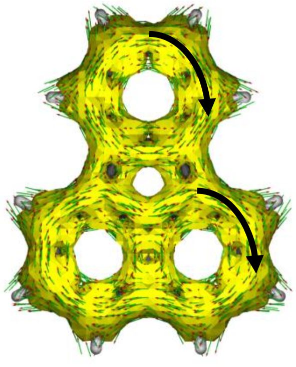	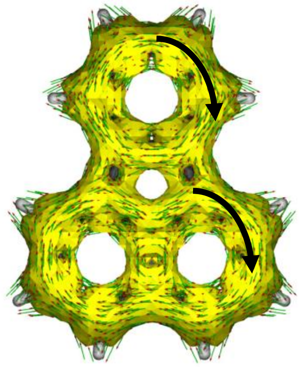
**T_1**			
B3LYP	M06	ωB97X-D	HF//B3LYP
$\langle S^2 \rangle$ = 2.0262	2.0356	2.0515	3.2092
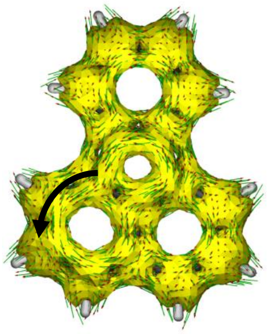	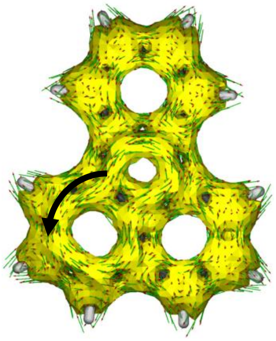	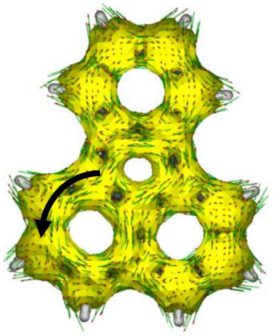	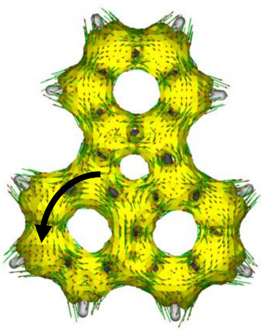

Supplementary Figure 115. ACID plots of fluoranthene in the S_0 and T_1 states with B3LYP/6-311+G(d,p), M06/6-311+G(d,p), ωB97X-D/6-311+G(d,p) and HF/6-311+G(d,p)//B3LYP/6-311+G(d,p). Isosurface values of 0.050.

**S_0**			
B3LYP	M06	ωB97X-D	HF//B3LYP
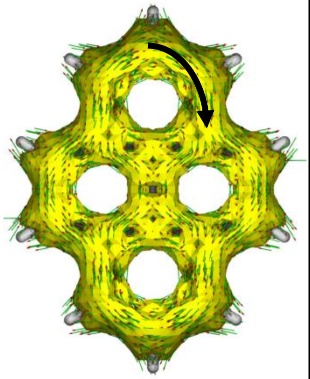	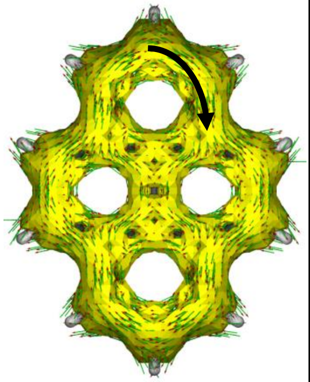	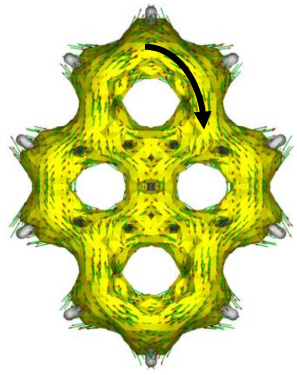	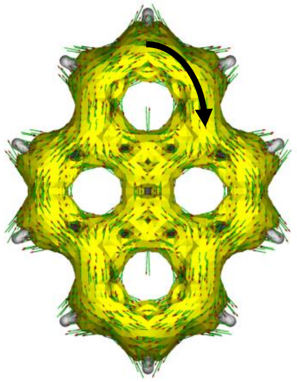
**T_1**			
B3LYP	M06	ωB97X-D	HF//B3LYP
$\langle S^2 \rangle$ =2.0366	2.0455	2.0612	2.4092
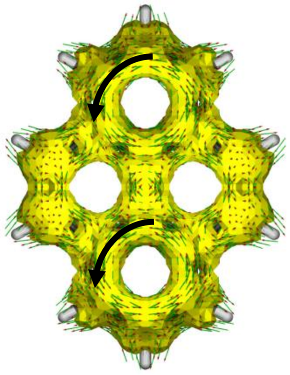	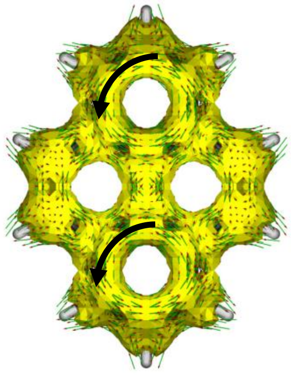	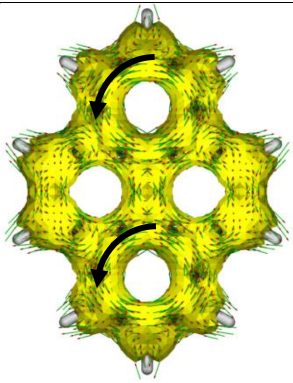	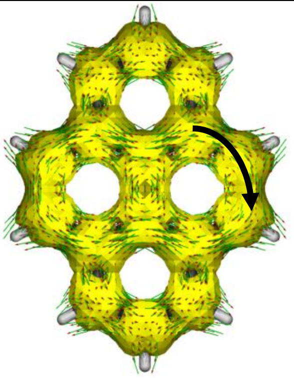

Supplementary Figure 116. ACID plots of pyrene in the S_0 and T_1 states with B3LYP/6-311+G(d,p), M06/6-311+G(d,p), ωB97X-D/6-311+G(d,p) and HF/6-311+G(d,p)//B3LYP/6-311+G(d,p). Isosurface values of 0.050.

S_0			
B3LYP	M06	ωB97X-D	HF//B3LYP
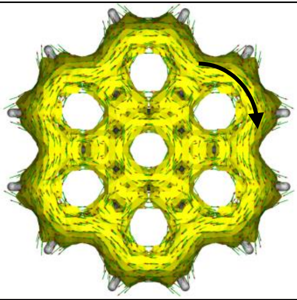	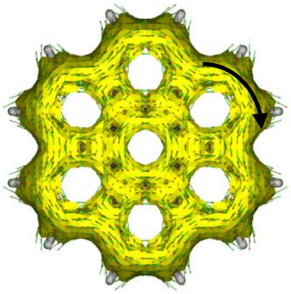	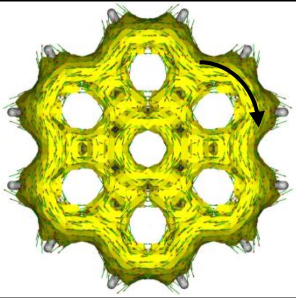	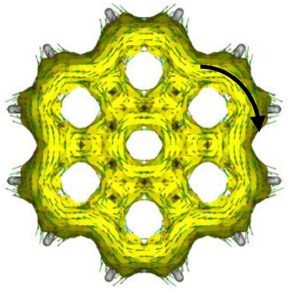
T_1			
B3LYP	M06	ωB97X-D	HF//B3LYP
$\langle S^2 \rangle$ = 2.0476	2.0612	2.1013	2.9081
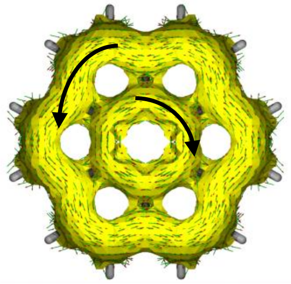	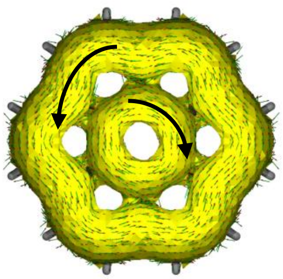	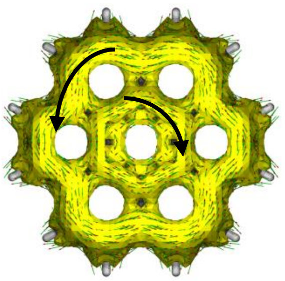	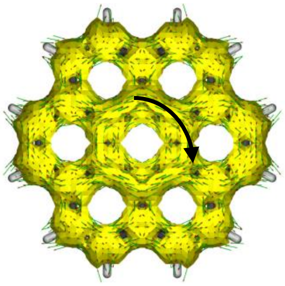

Supplementary Figure 117. ACID plots of coronene in the S_0 and T_1 states with B3LYP/6-311+G(d,p), M06/6-311+G(d,p), ωB97X-D/6-311+G(d,p) and HF/6-311+G(d,p)//B3LYP/6-311+G(d,p). Isosurface values of 0.050.

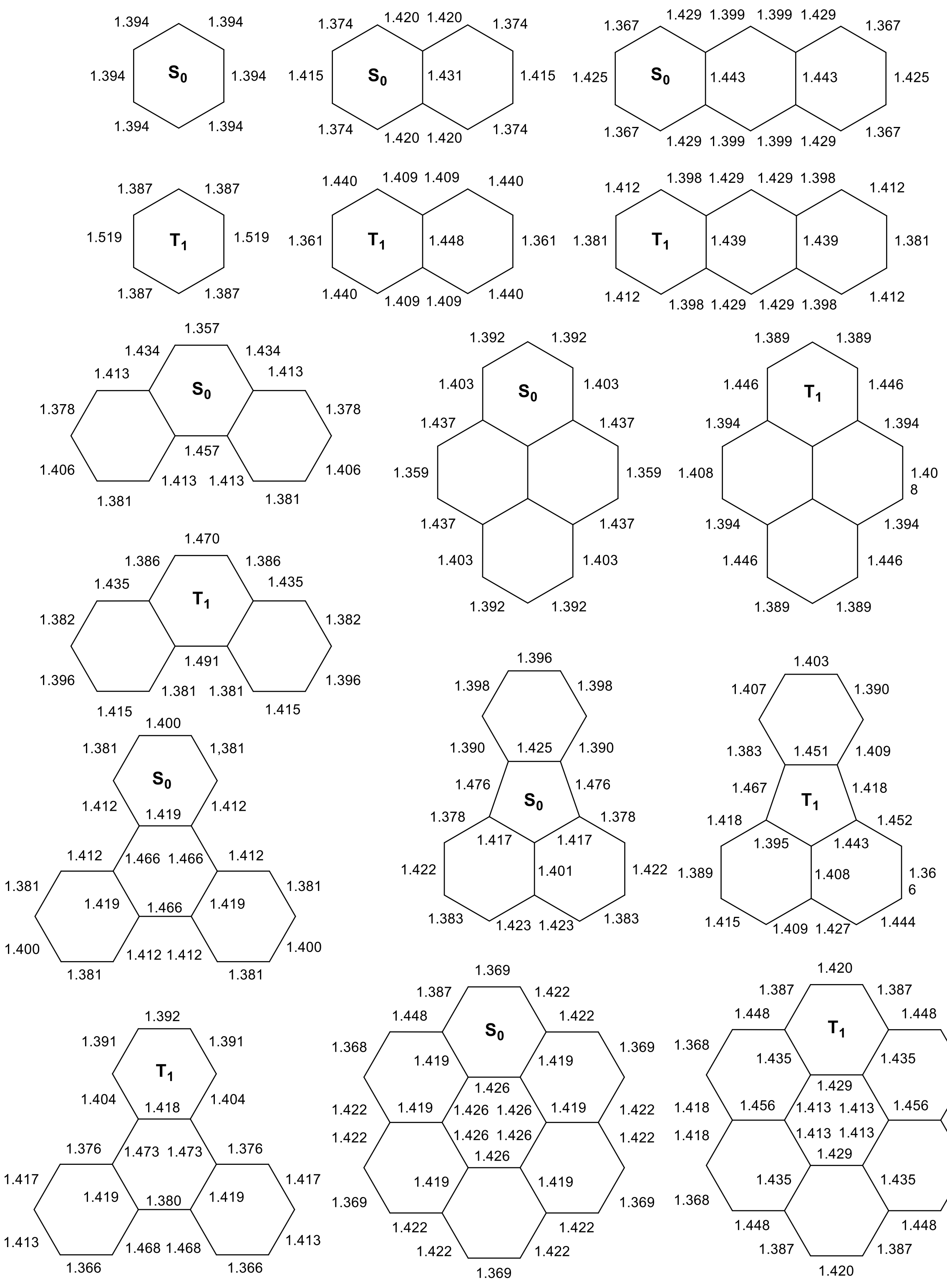

Supplementary Figure 118. Bond lengths for polycyclic aromatic hydrocarbons studied with the ACID method. Geometries were calculated at the B3LYP/6-311+(d,p) level, except for coronene, for which B3LYP/6-311G(d,p) was used.

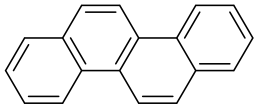

Spin density	ACID plot
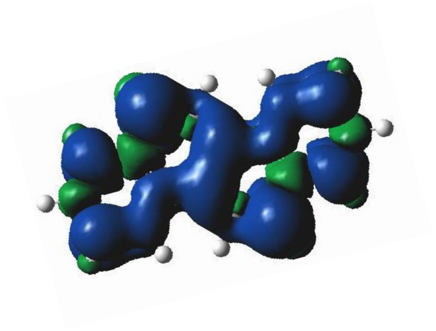	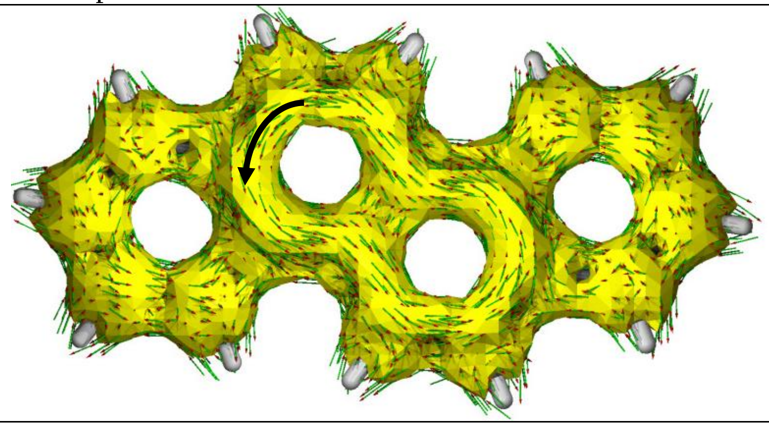

Supplementary Figure 119| Additional ACID plots for a PAH (Chrysene) indicating T_1 antiaromaticity localization. B3LYP/6-311+G(d,p) optimizations and ACID plots at the CSGT-B3LYP/6-311+G(d,p) level.

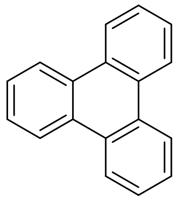

Spin density	ACID plot
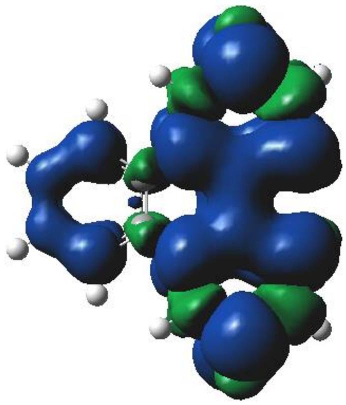	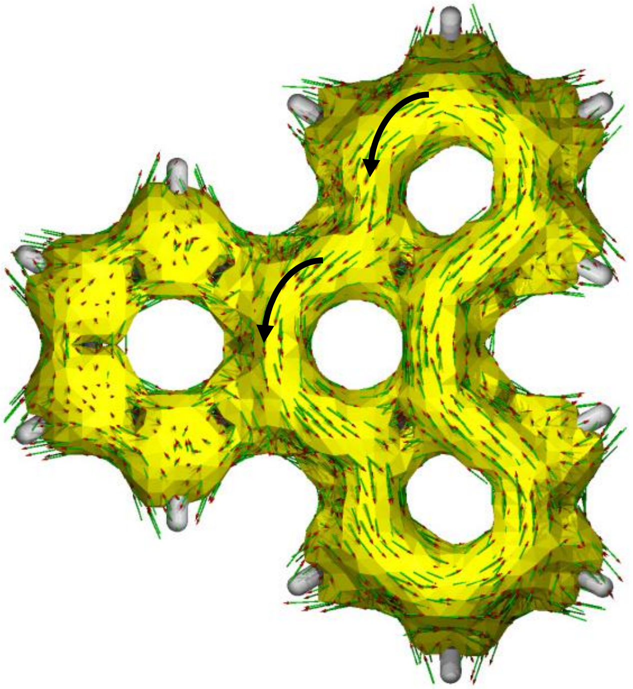

Supplementary Figure 120 | Additional ACID plots for a PAH (triphenylene) indicating T_1 antiaromaticity localization. B3LYP/6-311+G(d,p) optimizations and ACID plots at the CSGT-B3LYP/6-311+G(d,p) level.

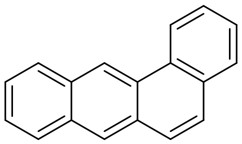

Spin density	ACID plot
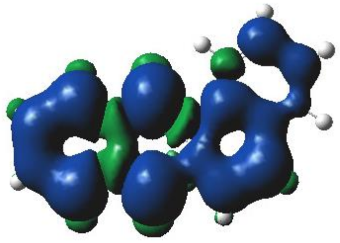	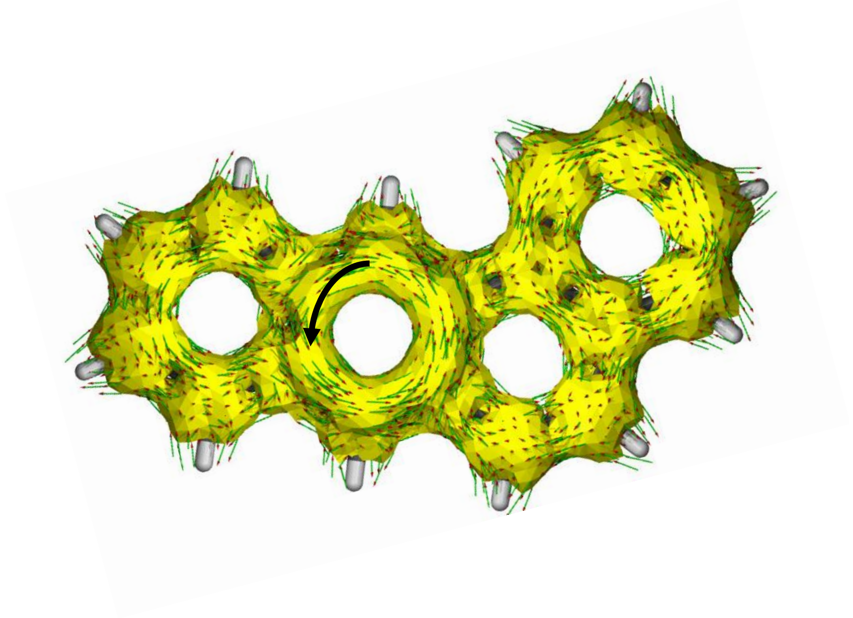

Supplementary Figure 121| Additional ACID plots for a PAH (benz(α)anthracene) indicating T_1 antiaromaticity localization. B3LYP/6-311+G(d,p) optimizations and ACID plots at the CSGT-B3LYP/6-311+G(d,p) level.

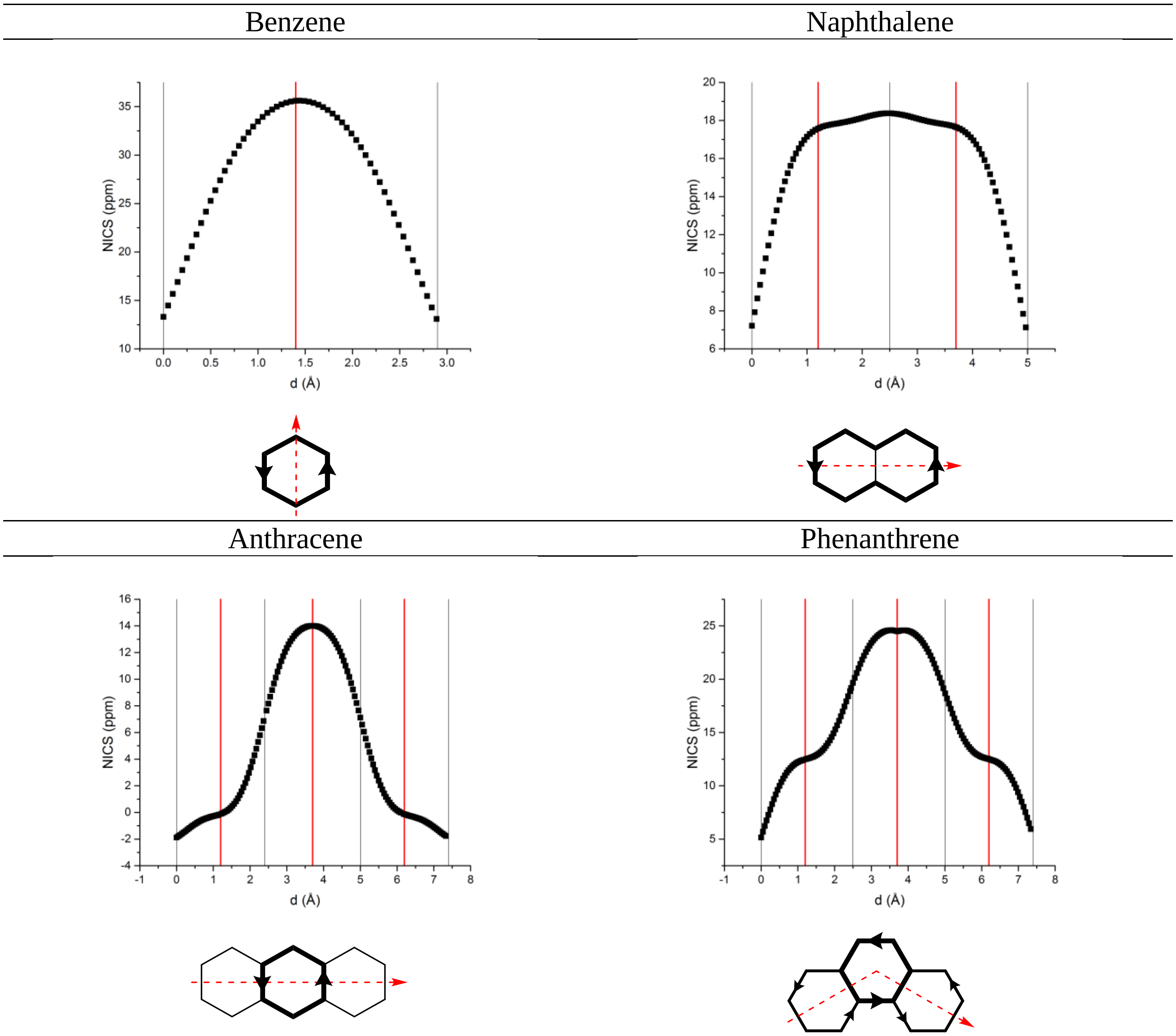

Supplementary Figure 122. NICS-XY scans for benzene, naphthalene, anthracene and phenanthrene in T_1.

Pyrene	Pyrene
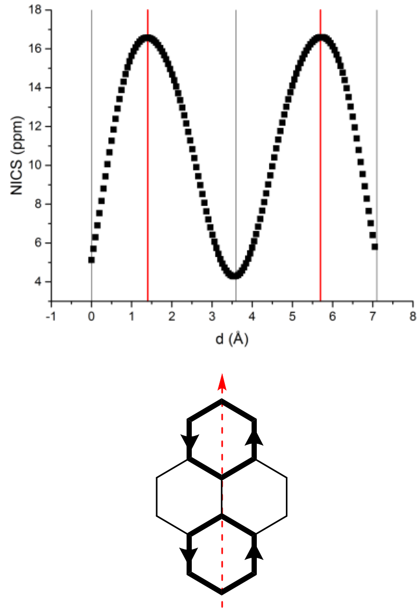	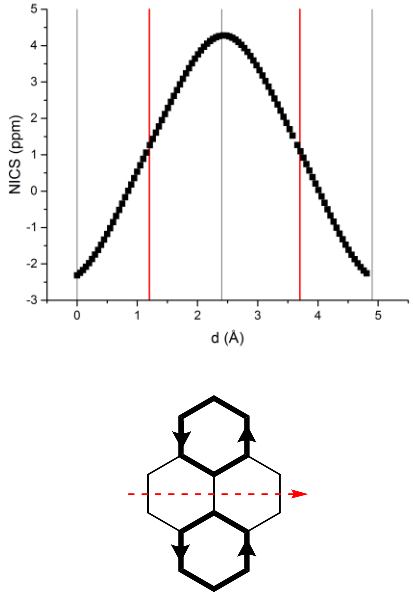

Fluoranthene	Fluoranthene
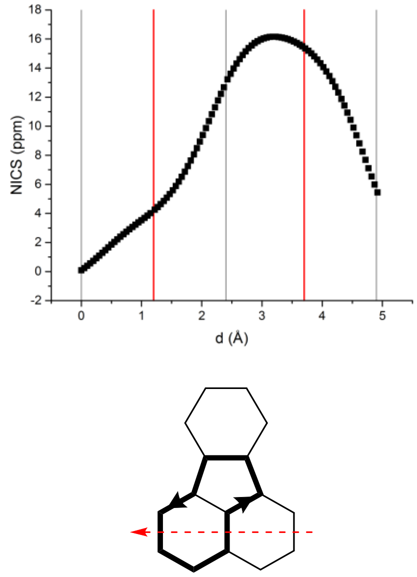	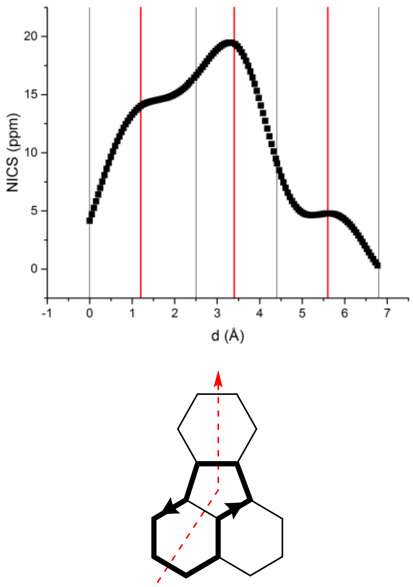

Supplementary Figure 123. NICS-XY scans for pyrene and fluoranthene in T_1.

Coronene	Coronene

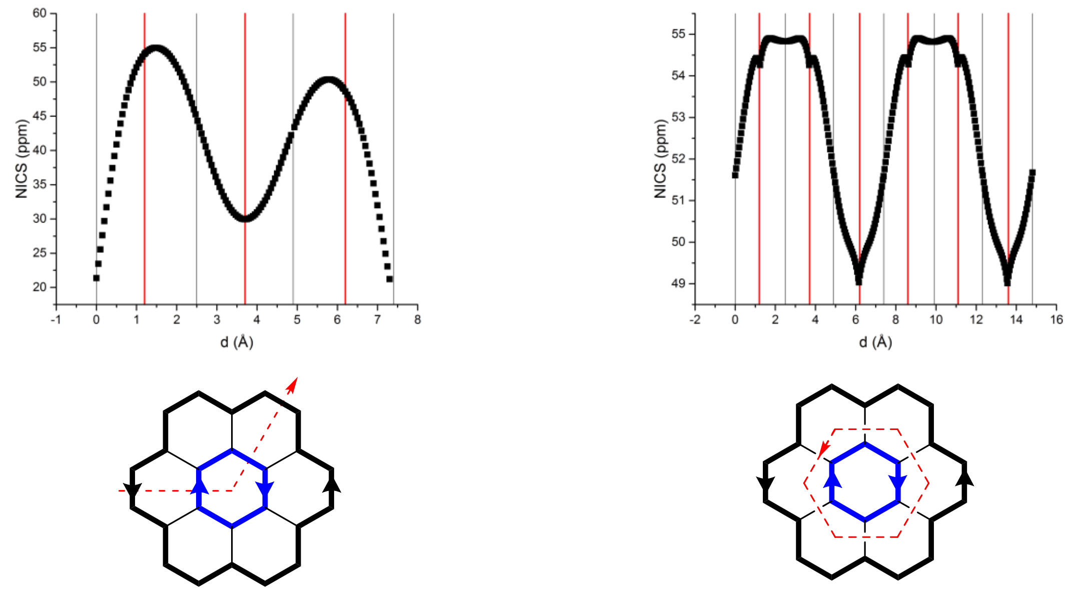

Supplementary Figure 124. NICS-XY scans for coronene in T_1 (D_{2h} symmetry). The interpretation of a central diatropic (aromatic) current is discussed further in the text. Note that the scale of the right-hand figure spans from 49-55 ppm, which are all significantly paratropic values.

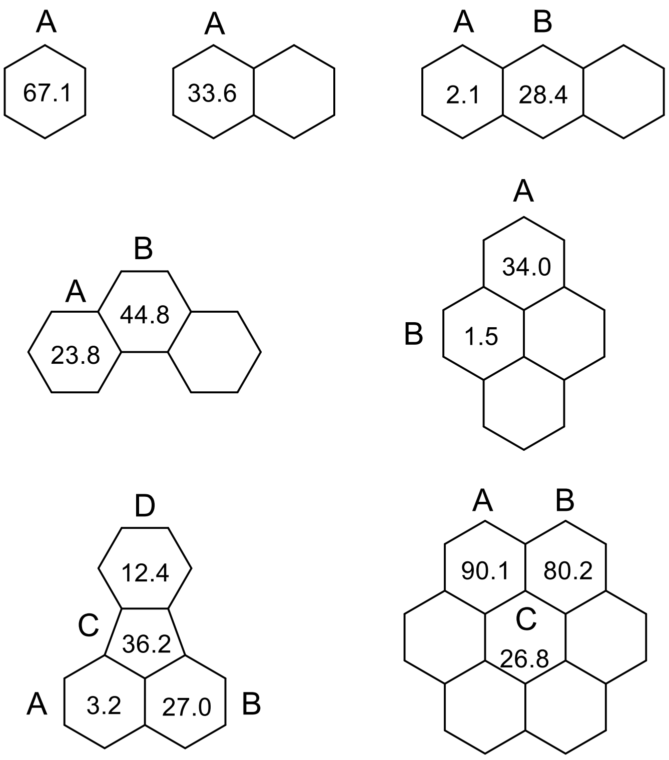

Supplementary Figure 125. NICS(1)$_{zz}$ values at the GIAO-B3LYP/6-311+G(d,p) level for triplet PAHs. Symmetry-unique rings are indicated by capital letters.

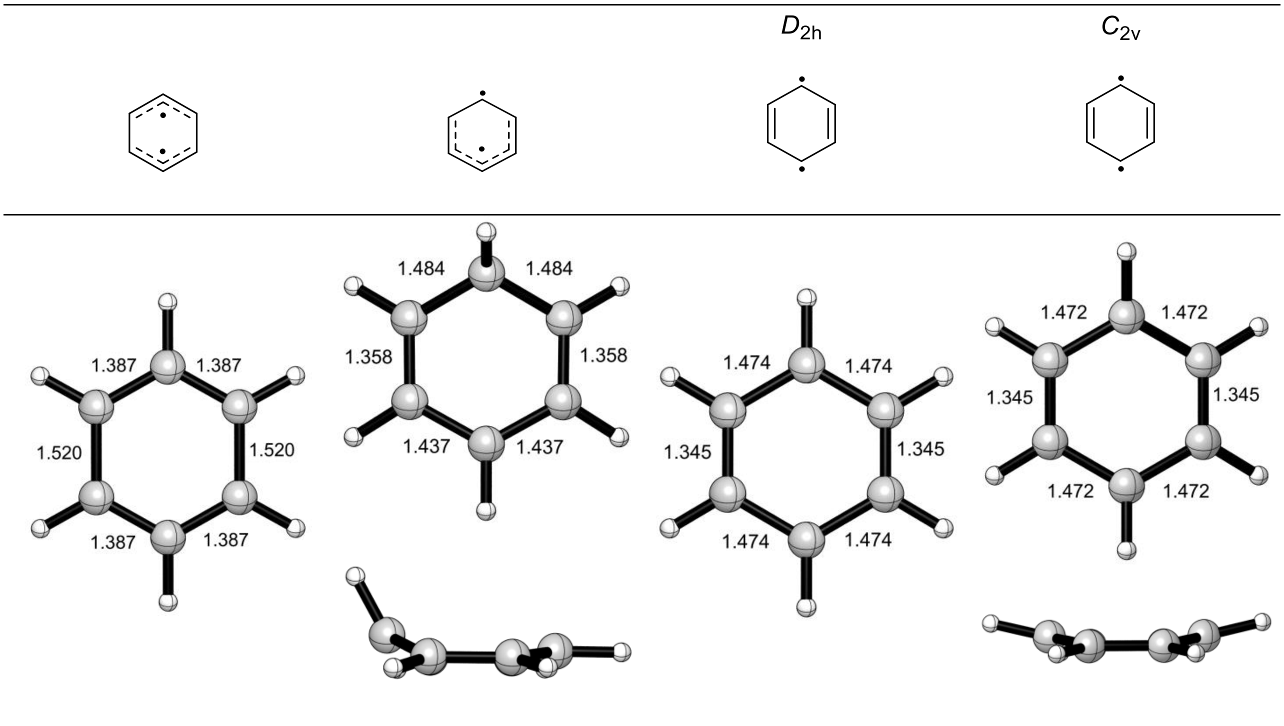

Supplementary Figure 126. Geometries of DA, MP, Q_{D2h} and Q_{C2v} at the B3LYP/6-311+G(d,p) level.

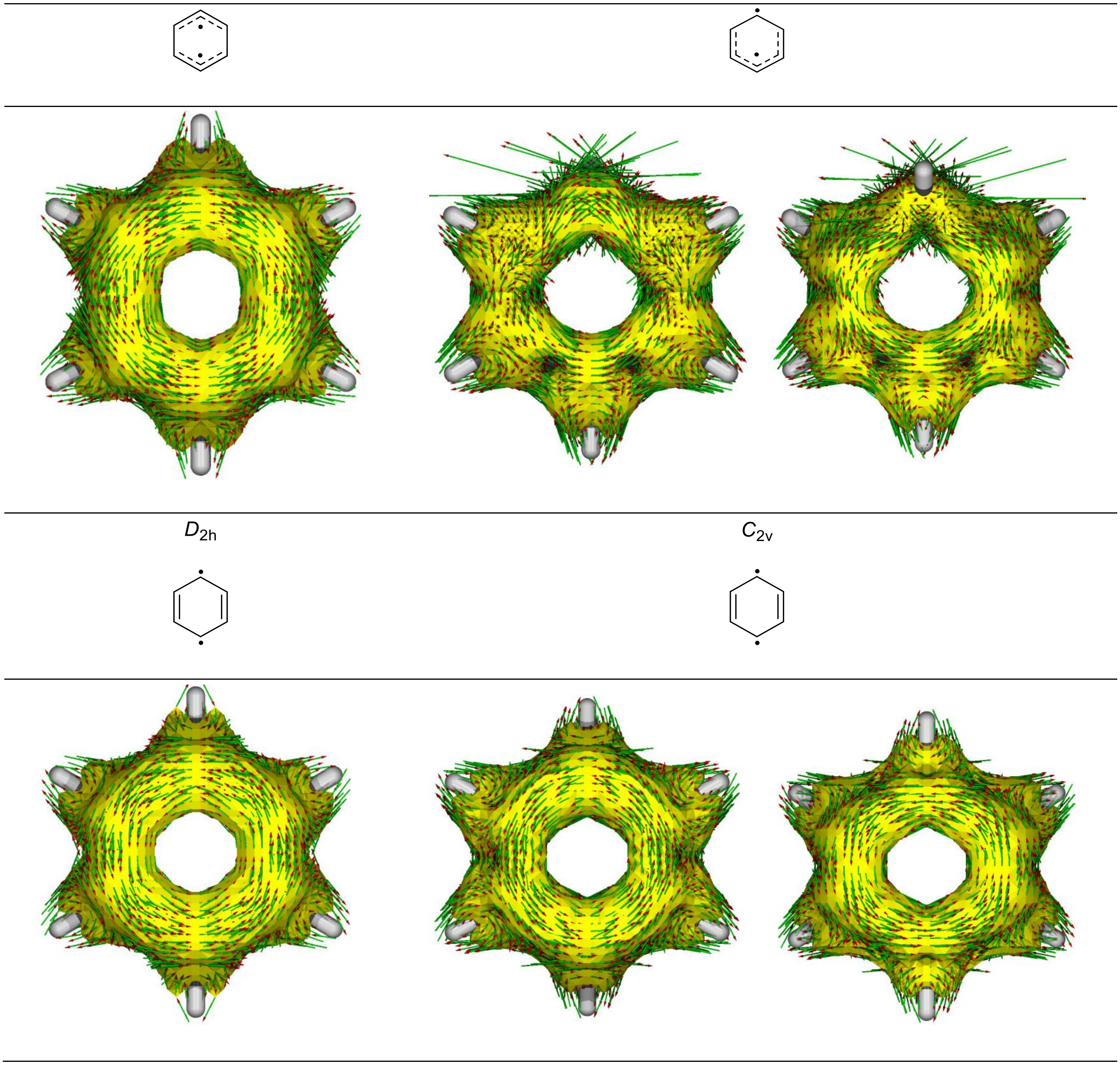

Supplementary Figure 127. ACID plots of DA, MP, Q_{D2h} and Q_{C2v} at the CSGT-B3LYP/6-311+G(d,p) level.

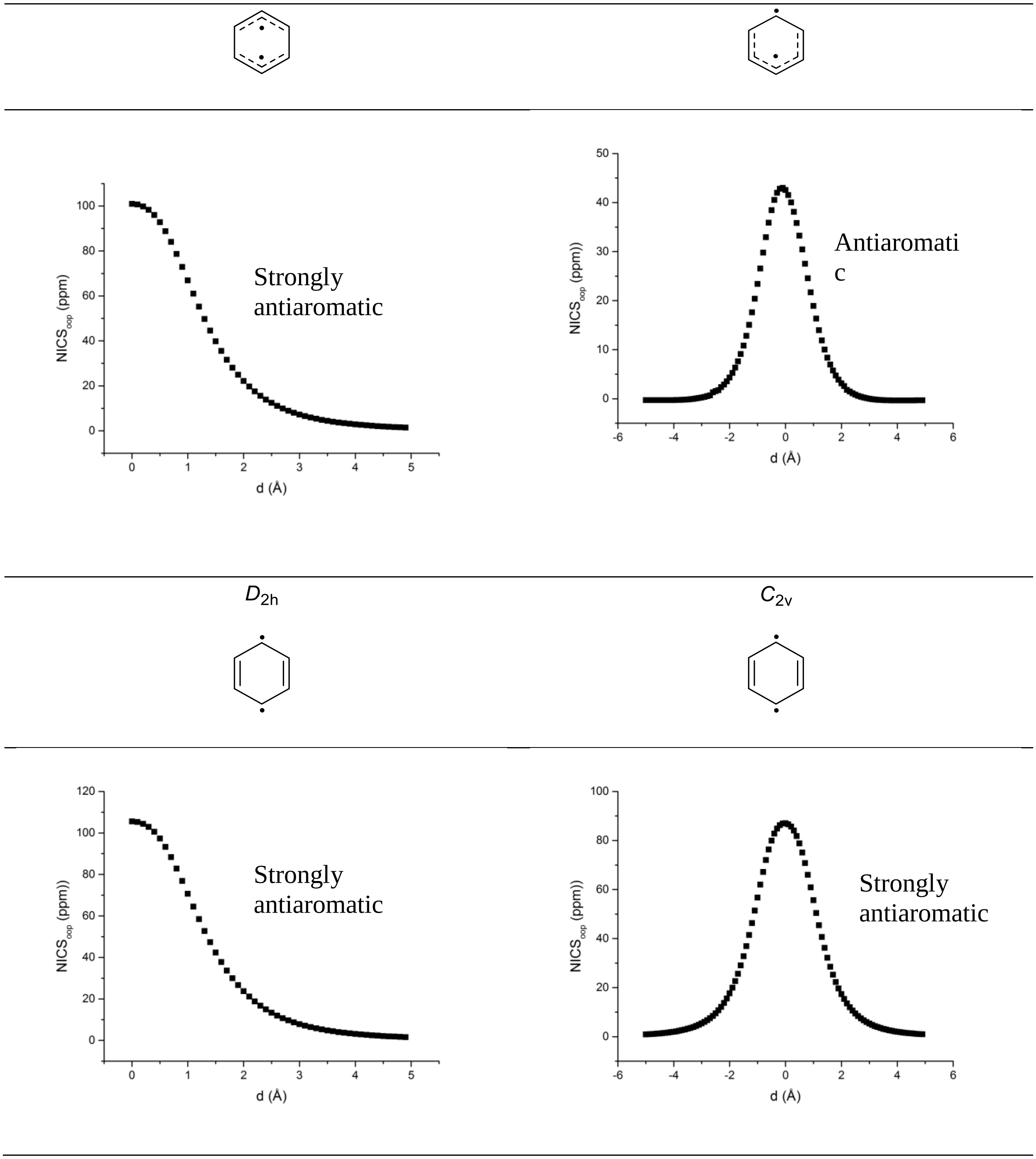

Supplementary Figure 128. NICS scans of DA, MP, Q_{D2h} and Q_{C2v} at the GIAO-B3LYP/6-311+G(d,p) level.

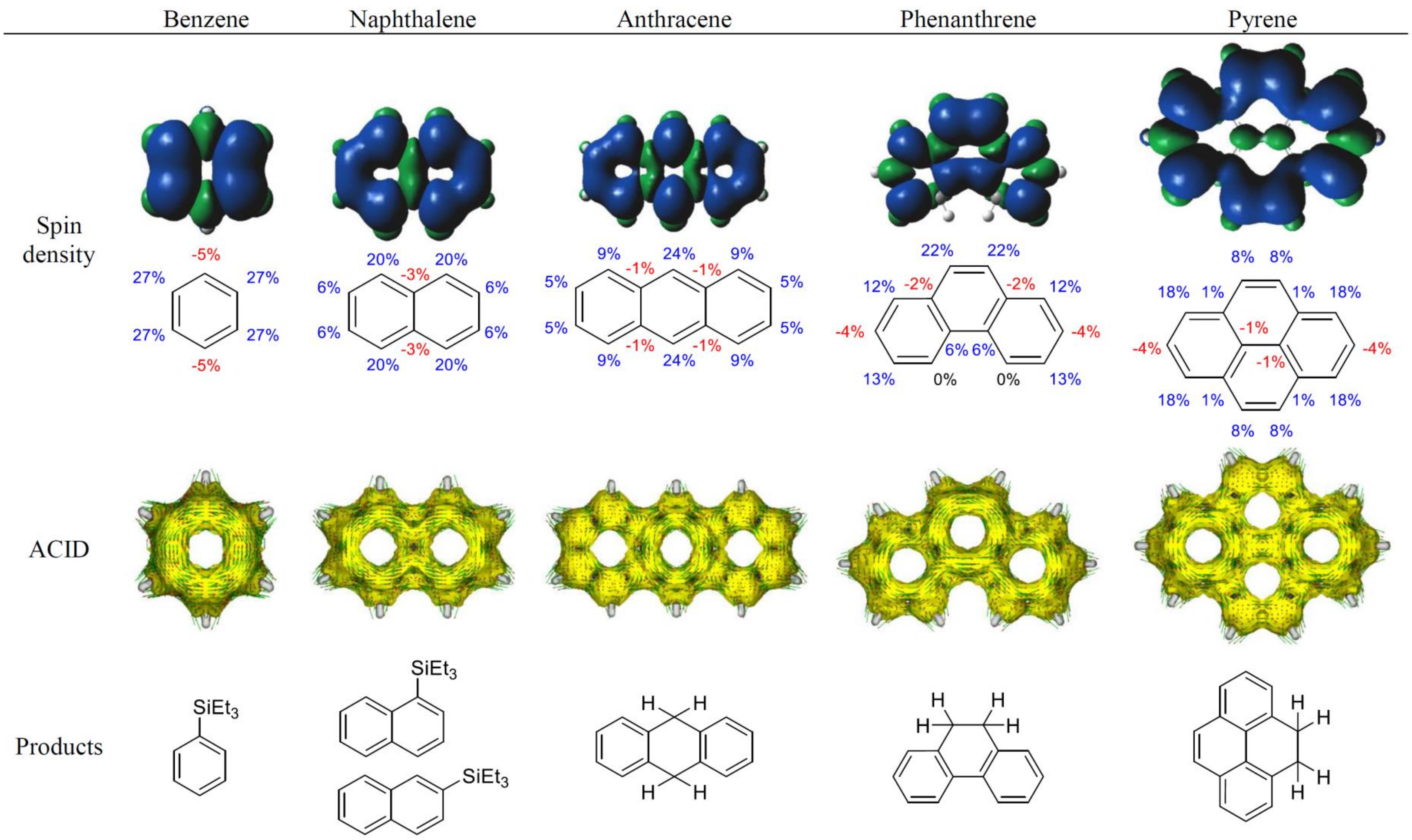

Supplementary Figure 129. Spin densities, ACID plots and observed hydrogentation and hydrosilylation products for benzene, naphthalene, anthracene, phenanthrene and pyrene. Calculations at the B3LYP/6-311+G(d,p) level. ACID plots as 0.050 isosurface value. Spin densities at 0.0004 isosurface values. Spin density integrated over AIM basins to acquire atomic spin densities.

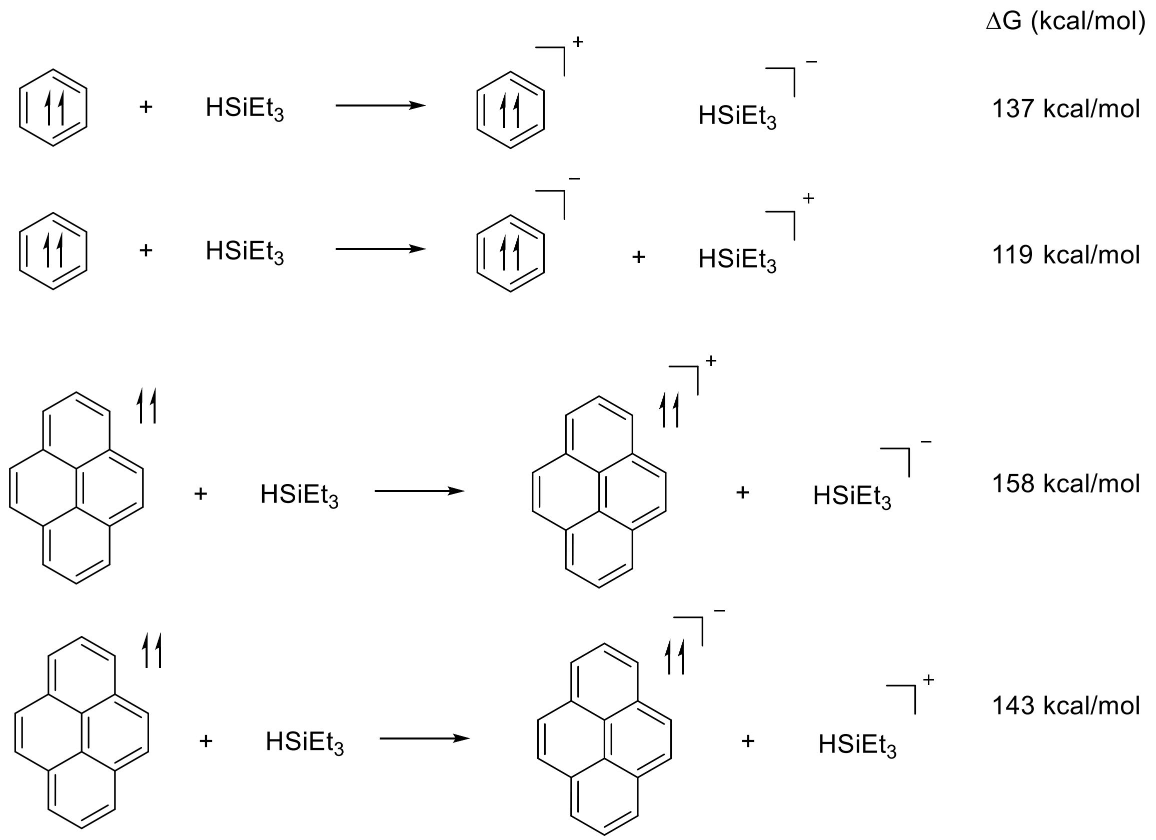

Supplementary Figure 130. Electron transfer energies for triplet benzene and pyrene at the G4 level.

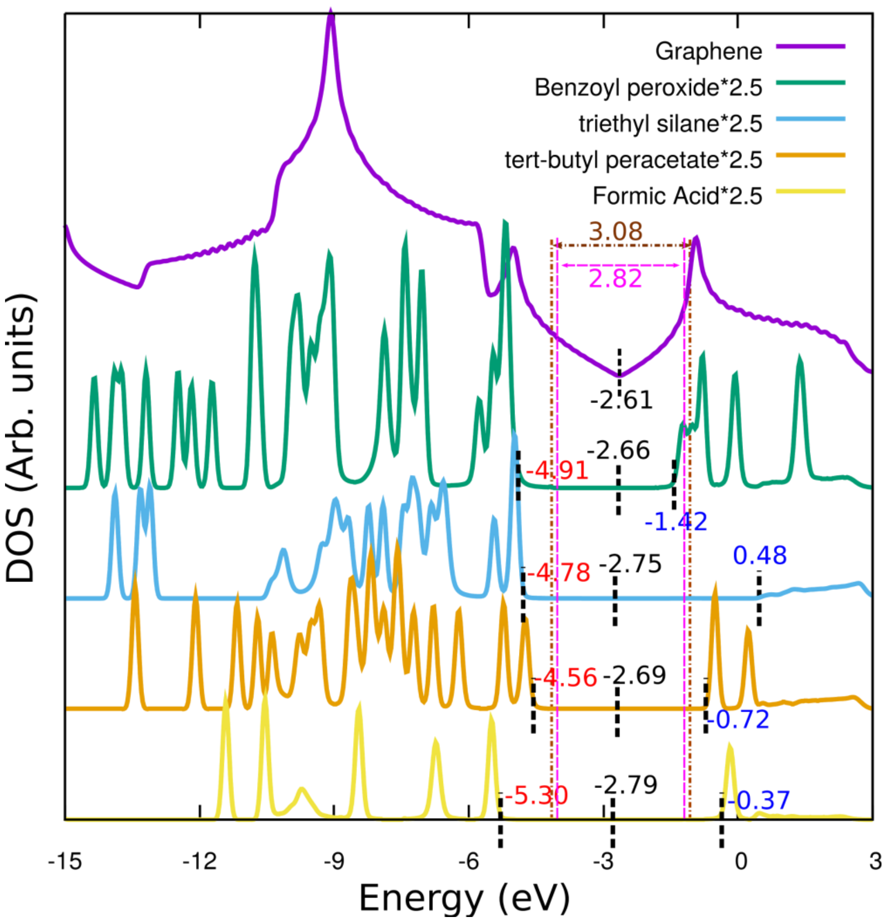

Supplementary Figure 131. Densities of states for benzoyl peroxide, *tert*-butyl peracetate, triethylsilane and formic acid physisorbed on graphene. Site projected densities of states (PDOS) for the molecules and the graphene substrate are separately shown. For a better representation, the PDOS for the molecules have been multiplied by a factor of 2.5. Red, black and blue numbers in the PDOS of the molecules indicate HOMO of the molecule, the Fermi level, and the LUMO of the molecule, respectively, for the molecule physisorbed on graphene. The position of graphene Dirac cone is separately shown (at 2.61 eV). Two vertical lines around the graphene Dirac cone were drawn to show the energy of the light used to irradiate graphene in the experiment.

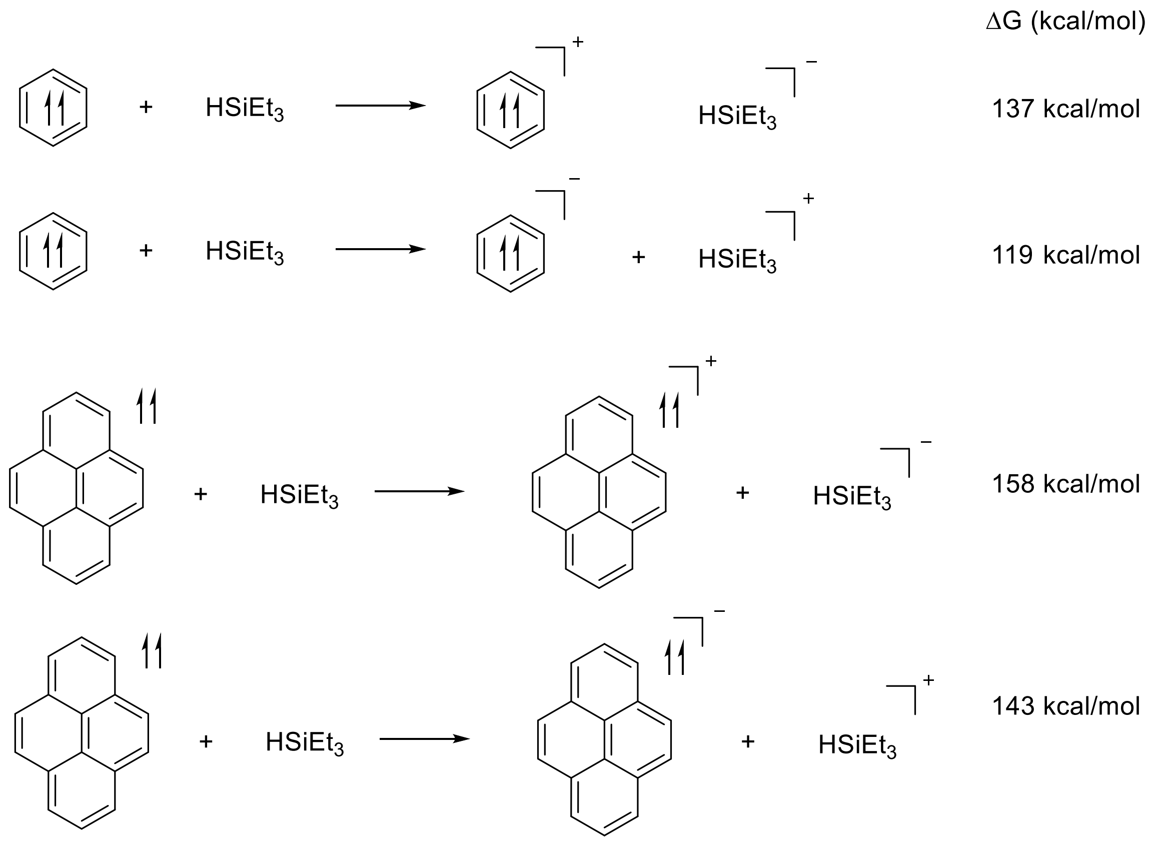

Supplementary Figure 132. Electron transfer energies for triplet benzene and pyrene at the G4 level.

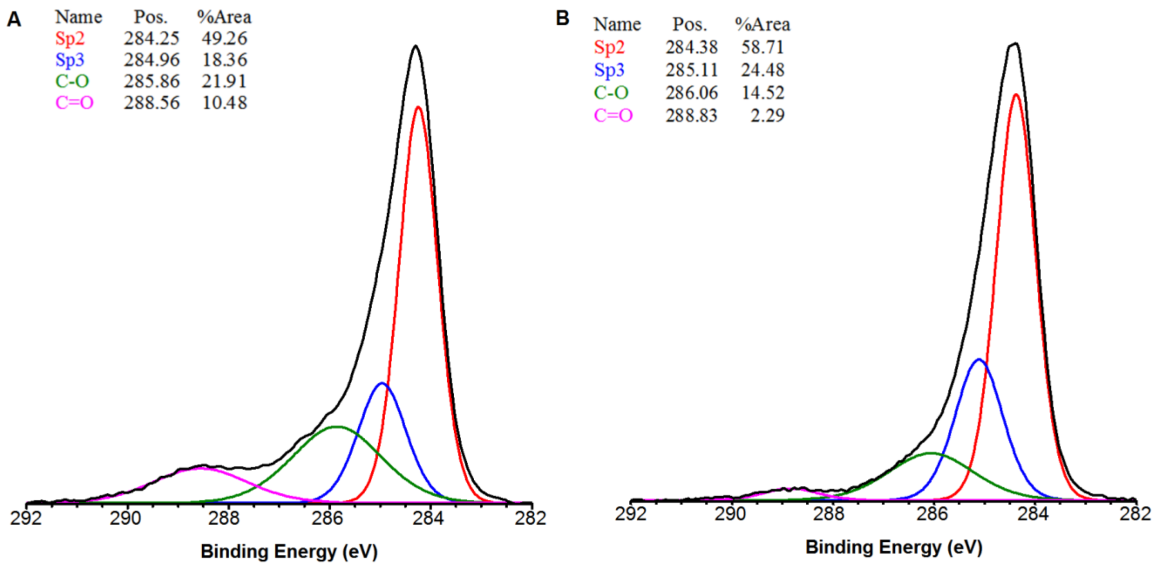

Supplementary Figure 133. High resolution C1s XPS after 24 h irradiation of a CVD grown graphene sample in 5 mM benzoyl peroxide solution in acetone (A) and in 50 % wt. *tert*-butylperacetate solution in mineral oil (B). Both experiments were conducted in a white LED photoreactor.

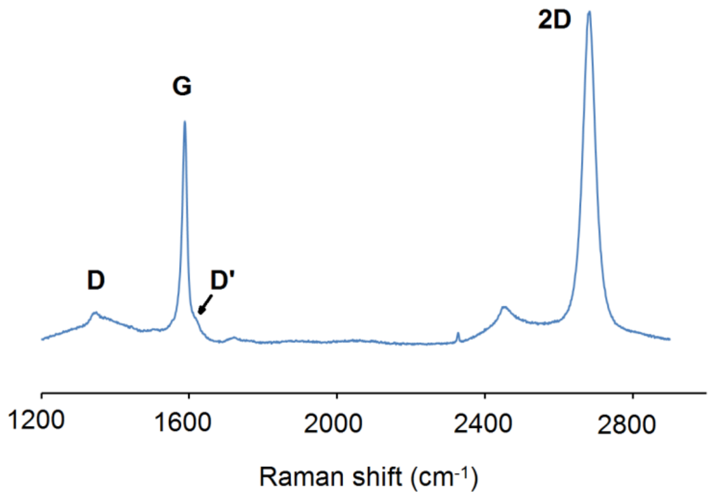

Supplementary Figure 134. Raman scattering spectrum obtained after 24 h irradiation of a CVD grown graphene smaple in 5 mM benzoyl peroxide solution in acetone.

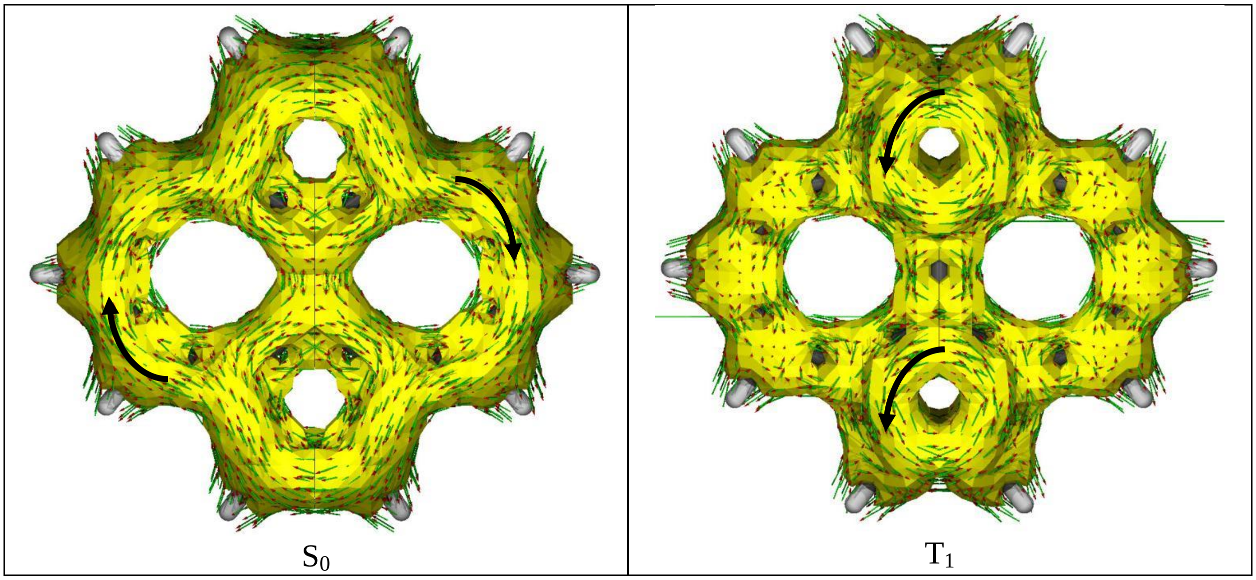

Supplementary Figure 135. ACID plots of azupyrene (a Stone-Wales defect model) in S_0 and T_1 at the CSGT-B3LYP/6-311+G(d,p)//B3LYP/6-311G(d,p) level.

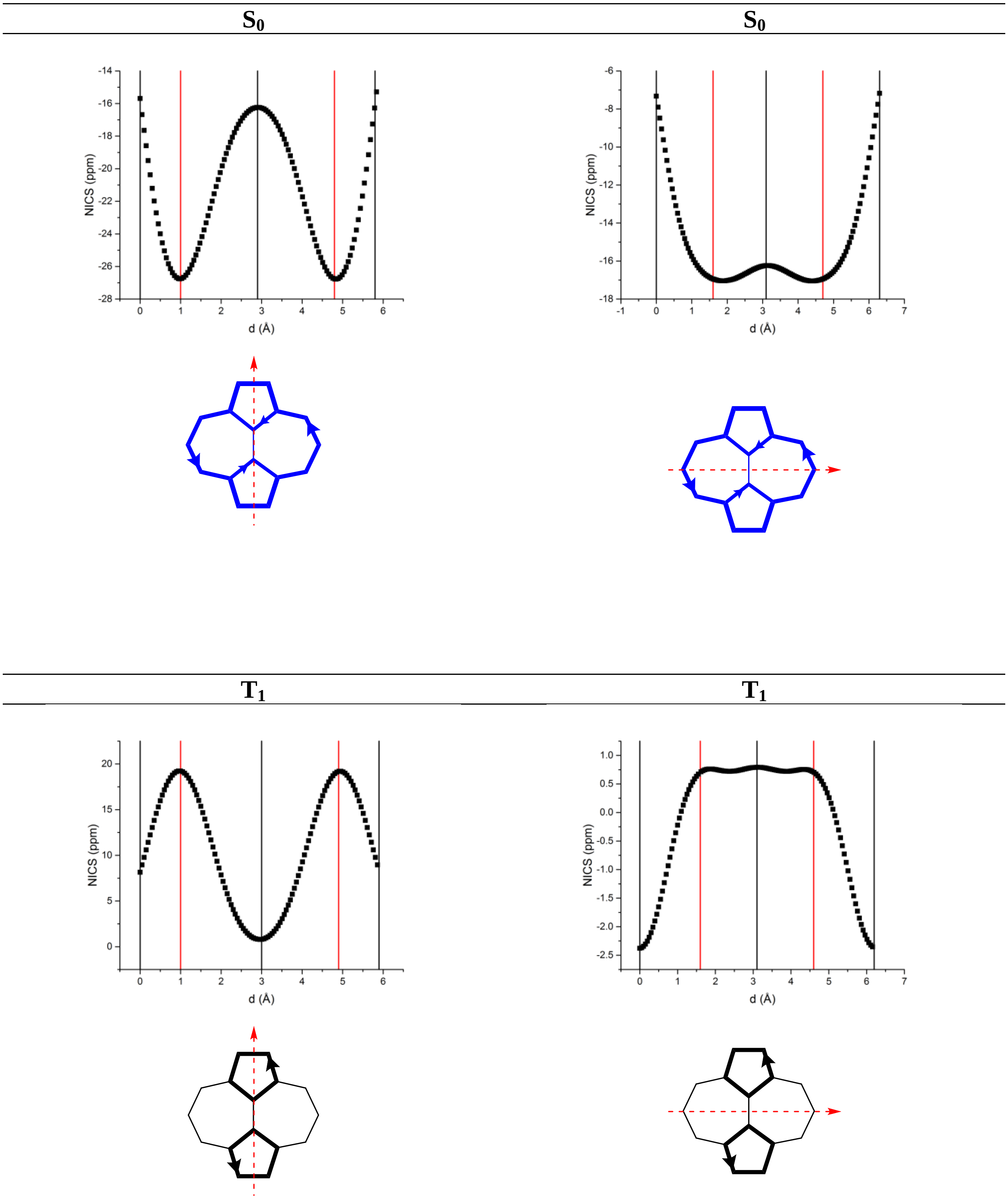

Supplementary Figure 136. NICS-XY scans of azupyrene (a Stone-Wales defect model) in S_0 and T_1 at the GIAO-B3LYP/6-311+G(d,p)//B3LYP/6-311G(d,p) level. Black lines indicate ring borders or center of bonds and red lines ring centers along the indicated scan direction.

Supplementary Tables

Supplementary Table 1: Intersystem crossing quantum yields (Φ_{ISC}) for benzene and PAHs used in this work.

Compound	Φ_{ISC}
Benzene	0.25[3]
Naphthalene	0.75[4]
Anthracene	0.71[4]
Phenanthrene	0.73[4]
Pyrene	0.37[4]
Fluoranthene	-
Coronene	0.53[5]

Supplementary Table 2: Results of all photoreactions of benzene and PAHs.

Compound	Hydrogenation yield	(Hydro)silylation yield
Benzene	Not applicable	6.0%
Naphthalene	Not applicable	21.0%
Anthracene	4.5%	4.5%
Phenanthrene	3.5%	11.5%
Pyrene	2.0%	0
Fluoranthene	0	0
Coronene	0	0

Supplementary Table 3. Hydrogenation energies for compounds with 4 π-electrons calculated at the (U)B3LYP/6-311+G(d,p) level.

	S_0				T_1			
**Entry**	**E[a]**	**G[a]**	**ΔE[b]**	**ΔG[b]**	**E[a]**	**G[a]**	**ΔE[b]**	**ΔG[b]**
A	-154.721160	-154.684259	-	-	-154.711758	-154.677334	-	-
A-H	-156.017234	-155.956831	-73.1	-57.5	-155.904042	-155.848464	-8.0	6.2
B	-193.197378	-193.144951	-	-	-193.214069	-193.161262	-	-
B-H	-194.488871	-194.412303	-70.2	-54.2	-194.380579	-194.311738	8.2	19.2
**Average**			**-71.7**	**-55.8**			**0.1**	**12.7**
Std. dev.			1.4	1.6			8.1	6.5
Range			2.9	3.3			16.2	13.0

a. In a. u.
b. In kcal/mol

Supplementary Table 4. Hydrogenation energies for compounds with 6 π-electrons calculated at the (U)B3LYP/6-311+G(d,p) level.

Entry	S_0 E[a]	G[a]	ΔE[b]	ΔG[b]	T_1 E[a]	G[a]	ΔE[b]	ΔG[b]
C	-193.580779	-193.528967	-	-	-193.469048	-193.420982	-	-
C-H	-194.740760	-194.667914	12.3	26.4	-194.694171	-194.621883	-28.6	-12.5
D	-232.311246	-232.237259	-	-	-232.169920	-232.107364	-	-
D-H	-233.483899	-233.390312	4.3	17.5	-233.403770	-233.316644	-34.1	-17.7
E	-270.740284	-270.650837	-	-	-270.617829	-270.537668	-	-
E-H	-271.920789	-271.811646	-0.6	12.7	-271.859854	-271.755563	-39.2	-23.2
**Average**			**5.3**	**18.9**			**-33.9**	**-17.8**
Std. dev.			5.3	5.7			4.3	4.4
Range			12.9	13.7			10.6	10.7

a. In a. u.
b. In kcal/mol

Supplementary Table 5. Hydrogenation energies for compounds with 8 π-electrons calculated at the (U)B3LYP/6-311+G(d,p) level.

Entry	S_0 E[a]	G[a]	ΔE[b]	ΔG[b]	T_1 E[a]	G[a]	ΔE[b]	ΔG[b]
F	-309.669047	-309.567493	-	-	-309.644368	-309.543850	-	-
F-H	-310.897187	-310.771900	-30.5	-14.7	-310.830428	-310.711526	-4.1	8.4
G	-308.447603	-308.367434	-	-	-348.067863	-347.954203	-	-
G-H	-309.680795	-309.577409	-33.9	-17.9	-349.281936	-349.145358	-21.7	-6.4
H	-348.101254	-347.983948	-	-	-308.433760	-308.355899	-	-
H-H	-349.334882	-349.193392	-33.6	-18.2	-309.616730	-309.518596	-2.1	11.5
**Average**			**-32.7**	**-16.9**			**-9.3**	**4.5**
Std. dev.			1.6	1.6			8.8	7.8
Range			3.4	3.5			19.5	17.9

a. In a. u.
b. In kcal/mol

Supplementary Table 6. Hydrogenation energies for compounds with 10 π-electrons calculated at the (U)B3LYP/6-311+G(d,p) level.

Entry	S_0				T_1			
**Entry**	**E[a]**	**G[a]**	**ΔE[b]**	**ΔG[b]**	**E[a]**	**G[a]**	**ΔE[b]**	**ΔG[b]**
I	-385.277313	-385.176891	-	-	-385.2077345	-385.113217	-	-
I-H1-*cis*	-386.488674	-386.367444	-19.9	-6.0	-386.4383972	-386.321116	-32.1	-16.9
I-H1-*trans*	-386.448596	-386.327088	5.2	19.3	-386.3994467	-386.281797	-7.6	7.8
I-H2	-386.460661	-386.342008	-2.4	10.0	-386.4357458	-386.318696	-30.4	-15.4
**Average**			**-5.7**	**7.8**			**-23.4**	**-8.2**
Std. dev.			10.5	10.5			11.2	11.3
Range			25.1	25.3			24.4	24.7
J	-424.014399	-423.894722	-	-	-423.9404705	-423.826653	-	-
J-H1	-425.208111	-425.066487	-8.9	5.8	-425.1583715	-425.021631	-24.1	-8.8
J-H2-*cis*	-425.214554	-425.072172	-12.9	2.2	-425.1565891	-425.019724	-22.9	-7.6
J-H2-*trans*	-425.201422	-425.059043	-4.7	10.5	-425.1492920	-425.011821	-18.4	-2.6
J-H3-*cis*	-425.228728	-425.086360	-21.8	-6.7	-425.1780129	-425.040395	-36.4	-20.5
J-H3-*trans*	-425.193142	-425.050526	0.5	15.8	-425.1417354	-425.003983	-13.6	2.3
J-H4	-425.212249	-425.071491	-11.5	2.7	-425.1701026	-425.033405	-31.4	-16.2
J-H5-*cis*	-425.206395	-425.064497	-7.8	7.0	-425.1638062	-425.026567	-27.5	-11.9
J-H5-*trans*	-425.199469	-425.056922	-3.5	11.8	-425.1471009	-425.009288	-17.0	-1.0
**Average**			**-8.8**	**6.1**			**-23.9**	**-8.3**
Std. dev.			6.4	6.5			7.2	7.3
Range			22.3	22.5			22.8	22.8
K1	-462.527082	-462.389234	-	-	-462.4434420	-462.312905	-	-
K2	-	-	-	-	-462.4422621	-462.310539	-	-
K-H1	-463.727444	-463.569689	-13.0	0.3	-463.6789498	-463.523939	-35.5	-19.6
K-H2-*cis*	-463.717564	-463.557870	-6.8	7.8	-463.6711026	-463.514872	-30.5	-13.9
K-H2-*trans*	-463.713399	-463.553250	-4.2	10.7	-463.6594001	-463.502974	-23.2	-6.4
K-H3	-463.719139	-463.560002	-7.8	6.4	-463.6704368	-463.515340	-30.1	-14.2
K-H4-*cis*	-463.708646	-463.548853	-1.3	13.4	-463.6682291	-463.513035	-28.7	-12.8
K-H4-*trans*	-463.698838	-463.539025	4.9	19.6	-463.6664253	-463.510478	-27.6	-11.1
K-H5	-463.711664	-463.552620	-3.1	11.0	-463.6747964	-463.519639	-32.9	-16.9
K-H6-*cis*	-463.702911	-463.543247	2.3	16.9	-463.6617400	-463.505645	-24.7	-8.1
K-H6-*trans*	-463.711406	-463.551435	-3.0	11.8	-463.6672550	-463.510987	-28.1	-11.5
**Average**			**-3.6**	**10.9**			**-29.0**	**-12.7**
Std. dev.			5.1	5.4			3.6	3.8
Range			18.0	19.2			12.3	13.2
**Total**								
**Average**			**-6.0**	**8.3**			**-25.4**	**-9.7**
Std. dev.			7.1	7.2			7.2	7.2
Range			27.0	26.3			28.8	28.3

a. In a. u.
b. In kcal/mol

Supplementary Table 7. Hydrogenation energies for compounds with 12 π-electrons calculated at the (U)B3LYP/6-311+G(d,p) level.

Entry	S_0 E[a]	S_0 G[a]	S_0 ΔE[b]	S_0 ΔG[b]	T_1 E[a]	T_1 G[a]	T_1 ΔE[b]	T_1 ΔG[b]
L1	-462.762639	-462.632520	-	-	-462.759628	-462.630231	-	-
L2	-462.759929	-462.629881	-	-	-	-	-	-
L-H1	-463.985977	-463.833276	-28.3	-13.2	-463.937836	-463.788842	0.9	14.0
L-H2-*cis*	-463.981522	-463.826775	-25.5	-9.1	-463.935575	-463.785271	2.3	16.3
L-H2-*trans*	-463.972827	-463.818189	-20.1	-3.8	-463.935006	-463.783971	2.6	17.1
L-H3	-463.978079	-463.824374	-23.4	-7.6	-463.934693	-463.784456	2.8	16.8
L-H4-*cis*	-463.979294	-463.825172	-24.1	-8.1	-463.939586	-463.788854	-0.2	14.0
L-H4-*trans*	-463.978890	-463.824444	-23.9	-7.7	-463.934915	-463.784256	2.7	16.9
L-H5	-463.980400	-463.826625	-24.8	-9.1	-463.933370	-463.783310	3.7	17.5
L-H6-*cis*	-463.968670	-463.814330	-17.5	-1.3	-463.925667	-463.775164	8.5	22.6
L-H6-*trans*	-463.977581	-463.823253	-23.0	-6.9	-463.934539	-463.784126	2.9	17.0
**Average**			**-23.4**	**-7.4**			**2.9**	**16.9**
Std. dev.			2.9	3.2			2.3	2.4
Range			10.9	11.9			8.7	8.6
M	-501.460644	-501.308107	-	-	-501.451442	-501.301264	-	-
M-H1-*cis*	-502.671476	-502.496376	-19.6	-4.6	-502.629837	-502.459477	0.7	14.3
M-H1-*trans*	-502.679362	-502.504385	-24.6	-9.6	-502.639866	-502.469326	-5.6	8.1
M-H2	-502.682362	-502.507539	-26.4	-11.6	-502.640150	-502.470021	-5.7	7.7
**Average**			**-23.5**	**-8.6**			**-3.5**	**10.0**
Std. dev.			2.9	2.9			3.0	3.0
Range			6.8	7.0			6.5	6.6
N	-539.927328	-539.760368	-	-	-539.921559	-539.754614		
N-H1-*cis*	-541.145882	-540.953128	-24.5	-7.4	-541.105261	-540.917491	-2.6	11.4
N-H1-*trans*	-541.146386	-540.954349	-24.8	-8.1	-541.113361	-540.925588	-7.7	6.3
N-H2	-541.148982	-540.957114	-26.4	-9.9	-541.114052	-540.926523	-8.1	5.7
N-H3-*cis*	-541.136621	-540.944604	-18.7	-2.0	-541.098972	-540.910234	1.4	15.9
N-H3-*trans*	-541.148513	-540.956918	-26.1	-9.8	-541.112457	-540.924069	-7.1	7.2
N-H4	-541.153167	-540.961123	-29.0	-12.4	-541.115182	-540.926553	-8.8	5.7
N-H5	-541.159613	-540.968190	-33.1	-16.8	-541.113924	-540.925905	-8.0	6.1
N-H6	-541.147393	-540.955184	-25.4	-8.7	-541.112031	-540.924151	-6.8	7.2
N-H7-*cis*	-541.141037	-540.948346	-21.4	-4.4	-541.104941	-540.916393	-2.4	12.1
P-H7-*trans*	-541.139677	-540.947648	-20.6	-3.9	-541.107995	-540.919552	-4.3	10.1
**Average**			**-25.0**	**-8.3**			**-5.5**	**8.8**
Std. dev.			4.0	4.1			3.2	3.3
Range			14.4	14.8			10.2	10.2
**Total**								
**Average**			**-24.0**	**-8.1**			**-2.0**	**11.9**
Std. dev.			3.5	3.6			4.8	4.9
Range			15.6	15.5			17.3	16.9

a. In a. u.
b. In kcal/mol

Supplementary Table 8. Relative energies of DA, MP, Q_{D2h} and Q_{C2v} at various levels of theory.

	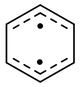	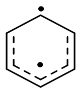	D_{2h} 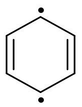	C_{2v} 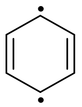
**B3LYP/6-311+G(d,p)**				
E_{rel} (kcal/mol)	0	-0.6	0.5	0.3
G_{rel} (kcal/mol)	0	0.5	0.3	-0.3
# imaginary freq.	0	0	1	1
**M06-2X/6-311+G(d,p)**				
E_{rel} (kcal/mol)	0	-3.3	-1.8	-2.2
G_{rel} (kcal/mol)	0	-0.8	0.4	-0.4
# imaginary freq.	1	0	1	1
**B2PLYP/6-311+G(d,p)**				
E_{rel} (kcal/mol)	0	-2.1	-1.5	-1.9
G_{rel} (kcal/mol)	0	0.4	4.5	1.1
# imaginary freq.	1	0	1	0
**G4**				
E_{rel} (kcal/mol)[a]	0	-0.9	-0.3	-0.4
G_{rel} (kcal/mol)	0	0.3	-0.4	-1.1
# imaginary freq.[b]	1	0	3	2
**CCSD(T)/cc-pVTZ**[c]				
E_{rel} (kcal/mol)	0	-0.8	-1.0	-1.1
G_{rel} (kcal/mol)	-	-	-	-
# imaginary freq.	-	-	-	-

[a] The electronic energies are taken as the non-thermal contributions to the G4 energy. [b] Frequencies are obtained at the B3LYP/ 6-31G(2df,p) level in G4 theory. [c] Using B3LYP/6-311+G(d,p) geometries.

Supplementary Table 9. CASSCF/RASSCF weights for the two most important configurations of vertically excited and relaxed triplet states of selected PAHs.

	Vertical		Relaxed	
Compound	Weight of 1st conf.	Weight of 2nd conf.	Weight of 1st conf.	Weight of 2nd conf.
Benzene	0.43	0.43	0.81	0.06
Naphthalene	0.64	0.09	0.74	0.05
Anthracene	0.64	0.04	0.69	0.03
Phenanthrene	0.44	0.18	0.64	0.03
Pyrene	0.72	0.03	0.76	0.01

Supplementary Table 10. Calculated energies of different Et_3SiH cis- and trans-adducts to graphene.

	**Same side 1**	**Same side 2**	**Same side 3**	**Lowest energy structure opposite side**
**Total energy (eV)**	-1028.09	-1028.28	-1027.77	-1028.69
**Relative energy difference considering lowest energy structure as reference (eV)**	0.60	0.40	0.92	0.0
**Relative energy difference considering lowest energy structure as reference (kcal/mol)**	13.8	9.2	21.2	0.0

Supplementary Table 11. Calculated structures of different Et_3SiH cis- and trans-adducts to graphene.

Same side 1	Same side 2	Same side 3	Opposite sides
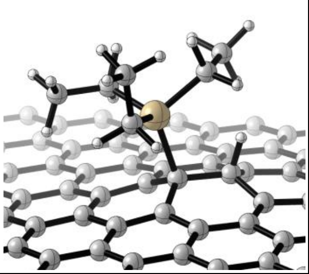	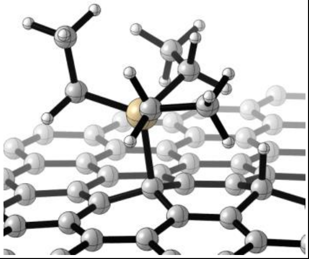	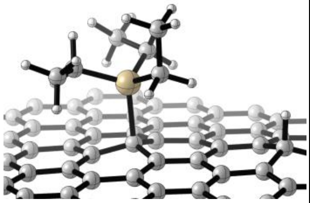	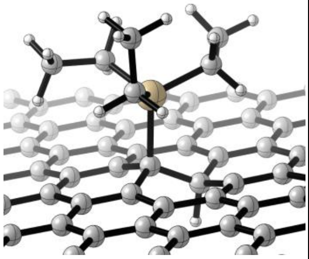
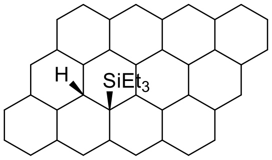	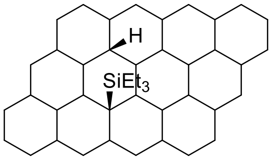	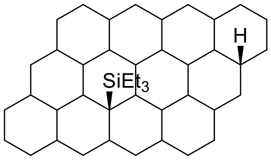	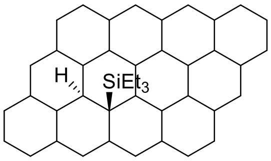

Supplementary Table 12. Cartesian coordinates for unit cell of hydrosilylated graphene

Atom	x	y	z
H	10.736481	6.947417	14.739334
H	9.974707	6.568980	13.203250
H	12.091136	4.797443	14.623125
H	10.340625	4.486439	14.596451
H	11.288003	4.378081	13.101595
H	14.776239	7.397853	13.215528
H	14.104786	5.776783	13.358744
H	13.162354	6.398717	15.671905
H	14.932789	6.536666	15.603864
H	13.923510	7.993953	15.521306
H	12.678150	9.366768	14.347573
H	12.547747	9.898132	12.681818
H	10.179041	9.149753	14.531356
H	10.632738	10.786983	14.011341
H	9.991179	9.625456	12.833254
H	12.341074	8.109364	9.080312
C	8.638976	14.845135	10.549108
C	1.259346	0.639682	10.523344
C	11.098730	14.840256	10.491590
C	3.719068	0.639670	10.488664
C	13.559631	14.840405	10.488939
C	6.178868	0.639877	10.514207
C	16.019405	14.845443	10.542567
C	8.639353	0.645023	10.573702
C	18.479633	14.850533	10.603755
C	11.098389	0.650534	10.632144
C	20.938917	14.851169	10.639446
C	13.559460	0.650333	10.638748
C	23.398556	14.850167	10.610902
C	16.018734	0.644758	10.585743
C	1.260384	2.060517	10.530415
C	2.489064	2.770429	10.510275
C	3.718933	2.058087	10.497778
C	4.948629	2.770462	10.499706
C	6.177609	2.060630	10.513598
C	7.406542	2.772236	10.531994
C	8.637788	2.064027	10.566011
C	9.866927	2.776395	10.594789

Atom	x	y	z
C	11.096586	2.070406	10.638118
C	12.328438	2.787802	10.667023
C	13.560728	2.070097	10.649832
C	14.790539	2.776014	10.620063
C	16.020039	2.063859	10.588901
C	17.251278	2.772045	10.558208
C	2.489525	4.190894	10.512701
C	3.718582	4.901948	10.505171
C	4.947760	4.190969	10.499790
C	6.177335	4.901306	10.501512
C	7.404251	4.191282	10.519219
C	8.635479	4.903187	10.529621
C	9.863785	4.197270	10.576941
C	11.089827	4.914817	10.645252
C	12.328115	4.201005	10.689602
C	13.565779	4.914309	10.674459
C	14.793182	4.197083	10.614480
C	16.021482	4.903175	10.569695
C	17.253078	4.191193	10.552315
C	18.479912	4.901154	10.527576
C	3.718526	6.323056	10.518789
C	4.948367	7.034265	10.519917
C	6.179221	6.324050	10.502935
C	7.407375	7.032799	10.497854
C	8.637579	6.321006	10.497012
C	9.871916	7.024336	10.514340
C	11.077662	6.323466	10.658092
C	12.323618	7.084504	10.948289
C	13.576224	6.323730	10.681332
C	14.783543	7.023704	10.549100
C	16.018547	6.320874	10.537610
C	17.249130	7.032473	10.535455
C	18.477791	6.323878	10.530737
C	19.708721	7.034164	10.534446
C	4.948170	8.454782	10.542997
C	6.178452	9.165222	10.545506

Atom	x	y	z
C	7.406260	8.456124	10.522319
C	8.634180	9.169195	10.510612
C	9.870110	8.466556	10.478515
C	11.070215	9.173192	10.379041
C	12.331150	8.394409	10.164832
C	13.589135	9.172835	10.400388
C	14.787512	8.466561	10.515028
C	16.023424	9.169314	10.549235
C	17.251225	8.455991	10.557858
C	18.479475	9.165219	10.567905
C	19.709650	8.454741	10.555609
C	20.939024	9.165827	10.562861
C	6.177731	10.588083	10.566752
C	7.403849	11.298764	10.549219
C	8.635304	10.587022	10.509714
C	9.863394	11.294605	10.470057
C	11.089714	10.577269	10.391833
C	12.329494	11.291917	10.380738
C	13.569560	10.578144	10.405299
C	14.795439	11.295122	10.488476
C	16.023104	10.587115	10.536664
C	17.254602	11.298989	10.566350
C	18.480745	10.588180	10.582356
C	19.710459	11.298641	10.597612
C	20.939281	10.587175	10.588336
C	22.168011	11.298651	10.591872
C	7.406446	12.717482	10.563322
C	8.637607	13.426121	10.536786
C	9.867006	12.715175	10.490461
C	11.097034	13.421324	10.466731
C	12.329400	12.703151	10.432958
C	13.561529	13.421390	10.468955
C	14.791712	12.715574	10.498295
C	16.020977	13.426539	10.540257
C	17.252132	12.717864	10.569777

C	18.480886	13.429514	10.598745
C	19.709703	12.719316	10.611365
C	20.939186	13.432136	10.623897
C	22.168848	12.719333	10.609694
C	23.397697	13.429210	10.598360
C	10.908269	6.433872	13.775068
C	11.171387	4.943793	14.035750
C	13.948534	6.825243	13.665556
C	13.993571	6.943690	15.196966
C	12.070881	9.248684	13.430380
C	10.640303	9.725815	13.714540
Si	12.300045	7.423203	12.956519

Supplementary Methods

General

CVD Graphene: Monolayer graphene on 1 cm × 1 cm silicon wafers (p-doped) with a 285 nanometer silicon dioxide coating were purchased from Graphene Supermarket (https://graphene-supermarket.com/). The graphene coverage of the samples was about 95% (predominantly single-layer graphene: more than 97%). The purchased graphene was grown on Cu according to standard procedures,[6] and transferred using a PMMA assisted transfer method.[7,8] In our laboratory the samples were cut in smaller square samples (most commonly: 0.5 cm × 0.5 cm) depending on our experimental needs, and they were further purified with $CHCl_3$ (immersed for 24 h) prior to use. The quality of the samples was checked through Raman scattering, as well as through XPS in order to exclude the existence of residual Cu.

All aromatic compounds were purchased from SIGMA-Aldrich (purity > 97%) and they were used without any further purification. Benzene was dried over molecular sieves (4A), and anhydrous *n*-heptane (99 %) as well as pentane (used for column chromatography) was used without any further purification. Triethylhydrosilane (Et_3SiH) of 99 % purity was used for all photo(hydro)silylations. Formic acid (> 98 %) was used for all transfer photohydrogenations. Water was deionized.

Photochemical reactors and glassware

- An RPR-100 Rayonet Photochemical Chamber Reactor was used for the photoreactions of benzene and PAHs equipped with a merry-go-round unit with a capacity of 8 tubes. Two different sets of lamps were used depending on the experiment: either a set of UV-lamps: 2537 Å and a total power of 35 W (RPR-2537A, **Supplementary Figure 1**) or a set of 16 UV-lamps: 3000 Å (RPR-3000A) all purchased from Southern New England Ultraviolet Company (the intensity characteristics of both types of lamps are shown in **Supplementary Figures 2 and 3**). Photoreactions were performed in three different scales. Quartz NMR tubes (Ø: 0.5 cm, 17.5 cm long; *suprasil*, cut-off wavelength 190 nm) were used, for the small-scale photoreactions (up to 1 mL), 15 mL quartz cylindrical tubes (RQV-5: Rayonet; Ø 13 mm) were used for the medium-scale photoreactions, and finally for the large-scale photoreactions cylinders made of quartz with a 185 mL volume capacity (RQV-118: Rayonet; Ø 20 mm) were used. The distance between a sample and the lamps was 8.5 cm ≈ 3.3" (see **Supplementary Figure 3B**).
- For experiments at small scale (using quartz NMR tubes) a low pressure Hg UV-lamp (16 W, 2537 Å) was used with the samples (typically 10 tubes) placed in a rack at a distance of 2 cm from the lamp.

- Xe-lamp irradiation experiments were performed using a Fluorolog-3-22 instrument (Horiba Jobin Yvon) containing a 450 W Xe lamp as a light source. The instrument is equipped with a double-grating excitation monochromator. The slit width was set to 14 nm, smaller than the width of the absorption band used for irradiation. In contrast to Hg-lamps, Xe-lamps have a more uniform spectrum and the (double-grating) monochromator excludes transmission of wavelengths outside the range of the slit width (small amounts of second-order, double wavelength tranmitted light would not be absorbed by the sample). The samples were prepared in a glove box under Ar atmosphere with O2 levels <0.1 ppm. Taking the solubility of O_2 in *n*-heptane of x_1=20.5[9] and the molar concentration of heptane of 6.83 M, an O_2 concentration of at most 1.4 nM results. This is orders of magnitude smaller than the concentrations of the hydrocarbons used and also reduces the probability of diffusional collision with O_2 during the excited (triplet) state lifetime to virtually zero. The cuvette was sealed with a ground-glass joint with silicon grease, wrapped with many layers of Parafilm to ensure low O2-levels over a long period of time.
- Alternatively a FEP photoreactor was used for photoreactions at a 15 mL scale and λ=254 mn. The photoreactor used was home-made consisting of a water-cooled quartz immersion well externally coiled with FEP tubing (**Supplementary Figure 4B**) with an inner diameter of 0.8 mm and an outer diameter 1.6 mm (tubing wall-thickness 0.4 mm). The FEP coil was purchased by Bola (http://www.bola.de). For the irradiation at λ=254 mn a TNN 15/32 lamp (**Supplementary Figure 4A**) purchased from the Peschl Ultraviolet company was used placed in the cavity of the immersion well photoreactor (**Supplementary Figure 4B**). The lamp cut-off wavelength is 240 nm (lamp-emission spectrum given in Supplementary Figure C). The liquid sample was introduced into the FEP coil under argon and the photoreactions were carried in an atmosphere of argon.
- Graphene photohydrogenation and photo(hydro)silylation experiments were conducted in a home-made photoreactor made of a set of 120 white light-emitting diodes (LEDs) as shown in **Supplementary Figure 5** [Characteristics of the LEDs used: λ_{peak} = 451.0 nm; λ_{main} = 490.2 nm; $\lambda_{centroid}$ = 439.8 nm; λ_{center} = 441.0 nm; Band-width: 24.0 nm; Color temperature: 6149 K; (x,y) = (0.3196, 0.3317); (u,v) = (0.2016, 0.3139). Color purity 0.048; Light power: 357.1710 mW; Light Efficacy: 78.768 lm/W]. The distance between the sample and the LEDs is 3.75 cm (see **Supplementary Figure 5A**).

Check for 185 nm light contamination

In order to check the contamination of the 254 nm Hg lamps which we have used in this work we have performed the following control experiments:

- We have used Me_6Si_2 as a chemical probe of the 185 nm emission. Hexamethyldisilane has its first strong absorption at 186.9 nm[10] and it photodissociates into trimethylsilyl radicals as reported by Brix et al. when irradiated at 193 nm.[11] Thus, to test for 185 nm light-contamination from our 254 nm lamps we performed the following two experiments involving hexamethyldisilane.
  a) A 12 mM solution of hexamethyldisilane in benzene was irradiated in a Rayonet photoreactor at λ=254 nm for 48 h. If the 185 nm contamination is present, it should result in silyl radicals that would lead to the formation of silylated benzenes. Yet after 48 hours of irradiation no silylated benzenes were formed and hexamethyldisilane remained unreacted (see **Supplementary Figure 8**). This observation reveals that the 185 nm line is sufficiently blocked.
  b) A solution of hexamethyldisilane in dry cyclohexene (100 μL hexamethyldisilane in 900 μL cyclohexene, $[Si_2Me_6]$ = 0.542 M) was purged with Ar and then added in a quartz NMR tube sealed with a rubber septum, using a syringe. The tube was then placed in a Rayonet photoreactor and irradiated at λ = 254 nm for 24 h. After this period no precipitate or polymer was identified. 100 μL of the solution were diluted in 500 μL of $CDCl_3$ and the sample was analysed through ^{1}H-NMR spectroscopy showing only cyclohexene and unreacted hexamethyldisilane (see **Supplementary Figure 9**).
- We have performed the photohydrosilylation of benzene using an aqueous KCl solution as a filter of the light with λ<200 nm (aqueous KCl strongly absorbing light at λ = 185 nm). This experiment led to the same photoproduct i.e. $PhEt_3Si$ in about the same yield (6%) according to GC-MS as in case of non-filtered irradiation i.e. without using the aqueous KCl solution. The procedure was the following: A solution of triethylhydrosilane in dry benzene (0.5 mL, $[Et_3SiH]$ = 12 mM) was initially purged with Ar and then added in a degassed quartz tube sealed with a rubber septum, using a syringe. The tube was placed in a quartz tubes (RQV-5 Rayonet quartz tube; Ø 13 mm) containing a 10% (w/v) solution of KCl, strongly absorbing light between 180 at 200 nm) as shown in **Supplementary Figure 10**. The tube was then placed in a Rayonet photoreactor and irradiated at λ = 254 nm for 24 h. After the reaction the formation of a yellowish polymer on the surface of the tubes was observed. The solution was then used directly for GC-MS. Different products were identified with MS, with Et_3SiPh (MW=192 g/mol) as the major product in solution in ~6% GC-MS conversion as shown in the GC-MS of

Supplementary Figure 11. The use of the filter did not affect the outcome of the photo(hydro)silylation of benzene.

- Moreover, we have successfully photosilylated naphthalene using a TNN 15/32 lamp from the Peschl Ultraviolet company with a cut-off at 240 nm and shorter (lamp-emission given in **Supplementary Figure 12**). Under these conditions a 29 mM *n*-heptane solution of naphthalene in a 50-fold excess of Et_3SiH resulted in a 1:1 mixture of α- and β-triethylsilylnaphthalene in a combined isolated yield of 21% (for details on the setup used see section **Photochemical reactors and glassware**).
- Additionally we have obtained similar results to those obtained using a Rayonet photoreactor at λ=254 nm when we used monochromatic light emitted by a Xe UV-lamp (details are found in section: **photochemical reactors and glassware**). Specifically we have:
  a) photosilylated benzene under monochromatic irradiation at 254 nm and
  b) photosilylated phenanthrene under monochromatic irradiation at 293 nm.

All the above control experiments prove that the lamps used in this work are not contaminated with emission at λ= 185 nm.

Check for the presence of ozone

In order to check the presence of ozone in our irradiation experiments we have performed the following control experiments:

- We attempted attempted photosilylation using benzene plus triethylsilane in 1:1 ratio in *n*-heptane in the presence of ozone (formed by an ozone generator), none of the products formed corresponded to phenyltriethylsilane or triethylsilylcyclohexadiene, and also no polymer was formed. According to GC-MS and ^{1}H-NMR spectroscopy various hydroxylated and carbonylated products were identified (**Supplementary Figures 13 and 14**). This clearly shows that ozone is not present in our experiments. The procedure followed for this experiments was the following:

In a quartz tube containing 20 mL of an *n*-heptane solution of benzene and Et_3SiH in equimolar quantities ([Et_3SiH] = [benzene] =10 mM) was bubbled ozone continuously. The ozone was formed through an ozone generator (Pilodist OGF 505) with a flow rate of 0.5 L/min. The sample was simultaneously irradiated with a low pressure Hg UV-lamp at λ=254 nm for 2h. After this period the sample was analyzed by GC-MS and ^{1}H-NMR spectroscopy (see **Supplementary Figures 13 and 14** respectively). The ^{1}H-NMR spectrum showed various peaks close to 10 and 11 ppm indicating the formation of aldehydes and acids as well as various peaks in the region 3-5ppm.

- We furthermore irradiated cyclohexene while dissolved in *n*-heptane (cyclohexene concentration was 0.987 M) in an open quartz tube. The irradiation experiment was conducted in a Rayonet photoreactor at λ= 254 nm. If ozone is produced during irradiation then ozonolysis of cyclohexene should be observed. However, according to H-NMR cyclohexene was recovered unchanged (**Supplementary Figure 15**). The procedure followed was the following:

A solution of cyclohexene in n-heptane (150 μL cyclohexene in 1 mL n-heptane, [cyclohexene] = 0.987 M) was added in a quartz NMR tube. The tube left intentionally open and was placed in a Rayonet photoreactor where it was irradiated at λ = 254 nm for 24 h. After this period no precipitate or polymer was identified. 100 μL of the solution were diluted in 500 μL of CDCl3 and the sample was analysed through 1H-NMR spectroscopy showing only cyclohexene and *n*-heptane signals.

Gas chromatography - Mass spectrometry (GC-MS)

GC-MS were performed using an Agilent 5975C inert MSD system equipped with a Triple-Axis Detector. Samples were introduced using split-injection (2μL injection volume; Split Ratio: 100:1; 250 ^{o}C inlet temperature; Flow Rate: 120 mL/min). The starting temperature of the column oven was 70 ^{o}C (0.5 min equilibration time) and the ending temperature was 320 ^{o}C. The temperature rate was set to 20 ^{o}C/min resulting in a 12.5 min total run time. Helium was used as a carrier gas at a flow rate of 1.2 mL/min. The column used was an Aligent 19091S-433: 325 ^{o}C: 30 m × 250 μm × 0.25 μm (front SS-inlet: He; out: vacuum). Mass spectrometer: Source temperature: 250 ^{o}C, Quad-temperature 150 ^{o}C.

NMR spectroscopy

NMR spectra were recorded on an Agilent MR (^{1}H-NMR at 399.97 MHz, ^{13}C-NMR at 100.58 MHz). The ^{1}H NMR spectra were calibrated by using residual undeuterated solvent as an internal reference, and ^{13}C NMR spectra were calibrated using the ^{13}C peaks of deuterated solvents according to literature. [12]

X-ray Photoelectron spectroscopy (XPS)

X-ray photoelectron spectra of CVD graphene samples were recorded on a Quantum 2000 Scanning ESCA instrument) using a monochromatic Al K-Alpha X-ray (1486.7 eV) excitation source.

Raman scattering spectroscopy

Raman scattering spectra were recorded on a confocal microprobe Raman system (Renishaw, inVia) with an excitation wavelength of 532 nm.

Scanning electron microscopy (SEM)

SEM images were recorded using a Zeiss 1550 instrument at 5.0 kV with an in-lens energy selective backscattered electron detector.

Synthetic methods and results

- **Metal-free transfer photohydrogenation of polycyclic aromatic hydrocarbons (PAHs)**

Photostability of HCOOH at λ = 300 nm

In order to investigate the photostability of HCOOH (UV spectrum shown in Supplementary Figure 16) under irradiation at 300 nm we have conducted the following experiment:

Neat formic acid (5 mL) was added either in a pyrex tube (blocking λ< 300 nm) or in a quartz tube and irradiated in a Rayonet photoreactor at λ = 300 nm for 24 h. In both cases, after the irradiation, 50 μL of the liquid were added in an NMR tube sealed with a rubber septum. $CDCl_3$ was added (500 μL) and the ^{1}H-NMR spectra were recorded. Formaldehyde was not identified in none of the cases (Supplementary Figure 17).

General method

Typically for the photohydrogenation of small aromatic compounds (the ones with absorbing light of energy lower than 4.15 eV; λ > 300 nm), the aromatic compound of interest was dissolved in 100 mL of benzene/AcOEt (1/1: v/v) in concentration of 5 mM, and then degassed using Ar. In the degassed solution, 10 mL HCOOH were added (HCOOH concentration: 2.5 M; 500 × equimolar). The resulting solution was irradiated for 48 h in Rayonet at 300 nm and then the solvent was evaporated and the residue was purified by column chromatography.

Transfer photohydrogenation of anthracene

Transfer photohydrogenation of anthracene resulted in:

9,10-Dihydroanthracene: R_f : 0.44 (pentane); 8.5 mg (0.047 mmol), 4.5 %. ^{1}H NMR (400 MHz, $CDCl_3$): δ 7.31-7.29 (m, 4H), 7.21-7.18 (m, 4H), 3.95 (s, 4H); ^{13}C NMR (100 MHz, $CDCl_3$): δ 136.83, 127.53, 126.23, 36.31. GC (retention time): 7.475 min; MS: 179.1: $[M-H]^+$.
Anthracene dimer: (Obtained through post-elution with PhH). R_f: 0.866 (PhH); 71 mg (0.199 mmol), 35.47 %. ^{1}H NMR (400 MHz, $CDCl_3$): δ(ppm): 6.94 – 6.91 (m, 8H), 6.84 – 6.81 (m, 8H), 4.55 (s, 1H); ^{13}C NMR (100 MHz, $CDCl_3$): δ(ppm): 143.71, 127.19, 125.65, 53.78, 29.85. [13, 14]

Anthracene (recovered): 35 mg (0.196 mmol) GC (retention time): 8.140 min; MS: 178.0: $[M]^+$. ^{1}H NMR (400 MHz, $CDCl_3$): δ(ppm): 8.42 (s, 2H), 8.02 – 7.99 (m, 4H),7.47 – 7.45 (m, 4H).

Control experiment in the dark

Anthracene was dissolved in a 1/1 (v/v) mixture of PhH and AcOEt (97.9 mg: 0.55 mmol in 100 mL solvent mix.) in a 200 mL round bottom flask. The flask was sealed with a rubber septum and the solution was degassed with Ar gas. The solution was then covered with Al-foil and black plastic membrane (in order to avoid light exposure of the solution) and then HCOOH (10 mL) was added in the dark while stirring. The concentration of anthracene was 5 mM (0.55 mmol in 110 mL of PhH/AcOEt/HCOOH (5/5/1: v/v/v). The solution was stirred for several minutes and then kept in the dark for 48 h. After this period, the solvents were removed and the residue was analyzed using NMR spectroscopy and GC-MS.

Transfer photohydrogenation of phenanthrene

Transfer photohydrogenation of phenanthrene was performed according to the general procedure described above. According to GC-MS analysis, 9,10-dihydrophenanthrene (DHP) is produced in 3.5% conversion after 48 h irradiation at 300 nm in PhH/AcOEt/HCOOH. The conversion was determined after verifying the retention time using an authentic sample of 9,10-dihydrophenanthrene showing identical mass spectra. For the determination of the amount of DHP produced through GC-MS, a calibration curve was constructed (using commercially available DHP: Sigma Aldrich: D106003) and acetophenone as an internal standard.

Control experiment in the dark

Phenanthrene was dissolved in a 1/1 (v/v) mixture of PhH and AcOEt (97.9 mg: 0.55 mmol in 100 mL solvent mix.) in a 200 mL round bottom flask. The flask was sealed with a rubber septum and the solution was degassed with Ar gas. The solution was then covered with Al-foil and black plastic membrane (in order to avoid light exposure of the solution) and then HCOOH (10 mL) was added in the dark while stirring. The concentration of phenanthrene was 5 mM (0.55 mmol in 110 mL of PhH/AcOEt/HCOOH (5/5/1: v/v/v). The solution was stirred for several minutes and then kept in the dark for 48 h. After this period, the solvents were removed and the residue was analyzed using NMR spectroscopy and GC-MS.

Transfer photohydrogenation of pyrene

Transfer photohydrogenation of pyrene was performed according to the following procedure (slightly modified general procedure). Pyrene (14 mg : 63.3 mmol) was dissolved in a mixture of PhH and AcOEt (3 mL + 3 mL) in an RQV-5 quartz tube sealed with a rubber septum and 1 mL of HCOOH was added ([pyrene] = 10 mM). The solution was purged with Ar and irradiated in a Rayonet photoreactor (λ_{max} = 300 nm) for 48 h. After this period the solution turned deep brown. GC-MS analysis of the reaction mixture after 48 h showed that 4,5-dihydropyrene (DHPy) was produced in 2.0 % conversion. The conversion was determined after verifying the retention time using an authentic sample of 4,5-DHPy (Sigma Aldrich: T303089-1EA) showing identical mass spectra.

- **Metal-free photo(hydro)silylation of benzene and PAHs**

Photostability of triethylhydrosilane at λ = 254 nm

Photo(hydro)silylations of benzene and PAHs were conducted using triethylhydrosilane as a hydrosilylation reagent. The UV spectrum of triethylhydrosilane as well as various control experiments on its photostability upon irradiation at 254 nm are listed below.

UV spectrum of triethylhydrosilane

The reason for the selection of triethylsilane as a transfer hydrosilylation reagent is the fact that it has no chromophore that absorbs the 254 nm light (corresponding to an energy of 4.88 eV). The first strong UV absorption of Et_3SiH according to previously reported experiments (**Supplementary Figure 29**) is found at an excitation energy of 66 000 cm^{-1} = 8.18 eV, corresponding to an irradiation wavelength of 151 nm. Calculations at TD-B3LYP/6-311+G(2d,p)//B3LYP/6-311+G(d,p) and TD-PBE0/6-311+G(2d,p)//B3LYP/6-311+G(d,p) levels place strongly allowed excitations at 7.99 eV (155 nm) and 8.16 eV (152 nm), respectively, in good agreement with the experimental vacuum-UV spectrum (**Supplementary Figure 29A**). However, the first vertically excited singlet state of Et_3SiH is found at 6.84 and 7.06 eV, respectively, depending on the method, i.e., at wavelengths of 181 and 176 nm. Yet, the transition to this state has very low oscillator strength at both TD-DFT levels (f = 0.0043 and 0.0060, respectively), revealing that the transition to this state is essentially forbidden. Thus, experiments and computations unambiguously show that the 254 nm irradiation provides insufficient energy to excite Et_3SiH electronically (**Supplementary Figure 29B**).

Irradiation of triethylhydrosilane in n-pentane at 254 nm

In a dry 70 mL quartz tube Et_3SiH (500 μL: 3.13 mmol) was dissolved in dry *n*-pentane (50 mL). The concentration of the Et_3SiH solution formed was 63 mM. The solution was purged with argon gas and irradiated in a Rayonet photoreactor λ = 254 nm for 24 h. An air cooled condenser was used in order to avoid *n*-pentane (b.p: 36 ^{o}C) escape during the irradiation. After irradiation, *n*-pentane was removed *in vacuo*, and the remaining liquid was analyzed by ^{1}H-NMR spectroscopy. It was found that the liquid was unreacted Et_3SiH (^{1}H-NMR spectrum below, **Supplementary Figure 30**).

Irradiation of cyclohexene in triethylhydrosilane at 254 nm

In a dry quartz tube sealed with a rubber septum, cyclohexene (60 μL; 0.5916 mM) was added and it was dissolved in excess of Et_3SiH (900 μL). The concentration of the solution in cyclohexene was 625 mM and Et_3SiH was in 9.5-fold excess. The solution was purged with Ar and then the quartz tube was placed in a Rayonet photoreactor and irradiated with λ = 254 nm for 24 h. No polymer formation/precipitate was observed. The ^{1}H-NMR spectrum of the liquid solution was then recorded in $CDCl_3$ (Figure 28). It was found that the liquid consisted of Et_3SiH and cyclohexene. Therefore, no photoreaction was observed. If triethylsilyl radicals would have been formed, they would react with cyclohexene.

Irradiation of cyclohexene in excess of triethylhydrosilane and t-Bu_2O_2 (a radical photoinitiator) at 254 nm

In a dry quartz NMR tube, 20 μL (0.197 mmol) of cyclohexene was dissolved in a mixture of Et_3SiH and *t*Bu_2O_2, a radical photoinitiator[15] (200 μL and 300μL, respectively). The concentration of cyclohexene was 378 mM, that of Et_3SiH was 2.38 M (6.36-fold excess), and that of *t*Bu_2O_2 was 3.57 M (8.12-fold excess). The quartz tube was sealed with a rubber septum and the solution was purged with Ar gas. Finally, the solution was irradiated at λ = 254 nm for 24 h. After this period, a white insoluble precipitate was formed. The supernatant solution was analyzed as follows: 100 μL of the solution were dissolved in 500 μL of $CDCl_3$ and the ^{1}H-NMR spectrum was recorded (**Supplementary Figure 32**). It was concluded that all cyclohexene reacted (no peaks for cyclohexene were identified). This experiments along with that of the above paragraph proves that Et_3SiH photoreacts with cyclohexene only in photochemical conditions under which triethylsilyl radicals are produced (in absence of the radical photoinitiator no reaction occurred at 254 nm; see paragraph above).

Irradiation of Si_2Me_6 in benzene at 254 nm

Procedure: A solution of hexamethyldisilane in dry benzene (1 mL, [Si_2Me_6] = 12 mM) was purged with Ar and then added in a quartz NMR tube sealed with a rubber septum, using a syringe. The tube was then placed in a Rayonet photoreactor and irradiated at λ = 254 nm for 24 h. After this period no precipitate or polymer was identified. 100 μL of the solution were diluted in 500 μL of $CDCl_3$ and the sample was analysed through ^{1}H-NMR spectroscopy showing only benzene and unreacted hexamethyldisilane (see **Supplementary Figure 8**). Additionally, no peaks appeared in the region 2 to 6 ppm indicating that no disilylated benzenes were formed.

Irradiation of 1,3,5-cyclooctatriene in Et_3SiH at 254 nm

In a dry quartz NMR tube, 36 μL (0.313 mmol) of 1,3,5-cyclooctatriene (synthesized according to Oda *et. al.*[16]) was dissolved in Et_3SiH (500 μL; 3.13 mmol) with sonication. In the solution formed the concentration of 1,3,5-cyclooctatriene was 625 mM, that of Et_3SiH was 6.25 M (i.e., a 10-fold excess of Et_3SiH). The quartz tube was sealed with a rubber septum and the solution was purged with Ar gas. Finally, the solution was irradiated at λ = 254 nm for 24 h. After this period, ~10 % of the initial 1,3,5-cyclooctatriene was consumed according to GC-MS (**Supplementary Figure 34**). However, 1,3,5-cyclooctariene did not react with Et_3SiH, as no precipitate was formed and non silylated products were detected. Contrary, 1,3,5-cyclooctariene is known to undergo several types of photoreactions upon irradiation in nonpolar solvents such as photocycloadditions and photoisomerization to different volatile isomers according to Chapman *et al.*[17] and this is the reason why ~10% of the initial 1,3,5-cyclooctariene is consumed. The fact that no photoreaction between 1,3,5-cyclooctatriene and Et_3SiH occurred indicates that no triethylsilyl radicals were formed upon irradiation at 254 nm. If triethylsilyl radicals would have been formed, they would react with 1,3,5-cyclooctatriene.

Irradiation of 1,3,5-cyclooctatriene in excess of triethylhydrosilane and t-Bu_2O_2 (a radical photoinitiator) at 254 nm

In a dry quartz NMR tube, 20 μL (0.176 mmol) of 1,3,5-cyclooctatriene (synthesized according to Oda *et. al.*[16]) was dissolved in a mixture of Et_3SiH and *t*Bu_2O_2, a radical photoinitiator[15] (200 μL and 300μL respectively). In the solution formed the concentration of 1,3,5-cyclooctatriene was 340 mM, that of Et_3SiH was 2.38 M (7-fold excess), and that of *t*Bu_2O_2 was 3.57 M (10.5-fold excess). The quartz tube was sealed with a rubber septum and the solution was purged with Ar gas. Finally, the solution was irradiated at λ = 254 nm for 24 h. After this period, an insoluble pale-yellow precipitate was formed (see **Supplementary Figure 35**). The supernatant solution was analyzed through GC-MS (**Supplementary Figure 36**) and it was concluded that ~81.5 % of the initial 1,3,5-cyclooctatriene was consumed. This experiment along with that described in the paragraph above proves that Et_3SiH undergoes photoreaction against 1,3,5-cyclooctatriene only in photochemical conditions under which triethylsilyl radicals are produced (in absence of the radical photoinitiator no reaction occurred; see paragraph above).

Irradiation of cyclooctatetraene (COT) in excess of triethylhydrosilane and t-Bu_2O_2 (a radical photoinitiator) at 254 nm

In a dry quartz NMR tube, 20 μL (0.177 mmol) of COT was dissolved in a mixture of Et_3SiH and *t*Bu_2O_2 (200 μL and 300 μL, respectively). The concentration of COT was 340 mM, that of Et_3SiH was 2.38 M (7-fold excess), and that of *t*Bu_2O_2 was 3.57 M (10.5-fold excess). The quartz tube was sealed with a rubber septum and the solution was purged with Ar gas. Finally, the solution was irradiated at λ = 254 nm for 24 h. After this period, a sticky and insoluble yellow precipitate was formed (see **Supplementary Figure 37**). The supernatant solution was analyzed as follows: 100 μL of the solution were dissolved in 500 μL of $CDCl_3$ and the ^{1}H-NMR spectrum was recorded. It was concluded that 60 % of COT was consumed after 24 h of irradiation, according to ^{1}H-NMR spectroscopy.

Irradiation of Ph_3CH in cyclohexene at 254 nm

In a quartz tube 12 mg of triphenylmethane (0.05 mmol) was dissolved in 4.0 mL of cyclohexene (39.5 mmol). The concentration of triphenylmethane was 12.5 mM. The solution was purged with Ar and then placed in a Rayonet photoreactor and irradiated at λ = 254 nm for 24 h. During the photoreaction, most of the Ph_3CH reacted to give polymers according to ^{1}H-NMR (**Supplementary Figures 38 and 39**).

Here it has to be noted that the driving force for the photochemical reactivity of Ph_3CH and Ph_3SiH could in fact be excited state antiaromaticity of the phenyl rings, as evidence by the ACID plots of their relaxed triplet states (**Supplementary Figure 40**).

Photo(hydro)silylation of benzene

A solution of triethylhydrosilane in dry benzene (16 mL, [Et_3SiH] = 12 mM) was initially purged with Ar and then added in sixteen degassed quartz tubes sealed with rubber septa, using a syringe. Each tube contained 1 mL of 12 mM triethylhydrosilane solution in benzene. The tubes were then placed in a Rayonet photoreactor and irradiated at λ = 254 nm for 24 h. After the reaction the formation of a yellowish polymer on the surface of the tubes was observed. The solutions were combined and solvent (PhH) was evaporated *in vacuo*. The residual was dissolved in *n*-pentane and purified with column chromatography (SiO_2; *n*-pentane as eluent) to yield phenyltriethylsilane in yield 6.0 % (2.0 mg; % yield based on Et_3SiH). R_f : 0.94 (*n*-pentane); ^{1}H NMR (400 MHz, $CDCl_3$): δ 7.51-7.48 (m, 2H), 7.36-7.34 (m, 3H), 0.99-0.95 (t, 9H, J = 7.7 Hz), 0.83-0.77 (q, 6H, J = 7.8); ^{13}C NMR (100 MHz, $CDCl_3$): δ 137.62, 134.34, 128.84, 127.79, 7.54, 3.49. GC: 5.210 min; MS: 192.0: $[M]^+$, 163.0: $[M\text{-}Et]^+$, 135.0: $[M\text{-}2Et+H]^+$, 107.0: $[M\text{-}3Et+2H]^+$, 79.0 $[Ph+H]^+$.

Alternative method

A solution of benzene in triethylhydrosilane (5 mL, [PhH] = 70 mM) was added in a 10 mL quartz tube sealed with a rubber septum and purged with Ar. The solution was then irradiated with a low-pressure Hg lamp (λ_{max} = 254 nm; 16 W) for 24 h. The solution was then used directly for GC-MS. Different products were identified with MS, among them Et_3SiPh (MW=192 g/mol) as well as a product (not isolated) possibly corresponding to hydrosilylated benzene (MW = 194 g/mol).

Control experiment

Dry benzene was dissolved in triethylhydrosilane (5 mL, [PhH] = 70 mM) in a tube sealed with a rubber septum protected by light. After 48 h in the dark at r.t. the mixture was analyzed through ^1H-NMR.

Irradiation of an aerated benzene solution of Et_3SiH at λ=254 nm in a Rayonet photoreactor

An aerated 12 mM benzene solution of triethylhydrosilane was placed in a quartz tube (without any degassing). The tube was then placed in a Rayonet photoreactor and irradiated at λ = 254 nm for 24 h. After the reaction formation of polymer was not observed. The liquid mixture obtained was analyzed using GC-MS. $PhEt_3Si$ was formed in a much lower yield (0.35%) and furthermore various siloxanes were observed (the yield under strictly unaerated conditions was 6%). This result clearly indicates that the presence of O_2 hampers a lot the photo(hydro)silylation of benzene and furthermore leads to various oxidation products.

Investigating the effect of O2 on the photo(hydro)silylation of benzene using a Xe monochromatic UV light source

Xe-lamp irradiation experiments on the photo(hydro)silylation of benzene were performed using a Fluorolog-3-22 instrument (for details on the instrumentation and procedures see section **photochemical reactors and glassware**). Experiments were carried out both under aerated and strictly unaerated conditions. In both cases a dry *n*-heptane solution containing benzene in 8 mM concentration and Et_3SiH in a 100-fold excess was used.

In case of the experiments under unaerated conditions, the samples were prepared in a glove box with O_2 levels <0.1 ppm. The cuvette was sealed with a ground-glass joint with silicon grease, wrapped with many layers of Parafilm to ensure low O_2-levels over a long period of time. **Supplementary Figures 47 and 48** depict the changes in benzene absorbance under aerated and strictly unaerated conditions respectively.

- GC-MS analysis of the sample obtained from the experiment under strictly unaerated conditions revealed the formation of a disilylatedbenzene derivative (See **Supplementary Figure 49**).
- GC-MS analysis of the sample obtained from the experiment un the presence of O_2 showed no formation of $PhEt_3Si$ or other silylated benzenes. Instead various oxidation products of Et_3SiH were observed e.g. Et_3Si-O-$SiEt_3$ as well as higher siloxanes.

These results are therefore consistent with those obtained using a Rayonet photoreactor. As mentioned, in the latter case (Rayonet irradiation) the presence of O_2 significantly reduced the yield of $PhEt_3Si$ from 6 to 0.35 %. Additionally, in the presence of O_2 both irradiations in a Rayonet photoreactor at λ=254 nm and using a Xe-lamp at λ=254 nm gave oxidation products such as various siloxanes.

Photo(hydro)silylation of benzene using a KCl_{aq} filter

A solution of triethylhydrosilane in dry benzene (0.5 mL, [Et_3SiH] = 12 mM) was initially purged with Ar and then added in a degassed quartz tube sealed with a rubber septum, using a syringe. The tube was placed in a quartz tubes (RQV-5 Rayonet quartz tube; Ø 13 mm) containing a 10% (w/v) solution of KCl, strongly absorbing light between 180 at 200 nm) as shown in the following Figure. The tube was then placed in a Rayonet photoreactor and irradiated at λ = 254 nm for 24 h. After the reaction the formation of a yellowish polymer on the surface of the tubes was observed. The solution was then used directly for GC-MS. Different products were identified with MS, with Et_3SiPh (MW=192 g/mol) as the major product in solution in ~6% GC-MS conversion as shown in the GC-MS below. The use of the filter did not affect the outcome of the photo(hydro)silylation of benzene (see **Supplementary Figures 10-11**).

Irradiation of of triethylsilylcyclohexa-1,4-diene

According to GCMS during the photoreaction of benzene shown in **Supplementary Figure 41**, transient amounts of monohydrosilylated benzenes are produced, which evolve further to result in polymer formation. In order to show that photoreactivity of monohydrosilylated benzenes we have synthesized separately triethylsilylcyclohexa-1,4-diene and irradiated that at 254 nm according to the following procedure.

In a quartz NMR tube 8 mg (0.041 mmol) of triethylsilylcyclohexa-1,4-diene (synthesized according to Simonneau and Oestreich[18]) were dissolved in 1 mL of *n*-heptane. The concentration of the resulting solution in triethylsilylcyclohexa-1,4-diene was 41 mM. The solution was purged with Ar and placed in a Rayonet photoreactor and irradiated at 254 nm for

24 h. After this period insoluble polymer was formed. The supernantant solution was analysed through GC-MS and it was shown that all triethylsilylcyclohexa-1,4-diene was consumed (**Supplementary Figure 52**).

The observed reactivity is consistent with the calculated TD-DFT spectra that show that both triethylsilylcyclohexa-1,4-diene (**Supplementary Figure 53**) and its isomer triethylsilylcyclohexa-1,3-diene (**Supplementary Figure 54**) do absorb at 254 nm.

Attempted photohydrosilylation of COT

In a dry quartz NMR tube 0.50 mL of Et_3SiH (3.13 mmol) and 33 mg of COT (0.313 mmol) were dissolved in 1 mL of *n*-heptane. The concentration of the Et_3SiH was 2.09 mM. The 0.0solution was purged with Ar and then placed in a Rayonet photoreactor and irradiated at λ = 254 nm for 24 h. After the photoreaction, only starting material was recovered according to ^{1}H-NMR (**Supplementary Figure 56**).
The same reaction was recorded after 24, 48 and 72 h of irradiation. Equimolar quantity of benzene with respect to the starting concentration of reaction mixture along with 0.4 mL of $CDCl_3$ was added in NMR tubes containing reaction mixtures before and after irradiation. All ^{1}H-NMR spectra were recorded with nt = 32, and dl = 10 sec. Difference in the integrals of peaks before and after irradiation at different irradiation times are shown in **Supplementary Figure 55**.

Here it has to be noted that COT when irradiated at 254 nm for 72 h in *n*-heptane in the absence of Et_3SiH it readily undergoes photodecomposition. According to ^{1}H-NMR, 58% of the initial COT was consumed after 72 h irradiation at 254 nm (**Supplementary Figure 57**).

Photo(hydro)silylation of naphthalene

Photo(hydro)silylation of naphthalene using a 50-fold excess of Et_3SiH in a home-made FEP-photoreactor

A 15 mL solution of naphthalene (55.7 mg) in dry *n*-heptane containing a 50-fold excess of Et_3SiH was purged with Ar. The concentration of naphthalene was 29 mM and that of Et_3SiH was 1.45 M. The degassed solution was introduced under Ar in a home-made batch FEP-photoreactor (see section **photochemical reactors and glassware**). The sample was irradiated for 16 h at λ=254 nm using a Peschl Hg lamp (see section **photochemical reactors and glassware**). After the irradiation the sample was collected and the solvent was removed under vacuum. The remaining oil was purified by column chromatography on a silica gel column eluting with pentane to give a ~1:1 mixture of α- and β-triethylsilylnaphtalene in a combined yield of 21 % (22 mg). ^{13}C NMR (100 MHz, $CDCl_3$): δ(ppm): 137.51(α), 135.19(β), 134.98(α), 134.78(β), 134.43(α), 133.60(β), 133.37(α), 132.94(β), 130.58(β), 129.55(α), 129.04(α),

127.99(β), 127.89(α), 127.66(β), 126.72(β), 126.11(β), 125.72(β), 125.48(α), 125.19(α), 125.02(α), 7.64(α), 7.43(β), 4.51(α), 3.39(β). (NMR assignments according to: Zarate *et al.* [19]). GC: 8.356 min; MS: 242.2: $[M]^+$ and GC: 8.456 min; MS: 242.2 $[M]^+$.

Photo(hydro)silylation of naphthalene using a 10-fold excess of Et_3SiH in a home-made FEP-photoreactor

A 15 mL solution of naphthalene (55.7 mg) in dry *n*-heptane containing a 10-fold excess of Et_3SiH was purged with Ar. The concentration of naphthalene was 29 mM and that of Et_3SiH was 290 mM. The degassed solution was introduced under Ar in a home-made batch FEP-photoreactor (see section **photochemical reactors and glassware**). The sample was irradiated for 16 h at λ=254 nm using a Peschl Hg lamp (see section **photochemical reactors and glassware**). After the irradiation the sample was analyzed with GC-MS indicating the formation of a ~1:1 mixture of α- and β-triethylsilylnaphtalene in a combined GC-MS yield of 15%.

Photo(hydro)silylation of naphthalene using a 10-fold excess of Et_3SiH in a a Rayonet photoreactor

In a 70 mL cylindrical quartz tube, a solution of naphthalene in dry *n*-heptane was added (187 mg, 1.46 mmol in 50 mL *n*-heptane). The tube was sealed and the solution was degassed with Ar and then 2420 μL (15.15 mmol: ~11-fold excess) of triethylhydrosilane was added under Ar. The final concentration of naphthalene was thus 28 mM and that of Et_3SiH was 289 mM. The quartz tube containing the reaction mixture was placed in the Rayonet photoreactor and irradiated for 72 h at 254 nm. After irradiation, the solvent was evaporated on a rotary evaporator and the remaining oil was purified by column chromatography on a silica gel column eluting with pentane to give 9.0 % yield (29 mg) of a mixture of α- and β-triethylsilylnaphthalene. R_f: 0.80 (pentane). ^{1}H NMR (400 MHz, $CDCl_3$): δ(ppm): 8.09-8.08 (d, 1H, α), 7.98 (s, 1H, β), 7.85-7.80 (m, 5H: 2Hα, 3Hβ), 7.67-7.66 (d, *J* = 4, 1H, α or β), 7.58-7.56 (d, *J* = 8.0, 1H, α or β), 7.50-7.43 (m, 5H, 2β, 3α). ^{13}C NMR (100 MHz, $CDCl_3$): δ(ppm): 137.51(α), 135.19(β), 134.98(α), 134.78(β), 134.43(α), 133.60(β), 133.37(α), 132.94(β), 130.58(β), 129.55(α), 129.04(α), 127.99(β), 127.89(α), 127.66(β), 126.72(β), 126.11(β), 125.72(β), 125.48(α), 125.19(α), 125.02(α), 7.64(α), 7.43(β), 4.51(α), 3.39(β). GC: 8.356 min; MS: 242.2: $[M]^+$ and GC: 8.456 min; MS: 242.2 $[M]^+$.

Photo(hydro)silylation of naphthalene using a 50-fold excess of Et_3SiH in a Rayonet photoreactor

In a 70 mL cylindrical quartz tube, a solution of naphthalene in dry *n*-heptane was added (187 mg, 1.46 mmol in 50 mL *n*-heptane: 29.2 mM). The tube was sealed and the solution was degassed with Ar and then 11.20 mL (75.75 mmol: ~50-fold excess). of triethylhydrosilane was

added under Ar. The final concentration of naphthalene was thus 24 mM and that of Et_3SiH was 1.26 M. The quartz tube containing the reaction mixture was placed in the Rayonet photoreactor and irradiated for 72 h at 254 nm. After irradiation, the solvent was evaporated on a rotary evaporator and the remaining oil was purified by column chromatography on a silica gel column eluting with pentane to give 11.0 % yield (36 mg) of a mixture of α- and β-triethylsilylnaphthalene. R_f: 0.80 (pentane). ^1H and ^{13}C NMR as described in paragraph above.

Control experiment (dark)

In a 100 mL round bottom flask a solution of naphthalene in *n*-heptane (187 mg: 1.46 mmol in 50mL *n*-heptane) was added, the flask was sealed with a septum and the solution was degassed with Ar gas. The solution was covered with Al-foil and black plastic membrane (in order to avoid light exposure of the solution) and then triethylhydrosilane (242 μL: 15.15 mmol) was added in the dark. The solution was stirred for several minutes and then kept in the dark for 48 h.

Photo(hydro)silylation of anthracene

A 5 mM benzene solution of anthracene containing a 125-fold excess of Et_3SiH ($[Et_3SiH]$= 625 mM) was degassed in a borosilicate glass tube through freeze-pump-thawing (4 cycles). The degassed sealed tube was placed inside a glovebox with low oxygen concentration (<1ppm) and irradiated with a Hg lamp at λ=365 nm for 24 h. After this period a white precipitate was formed (anthracene dimer). GC-MS analysis of the solution obtained showed the formation of 9-(triethylsily)-9,10-dihydroanthracene in a GC-MS yield of 4.5 % (**Supplementary Figure 66**). ^1H-NMR analysis results are shown in **Supplementary Figure 68**.

Control experiment (dark)

In a 200 mL round bottom flask a solution of anthracene in the PhH (100 mg: 0.562 mmol in 100 mL PhH) was added and then the flask was sealed with a septum and the solution was degassed with Ar gas. The solution was covered with Al-foil and black plastic membrane (in order to avoid light exposure of the solution) and then Et_3SiH (5 mL, 31.304 mmol)) was added in the dark while stirring. The concentration of anthracene was 5.35 mM (0.562 mmol in 105 mL of PhH/Et_3SiH. The solution was stirred for several minutes and then kept in the dark for 48 h. After this period, the solvents were removed and the residue was analyzed using NMR spectroscopy and GC-MS. It was shown that no reaction occurred and the starting compounds were fully recovered.

Photo(hydro)silylation of phenanthrene

In a dry 10 mL quartz tube sealed with a rubber septum a solution of 11.5 mg phenananthrene (0.0646 mmol) in 5 mL of dry *n*-heptane ([phenanthrene] ≈ 13 mM) was added using a syringe. To this solution 105 μL of Et_3SiH was added (10 equivalents). The resulting solution was purged with Ar and then placed in a Rayonet photoreactor and irradiated for 48 h (λ_{max} = 300 nm). A

snapshot of the reaction was analyzed after 24 h of irradiation. After 48 h of irradiation a small quantity of a thin yellow-orange precipitate was formed. After filtration of the precipitate (insoluble) the solution was analyzed using GC-MS.

Control experiment in the dark

In a 200 mL round bottom flask a solution of phenanthrene in *n*-heptane (100 mg: 0.562 mmol in 100 mL *n*-heptane) was added and the flask was sealed with a septum and the solution was degassed with Ar gas. The solution was covered with Al-foil (in order to avoid light exposure of the solution) and then Et_3SiH was added (5 mL, 31.304 mmol)) in the dark while stirring. The concentration of phenanthrene was 5.35 mM (0.562 mmol in 105 mL of *n*-heptane/Et_3SiH. The solution was stirred for several minutes and then kept in the dark for 48 h. After this period, the solvents were removed and the residue was analyzed using GC-MS.

Synthesis of 9-triethylsilylphenanthrene

9-Triethylsilylphenanthrene was synthesized according to a method described by Nogaideli *et al.*[20] This compound was synthesized in order to verify that it is formed photochemically through the reaction of **Supplementary Figure 70**. The GC of an authentic sample of 9-triethylsilylphenanthrene exhibits one peak with retention time: 11.039 min and m/z: 292 corresponding to: $[M]^+$ (**Supplementary Figure 71**). As shown in **Supplementary Figure 72** this compound was also identified through GC-MS after the photo(hydro)silylation of phenenanthrene as the main product (retention time: 11.039 min, m/z: 292: $[M]^+$).

Investigating the effect of O_2 on the photo(hydro)silylation of phenanthrene using a Xe monochromatic UV light source

Xe-lamp irradiation experiments on the photo(hydro)silylation of phenanthrene were performed using a Fluorolog-3-22 instrument (for details on the instrumentation and procedures see section **photochemical reactors and glassware**). Experiments were carried out both under aerated and strictly unaerated conditions. In both cases a dry *n*-heptane solution containing phenanthrene and Et_3SiH in a 100-fold excess was used.

In case of the experiments under unaerated conditions, the samples were prepared in a glove box with O_2 levels <0.1 ppm. The cuvette was sealed with a ground-glass joint with silicon grease, wrapped with many layers of Parafilm to ensure low O_2-levels over a long period of time. **Supplementary Figures 75** depicts the changes in phenanthrene absorbance under aerated and strictly unaerated conditions respectively. The effect of O_2 in the photo(hydro)silylation of phenanthrene is obvious through the plot of **Supplementary Figure 75**.

GC-MS of the sample obtained after irradiation in the presence of O_2 showed that a new product was formed which does not correspond to a (hydro)silylated phenanthrene. This means that phenanthrene in the presence of O_2 and under irradiation in excess of Et_3SiH follows another reaction pathway (see **Supplementary Figure 76**).

- ## Transfer photohydrogenation and photo(hydro)silylation of graphene

Potential impurities and defects in graphene

Check of metal impurities in graphene

In order to ensure that the CVD grown graphene samples used in this work were free of metal impurities we performed several survey XPS analysis on various samples. No other elements than O, Si (SiO_2 was used as the substrate of the CVD graphene samples) and C were identified (**Supplementary Figure 77**, **81** and **87**). Furthermore, as copper might be the only metal which was used for the preparation of the CVD graphene samples additional high resolution XPS analyses on Cu as well as Ni (a common impurity of copper) were performed (**Supplementary Figure 78**).

Check of dangling bonds in graphene

We furthermore checked if dangling bonds could appear in our CVD grown graphene samples. Dangling bonds in graphene samples are highly sensitive in Raman spectroscopy as a D peak (indicating defects) and it should have been clearly visible in our studies of the pristine graphene samples if we have had graphene with dangling bond defects. In order to explicitly address this issue we have now introduced dangling bonds by usage of the Focused Ion Beam (FIB) technique. Interestingly, even when introducing a rather low density of dangling bonds (max 2 dangling bonds per 10000 carbon atoms) into graphene a D peak of high intensity appears in the Raman scattering spectrum (**Supplementary Figure 79A**). However, the Raman scattering spectra of our pristine graphene samples show no D band (**Supplementary Figure 79B**). In other words, if dangling bonds were present in our pristine graphene samples, their Raman scattering spectra would contain a D band. It can therefore be concluded that the graphene samples we use are free of dangling bonds.

Transfer photohydrogenation of graphene

Usage of white light-emitting diodes

A typical experiment was performed as follows: a CVD graphene sample was immersed in aqueous HCOOH (typically mixtures HCOOH/H_2O were used in volume ratios: 1/4, 1/1, 4/1) in a glassy tube (borosilicate; 5 mm wide), capped with a septum and then the liquid mixture was degassed with Ar for several minutes. After that the tube was placed in the center of a light source, a set-up containing LEDs (see section **photochemical reactors and glassware**). The distance from the light source was 38 mm). Illumination time ranged between 6 and 48 h. After this period the liquid mixture was removed from the tube and the sample was collected and washed carefully with distilled/deionized water and then dried with a stream of Ar gas (results in **Supplementary Figures 82-84**) .

Control experiment

- Control experiments were conducted as follows: graphene samples were immersed in HCOOH/H_2O (1/1 v/v) contained in light-protected vials in the dark. The samples were degassed with Ar gas and remained in the dark for 24 h. After that, each sample was removed and washed with distilled/deionized water avoiding light exposure, and finally dried carefully.
- Additionally, transfer photohydrogenation was attempted in control experiments using acetic acid, and since this acid is not a transfer hydrogenation agent hydrogenated graphene was not observed

Photohydrogenation of graphene using sun-light

Photohydrogenation of graphene was conducted as follows: a CVD graphene sample was placed in a vial (borosilicate) and then a 1/1 mixture of HCOOH and H_2O (v/v) was added. The vial was sealed and then exposed to sun-light. The experiment was carried out close to Heraklion, Crete, Greece (35.3333° N, 25.1333° E) during the day-time of the 20th and 21st of August 2014, two days without any clouds. The total illumination time was 26 h and 38 min. Average temperatures: 26 ^{o}C on August 20 and 27 ^{o}C on August 21, 2014 (see **Supplementary Figure 85**).

Transfer photohydrogenation of graphene through UV-irradiation

In a quartz NMR tube containing a CVD-graphene sample, degassed with Ar and sealed with a rubber septum, 100 μL of a 1/1 (v/v) mixture of HCOOH and H_2O (degassed with Ar) was added. The sample was irradiated in a Rayonet photoreactor at 300 nm for 24 h, and then the graphene sample was washed with water (distilled/deionized) and finally dried with a stream of Ar gas (**Supplementary Figure 86**).

Photo(hydro)silylation of graphene

Typically CVD graphene samples were immersed in neat triethylhydrosilane (Et_3SiH) in a glass tube (borosilicate glass) degassed with Ar, and illuminated using series of white LEDs adjusted in distance of 3.75 cm from the LEDs (see section **photochemical reactors and glassware**) for periods varying between 6 and 24 h. The samples after the illumination were washed using very large amounts of dichloromethane (DCM) in order to remove residual unreacted Et_3SiH from the surface of the graphene sample (approximately 100 mL of DCM per sample were injected on the surface of the samples and dried using a stream of Ar gas).

Control experiment

Control experiments were conducted as follows: graphene samples were immersed in neat Et_3SiH contained in a light-protected vial in the dark. The sample was degassed with Ar gas and remained in the dark for 24 h. After that, each sample was removed and washed with very large amounts of dichloromethane avoiding light exposure, and finally dried carefully. The sample was analyzed through XPS. The same procedure was repeated twice.

Pristine graphene (**Supplementary Figure 90A**) shows mostly monolayer thick regions with distinct grain boundaries with a typical distance in the 10 micron range. After photohydrogenation (**Supplementary Figure 90B**), the graphene film shows interruptions and seperates into islands with a diameter in the 10 micron range suggesting that the graphene film is broken up at the place of the grain boundaries in **Supplementary Figure 90A**. A possible driving force for this evolution is stress that is related to the formation of sp^3 bonds. The breaking up of the the graphene film is observed to a lesser degree after photosilylation.

The impact of CVD-graphene defects on its photoreactivity

A reviewer suggested that the photoreaction may start at defects that propagate. To investigate this experimentally we have performed photohydrogenation on CVD grown graphene purchased from the company *Graphenea* (http://www.graphenea.com/) and compared to the samples from the *Graphene Supermarket* (https://graphene-supermarket.com/) which we previously used. Scanning electron microscopy (SEM) shows that the samples obtained from *Graphenea* have larger grain size and thus less defects (see **Supplementary Figure 91**A and B), and therefore we can investigate the impact of defects on the photoreactivity. However the extent of photohydrogenation in both cases is in the same order of magnitude according to C1s XPS analysis (**Supplementary Figure 91C-F**), showing that the photoreaction does not occur only at defects.

The following procedures were used for the experiments corresponding to Supplementary Figure 91C-F

Procedures for Graphenea:

Inside a glassy tube (borosilicate; 5 mm wide), a CVD-grown graphene sample purchased by Graphenea was immersed in a 1/1 (v/v) $HCOOH/H_2O$ mixture capped with a rubber septum and then the liquid mixture was degassed with Ar for several minutes. After that the tube was placed in the center of a light source, a set-up containing LEDs and irradiated for 6 h and for 24 h . The distance from the light source was 38 mm. After this period the liquid mixture was removed from the tube and the sample was collected and washed carefully with distilled/deionized water and then dried with a stream of Ar gas. The sample was analyzed using XPS. It was shown that the extent of hydrogenation was similar to the one obtained for a sample purchased from Graphene super market (Supplementary Figure 91) after identical phototreatment.

Procedures Graphene Supermarket:

Inside a glassy tube (borosilicate; 5 mm wide), a CVD-grown graphene sample purchased by Graphene Supermarket was immersed in a 1/1 (v/v) $HCOOH/H_2O$ mixture capped with a rubber septum and then the liquid mixture was degassed with Ar for several minutes. After that the tube was placed in the center of a light source, a set-up containing LEDs (**Supplementary Figure 4**) and irradiated for 6 and 24 h. The distance from the light source was 38 mm. After these periods the liquid mixture was removed from the tube and the sample was collected and washed carefully with distilled/deionized water and then dried with a stream of Ar gas. The sample was analyzed using XPS. It was shown that the extent of hydrogenation was similar to the one obtained for a sample purchased from Graphene super market (**Supplementary Figure 91**) after identical phototreatment.

Study of the effect of O_2 on the photo(hydro)silylation of graphene

All of the described photoreactions of graphene were carried out under inert conditions (after prior purging with Ar). In order to show that there is no effect of O_2, we have also performed irradiation experiments on graphene in the presence of O_2. It was unambiguously found that the extent of (hydro)silylation of graphene is not influenced by O_2.

The high resolution Si2p XPS of a sample irradiated for 24 h in saturated in air liquid Et_3SiH (**Supplementary Figure 92**) showed that the integral of the band at 102.3 which is attributed to (hydro)silylated graphene is as high as 17.3% i.e. slightly lower than that obtained after 24 h irradiation under deoxygenated conditions (20.1%).

Thermal reaction between HSiEt3 and graphene of graphene

We have furthermore investigated the thermal reaction between $HSiEt_3$ and graphene: Microwave irradiation of graphene at 180 ^{o}C in Et_3SiH containing 10% DMF by volume for 12 h. However, graphene was not altered in terms of C1s and Si2p XPS after this period.

Computational part

Computational details

DFT calculations were performed with Gaussian09, Revision D.01[21] at the B3LYP level[22] using the 6-311+G(d,p) basis set by Pople and co-workers[23]. Free energies were calculated at 298.15 K and 1 atm by Gaussian09 under the ideal gas approximation based on the obtained vibrational frequencies. Anisotropy of the induced current density (ACID) plots were generated with the AICD 2.0.0 program[24,25] at the B3LYP/6-311+G(d,p) level using the Continuous Set of Gauge Transformations (CSGT) method.[26] ACID is a general method for visualizing electron delocalization and ring currents. Atomic coordinates of all computationally investigated compounds are provided by the authors upon request. Additional ACID plots were generated with the ωB97X-D[27] and M06[28] functionals. TD-DFT calculations were performed with the B3LYP and PBE0 functionals[29] with the 6-311+(2d,p) basis set based on the B3LYP/6-311+G(d,p) geometries. The simulated spectra were generated with GaussView 5.[30] The NICS-XY scan is a method to assess ring currents in polycyclic systems[31]. We used the GIAO[32] procedure to calculate NICS values at 1.7 Å above the molecular plane and the σ-only model[33] to show the effect of the π electrons only. Minima with substantially negative NICS values in the NICS-XY scan represent aromatic rings, while maxima with substantially positive values represent antiaromatic rings. NICS values around zero correspond to non-aromatic rings. For benzene, the minimum NICS value at 1.7 Å distance above the ring plane is -16 ppm with the σ-only model, while the maximum value for D_{2h} cyclobutadiene is ca +20 ppm.[31] We used the following keywords in Gaussian09 to control the grid size for the GIAO calculations: “cphf=grid=fine” and “integral=grid=ultrafine”. The CASSCF[34] and CASPT2[35] calculations in conjunction with a ANO-RCC one-particle basis set[36] were performed with the MOLCAS 8.1 quantum chemistry program package. [37]

Hydrogenation energies

Hydrogenation energies were calculated according to the general reaction scheme depicted in **Supplementary Figure 94**. The computed reactions, involving anionic, neutral and cationic annulenes, are depicted in **Supplementary Figures 95-97**. The hydrogen atoms have been

placed so as to yield the longest possible linear conjugation paths in the hydrogenated products. The selection of compounds with 8 π-electrons at first included the cycloheptatrienyl anion. However, we decided to exclude this compound as its hydrogenated product displayed a Rydberg T_1 state instead of a ππ* state, which precluded a comparison with the other compounds. For the compounds with 10 and 12 π-electrons, the calculated compounds were chosen so that the π system is locked in a planar configuration. The structures were optimized at the (U)B3LYP/6-311+G(d,p) level, yielding minima with no imaginary frequencies.

Average hydrogenation energies were calculated with equal weight for all the isomers with the same number of π-electrons, and these are collected in **Supplementary Tables 3-7**. For the annulenes with 10 and 12 π-electrons, *cis* and *trans* isomers can be formed upon hydrogenation. Both of these isomers have been included in the energy average as separate items. For compounds **K** and **L**, two isomers were found for the parent annulenes. For **K**, the two isomers are found in the T_1 state, while for **L**, the two isomers are found in the S_0 state. When calculating the hydrogenation energies for the products of these compounds, the energy of the parent annulene is taken as the average of the energies of the two isomers. The annulenes **I**, **J**, **K**, **L**, **M**, and **N** were chosen instead of the simpler $C_9H_9^-$, $C_{10}H_{10}$, $C_{11}H_{11}^+$, $C_{11}H_{11}^-$, $C_{12}H_{12}$, and $C_{13}H_{13}^+$ as the former are nearly planar, while the latter have a tendency to distort to non-planar structures. For example, $C_{10}H_{10}$ is known to favor a non-planar structure with large bond length alternation,[38] lowering its potential S_0 aromaticity drastically. In contrast, 1,6-methano[10]annulene is forced to be planar and has a small bond length alternation in S_0.[39] This effect is of course even larger for antiaromatic structures than for $C_{10}H_{10}$ which has the potential to be S_0 aromatic in the planar form. By choosing annulenes that cannot distort, the effect of aromaticity and antiaromaticity becomes more apparent. To illustrate this, we calculated the hydrogenation free energy for $C_{10}H_{10}$ in S_0 which is highly exergonic (-18.9 kcal/mol) and similar to that of a non-aromatic polyene (ca. -30 kcal/mol, see **Supplementary Figure 106**). In contrast, the near-planar compounds used for Figure 1 (Manuscript Figure) have endergonic hydrogenation energies (average of +8.3 kcal/mol) due to the extra aromatic stabilization which is not present in the distorted $C_{10}H_{10}$.

Although the spread is large within each compound class, the similar separate averages over negative, positive and neutral compounds with equal number of electrons indicate that the results are trustworthy. However, the large range indicates that the predictions made by electron-counting rules might not be valid for all reactions for all compounds, but rather is a general rule of thumb.

The electronic energy of H_2 was calculated to -1.179571023 a.u. and the Gibbs free energy to -1.180994 a.u.

H-atom abstraction calculations by S_1 state benzene

Benzene in S_1 was optimized with CASSCF/ANO-RCC-VDZP using a state-average procedure with the two lowest states of singlet multiplicity and an active space of 6 electrons in 6 orbitals. The S_1 TS for hydrogen atom transfer between benzene and Et_3SiH (**Supplementary Figure 105**) was optimized using a state-average procedure with the two lowest states of singlet multiplicity and an active space of 8 electrons in 8 orbitals, incorporating the π orbitals of benzene as well as the σ and σ* orbitals of the Si-H bond. The identity of the TS was confirmed by IRC analysis. The electronic energies were computed with MS-CASPT2/ANO-RCC-VTZP using the CASSCF geometries, while the thermal corrections to the Gibbs free energy came from CASSCF/ANO-RCC-VDZP frequency calculations.

Optimization and frequency calculations for Et_3SiH was performed with HF/ANO-RCC-VDZ. Single-point energies were calculated with MP2/ANO-RCC-VTZP while the thermal corrections to the Gibbs free energy were obtained from the HF/ANO-RCC-VDZ frequencies.

The activation free energy relative to the free reactants in S_1 was 27.2 kcal/mol, which can be compared to the much lower T_1 activation energy of 7.9 kcal/mol relative to the reactants as calculated with B3LYP-D3(BJ)/6-311+G(d,p) (see **Supplementary Figure 100**). It should be noted that the S_1 TS coincides with conical intersection between S_1 and S_2 at the MS-CASPT2 level and the activation energy could possibly be somewhat lower if the TS structure was optimized at this level (a few kcal/mol). Due to the high activation energy in S_1, the hydrogen transfer reaction between Et_3SiH and benzene is more likely to occur in T_1.

Calculated hydrogenation energies for benzene and selected non-aromatic alkenes

To further assess the influence of excited state antiaromaticity on the energetics of hydrogenation, we compared the values for benzene with a set of non-aromatic alkenes (**Supplementary Figure 106**). The following conclusions can be made:

1. For the non-aromatic alkenes, the average hydrogenation energy is -16 kcal/mol in S_0 and -5 kcal in T_1. This represents a decrease in the exergonicity by 11 kcal/mol going from S_0 to T_1.
2. Hydrogenation of benzene is less excergonic than the average non-aromatic olefin by 33 kcal/mol in S_0 and more exergonic by 13 kcal/mol in T_1.

The finding that hydrogenation of T_1 state benzene is 13 kcal/mol more facile than for comparable non-aromatic olefins is consistent with the destabilization energy due to T_1 antiaromaticity that has been determined to 17 kcal/mol.[40,41] In the same way, the increased exergonicity in S_0 by 33 kcal/mol compares nicely to the aromatic stabilization energy of benzene in S_0 which is determined to 33 kcal/mol.[42]

Collapse of singlet radical pair to trimethylsilylcyclohexadiene

To investigate if the combination of the benzenium radical and trimethylsilyl radical (Me3Si$^{\bullet}$) is a barrierless process, we conducted a relaxed scan of the C-Si distance from the equilibrium at 1.934 Å to 3.334 Å in steps of 0.1 Å at the (U)B3LYP/6-31G(d) level of theory. The results are shown in **Supplementary Figure 107**. The curve smoothly approaches the calculated reaction energy of 54.9 kcal/mol without any barrier. At distances above 3.334 Å the optimization procedure fails as there is too weak interactions between the two fragments. The computed <S2> values indicate a large increase in biradical character as the bond is stretched above 3 Å. To validate our UDFT calculations we computed single-point CASPT2(6in6)/ANO-RCC-VDZP energies at the B3LYP geometries. The shape of the curve is the same, confirming the results from UDFT, although the reaction energy is higher at the CASPT2 level.

ACID plots of benzene and PAHs

In **Supplementary Figures 112-117**, ACID plots of benzene, naphthalene, anthracene, phenanthrene, fluoranthene, pyrene and coronene are presented. Clockwise arrows correspond to diatropic currents typical of aromatic compounds, while counterclockwise arrows correspond to paratropic currents typical of antiaromatic compounds. Results are given for B3LYP/6-311+G(d,p) as well as M06/6-311+G(d,p), ωB97X-D/6-311+G(d,p) and HF/6-311+G(d,p)//B3LYP/6-311+G(d,p) for comparison. ACID isosurfaces are at 0.050 a.u. For coronene, the 6-311+G(d,p) basis set gave unphysical imaginary frequencies both in S_0 and T_1 for the planar structures. This is similar to that observed by Jensen for the BLYP functional with the aug-pc-1 basis set.[43] Therefore, geometry optimizations for coronene were done using the 6-311G(d,p) basis set instead, yielding planar structures with no imaginary frequencies, while the ACID calculations used 6-311+G(d,p). The C-C bond lengths for the geometries which the ACID calculations are based on are given in **Supplementary Figure 118**.

The quality of the unrestricted Kohn-Sham solution can be assessed by the expectation value of the total spin-squared operator, $\langle S^2 \rangle$. For a triplet, the proper value of $\langle S^2 \rangle$ is 2.00. Although some spin contamination is always expected for unrestricted DFT,[44] the $\langle S^2 \rangle$ value can still serve as a diagnostic for the quality of the Kohn-Sham solution.[45] Generally, the results for HF

are heavily spin contaminated, and cannot be trusted. B3LYP, M06 and ωB97X-D agree on the ring current patterns in all compounds except the T_1 states of phenanthrene. In phenanthrene, antiaromatic current is also present in the outer rings with B3LYP and M06, while ωB97X-D localizes the antiaromaticity to the central ring to a higher extent. The behavior of ωB97X-D might be related to the higher degree of spin contamination (2.0787).

NICS-XY scans and NICS values

The NICS-XY scans for benzene, naphthalene, anthracene, phenanthrene, pyrene, fluoranthene and coronene are given in **Supplementary Figures 122 to 124** together with an interpretation of the ring currents based on the plots. The path of the scan is also given in the figures. Vertical red lines in the plots indicate the positions of ring centers, while vertical black lines indicate the positions of bonds. The results are fully consistent with the ring currents given in the ACID plots (**Supplementary Figure 111** to **Supplementary Figure 117**). Coronene (**Supplementary Figure 124**) deserves closer investigation. Although the circular scan displays some oscillations, the values are within a very narrow range 49-55 ppm that is clearly representative of an antiaromatic (paratropic) current. The central ring of coronene displays a lower NICS value (ca 30 ppm) as compared to the other rings (49-55 ppm). One interpretation which is consistent with the ACID plot is that there is a smaller diatropic current in the central ring which together with the paratropic ring current in the outer rings gives a lower paratropic NICS value. However, other interpretations are likely possible.

NICS Values: Also the computed $NICS(1)_{zz}$ in T_1 (**Supplementary Figure 125**) confirm the conclusions from the NICS-XY scans and ACID plots.

Conformers of triplet benzene

The conformational space of benzene has been investigated quite extensively.[46,47,48,49] In particular, Segev and co-workers identified four different conformations using DFT that were either minima or transition states.[49] These are shown in **Supplementary Figure 126** at the B3LYP/6-311+G(d,p) level and we label them as the quinoid at D_{2h} symmetry (**Q_{D2h}**), the diallyl (**DA**), the quinoid at C_{2v} symmetry (**Q_{C2v}**), and the methyl-pentadienyl (**MP**).

Their energies at the separately optimized B3LYP, M06-2X, B2PLYP[50] and G4[51] levels are given in **Supplementary Table 8**. In addition, single-point energies with CCSD(T)/cc-pVTZ at the B3LYP geometry are also given.[52,53] Different methods give different isomers as the most stable, and there is also a discrepancy when considering electronic energies or the free energies. Overall, the potential energy surface is very shallow. As pointed out by Takashi and Ohno,[48] there are strong anharmonicity effects and the standard frequency calculations are not reliable.

Therefore, the calculated free energies are not accurate. Experimentally, all structures are likely accessible, as expressed by Van Hemert and co-workers:[46] "The vibrational wave function of the zero-point vibration demonstrates that the molecule in this state is by no means confined to the trough but makes large excursions over the complete energy surface."

To elucidate the antiaromatic character of the different isomers, we calculated ACID plots (**Supplementary Figure 127**) and NICS scans[54] (**Supplementary Figure 128**) with B3LYP/6-311+G(d,p) (also used for the geometries). For the NICS scans of **MP** and $\mathbf{Q}_{C2\mathrm{v}}$ the scans were conducted on both sides as these conformers are non-planar. The ACID plots show that **DA**, $\mathbf{Q}_{D2\mathrm{h}}$ and $\mathbf{Q}_{C2\mathrm{v}}$ are all antiaromatic, while **MP** exhibits a much smaller ring current. The NICS scans quantify this picture, giving **DA**, $\mathbf{Q}_{D2\mathrm{h}}$ and $\mathbf{Q}_{C2\mathrm{v}}$ as almost equally and strongly antiaromatic (90-110 ppm at the ring center), while **MP** exhibits a substantially lower antiaromaticity (45 ppm at the ring center).

Multiconfigurational character of benzene and PAHs

It has previously been shown through quantum-chemical calculations that the first singlet excited state of benzene,[55] naphthalene,[56] anthracene,[57] phenanthrene[58] and pyrene[59] are multiconfigurational. The question relevant to our study is if this also applies to the lowest triplet excited state. Calculations show that benzene in its lowest *vertically* excited triplet state is indeed described by two configurations with equal weight.[55] However, one configuration dominates for vertically excited naphthalene and anthracene.[56,57] As we use DFT calculations to study the *relaxed* triplet states, the important question to answer is if these are well-described by a single configuration? If this is not the case, the DFT results may not be trustworthy.

To answer this question, we carried out CASSCF[34] calculations with Molcas 8[37] and the ANO-RCC-VDZP basis set[36] on the vertically excited and relaxed triplet states of benzene, naphthalene, anthracene, phenanthrene. All π orbitals and π electrons were included in the active space. For pyrene we did a RASSCF(16in16)/(4in4)/2 calculation[60] due to the large number of electrons and orbitals. This means 6 orbitals and electrons in RAS1, 4 orbitals and electrons in RAS2, 6 orbitals and electrons in RAS3 and 2 holes/electrons allowed in RAS1/RAS3. Geometries were obtained with (U)B3LYP/6-311+G(d,p). We do not here consider fluoranthene and coronene as we have not been able to find high-level calculations for these compounds and they are too large for us to calculate. However, the results for these are expected to be similar to those of the smaller PAHs. The results listed in **Supplementary Table 9** clearly show that the relaxed triplet states are dominated by a single electronic configuration. Although benzene and phenanthrene have influence of two configurations at their vertically excited geometries, these geometries are not used for the DFT calculations. The weights calculated here for the vertically

excited states are in good agreement with the literatures values, which are available only for benzene,[55] naphthalene[56] and anthracene.[57] We conclude that the relaxed triplet states of the polyaromatic hydrocarbons in this study are not multiconfigurational and that DFT therefore should be an adequate tool for their description.

Connection of product distribution to ring currents and spin densities

The main products from the hydrogenations and hydrosilylations of benzene, naphthalene, anthracene and phenanthrene are consistent with the ACID plots in that the addition occurs at rings which show antiaromatic ring currents (reference to figure). For pyrene, addition instead occurs at C4 and C5, which are not involved in any antiaromatic ring. Similar conclusions can be drawn from the spin densities (**Supplementary Figure 129**) for benzene, anthracene and phenanthrene where addition occurs at the positions with the highest spin densities. For naphthalene, two hydrosilylated products are observed in roughly equal amounts by addition of silyl either to C1 or C2. As it is not clear if hydrogen or silyl is added first, the higher spin density at C1 (20%) than at C2 (6%) is still consistent with this result. For pyrene, addition occurs at C4 and C5 with lower spin density (8%) than C1 (18%). In summary, the observed products are consistent with the expectations from both ACID plots and spin densities, except for pyrene. The observation for pyrene indicates that other factors besides excited state antiaromaticity influence the selectivity. One plausible explanation is rearrangements to gain and maximize ground state aromaticity after return to S_0.

Electron transfer energies for triplet benzene and pyrene and $HSiEt_3$

To investigate if the photoreactions could be rationalized through excited state electron transfer either (1) from benzene or the PAHs to $HSiEt_3$ or (2) from $HSiEt_3$ to benzene or the PAHs, we calculated electron transfer energies for benzene and pyrene in T_1 with the G4 method.[61] The results (**Supplementary Figure 130**) show that both electron transfer processes are highly endergonic both for pyrene and benzene and we can therefore rule out this reaction mechanism.

Investigations in the mechanism of photohydrogenation of graphene

There is a previous study on the photo-functionalization of graphene (ref 62) in which the authors propose the mechanism of hot electron transfer from photoexcited graphene to organic molecules following excitation by a 532 nm laser source. Are our experimental findings also explained by such a mechanism? We have repeated these experiments with our lamp (having emission maxima at 448 nm and 563 nm) and find the same results: benzoyl peroxide is reactive while *tert*-butylperacetate is unreactive. We can explain this trend with the calculated density of states of graphene and the LUMO levels of benzoyl peroxide and *tert*-butylperacetate when physisorbed to graphene (**Supplementary Figure 131**). The light used in our experiments

(maximum at 448 nm = 2.77 eV and highest energy light at 403 nm = 3.08 eV) is sufficient to excite electrons in graphene to an energy at or above the LUMO of benzoyl peroxide, but not *tert*-butylperacetate, making electron transfer possible. This is consistent with the reactivity of the former and the non-reactivity of the latter.

We have performed the following experiments:

a) Graphene + benzoylperoxide + hv (White LEDs)

Procedure: Inside a glassy tube (borosilicate; 5 mm wide), a CVD grown graphene sample was immersed in a 5 mM benzoyl peroxide solution in acetone, capped with a rubber septum and then the liquid mixture was degassed with Ar for several minutes. After that the tube was placed in the center of a light source, a set-up containing LEDs (see section **Photochemical reactors and glassware**) and irradiated for 24 h. The distance from the light source was 38 mm). After this period the liquid mixture was removed from the tube and the sample was collected and washed carefully with dichloromethane. The sample was analyzed using XPS. It was shown that reaction did occur.

b) Graphene + *tert*-butylperacetate + hv (White LEDs)

Procedure: Inside a glassy tube (borosilicate; 5 mm wide), a CVD grown graphene sample was immersed in a in 50 % wt. *tert*-butylperacetate solution in mineral oil, capped with a rubber septum and then the liquid mixture was degassed with Ar for several minutes. After that the tube was placed in the center of a light source, a set-up containing LEDs (see section **Photochemical reactors and glassware**) and irradiated for 24 h. The distance from the light source was 38 mm. After this period the liquid mixture was removed from the tube and the sample was collected and washed carefully with *n*-pentane and then dichloromethane. The sample was analyzed using XPS. It was shown that no reaction occurred.

We have done *ab-initio* density functional based calculations for benzoyl peroxide, *tert*-butyl peracetate, triethylsilane and formic acid physisorbed on graphene using plane wave based VASP code. The generalized gradient approximation of Perdew, Burke and Ernzerhof has been used for the exchange-correlation potential. We have considered a 9 × 9 × 1 supercell of graphene for our calculations. Such a large graphene supercell was required to remove interaction between the molecules from the periodic images. Also, a 21 Å vacuum was kept in the out-of-plane direction to avoid any interactions from the periodic images. We have optimized the structures using conjugate gradient method with the forces calculated using Hellman-Feynman theorem. The energy and force convergence threshold were kept at 10^{-5} eV and 10^{-2} eV/Å respectively. For geometry optimization, we have used a 9 × 9 × 1 Monkhorst-Pack k-grid. A 11 × 11 × 1 Monkhorst-Pack k-grid was used for total energy and electronic structure calculations.

Now with reagard to Et_3SiH and HCOOH, the LUMOs of these are even higher than for tert-butyl peracetate, and it is thus very unlikely that the reaction of graphene and Et_3SiH which we observe goes by the hot electron transfer mechanism. This is further consistent with our calculated very endergonic electron transfer energies from benzene (137 kcal/mol) or pyrene (158 kcal/mol) to Et_3SiH calculated with the G4 method. In addition, hot hole transfer from graphene to the HOMO of any of the molecules is also unlikely due to the same reason that hot electron transfer is unlikely. Therefore, another photochemical mechanism must be operative; a mechanism which relies on the excited states of graphene.

Cis vs trans addition of hydrosilane to graphene

Please see Supplementary Table 10 and 11.

ACID Plots and NICS XY scans of Stone-Wales defects

We calculated the ACID plots and NICS-XY scans to assess the aromaticity of dicyclopenta[ef,kl]heptalene (also called azupyrene), the smallest model for a Stone-Wales defect in graphene. The ACID plots at CSGT-B3LYP/6-311+G(d,p)//B3LYP/6-311G(d,p) in S_0 and T_1 are given in **Supplementary Figure 135**. In S_0, a 14-electron diatropic (aromatic) ring current encircles the perimeter, while there are smaller contributions from 5-center diatropic currents in the five-membered rings. In the geometry-relaxed T_1 state, antiaromaticity is confined to the five-membered rings only and no aromatic currents are present. The conclusions from the ACID plots are fully confirmed by the NICS-XY scans at the GIAO-B3LYP/6-311+G(d,p)//B3LYP/6-311G(d,p) level (**Supplementary Figure 136**).

Supplementary References:

1 Singleton, D.L., Paraskevopoulos, G. & Irwin, R.S. UV absorption cross-sections of the monomer and dimer of formic acid. *J. Photochem.* **37**, 209-216 (1987).

2 Roberge, R., Sandorfy, C., Matthews, J. I. & Strausz, O. P. The far ultraviolet and HeI photoelectron spectra of alkyl and fluorine substituted silane derivatives. *J. Chem. Phys.* **69**, 5105- 5112 (1978).

3 Turro, N. J., Ramamurthy, V. & Scaiano, J. C. *Modern molecular photochemistry of organic molecules.*(University Science Books, 2010).

4 Zhao, J., Wu, W., Sun, J. & Guo, S. Triplet photosensitizers: from molecular design to applications. *Chem. Soc. Rev.*, **42**, 5323-5351 (2013).

5 Ruetten, S. A. & Thomas, J. K. Fluorescence and Triplet Quantum Yields of Arenes on Surfaces. *J. Phys. Chem. B*, **102**, 598-606 (1998).

6. Li, X., Cai, W., An, J., Kim, S., Nah, J., Yang, D., Piner, R., Velamakanni, A., Jung, I., Tutuc, E., Banerjee, S. K., Colombo, L. & Ruoff, R. S. Large-area synthesis of high-quality and uniform graphene films on copper foils. *Science*, **324**, 1312-1314 (2009).

7. Li, X., Zhu, Y., Cai, W., Borysiak, M., Han, B., Chen, D., Piner, R. D., Colombo, L. & Ruoff, R. S. Transfer of large-area graphene films for high-performance transparent conductive electrodes. *Nano Lett*, **9**, 4359–4363 (2009).

8. Liang, X., Sperling, B. A., Calizo, I., Cheng, G., Hacker, C. A., Zhang, Q., Obeng, Y., Yan, K., Peng, H., Li, Q., Zhu, X., Yuan, H., Hight Walker, A. R., Liu, Z., Peng, L.-m. & Richter, C. A. Toward clean and crackless transfer of graphene. *ACS Nano*, **5**, 9144–9153 (2011).

9. Battino, R., Rettich, T. R. & Tominaga, T. The solubility of oxygen and ozone in liquids. *J. Phys. Chem. Ref. Data*. **12**, 163-178 (1983).

10. Raymond, M. K. & Michl, J. *Int. J. Quant. Chem.*, **72**, 361-367 (1999).

11. Brix, T., Bastian, E. & Potzinger, P. *J. Photochem. Photobiol. A*, **49**, 287-297 (1989).

12. Gottlieb, H. E., Kotlyar, V. & Nudelman, A. NMR Chemical shifts of common laboratory solvents as trace impurities *J. Org. Chem.*, **62**, 7512–7515 (1997).

13. Chandross, E. A. & Ferguson, J. Photodimerization of Crystalline Anthracene. The Photolytic Dissociation of Crystalline Dianthracene. *J. Chem. Phys.*, **45**, 3564 (1966).

14 Breton, G. W. & Vang, X. Photodimerization of Anthracene. *J. Chem. Educ.*, **75**, 81-82 (1998).

15 Chatgilialoglu, C., Ingold, K. U. & Scaiano, J. C. Absolute rate constants for the addition of triethylsilyl radicals to various unsaturated compounds. *J. Am. Chem. Soc.* **105**, 3292-3296 (1983).

16 Oda, M., Kawase, T. & Kurata, H. 1,3,5-Cyclooctatriene. *Organic Syntheses*, **73**, 240-244 (1996).

17 Chapman, O. L., Borden, G. W., King, R. W. & Winkler, B. Photoisomerization of 1,3,5-cyclooctatriene. *J. Am. Chem. Soc.* **86**, 2660-2663 (1964).

18 Simonneau, A. & Oestreich, M. 3-Silylated cyclohexa-1,4-dienes as precursors for gaseous hydrosilanes: The B(C6F5)3-catalyzed transfer hydrosilylation of alkenes. *Angew. Chem. Int. Ed.*, **52**, 11905-11907 (2013).

19 Zarate, C. & Martin, R. A Mild Ni/Cu-Catalyzed Silylation via C–O Cleavage. *J. Am. Chem. Soc.*, **136**, 2236-2239 (2014).

20 Nogaideli, A. I., Tabashidze, T. I. & Barabadze, S. S. Reaction of dimethyl-, methylphenyl-, and diphenyldichlorosilanes with 9-lithiumphenanthrene. *Zhurnal Obshchei Khimii*, **41**, 1086-1089 (1971).

21 Gaussian 09, Revision D.01, Frisch, M. J., Trucks, G. W., Schlegel, H. B., Scuseria, G. E., Robb, M. A., Cheeseman, J. R., Scalmani, G., Barone, V., Mennucci, B., Petersson, G. A., Nakatsuji, H., Caricato, M., Li, X., Hratchian, H. P., Izmaylov, A. F., Bloino, J., Zheng, G., Sonnenberg, J. L., Hada, M., Ehara, M., Toyota, K., Fukuda, R., Hasegawa, J., Ishida, M., Nakajima, T., Honda, Y., Kitao, O., Nakai, H., Vreven, T., Montgomery, J. A. Jr., Peralta, J. E., Ogliaro, F., Bearpark, M., Heyd, J. J., Brothers, E., Kudin, K. N., Staroverov, V. N., Kobayashi, R., Normand, J., Raghavachari, K., Rendell, A., Burant, J. C., Iyengar, S. S., Tomasi, J., Cossi, M., Rega, N., Millam, J. M., Klene, M., Knox, J. E., Cross, J. B., Bakken, V., Adamo, C., Jaramillo, J., Gomperts, R., Stratmann, R. E., Yazyev, O., Austin, A. J., Cammi, R., Pomelli, C., Ochterski, J. W., Martin, R. L., Morokuma, K., Zakrzewski, V. G., Voth, G. A., Salvador, P., Dannenberg, J. J., Dapprich, S., Daniels, A. D., Farkas, Ö., Foresman, J. B., Ortiz, J. V., Cioslowski, J., & Fox, D. J. *Gaussian, Inc.*, Wallingford CT (2009).

22 Stephens, P. J., Devlin, F. J., Chabalowski, C. F. & Frisch, M. J. Ab initio calculation of vibrational absorption and circular dichroism spectra using density functional force fields. *J. Phys. Chem.*, **98**, 11623-11627 (1994).

23 Krishnan, R., Binkley, J. S., Seeger, R. & Pople, J. A. Self-consistent molecular orbital methods. XX. A basis set for correlated wave functions. *J. Chem. Phys.*, **72**, 650-654 (1980).

24. Herges, R. & Geuenich, D. Delocalization of electrons in molecules. *J. Phys. Chem. A*, **105**, 3214-3220 (2001).

25. Geuenich, D., Hess, K., Kohler, F. & Herges, R. Anisotropy of the induced current density (ACID), a general method to quantify and visualize electronic delocalization. *Chem. Rev.* **105**, 3758-3772 (2005).

26. Keith, T. A. & Bader, R. F. W. Calculation of magnetic response properties using a continuous set of gauge transformations. *Chem. Phys. Lett.*, **210**, 223-231 (1993).

27. Chai, J.-D. & Head-Gordon, M. Long-range corrected hybrid density functionals with damped atom-atom dispersion corrections. *Phys. Chem. Chem. Phys.*, **10**, 6615-6620 (2008).

28. Zhao, Y. & Truhlar, D. The M06 suite of density functionals for main group thermochemistry, thermochemical kinetics, noncovalent interactions, excited states, and transition elements: two new functionals and systematic testing of four M06-class functionals and 12 other functionals. *Theor. Chem. Acc.*, **120**, 215-241 (2008).

29 Adamo C. & Barone V. Toward reliable density functional methods without adjustable parameters: The PBE0 model. J. Chem. Phys. 110, 6158-6170 (1999).

30 GaussView, Version 5, Dennington, Roy; Keith, Todd; Millam, John. Semichem Inc., Shawnee Mission, KS, 2009.

31 Gershoni-Poranne R. & Stanger A. The NICS-XY-scan: Identification of local and global ring currents in multi-ring systems. *Chem. - Eur. J.* **20**, 5673-5688 (2014).

32 Wolinski K., Hinton J. F.& Pulay P. Efficient implementation of the gauge-independent atomic orbital method for NMR chemical shift calculations. *J. Am. Chem. Soc.* **112**, 8251-8260 (1990).

33 Stanger A. Obtaining relative induced ring currents quantitatively from NICS. *J. Org. Chem.* **75**, 2281-2288 (2010).

34 Roos B. O., Taylor P. R. & Siegbahn P. E. M. A complete active space SCF method (CASSCF) using a density matrix formulated super-CI approach. *Chem. Phys.* **48**, 157-173 (1980).

35 Andersson K., Malmqvist P.-Å. & Roos B. O. Second-order perturbation theory with a complete active space self-consistent field reference function. *J. Chem. Phys.* **96**, 1218-1226 (1992).

36 Roos B. O., Lindh R., Malmqvist P.-Å., Veryazov V. & Widmark P.-O. Main group atoms and dimers studied with a new relativistic ANO basis set. *J. Phys. Chem. A* **108**, 2851-2858 (2003).

37 Aquilante F. et al. Molcas 8: New capabilities for multiconfigurational quantum chemical calculations across the periodic table. *J. Comput. Chem.* **37**, 506-541 (2016).

38 Castro C., Karney W. L., McShane C. M. & Pemberton R. P. [10]Annulene: bond shifting and conformational mechanisms for automerization. *J. Org. Chem.* **71**, 3001-3006 (2006).

39 Bianchi R., Pilati T. & Simonetta M. Structure of 1,6-methano[10]annulene. *Acta Crystallogr., Sect. B: Struct. Crystallogr. Cryst. Chem.* **36**, 3146-3148 (1980).

40 Zhu J., An K. & Schleyer P. v. R. Evaluation of triplet aromaticity by the isomerization stabilization energy. *Org. Lett.* **15**, 2442-2445 (2013).

41 An K. & Zhu J. Evaluation of triplet aromaticity by the indene-isoindene isomerization stabilization energy method. *Eur. J. Org. Chem.*, **20**, 2764-2769 (2014).

42 Schleyer P. v. R. & Pühlhofer F. Recommendations for the evaluation of aromatic stabilization energies. *Org. Lett.* **4**, 2873-2876 (2002).

43. Jensen, F. Polarization consistent basis sets. IV. The basis set convergence of equilibrium geometries, harmonic vibrational frequencies, and intensities. *J. Chem. Phys.*, **118**, 2459-2463 (2003).

44. Jacob, C. R. & Reiher, M. Spin in density-functional theory. *Int. J. Quantum Chem.*, **112**, 3661-3684 (2012).

45. Gräfenstein, J. & Cremer, D. On the diagnostic value of $\langle \hat{S}^2 \rangle$ in Kohn-Sham density functional theory. *Mol. Phys.* **99**, 981-989 (2001).

46 Buma W. J., van der Waals J. H. & van Hemert M. C. Conformational instability of the lowest triplet state of the benzene nucleus. I. The unsubstituted molecule. *J. Chem. Phys.* **93**, 3733-3745 (1990).

47 Koseki S. & Toyota A. Energy component analysis of the pseudo-Jahn−Teller effect in the ground and electronically excited states of the cyclic conjugated hydrocarbons: cyclobutadiene, benzene, and cyclooctatetraene. *J. Phys. Chem. A* **101**, 5712-5718 (1997).

48 Ohno K. & Takahashi R. Excited-state vibrations of benzene and polycyclic aromatic hydrocarbons: simple force field models based on molecular orbital characteristics of hexagonal carbon networks. *Chem. Phys. Lett.* , 409-422 (2002).

49 Zamstein N., Kallush S. & Segev B. A phase-space approach to the T1$\rightsquigarrow$S0 radiationless decay in benzene: the effect of deuteration. *J. Chem. Phys.* **123**, 074304-1-074304-8 (2005).

50 Grimme S. Semiempirical hybrid density functional with perturbative second-order correlation. *J. Chem. Phys.* **124**, 034108-1-034108-16 (2006).

51 Curtiss L. A., Redfern P. C. & Raghavachari K. Gaussian-4 theory. *J. Chem. Phys.* **126**, 084108-1-084108-12 (2007).

52 Raghavachari K., Trucks G. W., Pople J. A. & Head-Gordon M. A fifth-order perturbation comparison of electron correlation theories. *Chem. Phys. Lett.* **157**, 479-483 (1989).

53 Dunning T. H. Gaussian basis sets for use in correlated molecular calculations. I. The atoms boron through neon and hydrogen. *J. Chem. Phys.* **90**, 1007-1023 (1989).

54 Stanger A. Nucleus-independent chemical shifts (NICS): distance dependence and revised criteria for aromaticity and antiaromaticity. *J. Org. Chem.* **71**, 883-893 (2006).

55 Hashimoto T., Nakano H. & Hirao K. Theoretical study of valence and Rydberg excited states of benzene revisited. *J. Mol. Struct.: THEOCHEM* **451**, 25-33 (1998).

56 Rubio M., Merchán M., Ortí E. & Roos B. O. A theoretical study of the electronic spectrum of naphthalene. *Chem. Phys.* **179**, 395-409 (1994).

57 Kawashima Y., Hashimoto T., Nakano H. & Hirao K. Theoretical study of the valence $\pi \rightarrow \pi^*$ excited states of polyacenes: anthracene and naphthacene. *Theor. Chem. Acc.* **102**, 49-64 (1999).

58 González-Luque R., Serrano-Andrés L., Merchán M.& Fülscher M. P. Theoretical characterization of the absorption spectra of phenanthrene and its radical cation. *Theor. Chem. Acc.* **110**, 224-232 (2003).

59 Park Y. H. & Cheong B.-S. Theoretical investigation of electronic structures of the ground and excited states of pyrene and its derivatives. *Curr. Appl. Phys.* **6**, 700-705 (2006).

60 Olsen J., Roos B. O., Jørgensen P. & Jensen H. J. A. Determinant based configuration interaction algorithms for complete and restricted configuration interaction spaces. *J. Chem. Phys.* **89**, 2185-2192 (1988).

61 Curtiss L. A., Redfern P. C. & Raghavachari K. Gaussian-4 theory. *J. Chem. Phys.* **126**, 084108 (2007).

62 Liu, H., Ryu, S., Chen, Z., Steigerwald, M. L., Nuckolls, C. & Brus, L. E. Photochemical Reactivity of Graphene. Photochemical Reactivity of Graphene. *J. Am. Chem. Soc.*, **131**, 17099–17101 (2009).
