# Supplementary material for: Metal-free photochemical silylations and transfer hydrogenations of benzenoid hydrocarbons and graphene
Source: Nat Commun. 2016 Oct 6;7:12962. doi: 10.1038/ncomms12962 (PMC5059713; doi:10.1038/ncomms12962)
Supplement: Peer review file [file ncomms12962-s2.pdf]

Editorial Note: this manuscript has been previously reviewed at another journal that is not operating a transparent peer review scheme. This document only contains reviewer comments and rebuttal letters for versions considered at Nature Communications.

REVIEWERS' COMMENTS:

Reviewer #3 (Remarks to the Author):

The manuscript "Metal-free photochemical silylations and transfer hydrogenations of benzene, polycyclic aromatic hydrocarbons and graphene" by Ottosson and co-authors for Nature Communications provides a meaningful aspects of aromaticity reversal in the lowest triplet states. They demonstrates silylation processes of polycyclic aromatic hydrocarbons (PAHs) using formic acid in the presence of LED light. This manuscript provides significant information for synthetic photochemistry as well as triplet aromaticity. I am highly impressed with the content of this manuscript. Moreover, I think that this is a very interesting paper, which should be in the interest of a very broad readership. Thus, it should be published in Nature Communications.
